# Identification and Molecular Characterization of MYB Transcription Factor Superfamily in C_4_ Model Plant Foxtail Millet (*Setaria italica* L.)

**DOI:** 10.1371/journal.pone.0109920

**Published:** 2014-10-03

**Authors:** Mehanathan Muthamilarasan, Rohit Khandelwal, Chandra Bhan Yadav, Venkata Suresh Bonthala, Yusuf Khan, Manoj Prasad

**Affiliations:** National Institute of Plant Genome Research, New Delhi, India; University of Delhi South Campus, India

## Abstract

MYB proteins represent one of the largest transcription factor families in plants, playing important roles in diverse developmental and stress-responsive processes. Considering its significance, several genome-wide analyses have been conducted in almost all land plants except foxtail millet. Foxtail millet (*Setaria italica* L.) is a model crop for investigating systems biology of millets and bioenergy grasses. Further, the crop is also known for its potential abiotic stress-tolerance. In this context, a comprehensive genome-wide survey was conducted and 209 MYB protein-encoding genes were identified in foxtail millet. All 209 *S. italica* MYB (SiMYB) genes were physically mapped onto nine chromosomes of foxtail millet. Gene duplication study showed that segmental- and tandem-duplication have occurred in genome resulting in expansion of this gene family. The protein domain investigation classified SiMYB proteins into three classes according to number of MYB repeats present. The phylogenetic analysis categorized SiMYBs into ten groups (I - X). *SiMYB*-based comparative mapping revealed a maximum orthology between foxtail millet and sorghum, followed by maize, rice and *Brachypodium*. Heat map analysis showed tissue-specific expression pattern of predominant SiMYB genes. Expression profiling of candidate MYB genes against abiotic stresses and hormone treatments using qRT-PCR revealed specific and/or overlapping expression patterns of *SiMYB*s. Taken together, the present study provides a foundation for evolutionary and functional characterization of MYB TFs in foxtail millet to dissect their functions in response to environmental stimuli.

## Introduction

In plants, transcription factors (TFs) play crucial role in regulating the gene expression and thereby guiding various biological processes including growth, development, cell division and response to stresses. Deciphering the molecular and physiological roles of these TFs has become hottest topic of research since understanding the control of gene expression would enable researchers to develop elite varieties of crop plants with desired agronomic traits which will ensure global food security in the scenario of climate change. Hence research on TFs had gained momentum and numerous TFs regulating vital biological processes in plants have been identified. MYB (myeloblastosis) TFs are omnipresent in eukaryotic systems and in plants was first identified in *Zea mays* involving the regulation of anthocyanin biosynthesis [1].

MYB proteins possess a highly conserved MYB DNA-binding domain throughout the eukaryotes at N-terminus and 1–4 imperfect repeats (R0, R1, R2, and R3) [2]. Each of these repeats contains 50–53 amino acids and encodes three α-helices, with second and third helices forming a helix–turn–helix (HTH) structure. Upon binding to DNA, HTH intercalates in the major groove [2], [3]. In addition, each MYB repeat comprises three regularly spaced tryptophan residues, which form a cluster in a hydrophobic core of the repeat and stabilize the structure of DNA-binding domain [4]. Contrarily, C-terminus is the activation domain and varies significantly between MYB proteins, which results in wide range of regulatory roles of MYB gene family [3], [5], [6].

There are numerous reports available demonstrating the role of MYB TFs in various plant processes such as secondary metabolism [7]–[10], hormone signal transduction [11], [12], environmental stresses [13]–[15], cell structuring and organ development [16]–[19]. The emergence of next-generation sequencing technologies and high-throughput analysis platforms had expedited genome sequencing projects in crop plants [20]. The availability of genome sequence information facilitated the crop research community to perform genome-wide analysis of TFs which would be more informative in conducting further research on functional characterization of candidate TFs. Of the various TFs, MYBs are most extensively studied in almost all land plants whose genome sequence is available [21]–[25], though there was no such genome-wide study on MYB TFs conducted in foxtail millet (*Setaria italica* L.).

Foxtail millet is a member of Poaceae family and it is the second most-widely cultivated millets, next to pearl millet (FAOSTAT 2012; http://faostat.fao.org/). Being a C_4_ panicoid crop, it is efficient in photosynthesis and better water use efficiency (WUE). Photosynthesis in C_4_ crops is more efficient than C_3_ plants even under high light intensity and high temperatures since phosphoenolpyruvate carboxylase (PEPC) helps in immediate uptake of carbon di oxide and delivering it to RuBisCO. The immediate quenching of carbon di oxide and delivery by PEPC does not require keeping stomata open for a long period and hence less water is lost by transpiration resulting in better WUE [26], [27]. Moreover, foxtail millet has been considered as a model crop for studying systems biology of biofuel crops due to its close phylogenetic relationships with several biofuel crops such as switchgrass (*Panicum virgatum*), napier grass (*Pennisetum purpureum*) and pearl millet (*Pennisetum glaucum*) [26], [27]. Hence considering its importance, BGI (Beijing Genomics Institute), China and the Joint Genome Institute (JGI) of Department of Energy, USA has sequenced the genome of two foxtail millet accessions [28], [29]. This motivated the present study, to perform (i) genome-wide identification of MYB TFs encoded in foxtail millet genome, (ii) protein and gene structure analysis using proteomic tools as well as phylogenetic analysis, (iii) promoter and miRNA analysis, (iv) *in silico* expression profiling using RNA-Seq data, (v) ortholog identification in related grass genomes, (vi) evolutionary analysis of orthologs and paralogs, and (vii) expression analysis of candidate *MYB* genes using qRT-PCR.

## Materials and Methods

### Identification, chromosomal location and gene duplication analysis of MYB proteins

The Hidden Markov Model (HMM) profile of MYB DNA-binding domain (PF00249) downloaded from Pfam v27.0 (http://pfam.sanger.ac.uk/family/PF00249) [30] was queried against Phytozome v9.1 (www.phytozome.net) of *Setaria italica*. All hits with expected values less than 1.0 were retrieved and compared with MYB protein dataset available in Foxtail millet Transcription Factor Database (http://59.163.192.91/FmTFDb/) [31]. In addition, *S. italica* MYB (SiMYB) proteins were examined using CDD search (http://www.ncbi.nlm.nih.gov/Structure/cdd/wrpsb.cgi) for presence of one or more MYB DNA-binding domains. The chromosomal location and identification of alternate transcripts of SiMYBs were performed by executing BLASTP search against the foxtail millet protein sequences in Phytozome and redundant sequences with different identification numbers and same chromosome locus were discarded. Based on ascending order of chromosomal position from short arm telomere to long arm telomere, *SiMYBs* were annotated as *SiMYB001* to *SiMYB209* and physical map was constructed using MapChart v2.2 [32]. The map was manually inspected for identifying tandem duplications. Adjacent genes of same sub-family located within 10 predicted genes apart or within 30 kb of each-other are considered as tandem duplicated genes [33]–[36]. Segmental duplications were predicted using Multiple Collinearity Scan toolkit (MCScanX) [37] as described by Puranik et al. [35] and Mishra et al. [36].

### Protein structure, gene organization and phylogenetic analysis

The presence of conserved domains in SiMYB proteins were analysed by employing hmmscan tool (http://hmmer.janelia.org/search/hmmscan). BioEdit tool (http://www.mbio.ncsu.edu/bioedit/bioedit.html) was used to perform multiple sequence alignment (MSA) of SiMYB amino acid sequences. The physicochemical properties of SiMYB proteins were then investigated using ExPASy ProtParam tool (http://web.expasy.org/protparam/). Gene Structure Display Server was used to identify the position of introns and exons in respective SiMYB genes (http://gsds.cbi.pku.edu.cn/) [38]. The SiMYB protein sequences were then imported into MEGA v6.06 [39] and MSA was performed using ClustalW with default parameters. The alignment file was used to construct neighbor-joining phylogenetic tree using a bootstrapping method with 1000 replicates.

### Gene Ontology annotation, promoter analysis and prediction of miRNAs targeting SiMYBs

The amino acid sequences of SiMYB are loaded in Blast2GO suite [40] to perform BLASTP, against *Oryza sativa* protein sequences of NCBI, with default parameters. The hits were mapped to retrieve GO terms and annotation of GO terms associated with BLAST hits was executed. For promoter analysis, 2 kb upstream sequences of SiMYB genes were retrieved from Phytozome through in-house perl programming and *cis*-regulatory elements were identified using database of Plant *Cis*-acting Regulatory DNA Elements (PLACE) [41]. Moreover, *Setaria italica* miRNAs (Sit-miRs) targeting *SiMYB*s were predicted by aligning Sit-miRs [42], [43] with *SiMYB* transcript sequences using psRNATarget [44].

### *In silico* expression profiling and marker localization

*Setaria italica* Illumina RNA-HiSeq reads for 4 tissues namely spica, stem, leaf and root were retrieved from European Nucleotide Archive [SRX128226 (spica); SRX128225 (stem); SRX128224 (leaf); SRX128223 (root)] [45]. The RNA-seq data was then filtered by NGS toolkit (http://59.163.192.90:8080/ngsqctoolkit/) [46] to remove low quality reads and mapped onto the gene sequences of *S. italica* using Bowtie2 [47]. The number of reads mapped was normalized by RPKM (reads per kilobase per million) method. The heat map showing tissue specific expression was generated on RPKM values for each gene in all the tissue samples using TIGR MultiExperiment Viewer (MeV4) software [48], [49]. The physical positions of DNA markers reported in foxtail millet [50] including simple sequence repeats (SSRs) [51], [52], EST-derived SSRs (eSSRs) [53] and intron length polymorphic markers (ILPs) [54], [55] were compared with the chromosomal location of SiMYB genes to identify the transcription factor gene-derived functional markers.

### Identification of SiMYB orthologs in related grass genomes and evolutionary analysis of paralogs and orthologs

SiMYB protein sequences were BLASTP searched against the peptide sequences of sorghum, maize, rice and *Brachypodium* (http://www.gramene.org/; http://www.phytozome.net/) to predict the MYB orthologs in these grass species. A reciprocal BLAST (BLASTP) was also performed to ensure unique relationship between the orthologous genes and all hits with E-value≤1e-5 and at least 80% homology were considered significant. The orthologous relationships between foxtail millet and these grass species were then visualized using Circos v0.55 (http://circos.ca/). The synonymous (Ks) and non-synonymous (Ka) substitution rates were estimated for paralog and ortholog MYB gene pairs through CODEML program in PAML interface tool of PAL2NAL (http://www.bork.embl.de/pal2nal/) [56]. Time (million years ago, Mya) of duplication and divergence of SiMYB gene pairs were estimated using a synonymous mutation rate of λ substitutions per synonymous site per year, as T = Ks/2λ, where, λ = 6.5×10^−9^ [35], [57], [58], [59].

### Plant materials and treatments

A salt and dehydration tolerant foxtail millet cultivar ‘Prasad’ [60], [61] was chosen for analysing the expression pattern of candidate *SiMYB*s. The seedlings were grown in a plant growth chamber (PGC-6L; Percival Scientific Inc., USA) for 21 d after germination at 28±1°C day/23±1°C night/70±5% relative humidity with a photoperiod of 14 h and a photosynthetic photon flux density of 500 µmol m^−2^ s^−1^. The plants were watered daily with one-third strength Hoagland's solution [62]. For stress and hormone treatments, 21-day-old seedlings were individually exposed to 250 mM NaCl (salinity), 20% PEG 6000 (dehydration), 100 µM abscisic acid (ABA), 100 µM methyl jasmonate (MJ) and 100 µM salicylic acid (SA) for 1 h (early) and 24 h (late) following previous reports [35], [61], [63]. After respective treatment, whole seedlings are immediately frozen in liquid nitrogen and stored at −80°C for RNA isolation. Untreated plants were maintained as control. The above experiments were repeated thrice to ensure precision and reproducibility.

### RNA isolation and quantitative real-time PCR analysis

Total RNA was isolated by following the method described by Longeman et al. [64] and treated with RNase-free DNase I (50 U/µl; Fermentas, USA). Its quality and purity was ensured using NanoDrop Spectrophotometer (Thermo Fisher Scientific, USA) [OD_260_: OD_280_ nm absorption ratio (1.8–2.0)] and integrity of RNA was checked by resolving on 1.2% agarose gel containing 18% formaldehyde. An amount of ∼1 µg total RNA was reverse transcribed to first strand cDNA using random primers by Thermo Scientific Verso cDNA kit (Thermo Fisher Scientific, USA) following manufacturer's instructions. The qRT-PCR primers were designed using GenScript Real-time PCR Primer Design tool (https://www.genscript.com/ssl-bin/app/primer) (Table S1). qRT-PCR analysis was performed in three technical replicates for each biological duplicate by StepOne Real-Time PCR Systems (Applied Biosystems, USA). The PCR mixtures and reactions were used as described previously by Kumar et al. [65] Melting curve analysis (60 to 95°C after 40 cycles) and agarose gel electrophoresis were performed to check amplification specificity. A constitutive *Act2* gene-based primer was used as endogenous control [65]. The amount of transcripts accumulated for SiMYB genes normalized to the internal control *Act2* were analysed using 2^−ΔΔCt^ method cDNA synthesis [65]. The PCR efficiency was calculated as: Efficiency = 10^(−1/slope)^−1 by the default software (Applied Biosystems).

### Homology modeling and active site prediction

SiMYB protein sequences were searched against Protein Data Bank (PDB) (http://www.rcsb.org/pdb/) for predicting the best template with homologous amino acid sequence and known structure. The result was then imported in Phyre2 server (Protein Homology/AnalogY Recognition Engine; http://www.sbg.bio.ic.ac.uk/phyre2) for predicting three-dimensional structures of proteins by homology modeling under ‘normal’ mode [66]. Active sites were precited using COACH server (http://zhanglab.ccmb.med.umich.edu/COACH/) and highlighted using UCSF Chimera 1.8.

## Results and Discussion

### Identification of SiMYB genes

The availability of foxtail millet whole genome sequence [28], [29] facilitated genome wide identification of MYB transcription factor (TF) gene family. The HMM search showed the presence of 229 MYB genes in foxtail millet genome (Table S2). The result was then confirmed by comparing the data with Foxtail millet Transcription Factor Database (FmTFDb; http://59.163.192.91/FmTFDb/) [31]. Further, all 229 SiMYB genes were BLAST searched against foxtail millet genome in Phytozome to identify alternate transcripts and chromosomal location. The results showed that 209 *SiMYBs* were primary transcripts and 20 were alternate transcripts. SiMYB017 had a maximum of three alternate transcripts, whereas SiMYB080, SiMYB137, SiMYB143 and SiMYB185 have two splice variants. Thirteen *SiMYBs* were evidenced to have one splice variant each.

The 209 SiMYB genes reported in the present study is higher than the numbers reported in Arabidopsis (197) [67], grape (118) [68] and Populus (192) [69]. On contrary, this number is lesser than soybean (244) [23] and apple (229) [70] (Figure S1). Among sequenced grass genomes, foxtail millet was evidenced to encode for a highest number of MYB TFs. Its closest relative sorghum has 100 MYB genes, whereas maize, rice and *Brachypodium* has 132, 155 and 98 MYB genes, respectively (http://planttfdb.cbi.pku.edu.cn/; Figure S1). Of note, further experimentations are requisite to identify the pseudogenes among the 209 SiMYBs.

### *SiMYBs* location and duplication in foxtail millet genome

**Figure 1:**
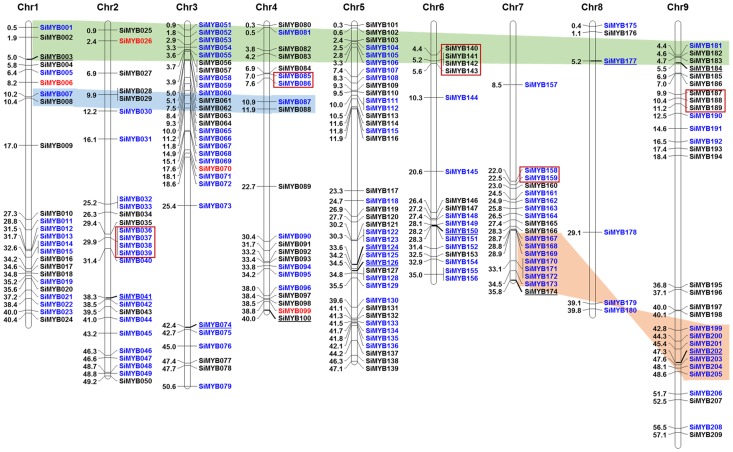
Physical map of 209 SiMYB genes. SiMYB genes were plotted onto nine chromosomes of foxtail millet. The numbers at left indicates the position of SiMYB genes, and the names of the respective genes are given in right. The ‘MYB-Related’ are given in black, ‘MYB-R2R3’ are in blue and ‘MYB-R1R2R3’ are in red. The SiMYB genes used for expression analysis through qRT-PCR are underlined. Segmentally duplicated gene-pairs are highlighted with different colours. Tandemly duplicated gene-pairs are enclosed within red boxes.

All the 209 SiMYB genes were physically mapped onto nine chromosomes of foxtail millet (Figure 1). The map revealed an uneven distribution of SiMYB genes in foxtail millet genome (Table S2). Highest number of SiMYB genes were observed in chromosome 5 (39; ∼19%) and lowest in chromosome 8 (6; ∼3%), with a density of 0.8 and 0.1 MYB genes/Mb, respectively. The distribution pattern of *SiMYBs* on individual chromosomes also revealed certain physical regions with a relatively higher accumulation of gene clusters. For instance, *SiMYBs* located on chromosomes 1, 3, 4, 5, 6 and 9 appear to be clustered at both upper end and lower end of the arms. Moreover, examination of gene duplication showed that SiMYB genes underwent both tandem and segmental duplications (Figure 1). Twelve gene-pairs were evidenced to be segmentally duplicated, whereas 15 genes were tandemly duplicated.

### Structural classification of SiMYB proteins

**Figure 2:**
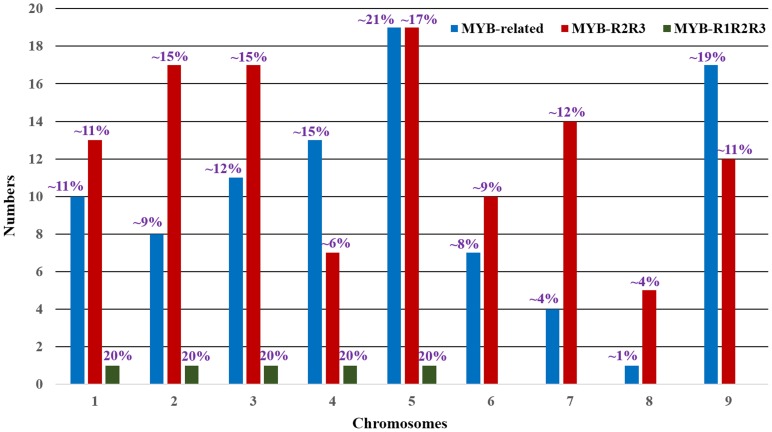
Chromosome-wise distribution of SiMYB genes. The distribution of three different SiMYB genes on individual chromosomes is shown.

The CDD search demonstrated the presence of conserved motifs in SiMYB proteins (Figure S2). It also showed the presence of more than one MYB repeats in some of these proteins along with the presence of additional domains. The hmmscan analysis provided comprehensive information on functional domains present in SiMYB proteins (Table S3). Based on the presence of one, two and three MYB repeats, the SiMYBs are classified into ‘MYB-related’, ‘MYB-R2R3’ and ‘MYB-R1R2R3’ (Table S2). The multiple sequence alignment of other three types of SiMYBs confirmed the presence of MYB repeats in the protein sequences (Figure S3–S5). Of the three types, ‘MYB-R2R3’ genes were found to be in maximum (114; ∼55%) followed by ‘MYB-related’ (90; ∼43%). Only 5 ‘MYB-R1R2R3’ were evidenced in foxtail millet distributed one MYB-R1R2R3 encoding gene each in chromosome 1 to 5 (Figs. 1, 2). The predominance of ‘MYB-R2R3’ type *SiMYB*s suggest that they could have been evolved from MYB-R1R2R3 gene progenitor through loss of R1 repeat or from a MYB-R1 gene through duplication of R1 repeat [71], [72]. Interestingly, no ‘Atypical MYB’ was identified in the foxtail millet, though one ‘Atypical MYB’ was reported in rice, two each in *Arabidopsis* and soybean [23], [67]. Among ‘MYB-R2R3’ and ‘MYB-related’ genes, highest numbers were found to be encoded in chromosome 5 (19 each). The lowest number of these genes are in chromosome 8 (5; ∼4% and 1; ∼1%, respectively) (Figs. 1, 2).

Further, hmmscan analysis also revealed the presence of specific, functionally important domains present in C-terminal regions outside MYB domain which are involved in transcriptional activity and protein-protein interactions (Table S3). SiMYB003, SiMYB094, SiMYB160 and SiMYB185 possess SWIRM domain. It is a small alpha-helical domain of about 85 amino acid residues found in chromosomal proteins. It contains a helix-turn-helix motif and binds to DNA [73]. ‘Response_reg’ is ‘response regulator receiver domain’, which was first identified to receive signal from the sensor partner in bacterial two-component systems. SiMYB004, SiMYB024, SiMYB082, SiMYB091, SiMYB093, SiMYB137, SiMYB193 and SiMYB207 have this domain. It is usually found at N-terminal to a DNA binding effector domain and its role in plant systems remain elusive [74]. The domain ‘P_C’ in SiMYB205 denotes ‘P protein C-terminus’, which represents C-terminus of plant P proteins. The maize P gene is a transcriptional regulator of genes encoding enzymes for flavonoid biosynthesis in the pathway leading to production of a red phlobaphene pigment [75] and P proteins are homologous to DNA-binding domain of MYB-like transcription factors [76]. The ‘ZZ’ domain is ZZ type-Zinc finger whose binding to DNA is not yet identified [77]. This domain is present in SiMYB094, SiMYB184 and SiMYB185. It has 4–6 cysteine residues in its sequence, which are responsible for coordinating zinc ions and to reinforce the structure [78]. SiMYB153 was evidenced to have ‘Bromodomain’, which is reported to recognize acetylated lysine residues. This recognition is prerequisite for protein-histone association and chromatin remodelling [79]. The domain ‘DUF3651’ is present in SiMYB055 and SiMYB106, which denotes ‘Domain of Unknown Function’. The ‘Linker_histone’ present in SiMYB110, SiMYB119, SiMYB127 and SiMYB188, which is an essential component of chromatin structure [80].

Except for the presence of conserved MYB domain, SiMYBs varied notably in size, sequence, intron-exon numbers and distribution and its physicochemical properties (Table S2). Gene structure analysis showed that 10 *SiMYB* were intron-less while *SiMYB026* and *SiMYB184* have maximum of 11 introns (Figure S6). The longest gene was *SiMYB094* with 8194 bases and the shortest was *SiMYB186* (348 bases). The longest protein sequence was SiMYB053 (1741 amino acids) and the least was SiMYB194 (98 amino acids) (Table S2). The enormous diversity with respect to MYB gene and proteins observed in foxtail millet was not reported in any other crop species [21]–[25], and this shows the presence of putative novel variants.

### Phylogenetic classification of SiMYB proteins

**Figure 3:**
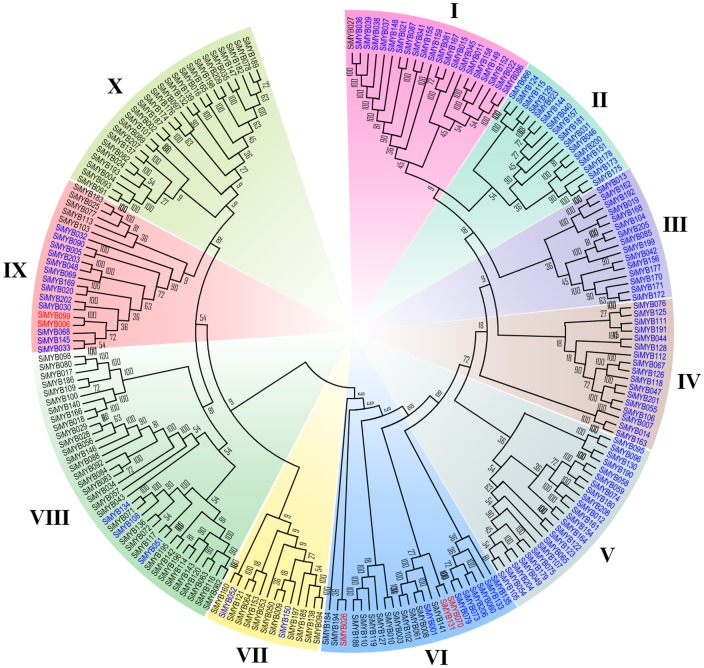
Phylogenetic relationships of foxtail millet MYB transcription factors. The unrooted phylogenetic tree was constructed by neighbor-joining method with 1000 bootstrap replicates. The bootstrap values are shown at the nodes. The tree was divided into ten phylogenetic cluster designated as I to X.

The phylogenetic tree constructed using neighbour-joining (NJ) method for all 209 SiMYB proteins conformed to the phylogenetic classification enlisted in FmTFDb (Figure 3). The internal branches were detected to have high bootstrap values and this lead to the derivation of statistically reliable pairs of possible homologous proteins possessing similar functions from a common ancestor. Based on topology and clade support values, the phylogenetic tree was divided into ten clades (I to X) (Figure 3). Although SiMYB proteins tended to cluster together in the tree with respect to their type and they were not equally distributed in the clades which may be due to the occurrence of duplication and divergence of SiMYB genes (Figure 3).

### Gene Ontology annotation

**Figure 4:**
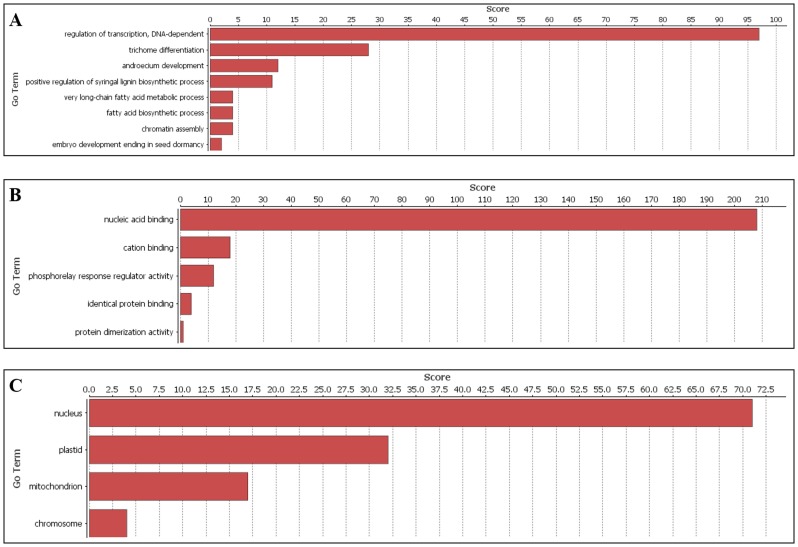
Gene Ontology distribution for SiMYB proteins. Blast2GO program defines the gene ontology under three categories. (**A**) biological processes, (**B**) molecular functions and (**C**) cellular component.

Gene Ontology (GO) annotation for all 209 SiMYB proteins was performed using Blast2GO. The SiMYB protein sequences were BLAST searched against *Oryza sativa* protein database and its biological processes, molecular functions and cellular components were identified (Figure 4). The results indicate that SiMYB proteins participate in diverse biological and molecular functions in the cell (Table S4). Noteworthy, predominant of SiMYBs were predicted to function in regulation of transcription in a DNA-dependent manner, followed by playing pivotal role in trichome differentiation and androecium development. SiMYBs are reported to least participate in breaking seed dormancy (Figure 4). The biological function analysis also revealed that 68 (32.5%) SiMYB proteins function in response to stress (Table S4). The molecular role showed that most of SiMYBs have function in nucleic acid binding followed by cation binding. Cellular component data predicted that SiMYBs are nuclear localized. In addition, SiMYB proteins were also indicated to localize in plastids and mitochondria (Figure 4). In addition, Blast2GO was performed to correlate the domain composition of families/sub-families with functional classes, but no correlation was observed.

### *Cis*-acting elements and *SiMYB* targeting miRNAs

The promoter analysis identified ∼300 diverse *cis*-acting elements upstream of *SiMYB* genes (Table S5). Of these, ARR1AT, CAATBOX1, CACTFTPPCA1, DOFCOREZM, EBOXBNNAPA, GATABOX, GT1CONSENSUS, GTGANTG10, MYCCONSENSUSAT, POLLEN1LELAT52, WBOXNTERF3 and WRKY71OS were present in all 209 *SiMYB* genes. ARR1AT (NGATT) is a cytokinin response regulator binding motif [81] and CAATBOX1 (CAAT) are reported to regulate flowering [82]. CACTFTPPCA1 (CACGTG) is responsible for mesophyll-specific gene expression of C4 phosphoenolpyruvate carboxylase gene in C_4_ plants [83] and DOFCOREZM (AAAG) is the binding cite of Dof transcription factors [84]. EBOXBNNAPA (CANNTG) is E-box sequence responsible for light responsiveness and is regulated by bHLH and MYB-transcription factor in directing tissue-specific expression [13]. GATABOX (GATA) is the binding site for transcription factors with a zinc finger motif [85], GT1CONSENSUS (GRWAAW) recognizes GT-1 proteins which have tri-helix DNA-binding domains [86] and GTGANTG10 (GTGA) is a pollen-specific *cis*-elements [87]. MYCCONSENSUSAT (CANNTG) is a MYC recognition site which regulates transcription of genes under cold conditions by a MYC-like bHLH transcriptional activator [88], POLLEN1LELAT52 (AGAAA) is a regulatory element responsible for pollen-specific activation of gene expression [89], WBOXNTERF3 (TGACY) is a W-box promoter motif functioning in response to wound signal [90] and WRKY71OS (TGAC) is a binding site of rice WRKY71, a transcriptional repressor of gibberellin signaling pathway [91]. In addition to these, some *cis*-elements were present unique to specific *SiMYBs* such as AAGACGTAGATACL12 in *SiMYB167*, ABADESI1 in *SiMYB025*, ABADESI2 in *SiMYB110*, ABRE3HVA22 in *SiMYB158* and ABREDISTBBNNAPA in *SiMYB122*. Further, *Setaria italica* miRNAs (Sit-miRs) targeting the *SiMYB* transcripts for post-transcriptional gene regulation were also identified. Three Sit-miRs namely Sit-miR5568.58, Sit-miR2118d and Sit-miR159a were evidenced to target *SiMYB026*, *SiMYB028*, *SiMYB029*, *SiMYB130* and *SiMYB190* (Table 1). This data would assist in deciphering post-transcriptional control of gene regulation during physiological and stress-induced cellular responses.

**Table 1:** List of *Setaria italica* miRNAs targeting *SiMYB* transcripts.

miRNA ID	Target.	E-value	UPE	Target start	Target end	miRNA aligned fragment	Target aligned fragment	Inhibition
Sit-miR5568.58	SiMYB026	3.0	16.26	1923	1943	UUUCUAGGUUUAUAUCUUUUG	CACAAGAGAUAGAUCUAGAAA	Cleavage
Sit-miR2118d	SiMYB028	2.5	14.91	150	171	UUCCUGAUGCCUCUCAUUCCUA	UUGGAAGGAGAGGCAUCAGGUA	Cleavage
Sit-miR2118d	SiMYB029	2.5	13.89	152	171	UUCCUGAUGCCUCUCAUUCC	GGAAGGAGAGGCAUCAGGUA	Cleavage
Sit-miR159a	SiMYB130	2.5	16.88	960	979	UUUGGAUUGAAGGGAGCUCU	GGAGCUCCCUUCACUCCAAG	Cleavage
Sit-miR159a	SiMYB190	3.0	19.88	884	904	UUUGGAUUGAAGGGAGCUCUG	UGGAGCUCCCUUCAAACCAAU	Cleavage

UPE - unpaired energy.

### Heat map analysis and identification of transcription factor-derived markers

**Figure 5:**
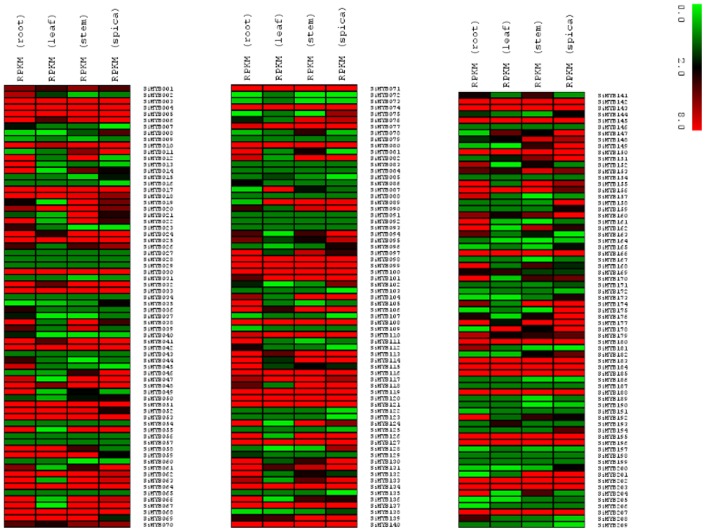
Heat map representation of SiMYB genes in four tissues. The Illumina RNA-seq data were re-analyzed and heat map was generated. Bar at the top represents log_2_ transformed values, thereby values 0.0, 2.0 and 8.0 represent low, intermediate and high expression, respectively.

The publicly available Illumina RNA-Seq data for four tissues of foxtail millet root, leaf, stem and spica were used to analyse the expression pattern of SiMYB genes. The heat map showed differential expression of *SiMYBs* across the tissues (Figure 5). Predominant of *MYB* genes including *SiMYB003*, *SiMYB004*, *SiMYB005*, *SiMYB017*, *SiMYB025*, *SiMYB051*, *SiMYB064*, *SiMYB080*, *SiMYB089*, *SiMYB090*, *SiMYB0100* were predicted to be highly expressed in all the tissues. Interestingly, tissue-specific higher expression was also noted. *SiMYB007* and *SiMYB087* were highly expressed in leaf, while *SiMYB192* showed higher expression in root (Figure 5). This expression profiling would facilitate combinatorial usage of *SiMYBs* in transcriptional regulation of different tissues, whereas ubiquitously expressed *SiMYBs* might regulate the transcription of a broad set of genes. Further, this heat map data also enables selection and designing of studies to impart stress tolerance in both foxtail millet and related crop species.

In addition, molecular markers present in *SiMYBs* were also identified. Seventy three SiMYB genes (∼35%) were evidenced to possess DNA markers (Table S6). Of the various types of markers reported in foxtail millet, presence of microsatellites (SSRs and eSSRs) in *SiMYB*s were found predominant (∼90%) followed by ILP markers (∼10%). Among the SSRs (63), tri-nucleotide repeats were maximum (41) followed by di-nucleotide repeats (20). Only two eSSRs namely SieSSR302 and SieSSR70 were found to be present in SiMYB030 and SiMYB094, respectively, and both the markers were of tri-nucleotide repeat type. SSRs SiGMS12780 and SiGMS8287 were a tetra- and hexa-nucleotide repeats, evidenced to be present in SiMYB179 and SiMYB125, respectively (Table S6). The functional relevance of these informative genic markers needs to be demonstrated by integrating agronomic trait association analysis with genetic mapping, differential expression profiling, protein modelling and haplotype gene evolution study in relation to *MYB* genes.

### SiMYB orthologs in sorghum, maize, rice and *Brachypodium*

**Figure 6:**
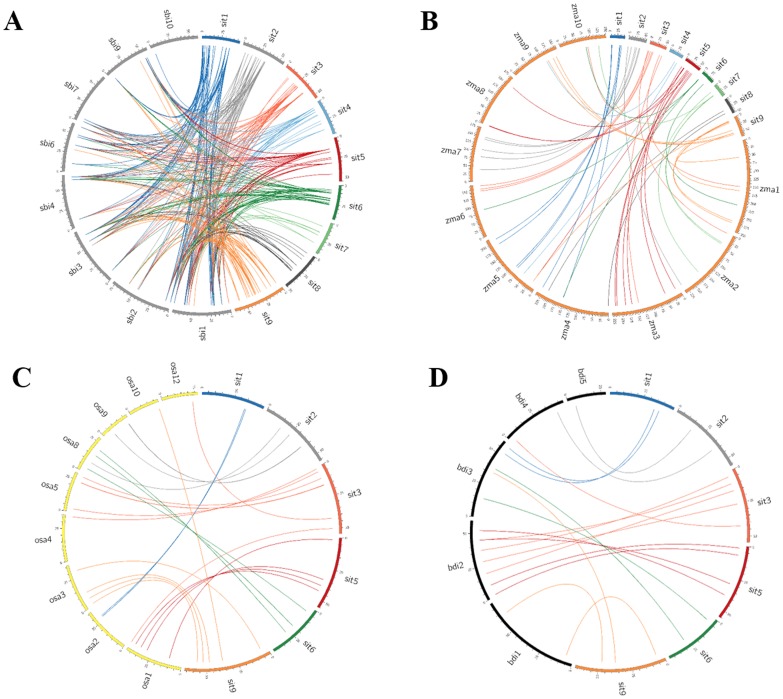
Comparative physical mapping revealed high degree of orthologous relationships of SiMYB genes located on nine chromosomes of foxtail millet. (**A**) *Sit* (foxtail millet) – *Sbi* (sorghum), (**B**) *Sit* – *Zma* (maize), (**C**) *Sit* – *Osa* (rice), and (**D**) *Sit* – *Bdi* (*Brachypodium*).

Of the 209 SiMYB genes, 72 (∼34%) showed significant orthologous relationships with sorghum, 55 (∼26%) with maize, 23 (∼11%) with rice and 13 (∼6%) with *Brachypodium* (Figure 6; Tables S7–S10). Foxtail millet chromosome 5 showed highest synteny with sorghum chromosome 3 (14; ∼19%), and maize chromosomes 3 and 8 (11; 20%). Chromosome 3 of foxtail millet has highest orthologous pairs with rice chromosomes 4, 5 and 12 (∼22%), and *Brachypodium* chromosomes 2, 4 and 5 (∼38%). Except the presence of conserved nucleotide and amino acid sequences of MYB domain between foxtail millet-rice and foxtail millet-*Brachypodium*, the regions other than the MYB domain were highly diverse. The degree of decrease in orthologous relationships among foxtail millet-sorghum, -maize, -rice and *Brachypodium* is due to genetic relatedness of these genomes. Foxtail millet along with sorghum and maize belong to Panicoideae, whereas rice and *Brachypodium* belong to Ehrhartoideae and Pooideae, respectively. This pattern of decreasing synteny was also observed in the comparative mapping of NAC TFs [35], WD40 genes [36], Dicer-like, Argonaute, RNA-dependent RNA polymerase genes [92], and C_2_H_2_-type zinc finger TFs [59]. This comparative map would enable the researchers to select candidate MYB genes from foxtail millet and utilize them in genetic enhancement of related grass family members.

### Evolutionary aspects of duplication and divergence

**Figure 7:**
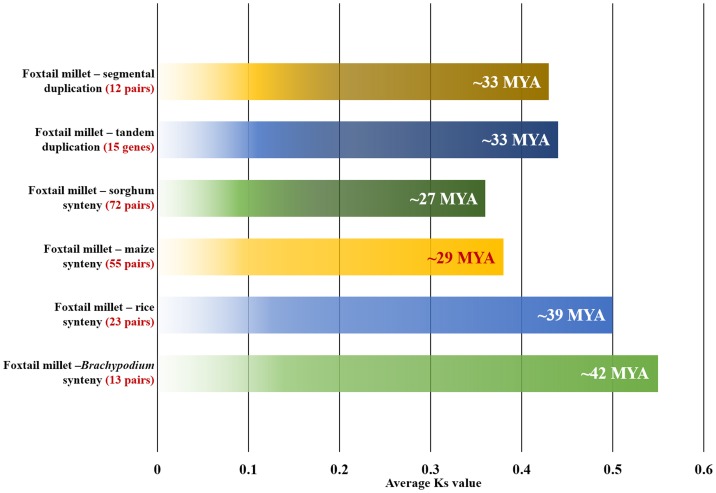
Time of duplication and divergence (MYA) based on synonymous substitution rate (Ks) estimated using duplicated SiMYB gene-pairs of foxtail millet and orthologous *MYB* gene pairs between foxtail millet- sorghum, -maize, -rice and *Brachypodium*.

The ratios of non-synonymous (Ka) versus synonymous (Ks) substitution rate (Ka/Ks) were estimated for duplicated and orthologous gene-pairs of *SiMYB*. The ratios of Ka/Ks for segmentally and tandemly duplicated gene-pairs ranged from 0.02 to 0.1 and 0.07 to 0.1, respectively (Tables S11–S12). The estimated Ka/Ks was <1, and this justified that the duplicated SiMYB genes were under strong purifying selection pressure. This is in agreement with previous genome-wide reports on NAC TFs by Puranik et al. [35], WD40 gene family by Mishra et al. [36], and C_2_H_2_-type zinc finger TFs by Muthamilarasan [59]. In addition, it was also estimated that the duplication event of these segmentally and tandemly duplicated genes occurred ∼33 Mya (Figure 7). Although further experimentations are necessary to validate this, the present study suggests that these duplication events must be specific to *Setaria* since divergence of sorghum and maize had occurred ∼13 Mya [28], [29].

Among orthologous gene-pairs of *SiMYB*, average Ka/Ks value was maximum between foxtail millet and *Brachypodium* followed by foxtail millet and rice (Figure 7). The Ka/Ks was minimum for foxtail millet-sorghum gene-pairs. The relatively higher rate of synonymous substitution between *Brachypodium* and foxtail millet *MYB* genes suggested their earlier divergence around 42 Mya. The evolutionary analysis also predicts the divergence time of foxtail millet-rice as ∼39 MYA, foxtail millet-maize as ∼29 MYA and foxtail millet-sorghum as ∼27 MYA (Figure 7). These Ka/Ks dating data along with comparative map would assist in understanding the evolution of MYB genes in grasses genomes.

### Expression pattern of *SiMYB*s under abiotic stress and hormone treatments

**Figure 8:**
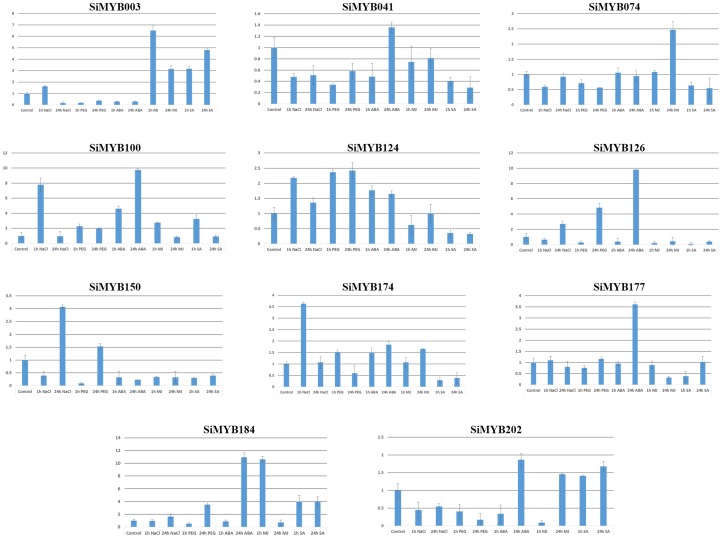
The relative expression ratio of 11 candidate SiMYB genes analysed using qRT-PCR under salinity stress, dehydration stress and hormone treatments for 1 h (early) and 24 h (late). The relative expression ratio of each gene was calculated relative to its expression in control sample (0 h). *Act2* was used as an internal control to normalize the data. Error bars representing standard deviation were calculated based on three technical replicates for each biological duplicate. *ABA - abscisic acid, MJ - methyl jasmonate, SA - salicylic acid.*

To study the expression patterns of identified *SiMYBs*, 11 candidate SiMYB genes were chosen representing all the nine chromosomes of foxtail millet and all the 10 clades of phylogenetic tree. The relative transcript abundance of these candidate *MYBs* at both early (1 h) and late (24 h) duration of abiotic stresses (salinity and dehydration), and hormone treatments (ABA, MJ, SA) were studied using qRT-PCR (Figure 8). During early phase of salinity stress *SiMYB003*, *SiMYB100*, *SiMYB124* and *SiMYB174* were highly up-regulated whereas *SiMYB041*, *SiMYB074*, *SiMYB126*, *SiMYB150* and *SiMYB202* were down-regulated. Though *SiMYB100* and *SiMYB174* showed significant up-regulation during early hours of salinity stress, a drastic down-regulation was observed at late phase. *SiMYB150* showed ∼2 fold higher expression during late phase of salinity stress. In course of dehydration stress, up-regulation during early phase was evidenced in *SiMYB074*, *SiMYB100*, *SiMYB174* and *SiMYB202*. SiMYB124 showed higher expression at both early and late phases. A drastic up-regulation of SiMYB genes at 24 h after dehydrations stress was noticed in *SiMYB126, SiMYB110* and *SiMYB184*.

During hormone treatments, *SiMYB126*, *SiMYB150* and *SiMYB177* were down-regulated in all the treatments and time points, except a considerable higher expression of *SiMYB126* and *SiMYB177* at 24 h post ABA treatment. *SiMYB003* was down-regulated at both the phases of ABA treatment, while significant up-regulation at 24 h post ABA application was observed in *SiMYB184* and *SiMYB202*. The genes *SiMYB003, SiMYB100* and *SiMYB184* were up-regulated during early phase of MJ treatment, whereas up-regulation was observed at late phase in *SiMYB074* and *SiMYB202*. Up-regulation of *SiMYB100* and *SiMYB184* was evidenced in early phase of SA treatment. After 24 h, *SiMYB003* and *SiMYB202* showed higher expression.

Since foxtail millet is well known for its adaptability to abiotic stresses such as dehydration and salinity [60]–[62], deciphering the role of MYB TFs will provide novel insights into the stress tolerance mechanism of this model crop. This preliminary study on expression profiling of candidate SiMYB genes in response to different stress and hormone treatments would serve as a foundation for downstream characterization of these TFs.

### Homology modelling of SiMYB proteins

Three dimensional protein models were constructed for all 209 SiMYBs and of note, the proteins were modelled at 90% confidence (Table S13). Further, its potential active sites were also identified (Figure S7). As mentioned earlier, the SiMYBs were classified into ‘MYB-related’, ‘MYB-R2R3’ and ‘MYB-R1R2R3’ based on the presence of one, two and three MYB repeats in the N-terminus. The homology modelling showed that each MYB repeat comprised of 52 amino acids and encoded three α-helices, with the second and third helices forming a helix-turn-helix (HTH) structure. Further, it included three regularly spaced tryptophan residues, forming a hydrophobic core in the three dimensional HTH structure (Figure S7). The third helix of each repeat formed ‘recognition helix’, which was reported to interact with DNA and intercalate in the major groove [6]. Contrarily, the C-terminus varied considerably between SiMYB proteins and this could be responsible for the wide range of regulatory roles of SiMYB gene family. This preliminary data would provide a base for further molecular structure analyses and interaction studies of SiMYB proteins.

In summary, the present study identified 209 MYB proteins in model crop foxtail millet and physically mapped them onto the genome. The protein structure and gene organization of individual SiMYBs were analysed and a phylogenetic tree was constructed. The domain analysis showed the presence of diverse protein domains in MYB proteins and the multiple sequence alignment revealed the conserved sequences in these domains. In addition, DNA markers present in *SiMYB* genes were also investigated. Candidate *SiMYB*s were chosen for expression profiling through qRT-PCR and the analysis showed the role of these genes in response to abiotic stresses and hormone treatments. The present study is the first report on genome-wide identification, characterization and expression profiling of MYB transcription factors in foxtail millet and promisingly, the data obtained from this study would contribute to a better understanding of the complexity of MYB TFs in plants and provide a useful reference for further functional analysis of MAPK genes in foxtail millet and related grasses.

## Supporting Information

Figure S1 **Phylogenetic relationships between all the sequenced plant species.** The total number of MYB proteins found in each genome is indicated on the right. The data was retrieved from PlantTFDB (http://planttfdb.cbi.pku.edu.cn/). The data of foxtail millet (*Setaria italica*) excludes alternate transcripts.
(PDF)

Figure S2 **Conserved domains in SiMYB proteins, identified using CDD database.**
(PDF)

Figure S3 **The multiple sequence alignment of ‘MYB-related’ proteins.**
(PDF)

Figure S4 **The multiple sequence alignment of ‘MYB-R2R3’ proteins.**
(PDF)

Figure S5 **The multiple sequence alignment of ‘MYB-R1R2R3’ proteins.**
(PDF)

Figure S6 **Gene structures of SiMYB proteins. Exons and introns are represented by green boxes and black lines, respectively.**
(PDF)

Figure S7 **Predicated three dimensional structures of all the 209 SiMYB proteins.**
(PDF)

Table S1 **Details of primers used for quantitative real-time PCR.**
(DOC)

Table S2 **Characteristic features of 209 MYB Transcription factor gene family members identified in** ***Setaria italica*** **.**
(XLS)

Table S3 **Summary of functional domains present in the 209 SiMYB proteins.**
(XLS)

Table S4 **Blast2GO annotation details of SiMYB protein sequences.**
(XLS)

Table S5 **Characteristics of the promoter region of SiMYB genes.**
(XLS)

Table S6 **Details of DNA markers present in SiMYB genes.**
(XLS)

Table S7 **The Ka/Ks ratio, estimated divergence time and percentage identity for orthologous SiMYB proteins between foxtail millet and sorghum.**
(XLS)

Table S8 **The Ka/Ks ratio, estimated divergence time and percentage identity for orthologous SiMYB proteins between foxtail millet and maize.**
(XLS)

Table S9 **The Ka/Ks ratio, estimated divergence time and percentage identity for orthologous SiMYB proteins between foxtail millet and rice.**
(XLS)

Table S10 **The Ka/Ks ratio, estimated divergence time and percentage identity for orthologous SiMYB proteins between foxtail millet and** ***Brachypodium*** **.**
(XLS)

Table S11 **The Ka/Ks ratios and estimated divergence time for segmentally duplicated SiMYB genes.**
(DOC)

Table S12 **The Ka/Ks ratios and estimated divergence time for tandemly duplicated SiMYB genes.**
(DOC)

Table S13 **List of templates used in homology modelling of 209 SiMYB proteins.**
(XLS)
