# Supplementary figures and images for: Identification and Molecular Characterization of MYB Transcription Factor Superfamily in C4 Model Plant Foxtail Millet (Setaria italica L.)
Source: PLoS One. 2014 Oct 3;9(10):e109920. doi: 10.1371/journal.pone.0109920 (PMC4184890; doi:10.1371/journal.pone.0109920)

## SPECIES

## Number of MYBs

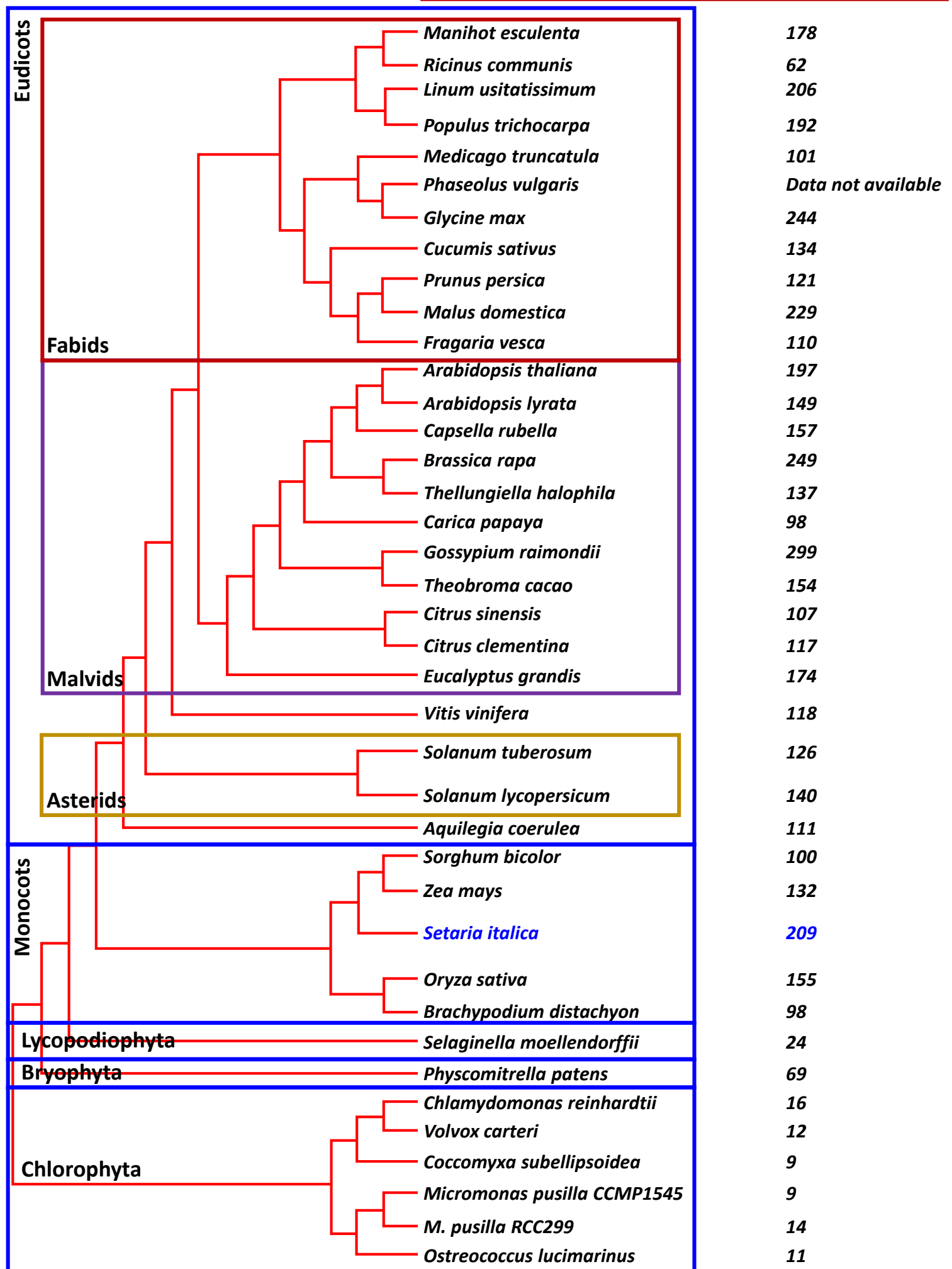

Supplement: Figure S1 — Phylogenetic relationships between all the sequenced plant species. The total number of MYB proteins found in each genome is indicated on the right. The data was retrieved from PlantTFDB (http://planttfdb.cbi.pku.edu.cn/). The data of foxtail millet (Setaria italica) excludes alternate transcripts. (PDF) [file pone.0109920.s001.pdf]

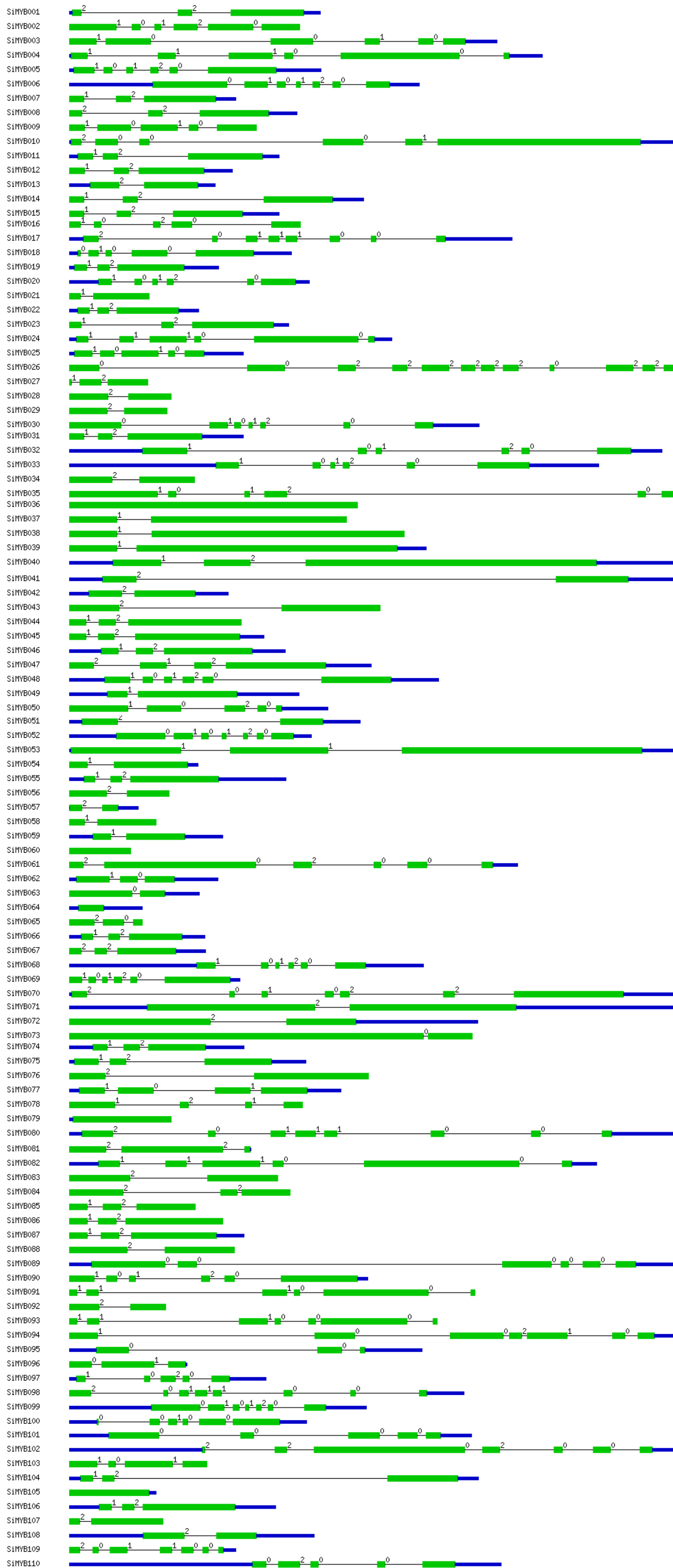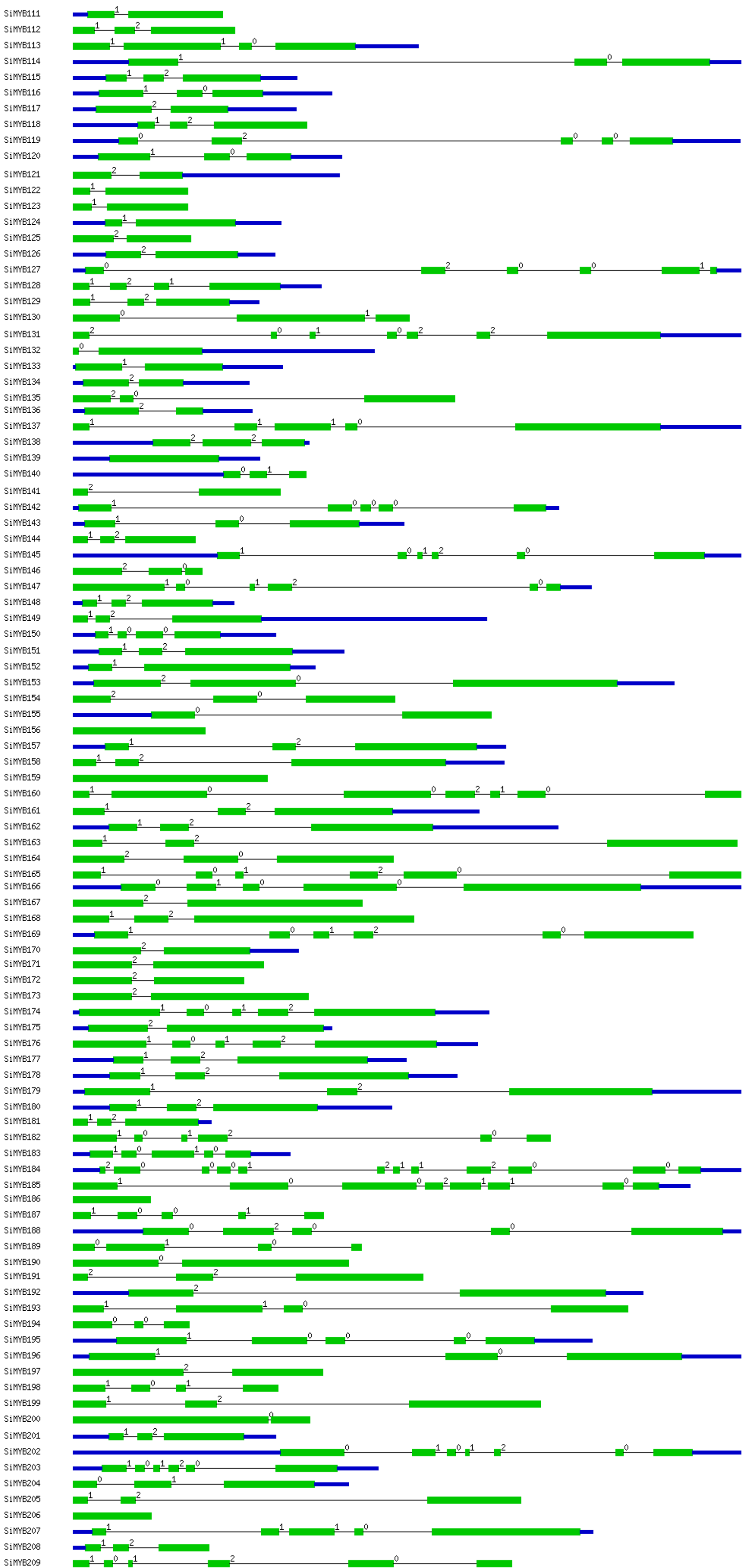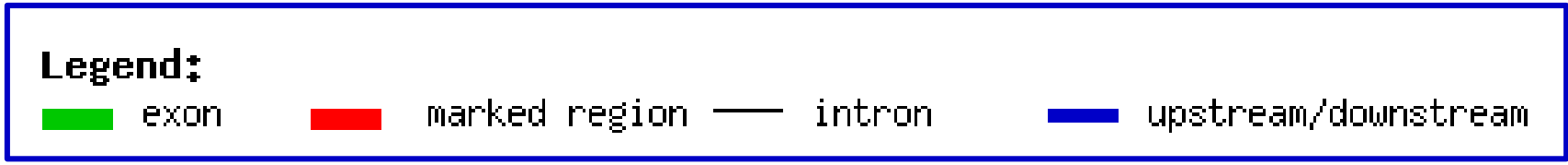

Supplement: Figure S6 — Gene structures of SiMYB proteins. Exons and introns are represented by green boxes and black lines, respectively. (PDF) [file pone.0109920.s006.pdf]
