# Supplementary material for: Identification and Molecular Characterization of MYB Transcription Factor Superfamily in C4 Model Plant Foxtail Millet (Setaria italica L.)
Source: PLoS One. 2014 Oct 3;9(10):e109920. doi: 10.1371/journal.pone.0109920 (PMC4184890; doi:10.1371/journal.pone.0109920)
Supplement: Figure S2 — Conserved domains in SiMYB proteins, identified using CDD database. (PDF) [file pone.0109920.s002.pdf]

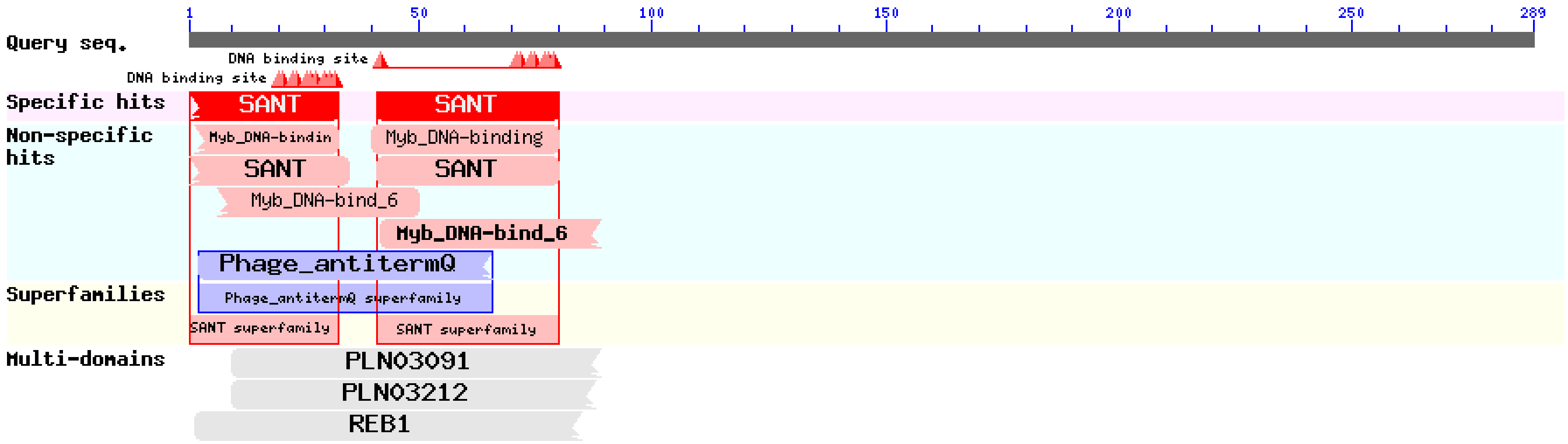

SiMYB001

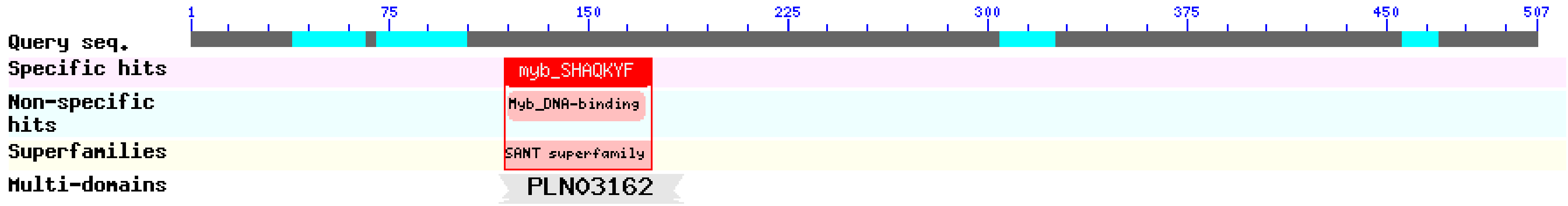

SiMYB002

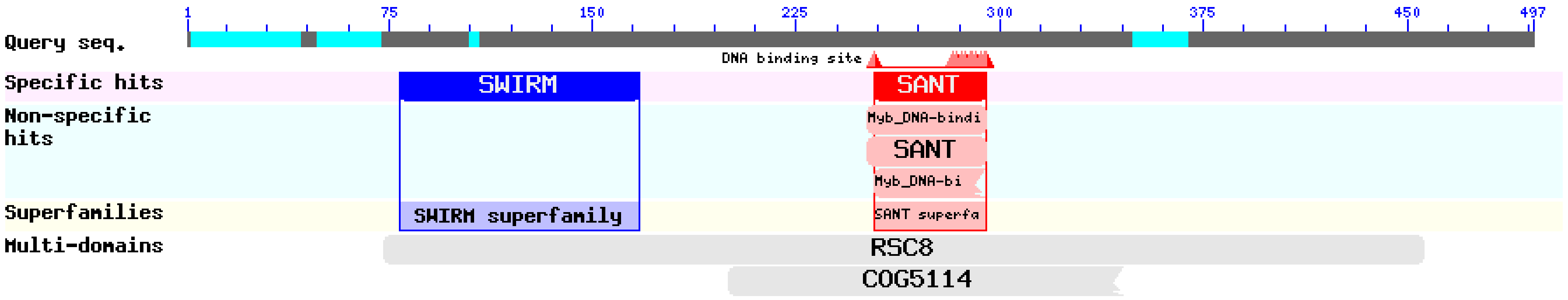

SiMYB003

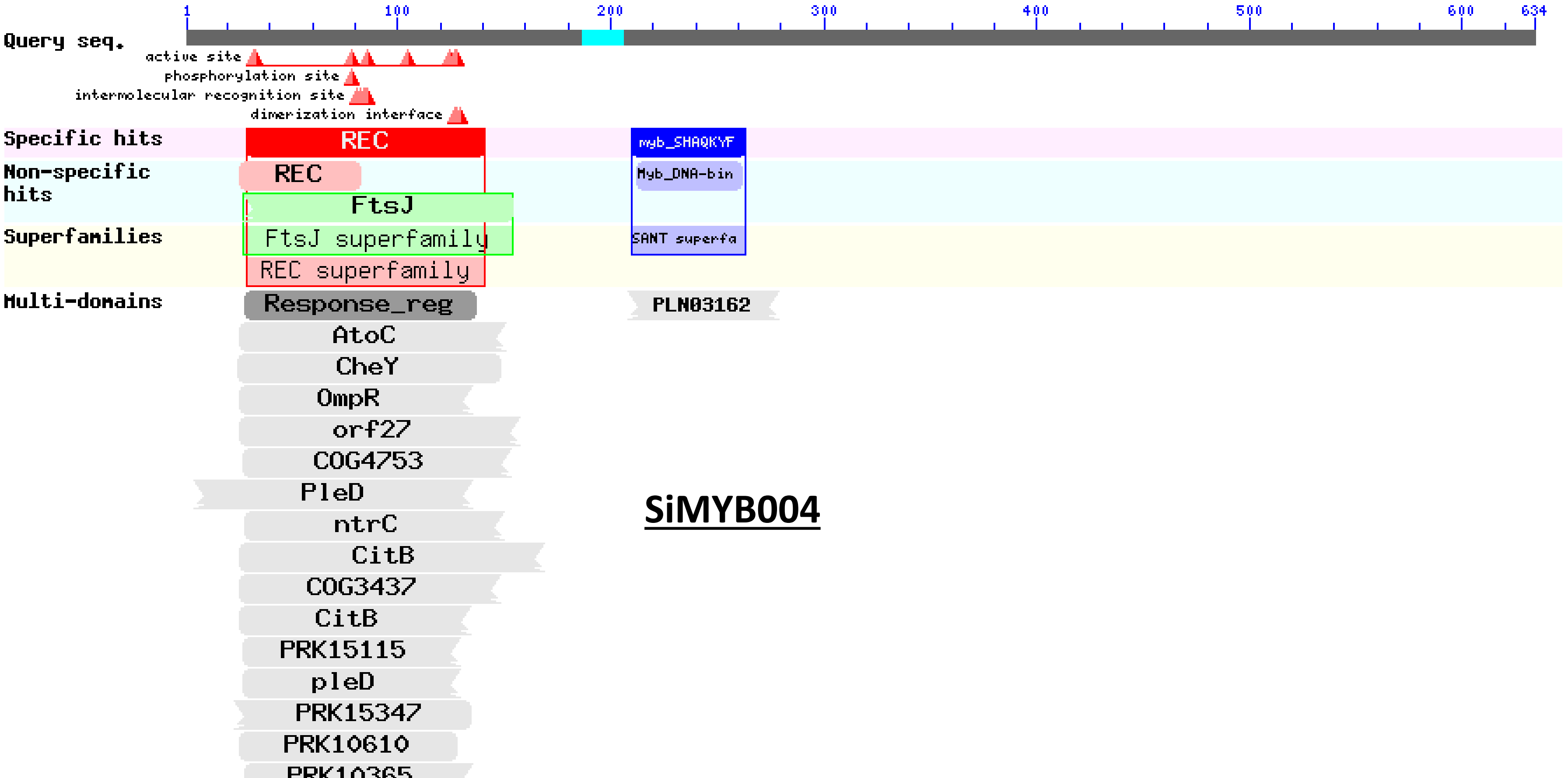

SiMYB004

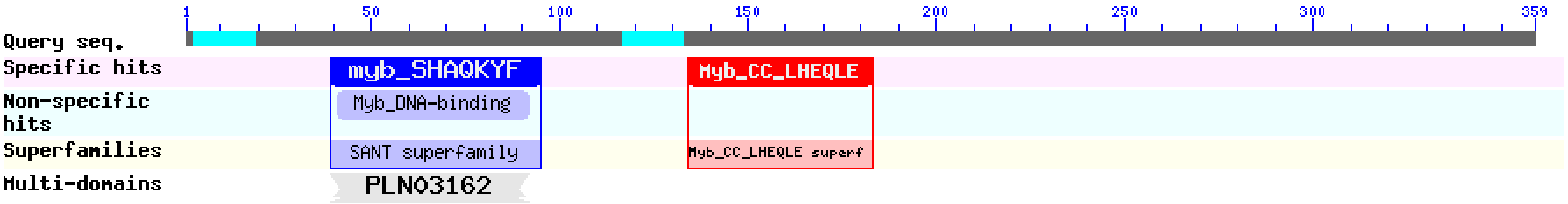

SiMYB005

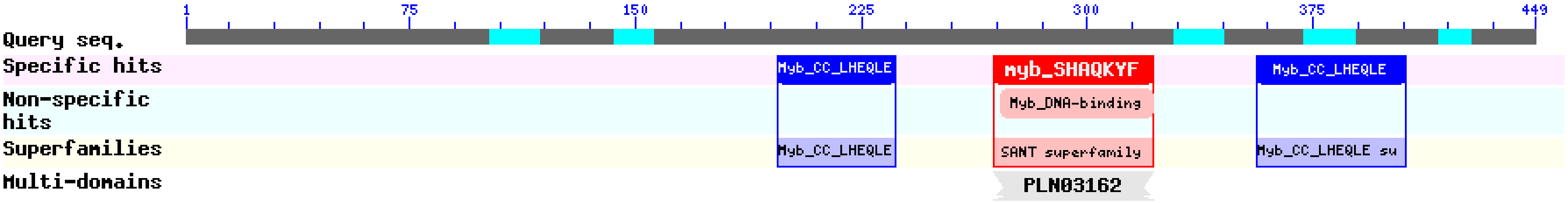

SiMYB006

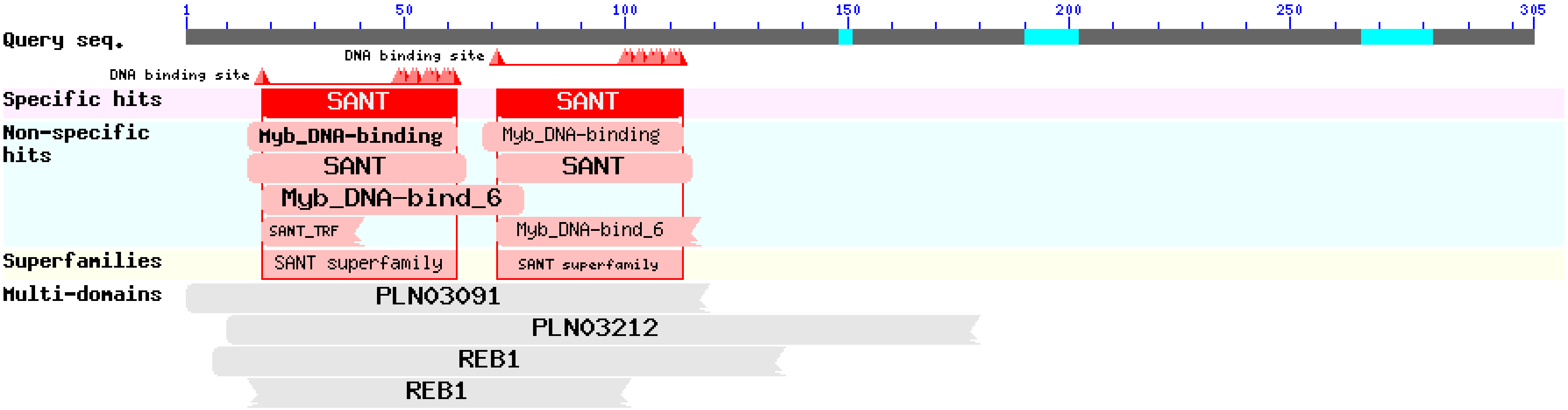

SiMYB007

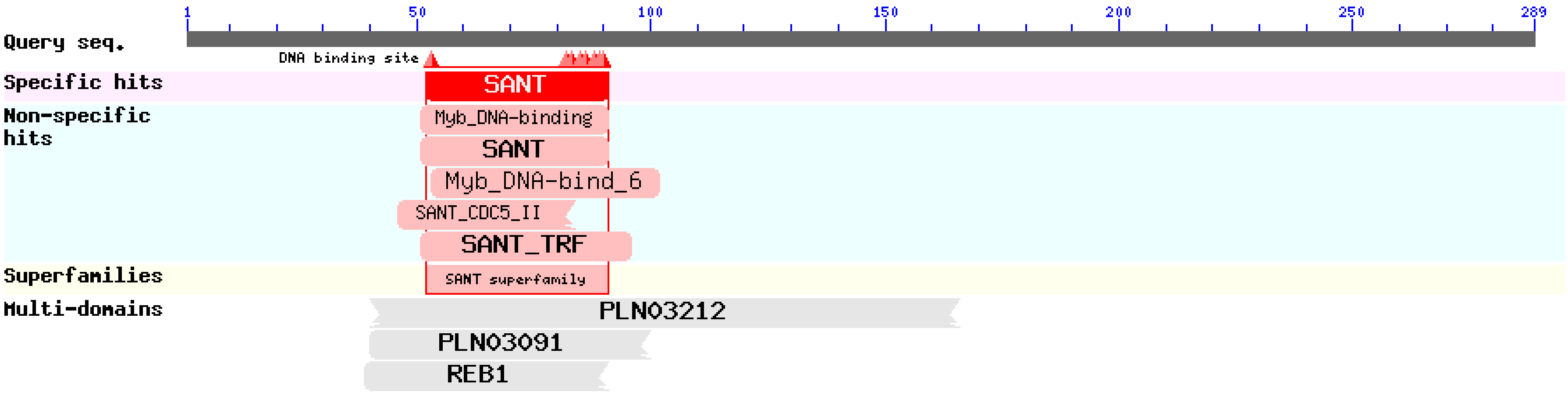

SiMYB008

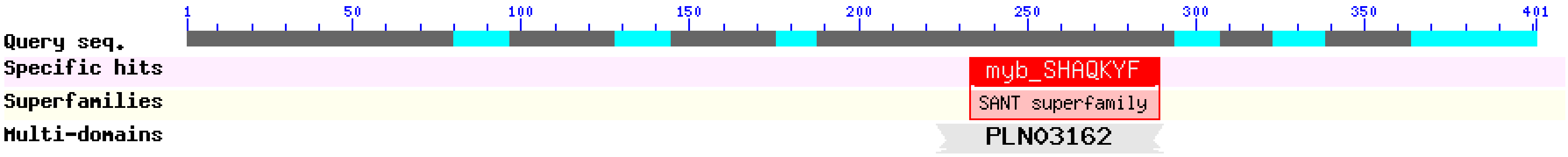

SiMYB009

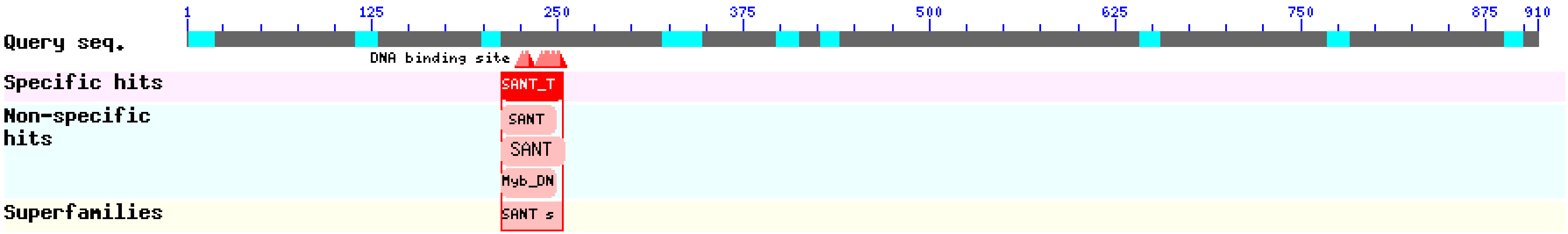

SiMYB010

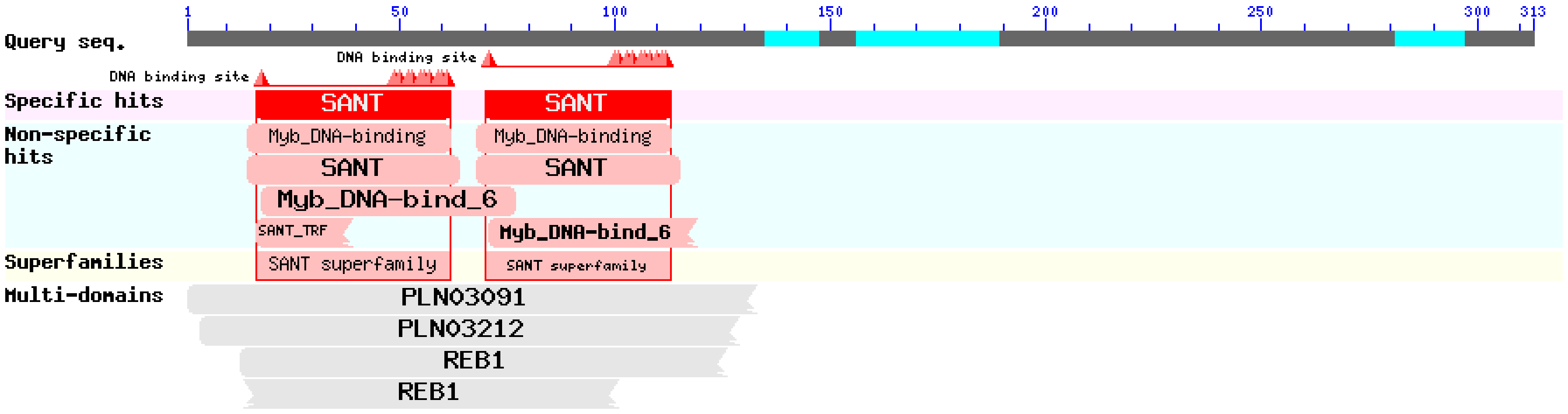

SiMYB011

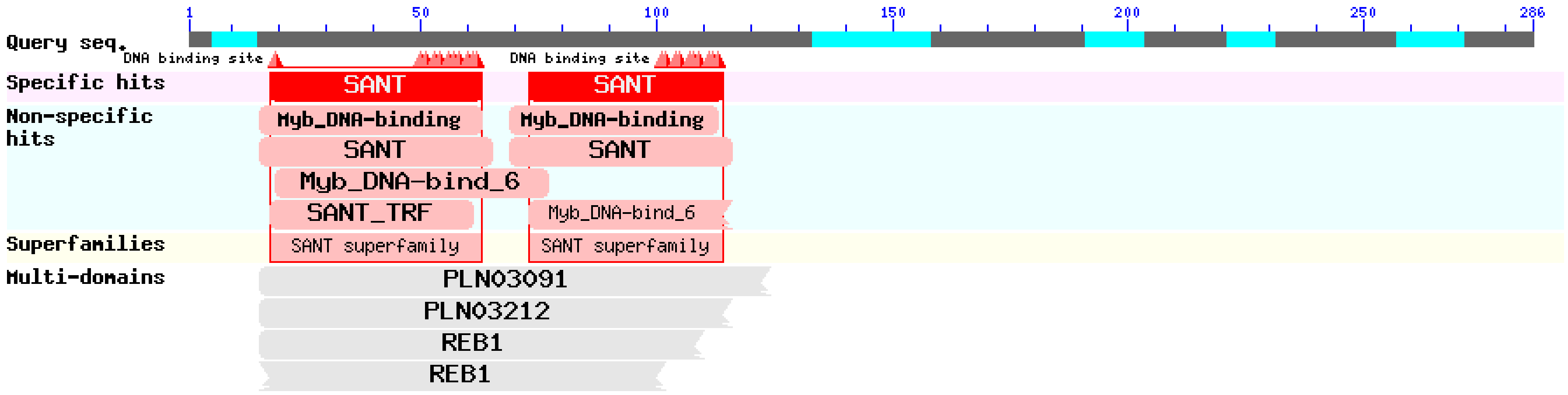

SiMYB012

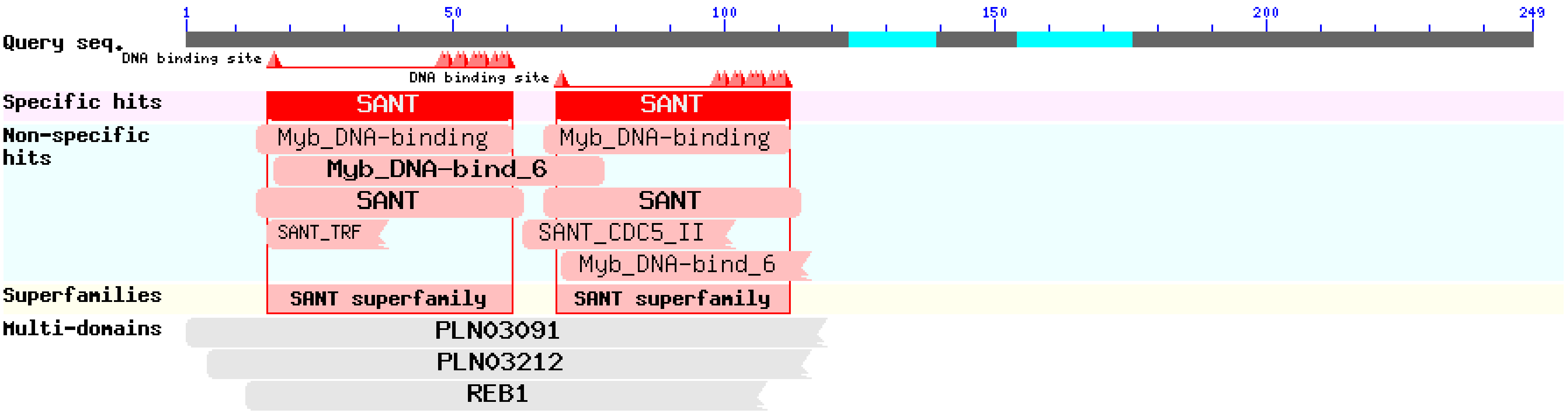

SiMYB013

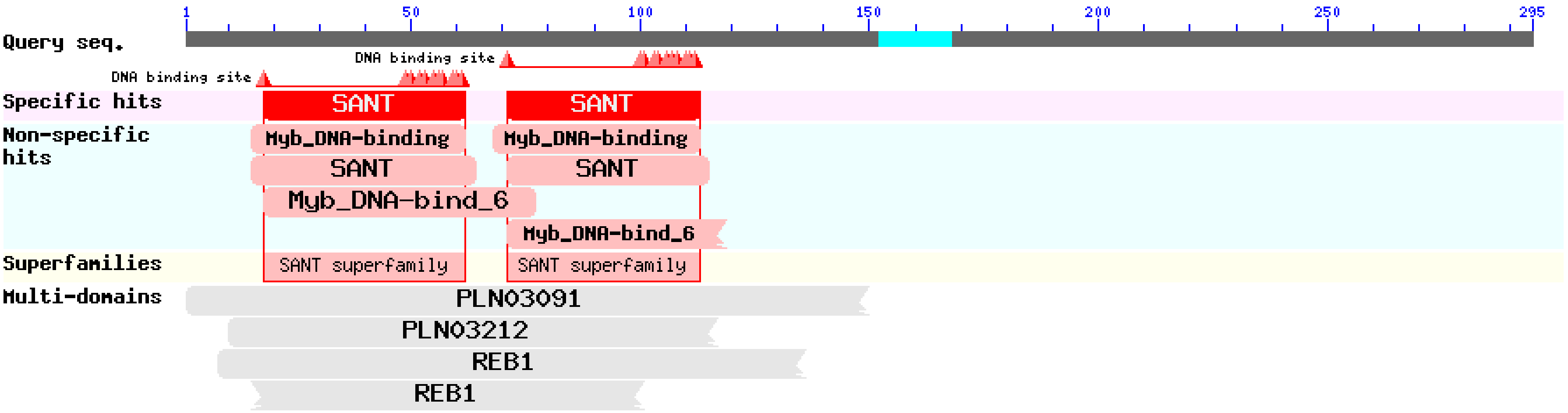

SiMYB014

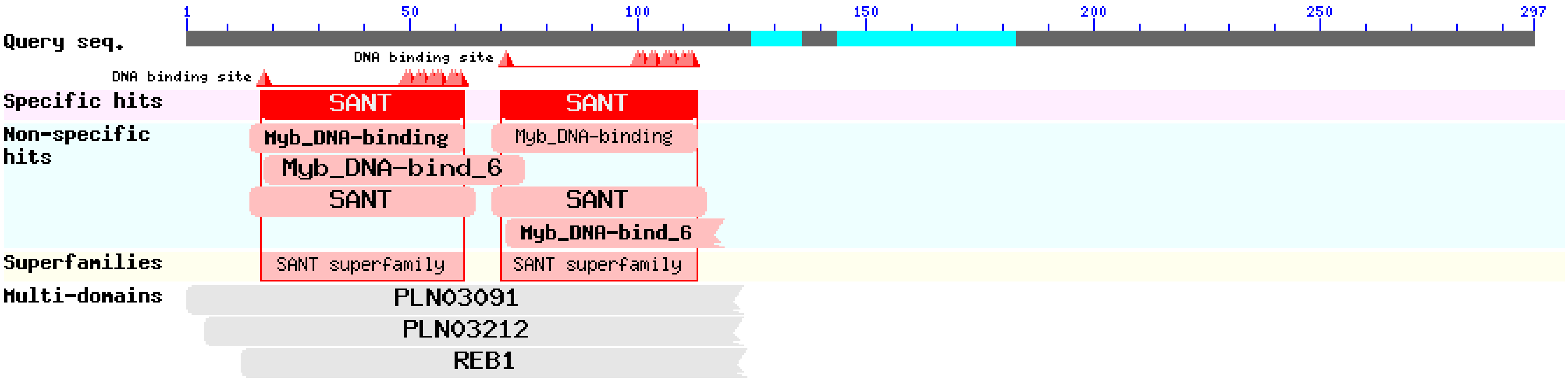

SiMYB015

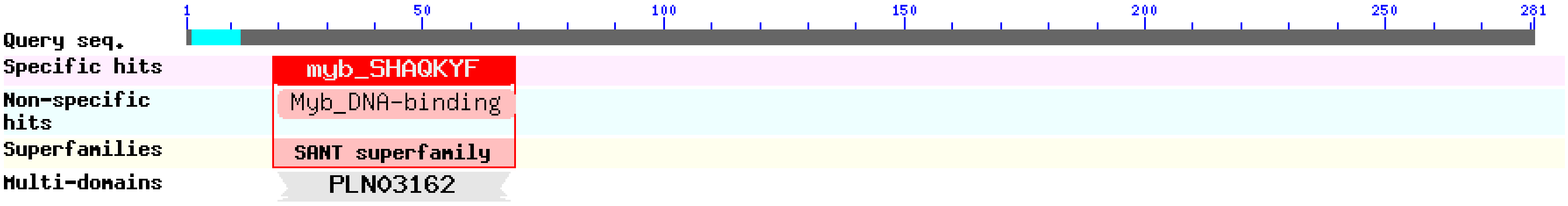

SiMYB016

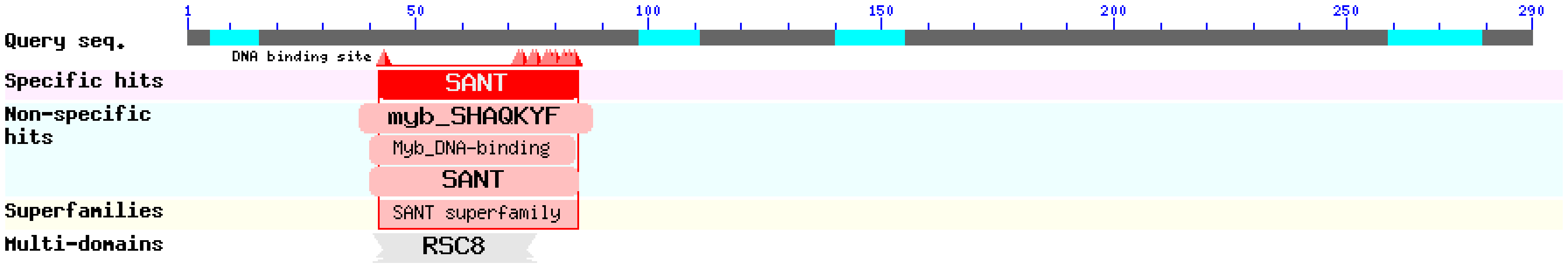

SiMYB017

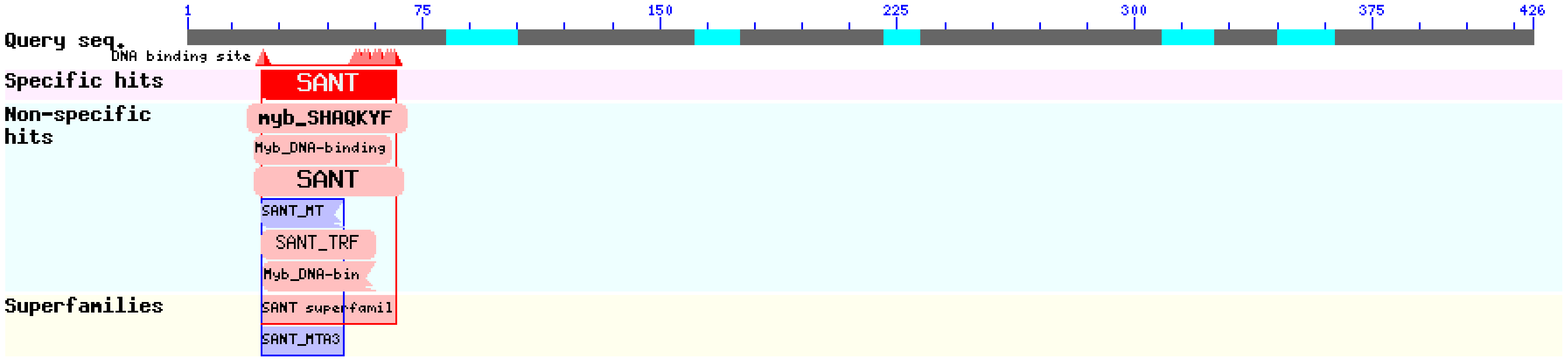

SiMYB018

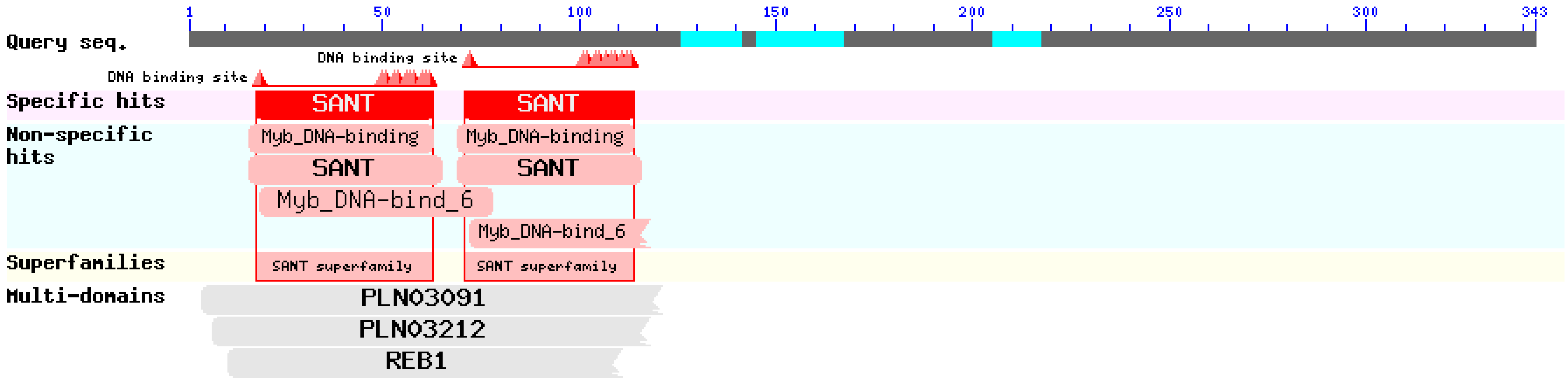

SiMYB019

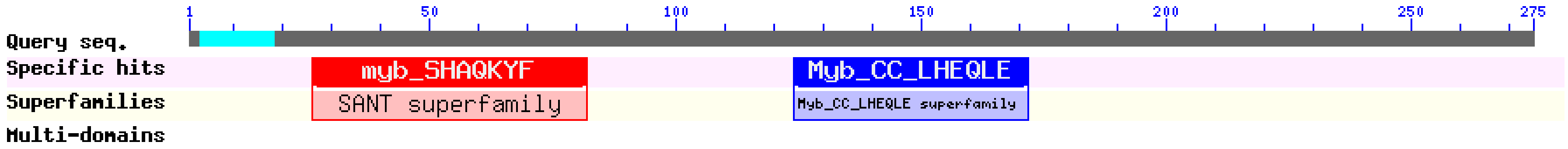

SiMYB020

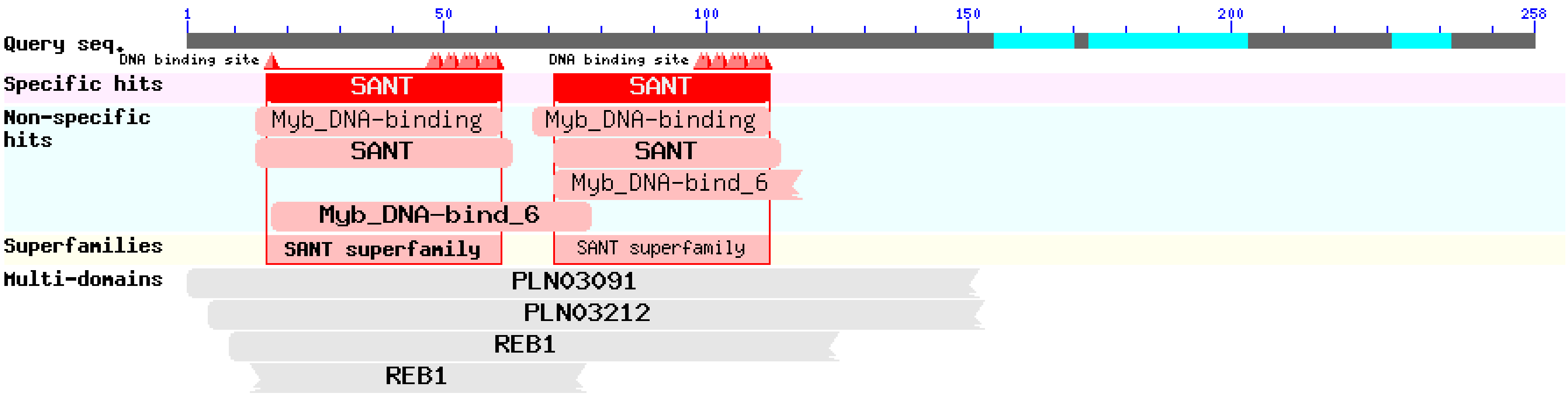

SiMYB021

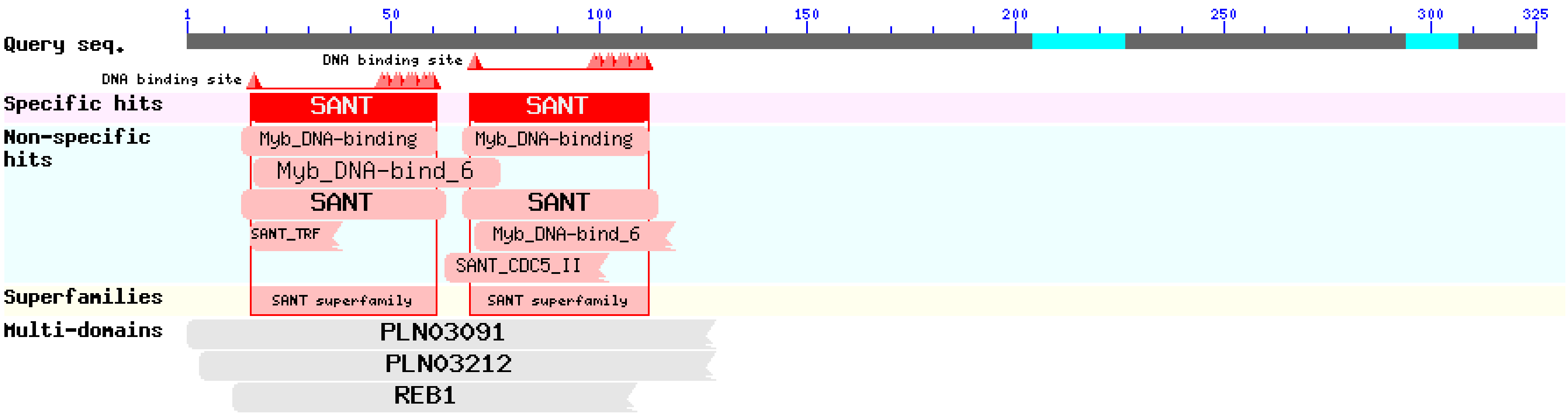

SiMYB022

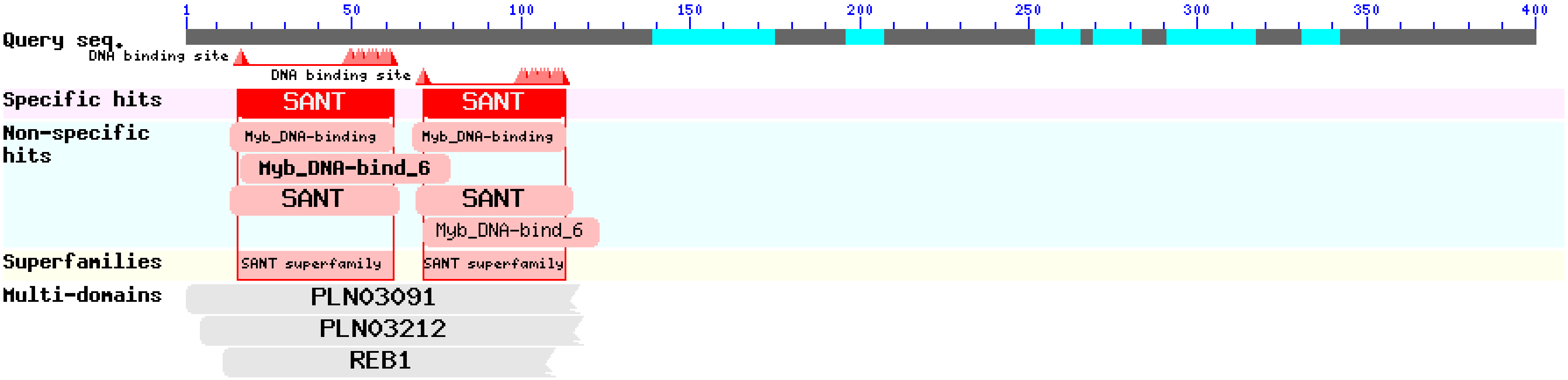

SiMYB023

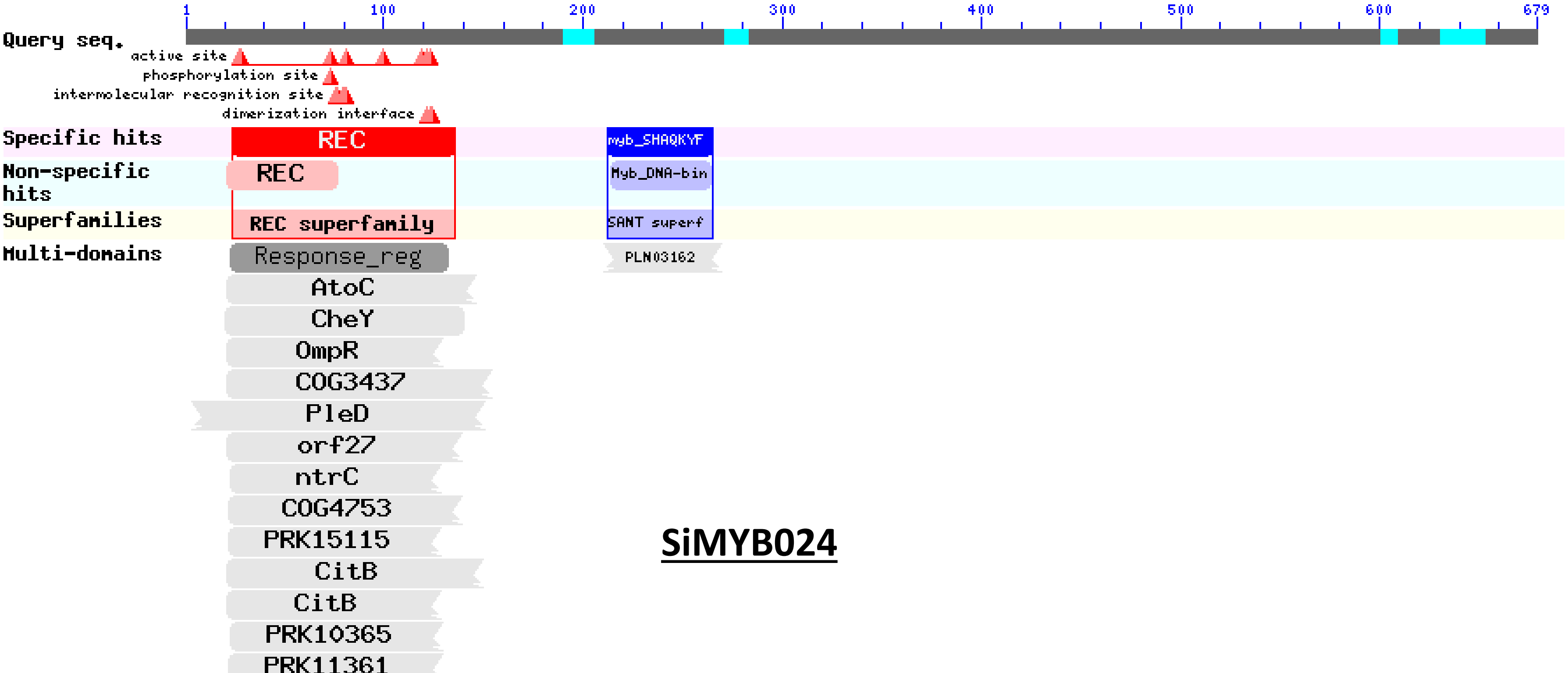

SiMYB024

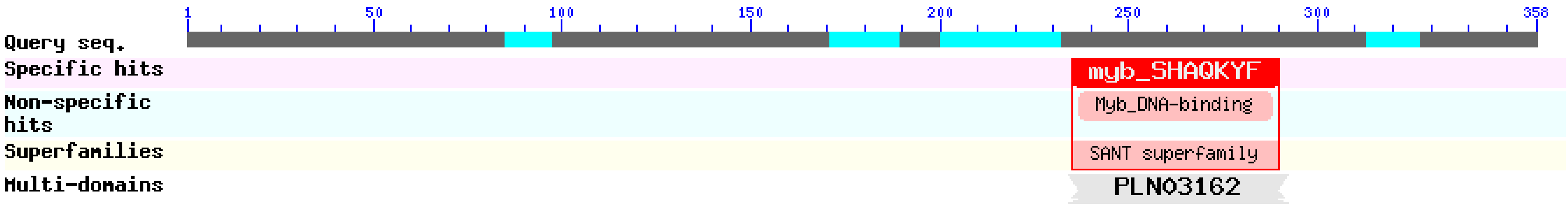

SiMYB025

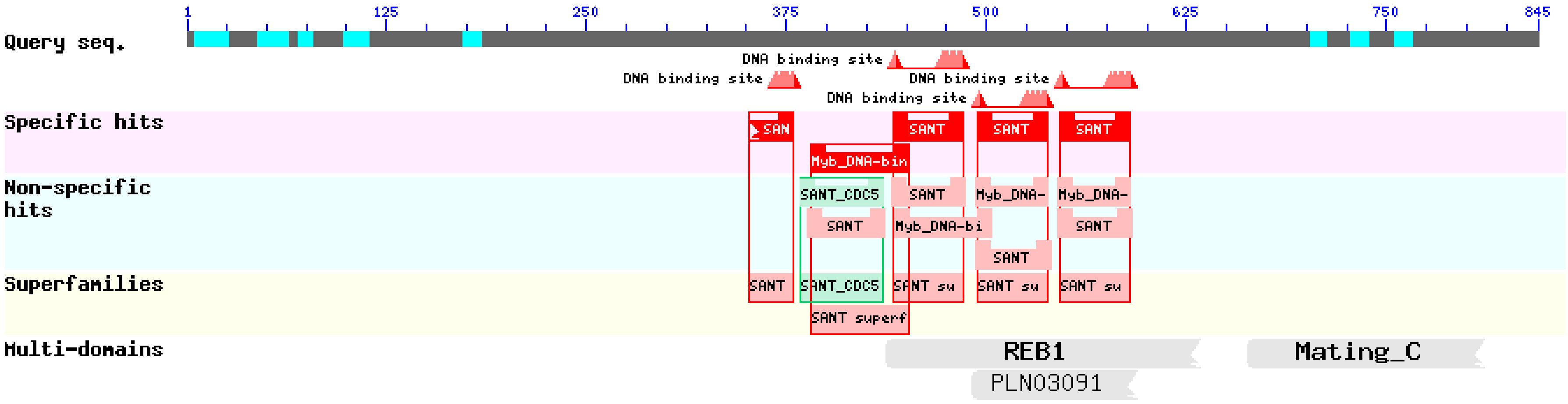

SiMYB026

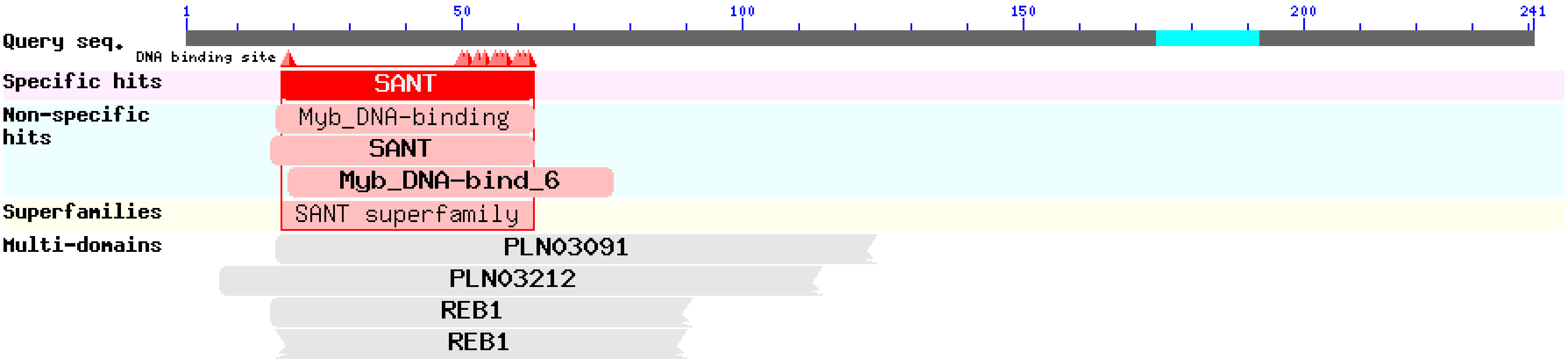

SiMYB027

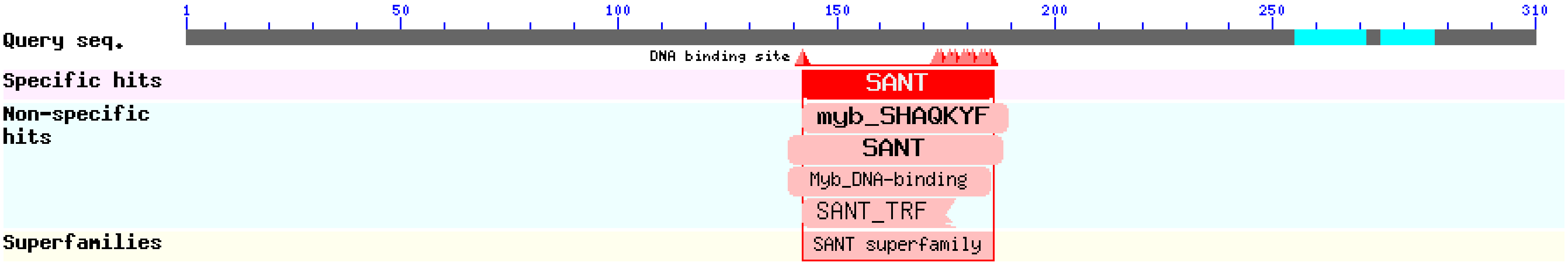

SiMYB028

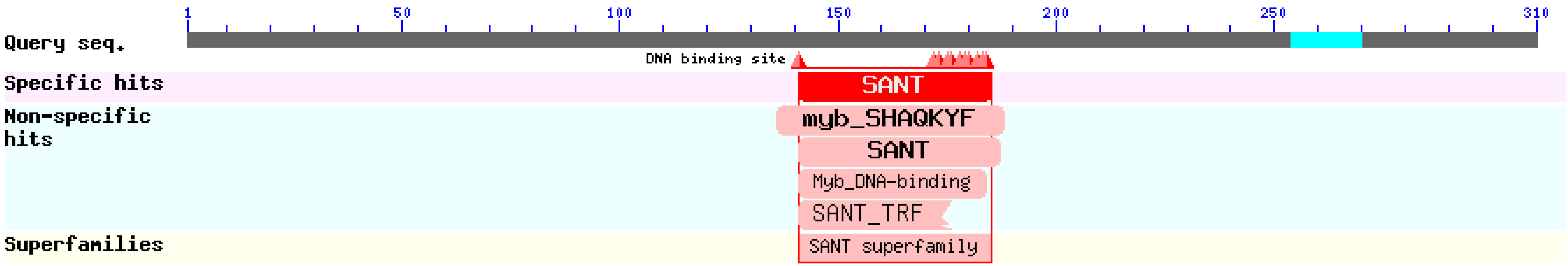

SiMYB029

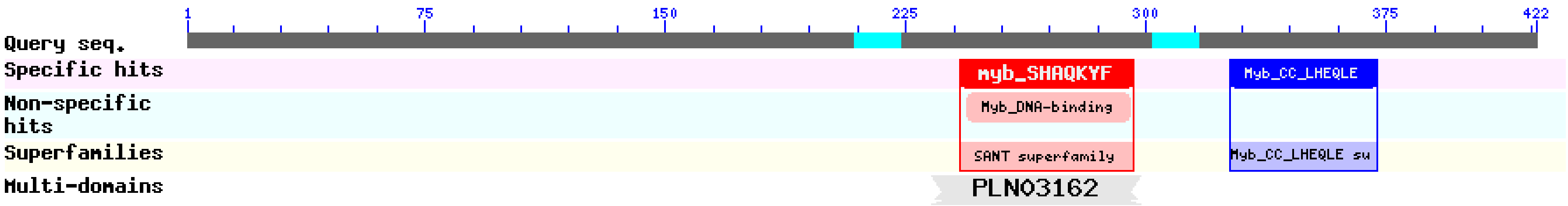

SiMYB030

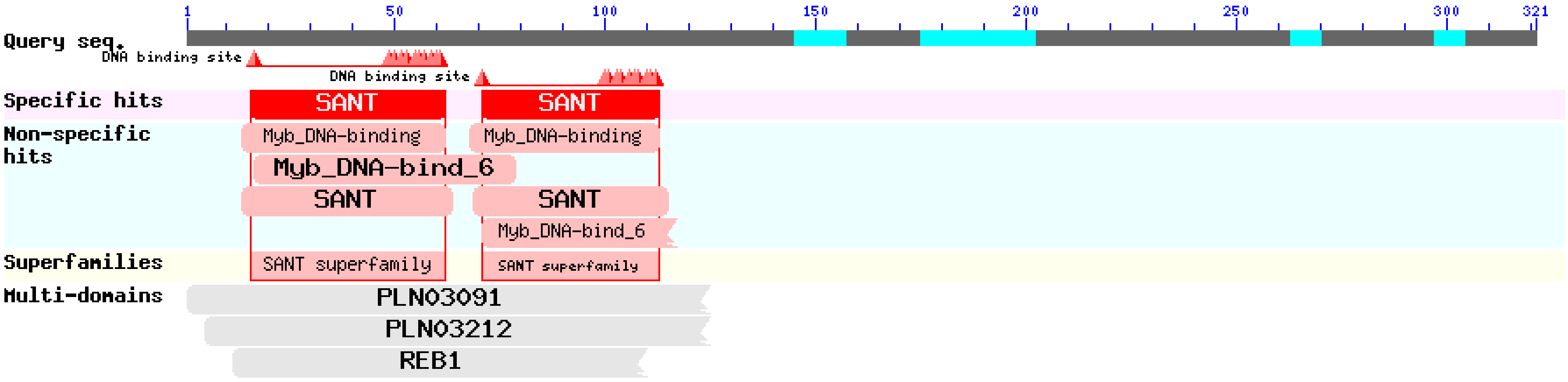

SiMYB031

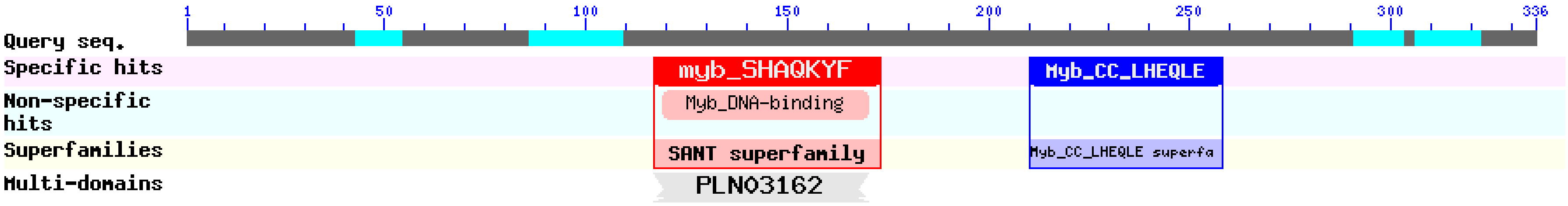

SiMYB032

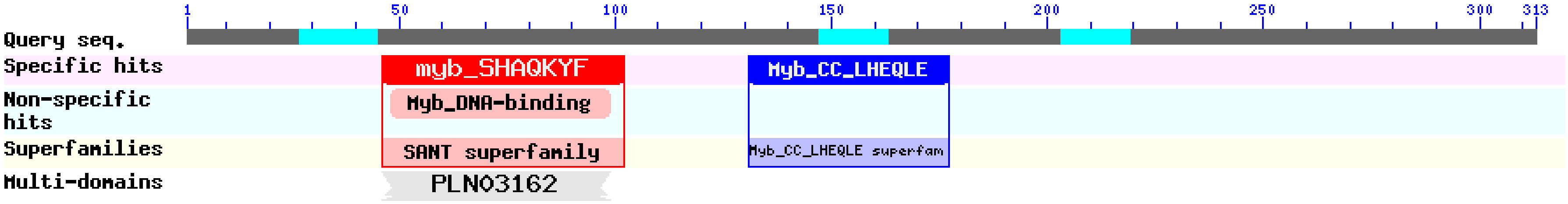

SiMYB033

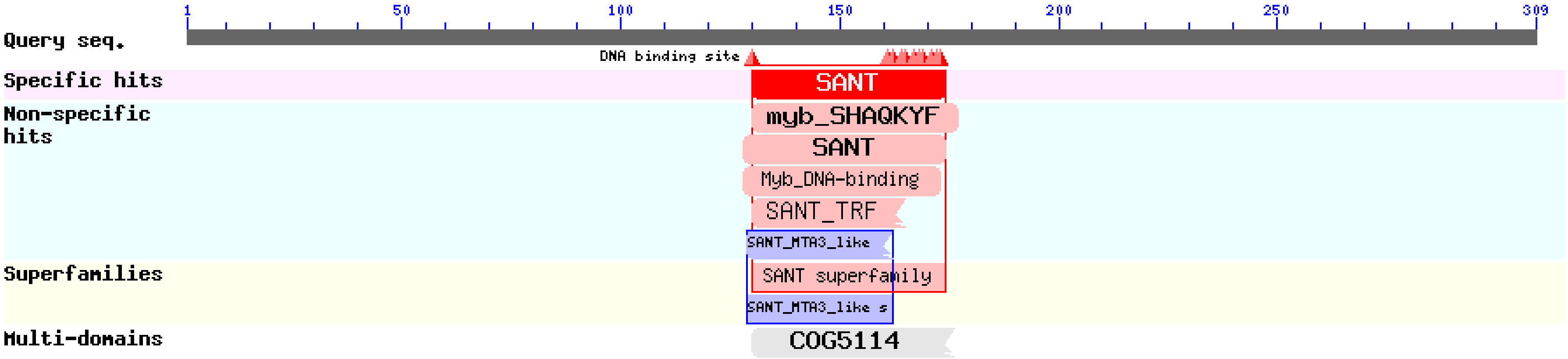

SiMYB034

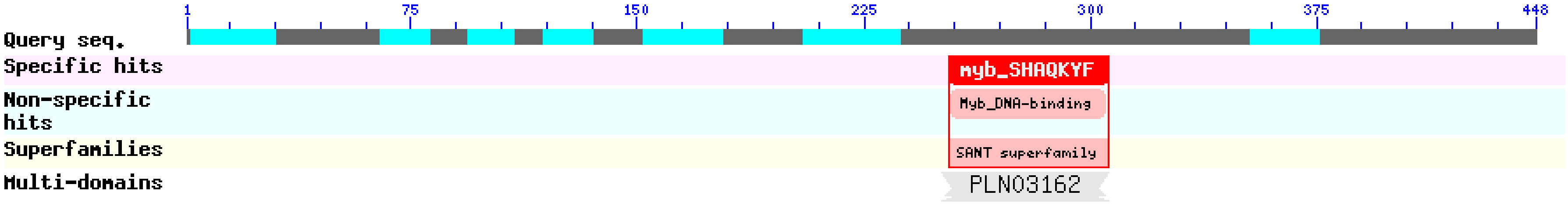

SiMYB035

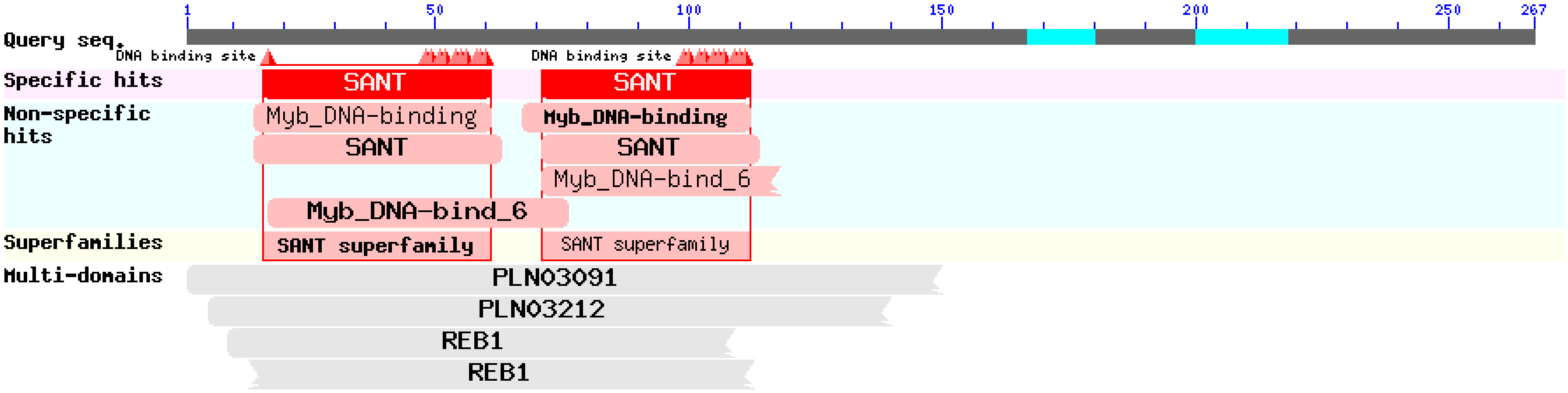

SiMYB036

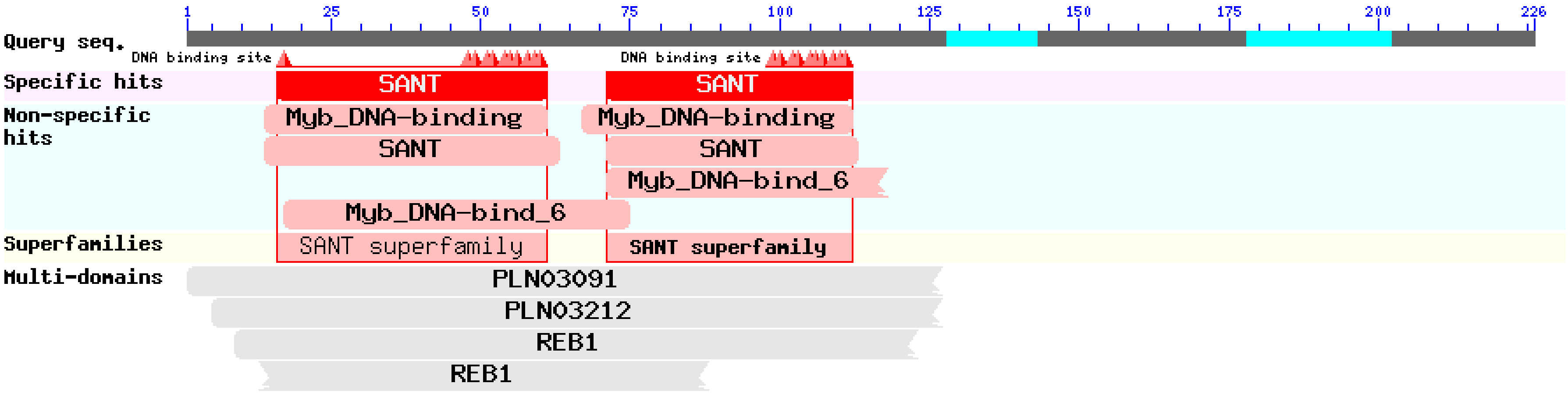

SiMYB037

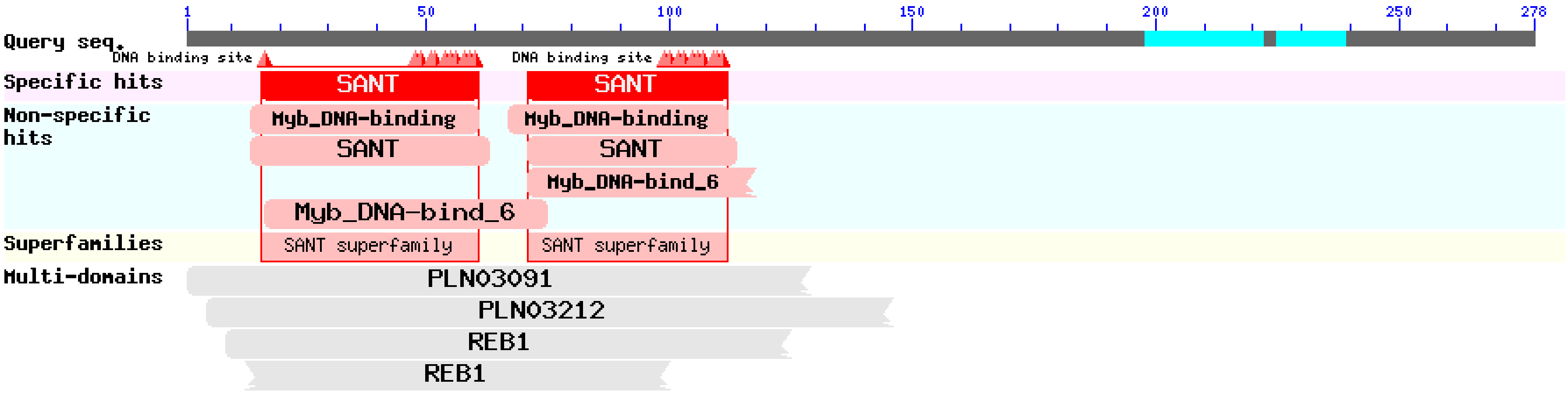

SiMYB038

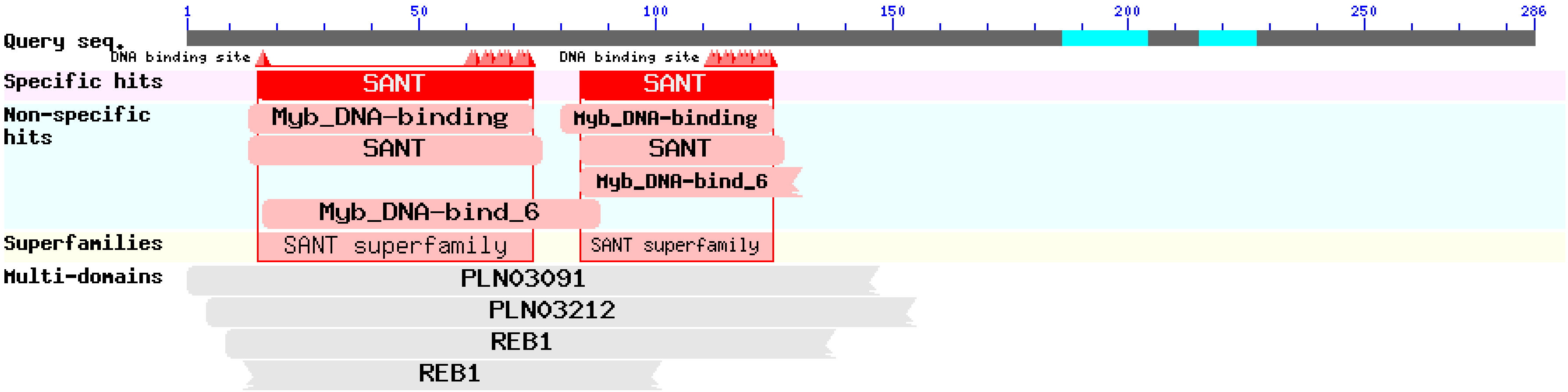

SiMYB039

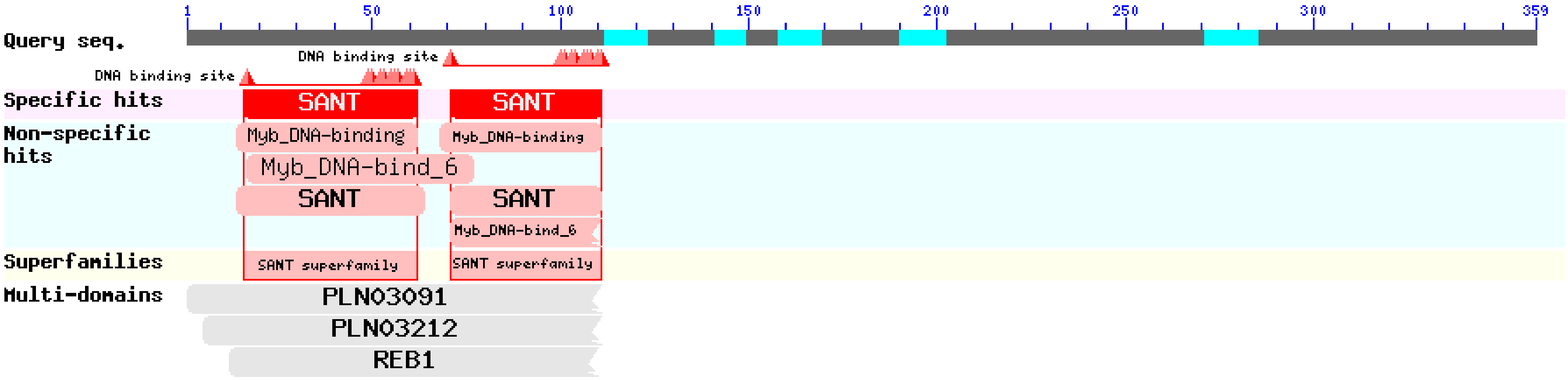

SiMYB040

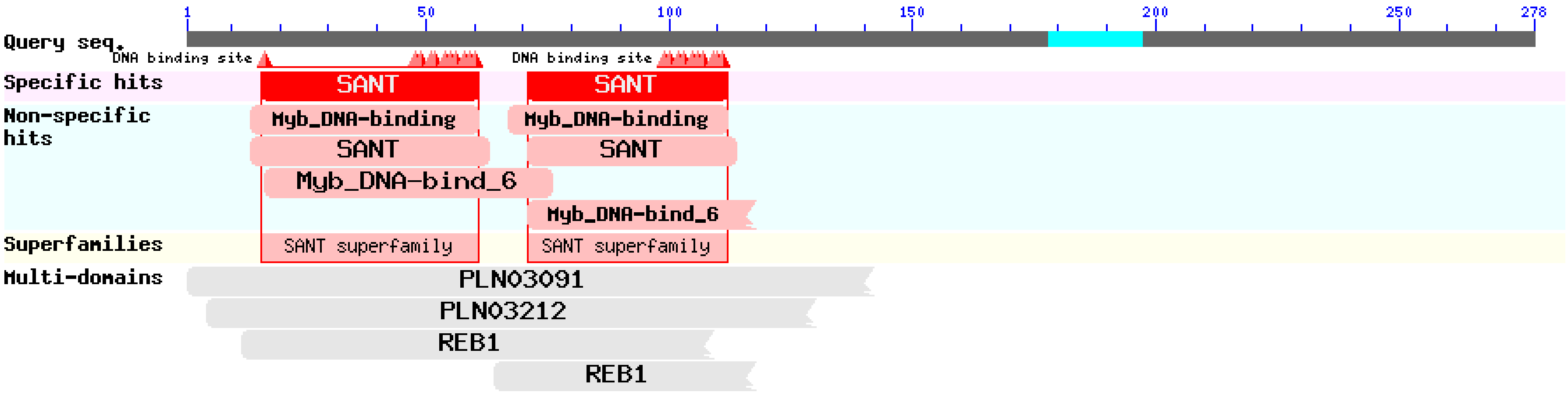

SiMYB041

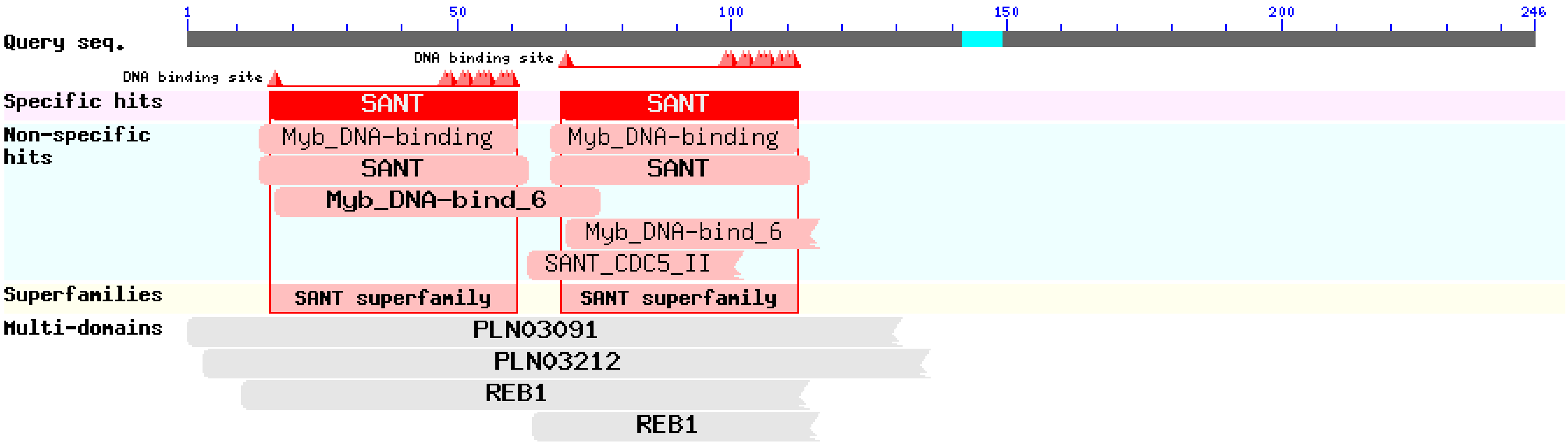

SiMYB042

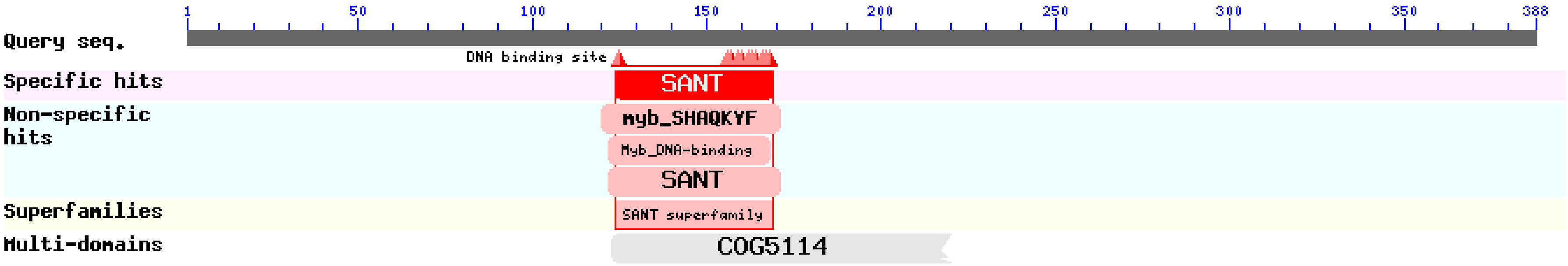

SiMYB043

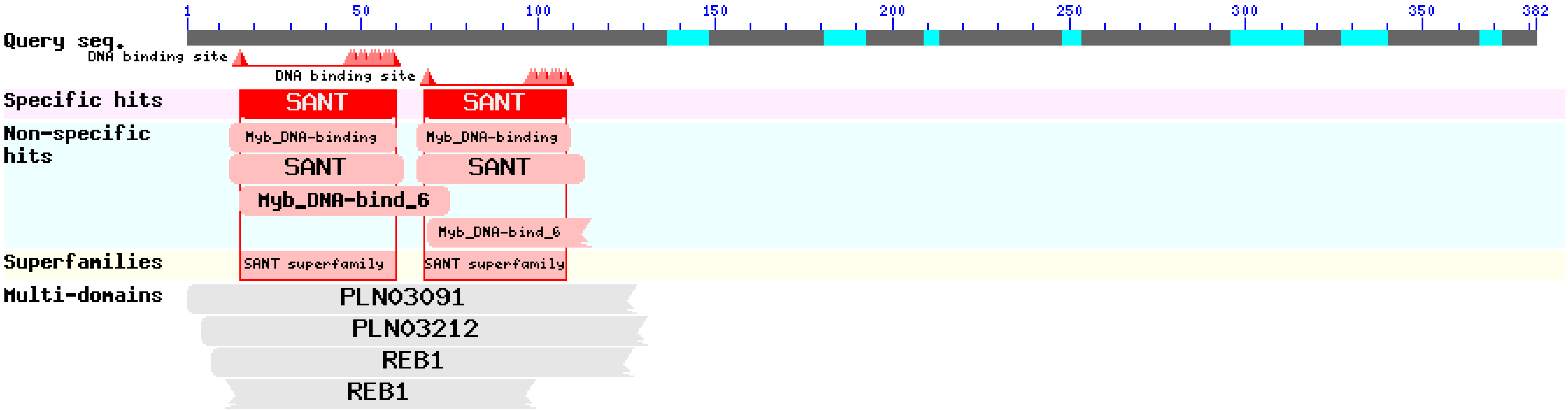

SiMYB044

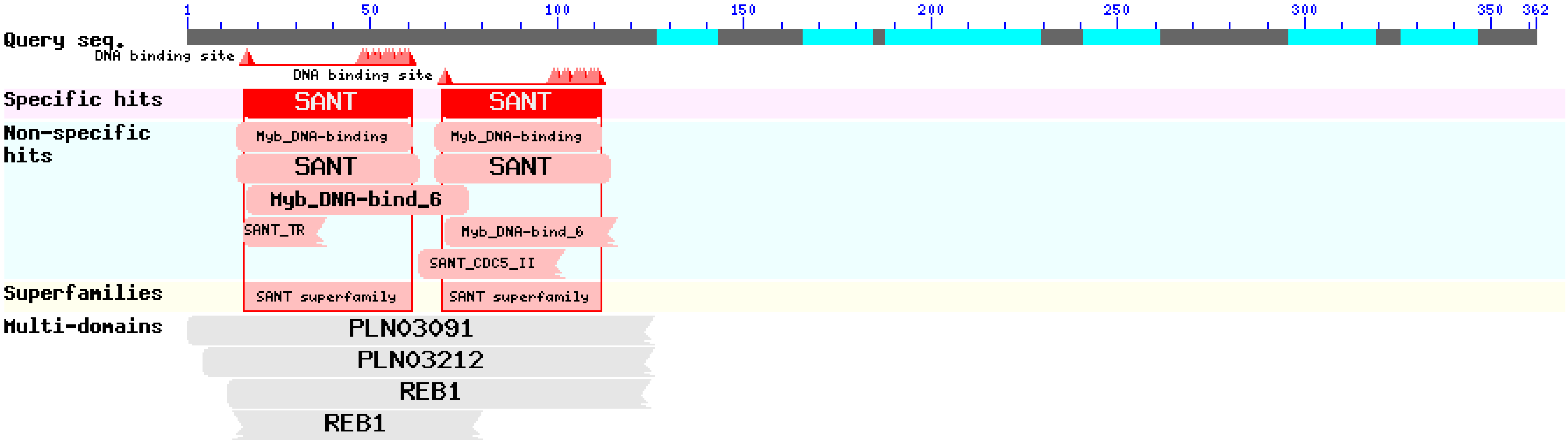

SiMYB045

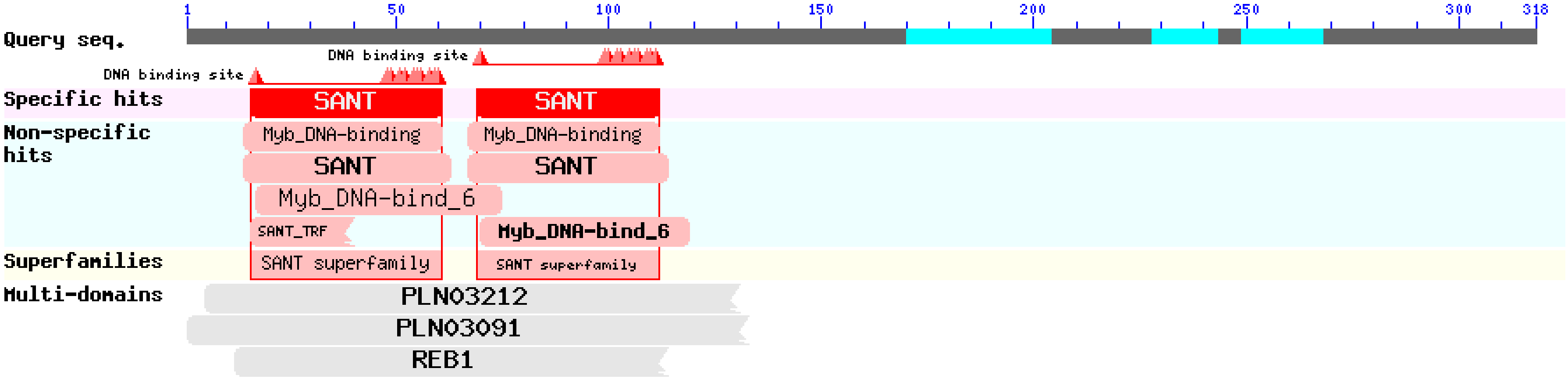

SiMYB046

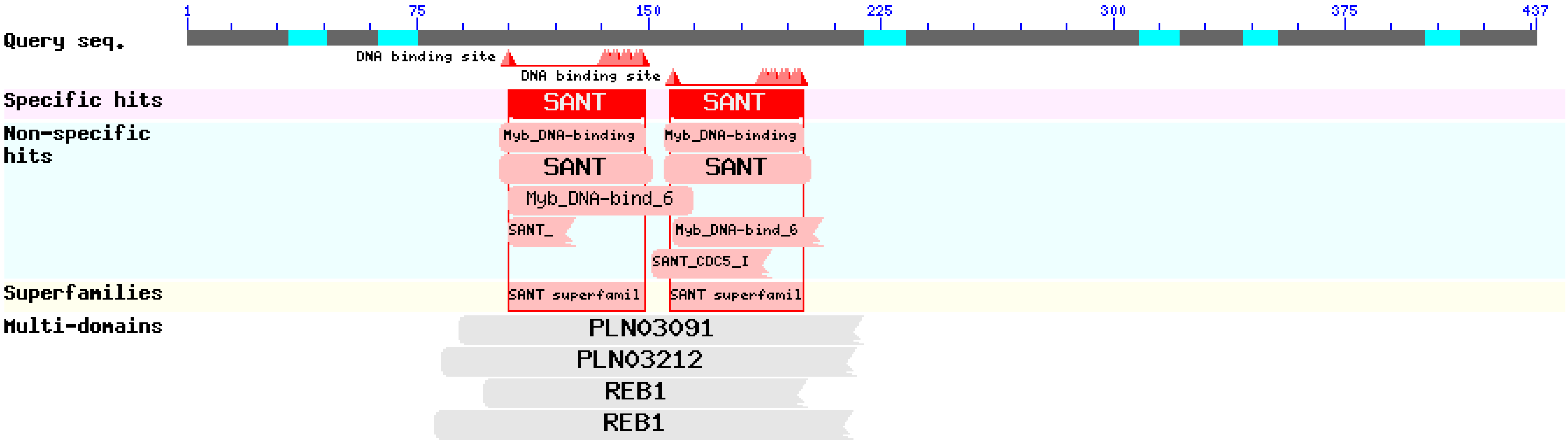

SiMYB047

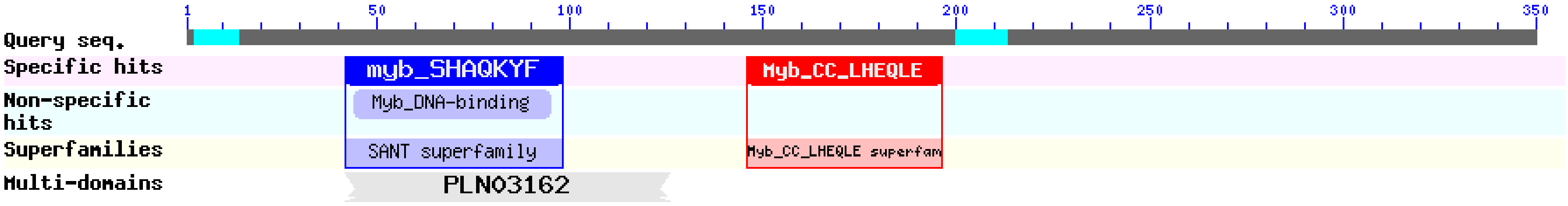

SiMYB048

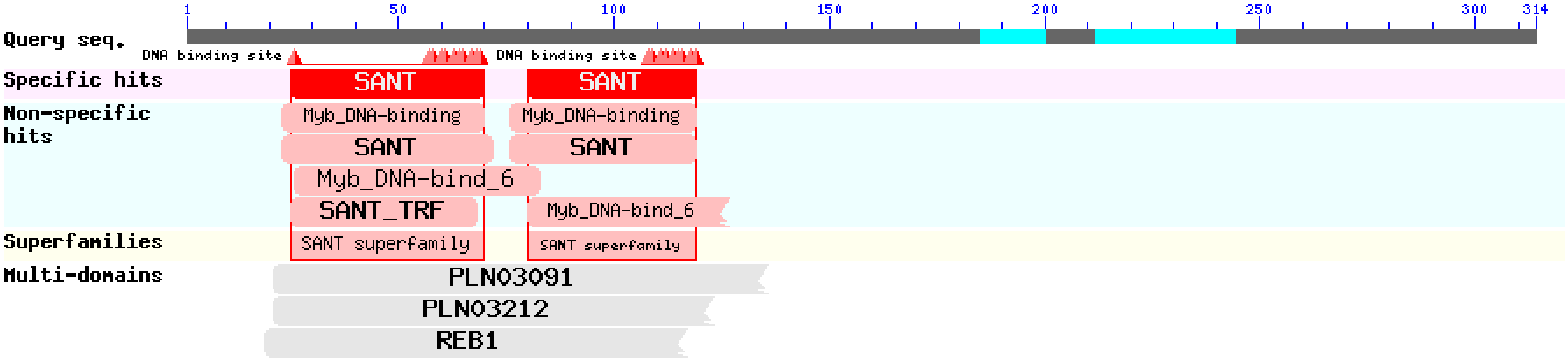

SiMYB049

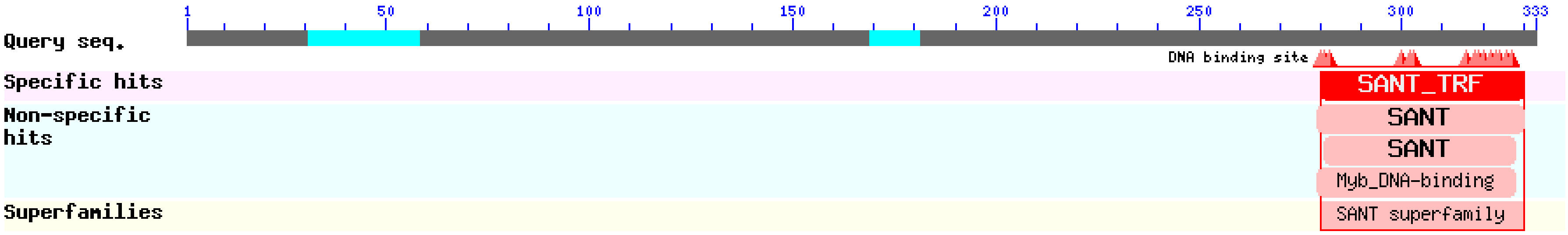

SiMYB050

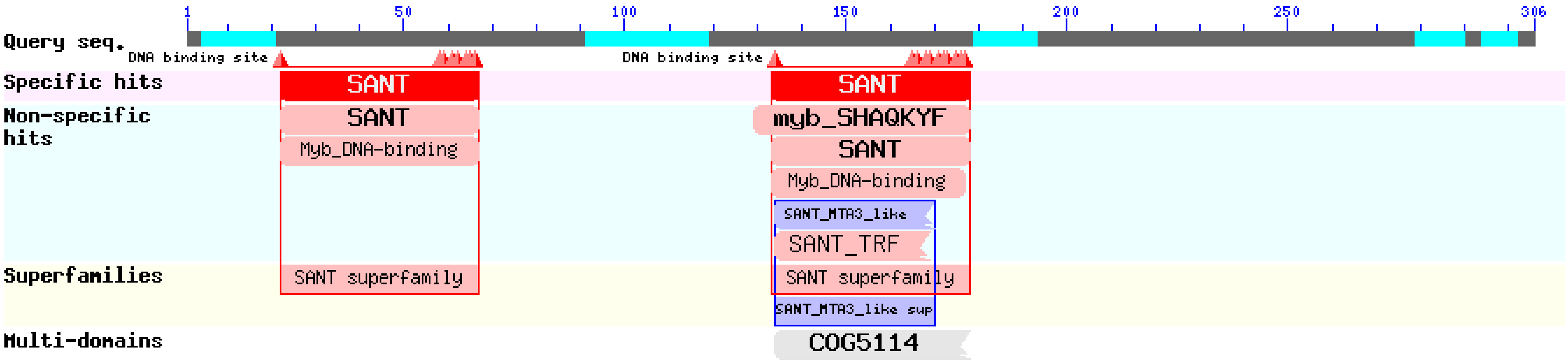

SiMYB051

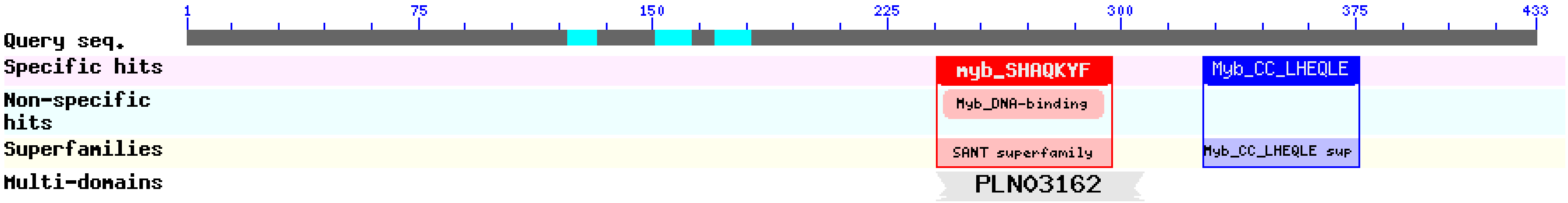

SiMYB052

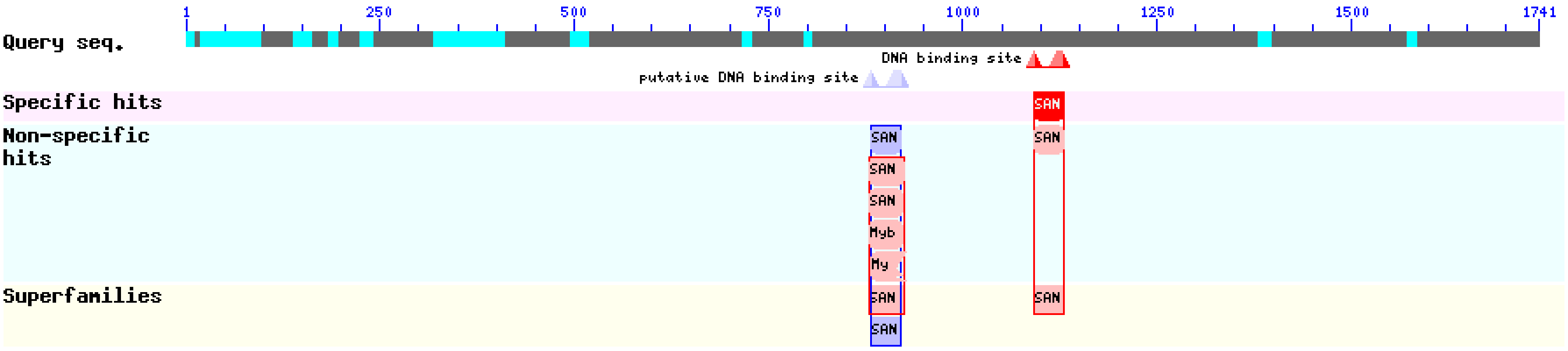

SiMYB053

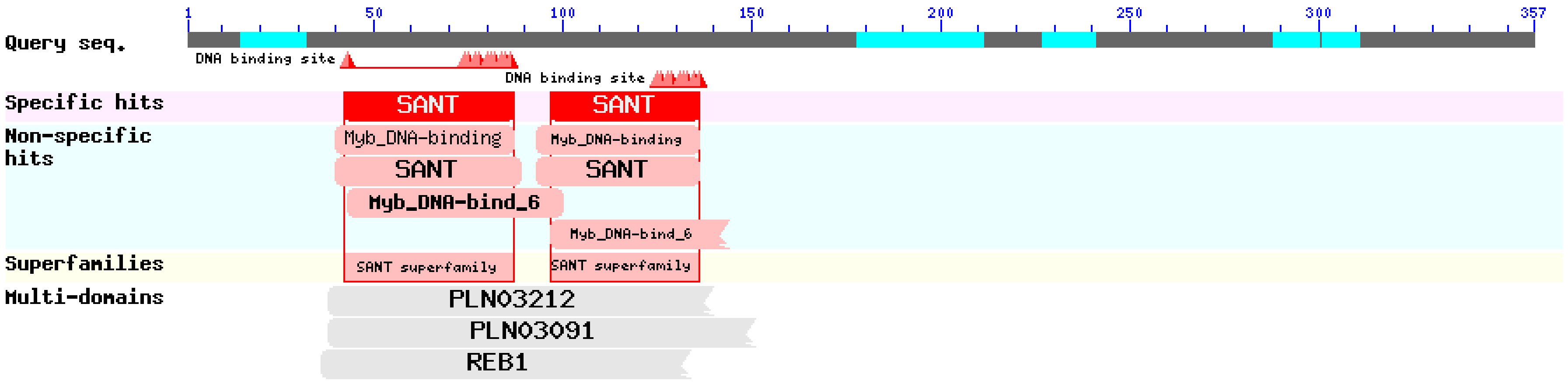

SiMYB054

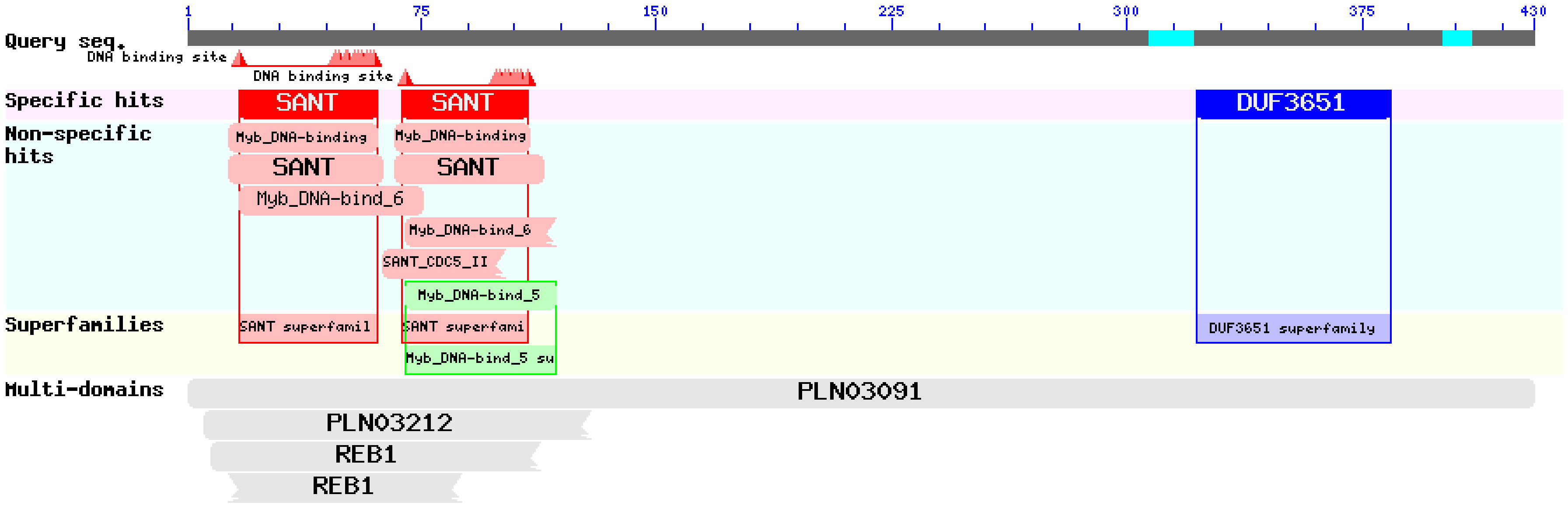

SiMYB055

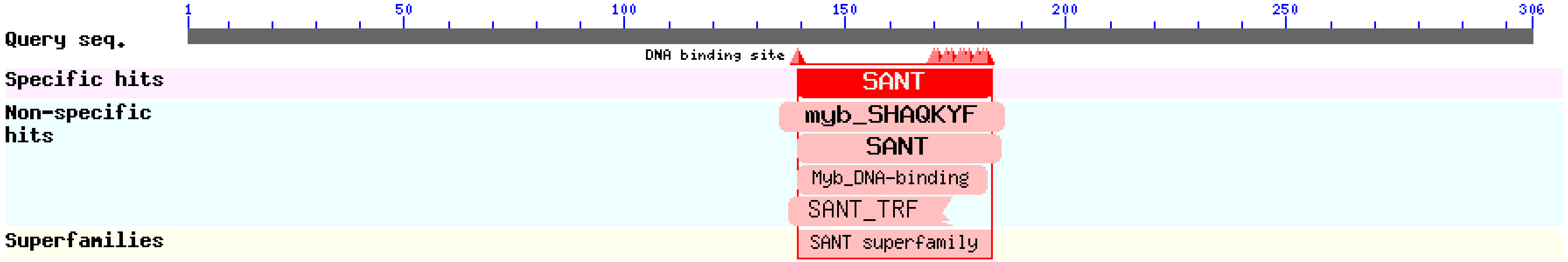

SiMYB056

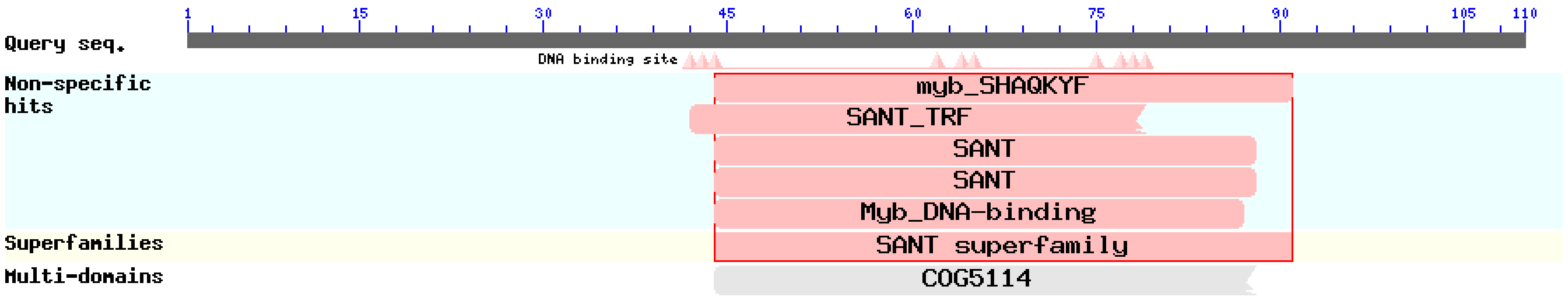

SiMYB057

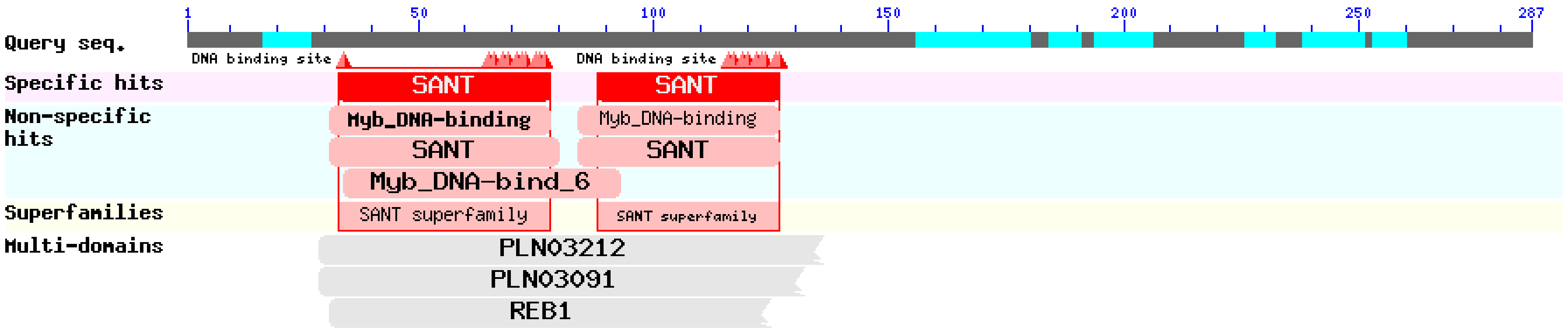

SiMYB058

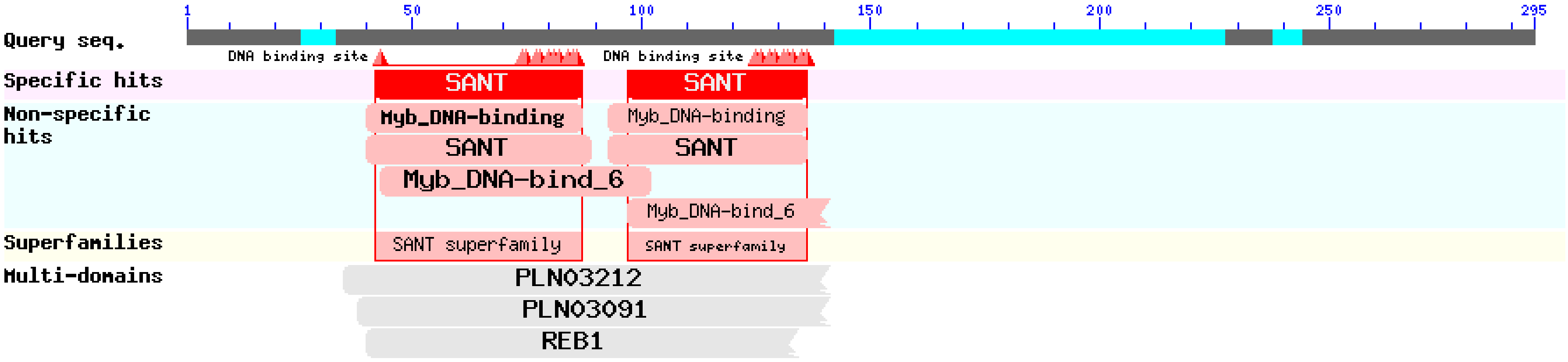

SiMYB059

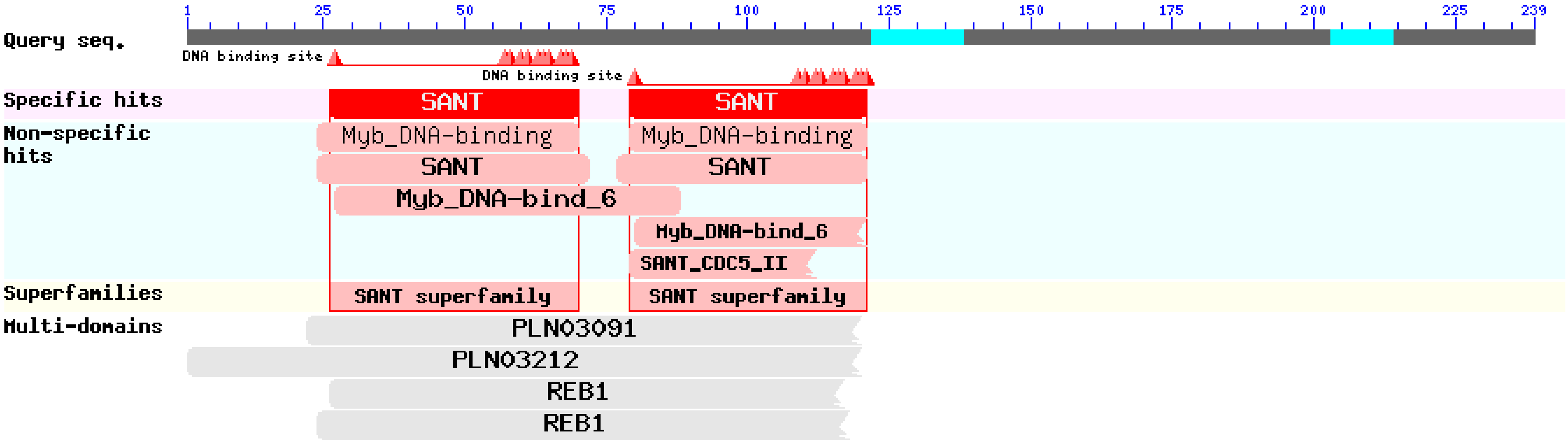

SiMYB060

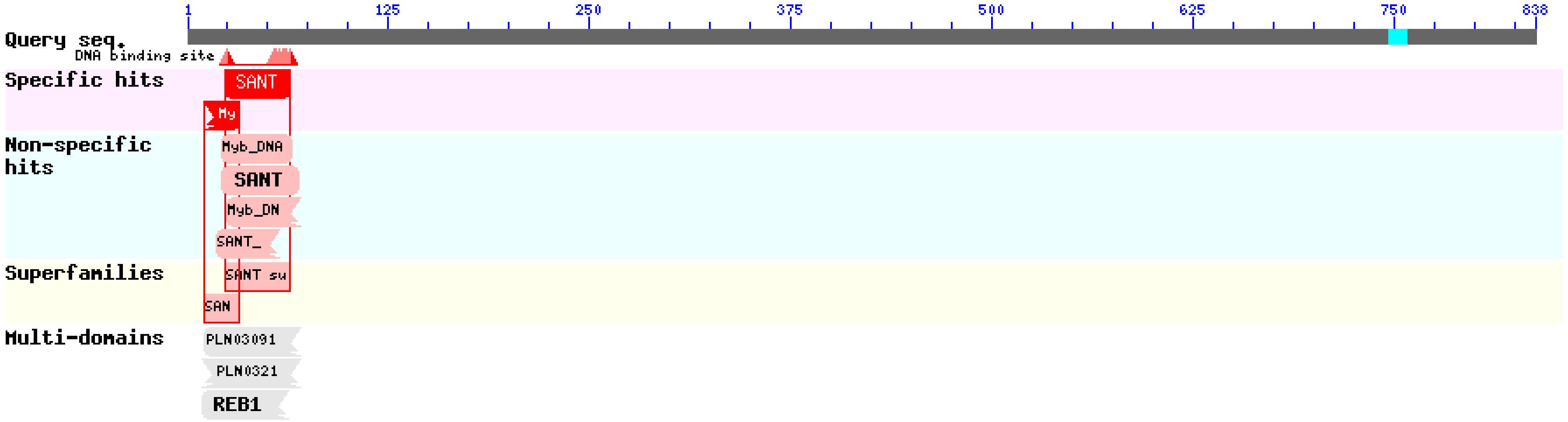

SiMYB061

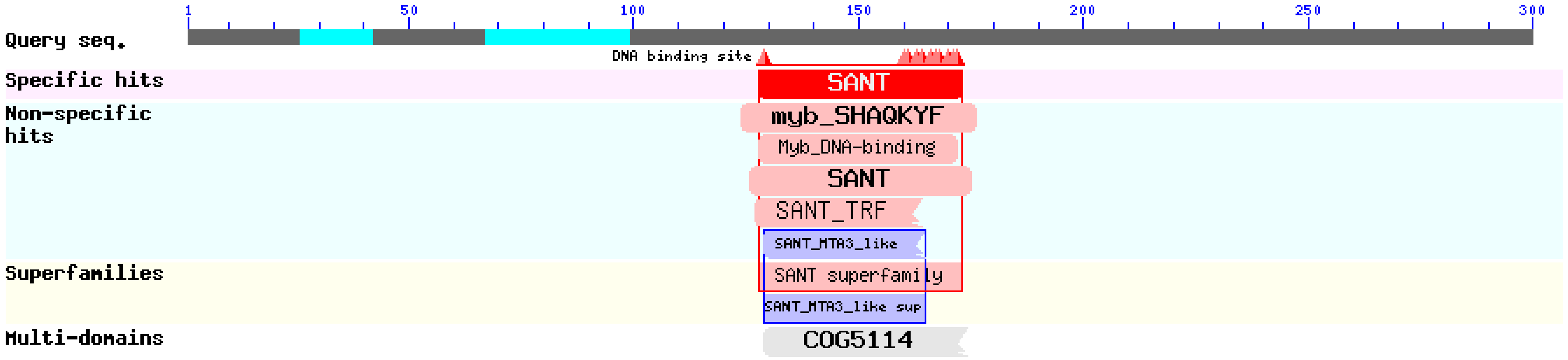

SiMYB062

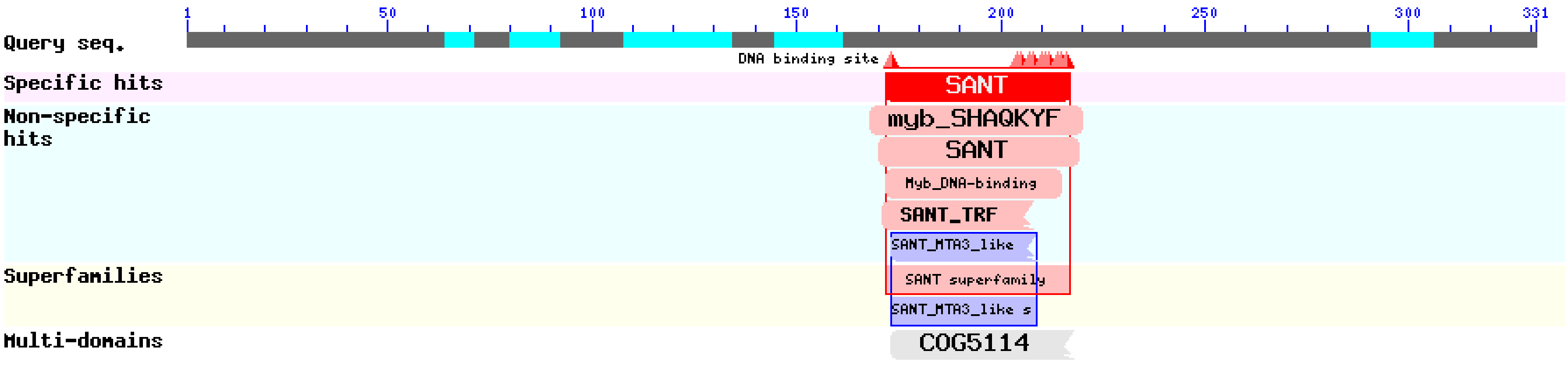

SiMYB063

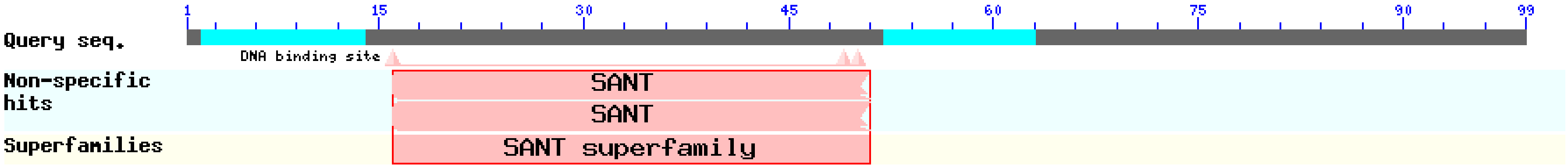

SiMYB064

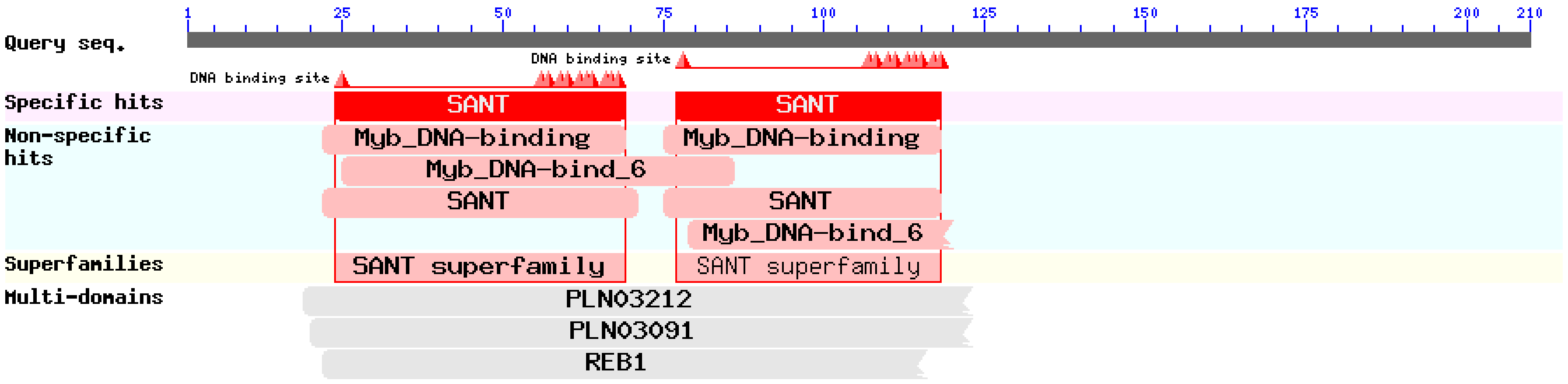

SiMYB065

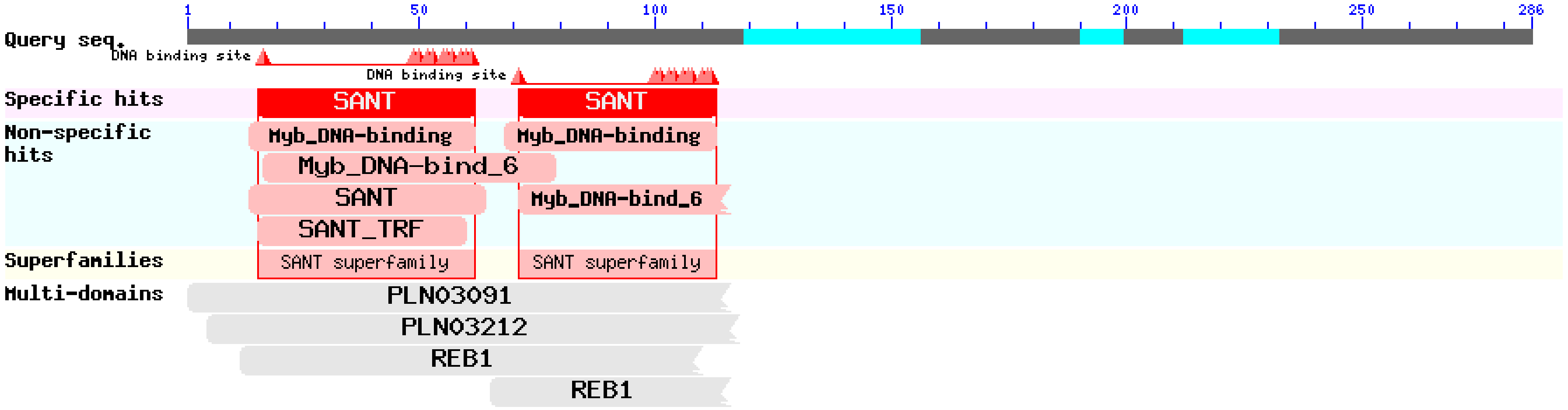

SiMYB066

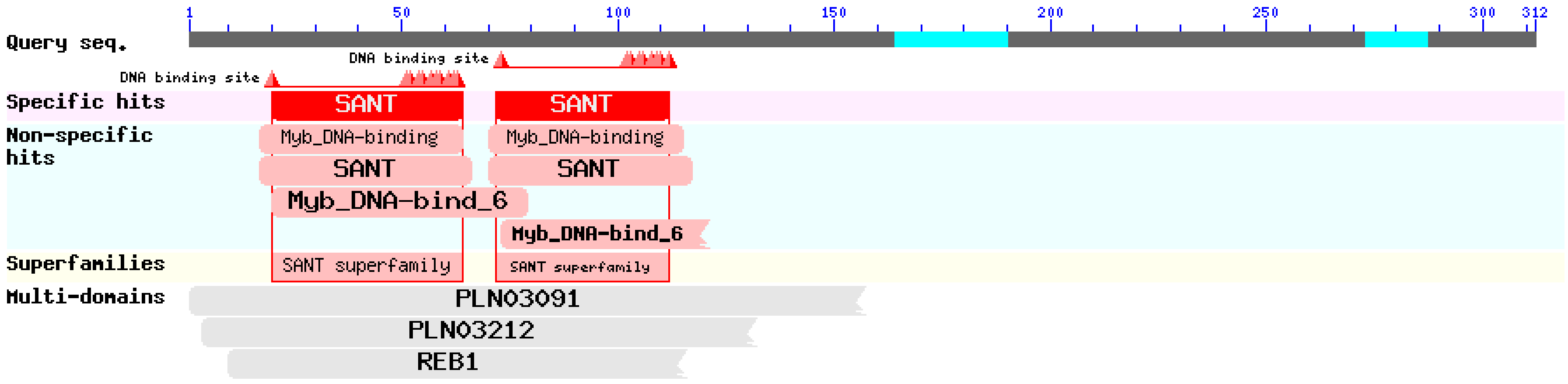

SiMYB067

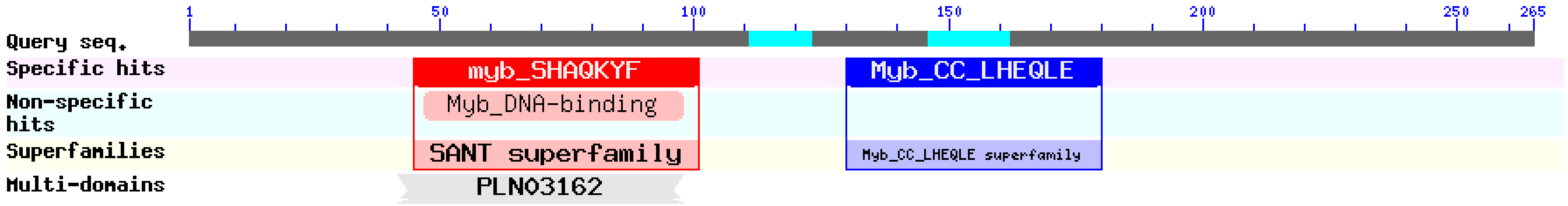

SiMYB068

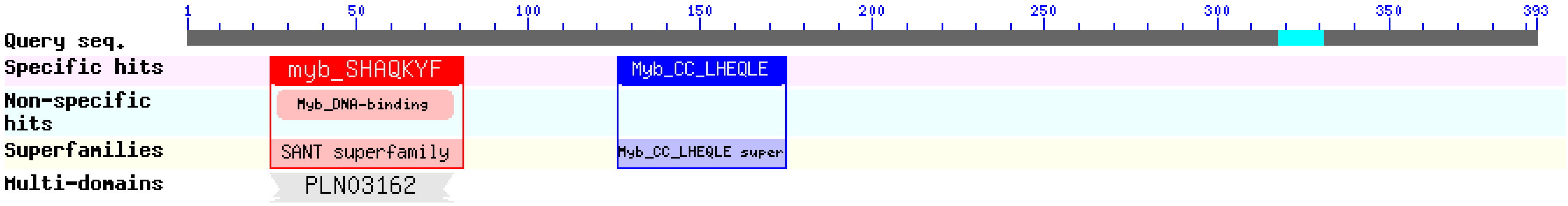

SiMYB069

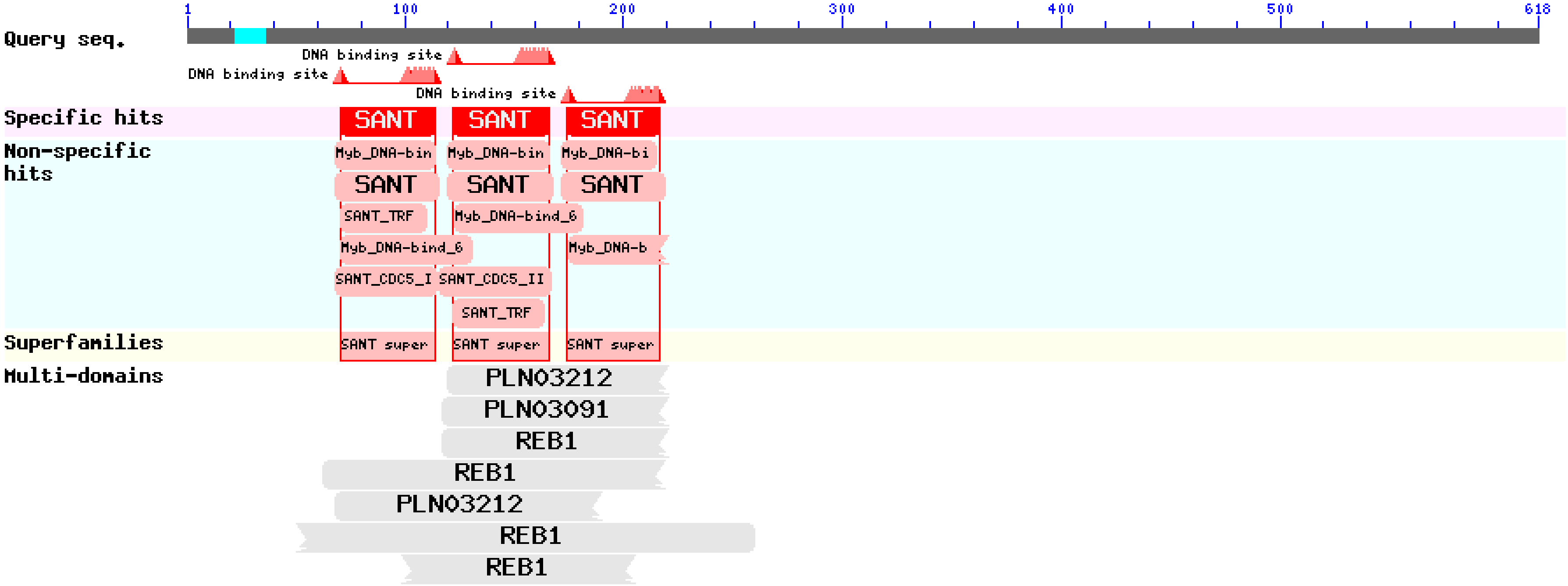

SiMYB070

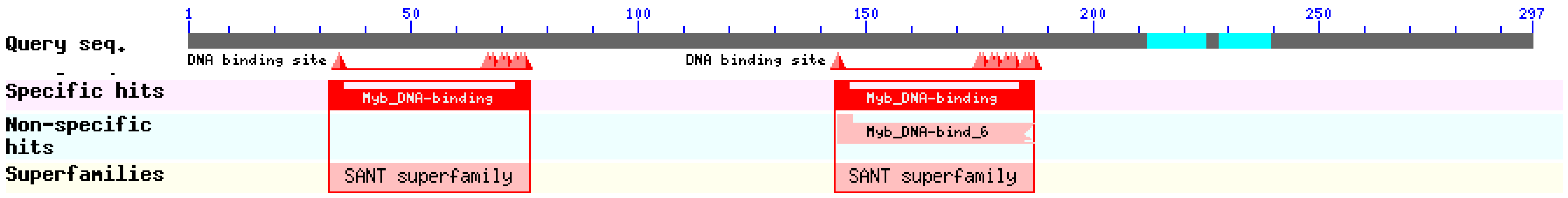

SiMYB071

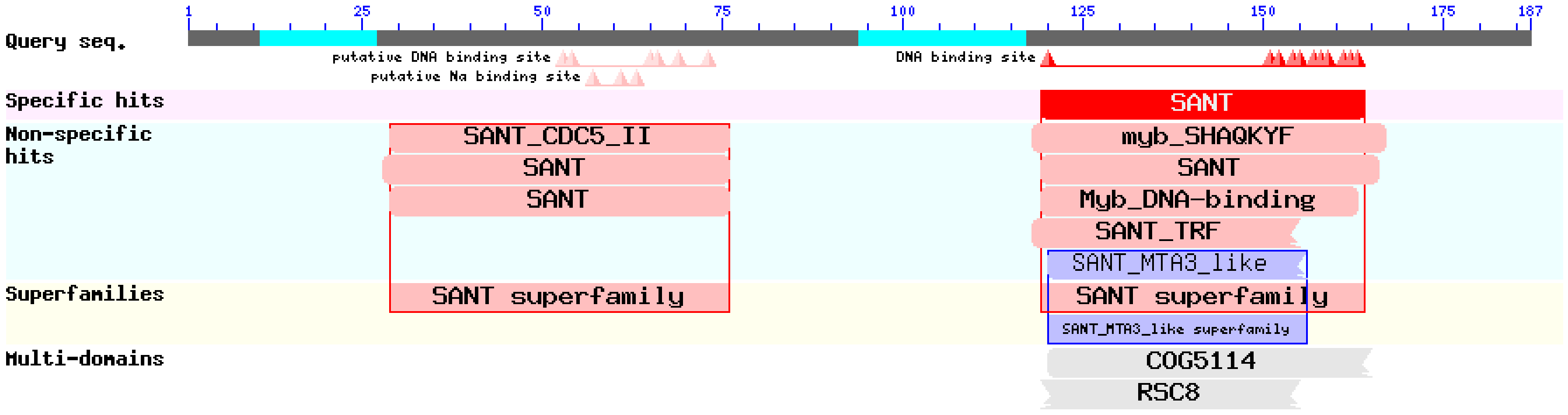

SiMYB072

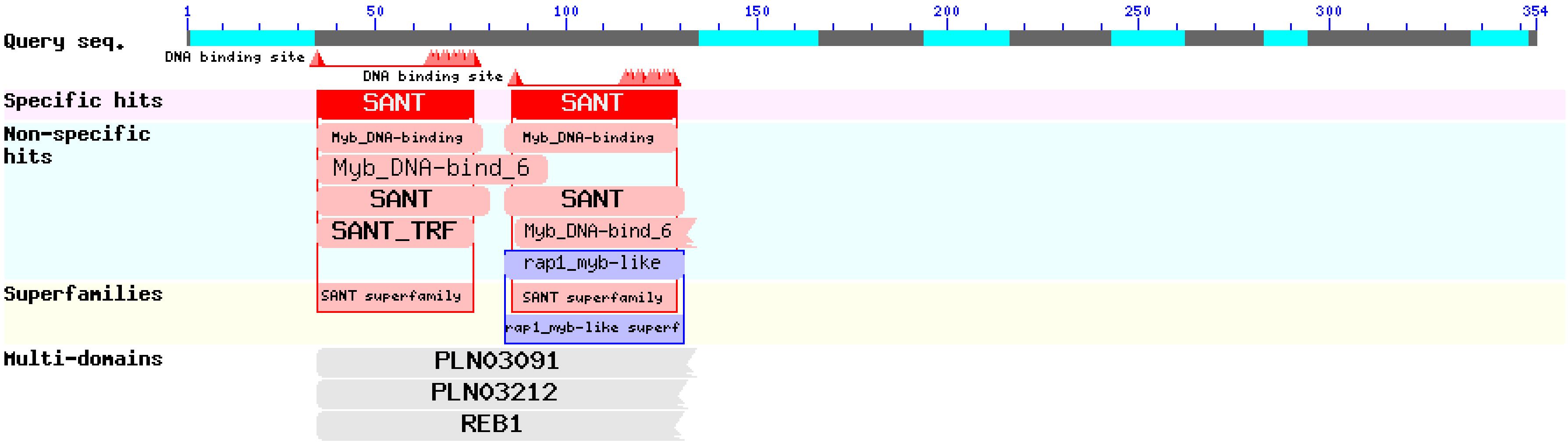

SiMYB073

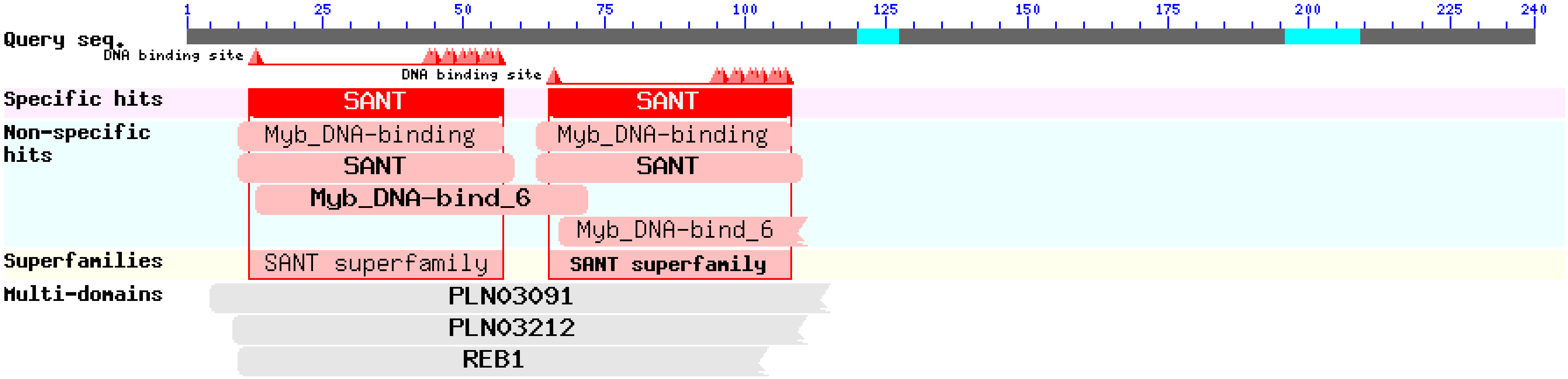

SiMYB074

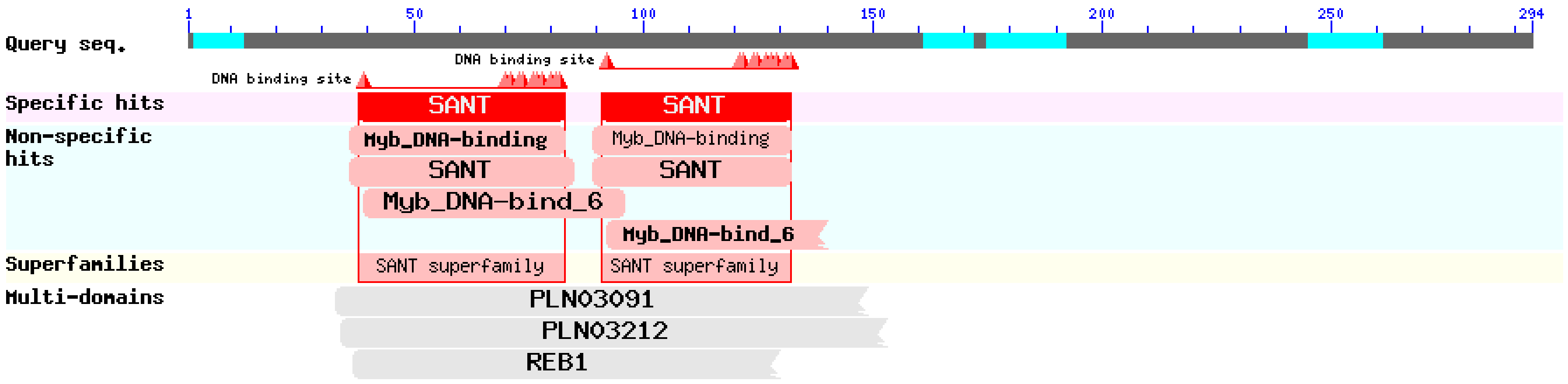

SiMYB075

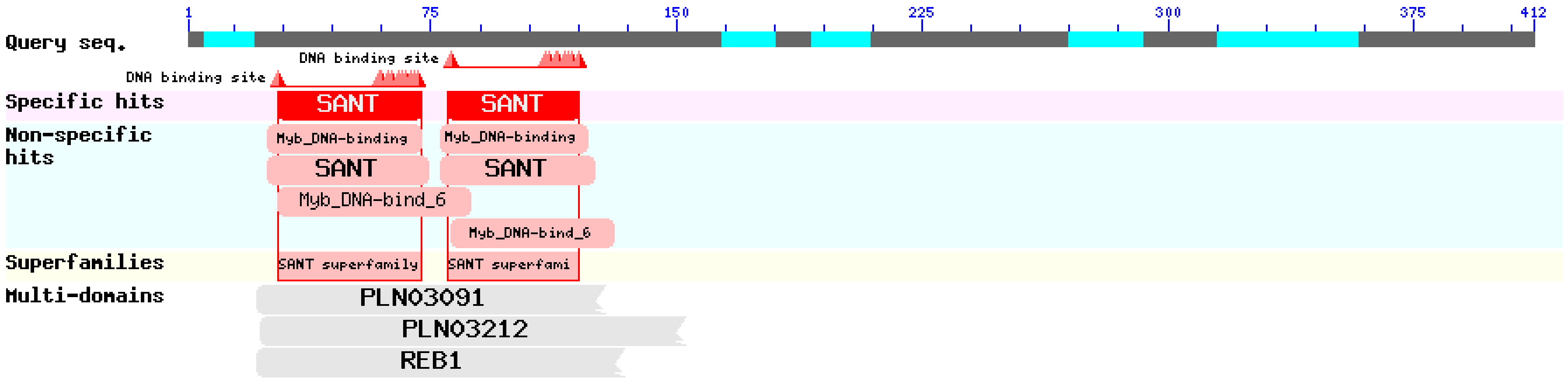

SiMYB076

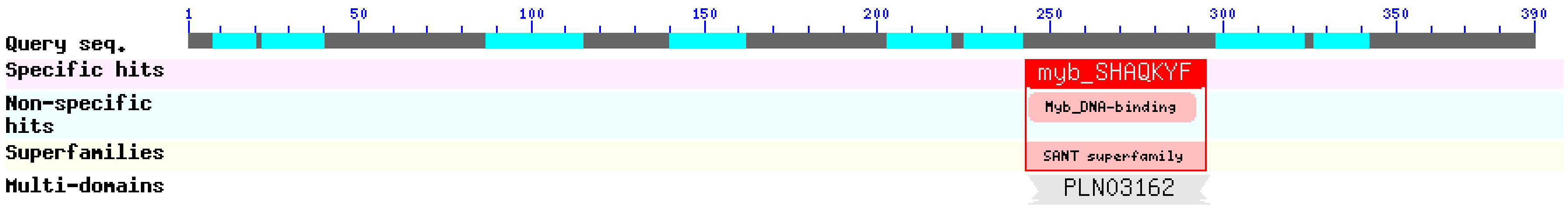

SiMYB077

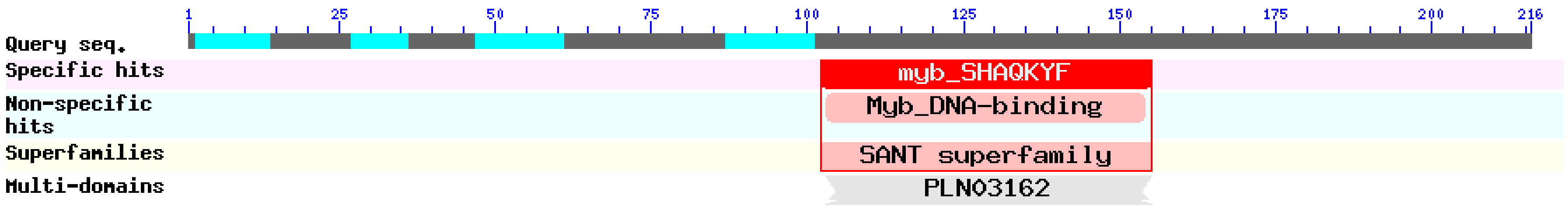

SiMYB078

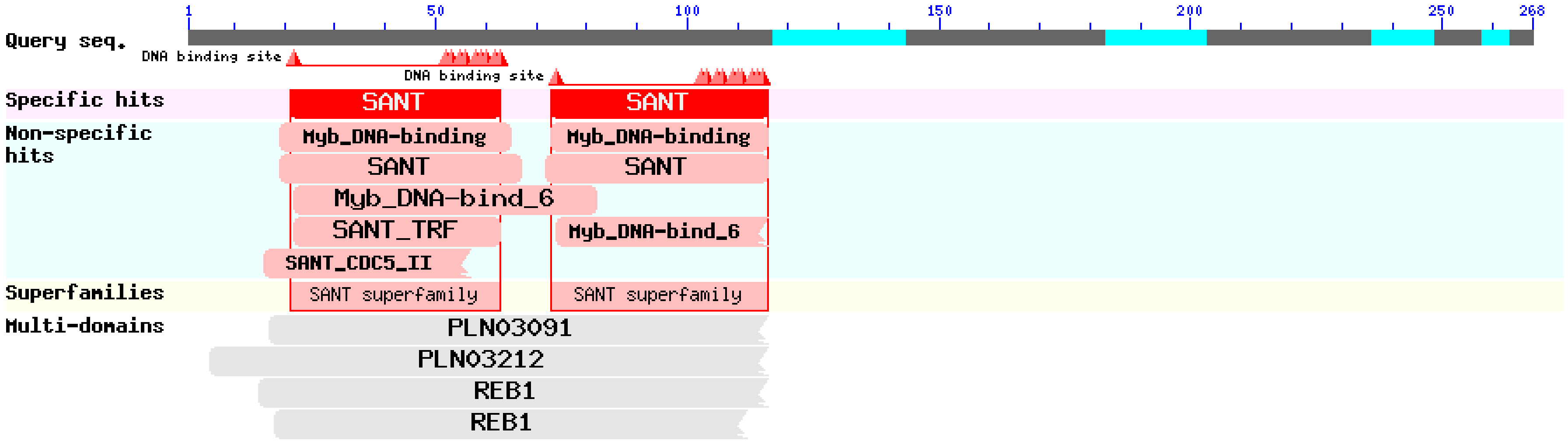

SiMYB079

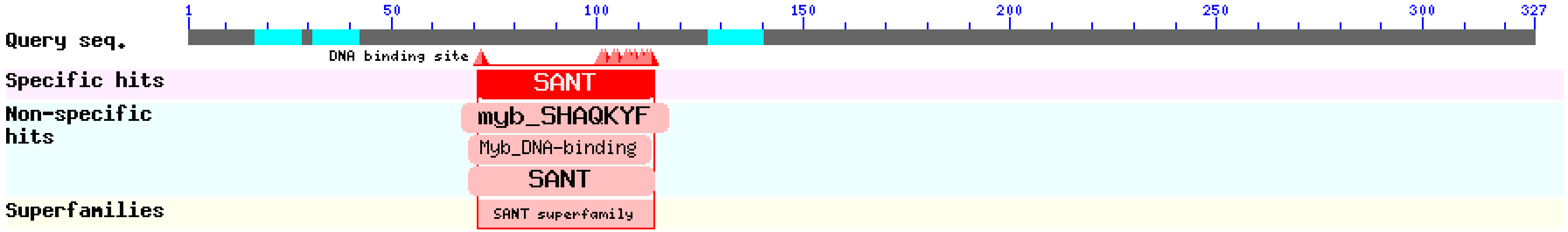

SiMYB080

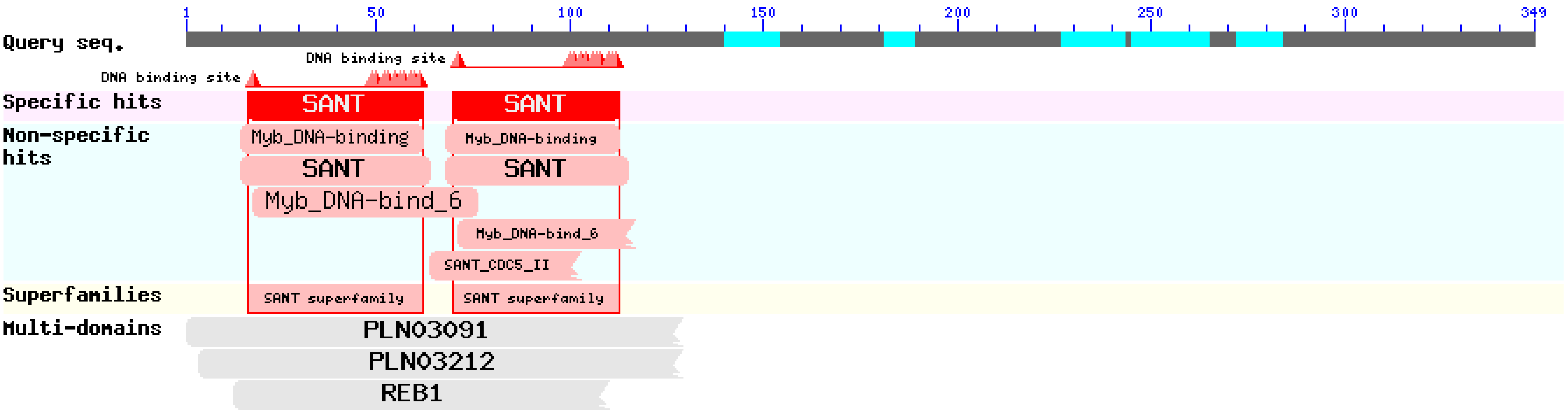

SiMYB081

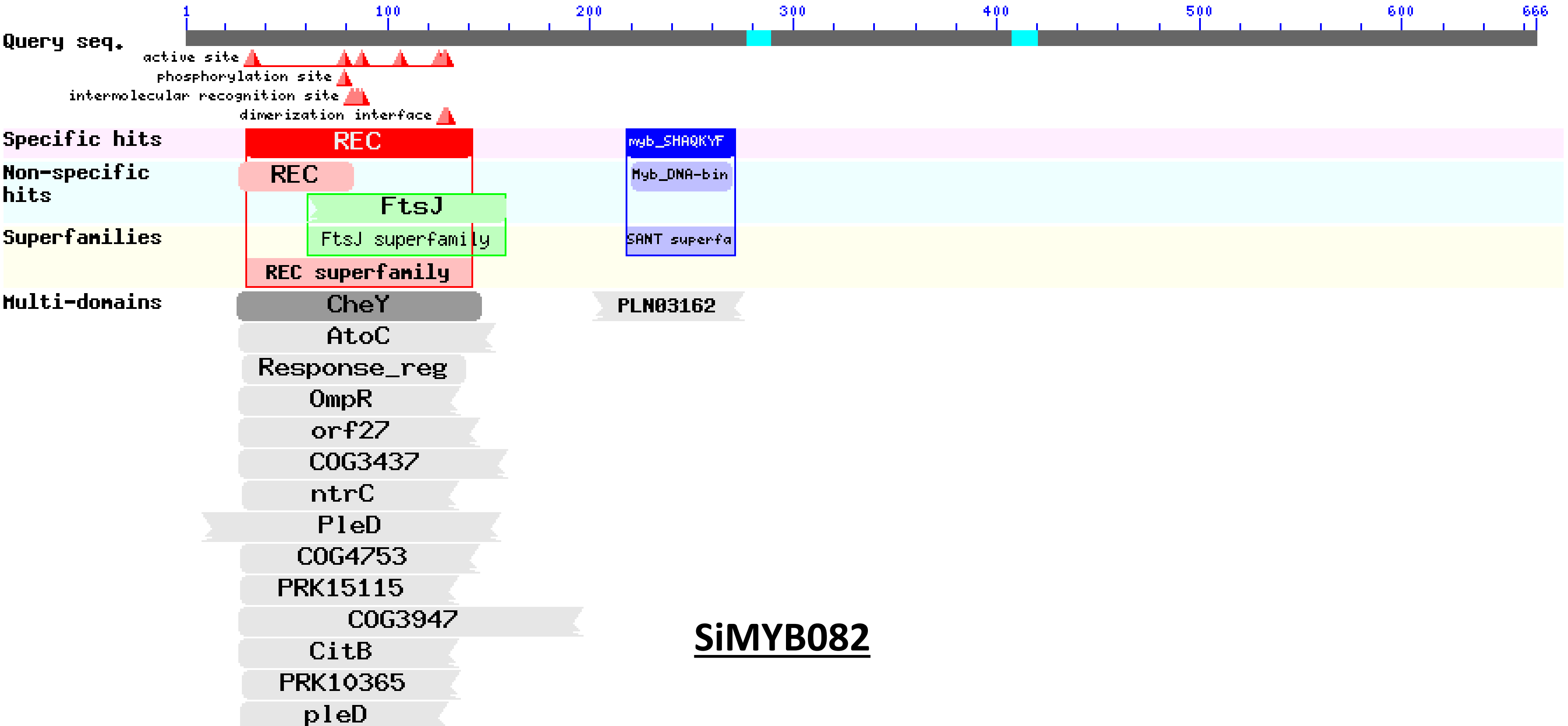

SiMYB082

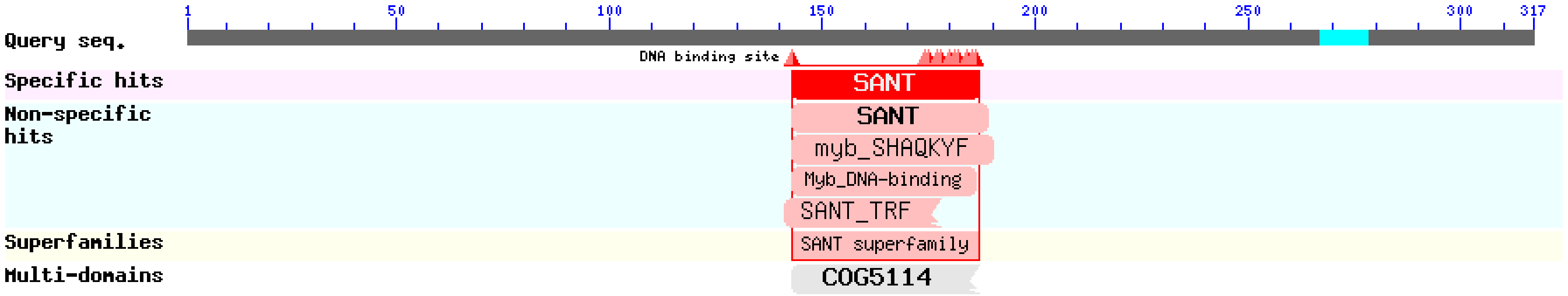

SiMYB083

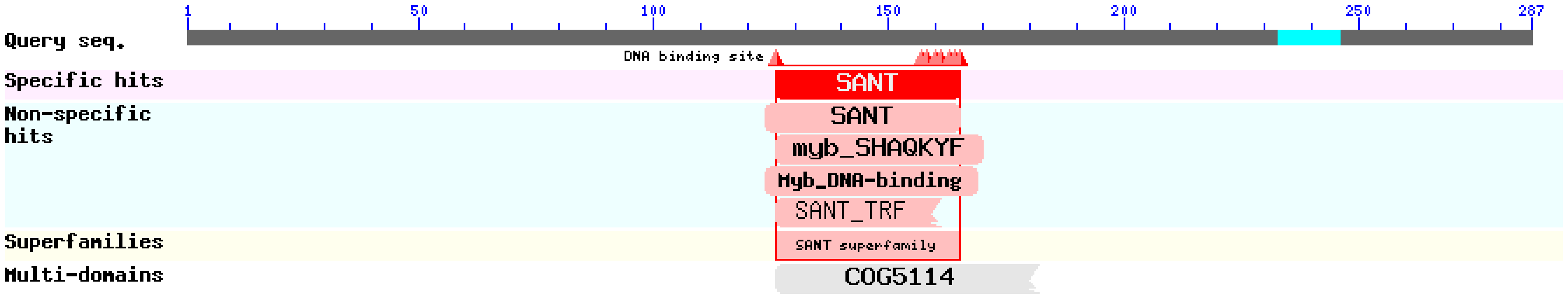

SiMYB084

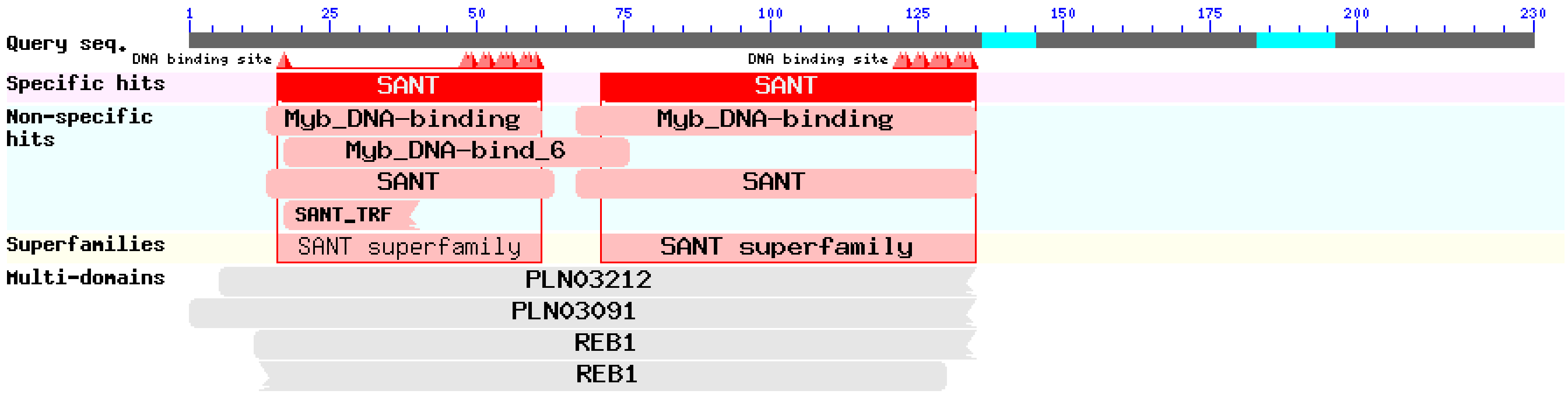

SiMYB085

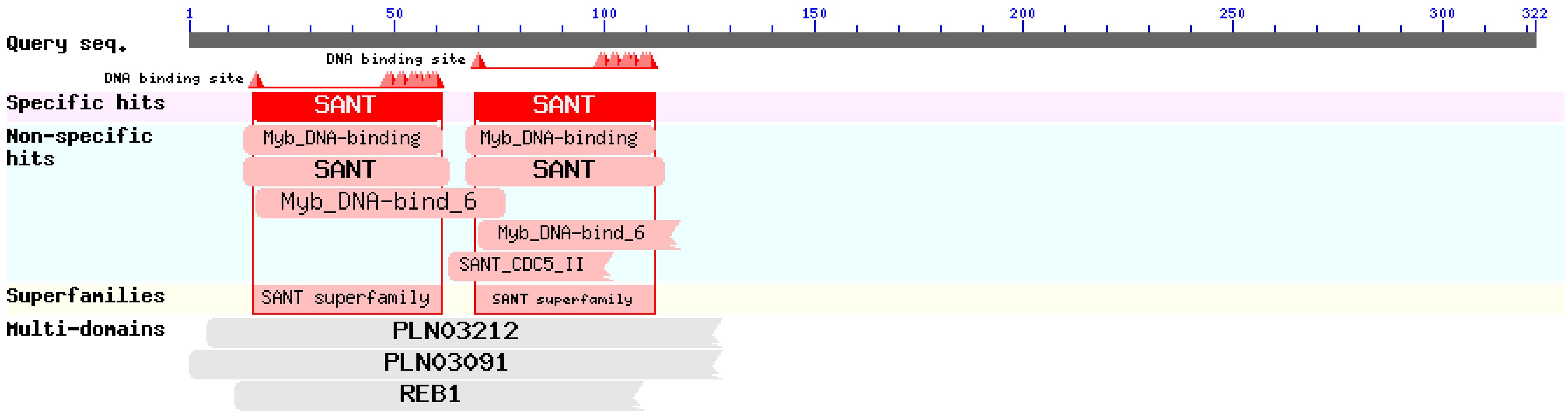

SiMYB086

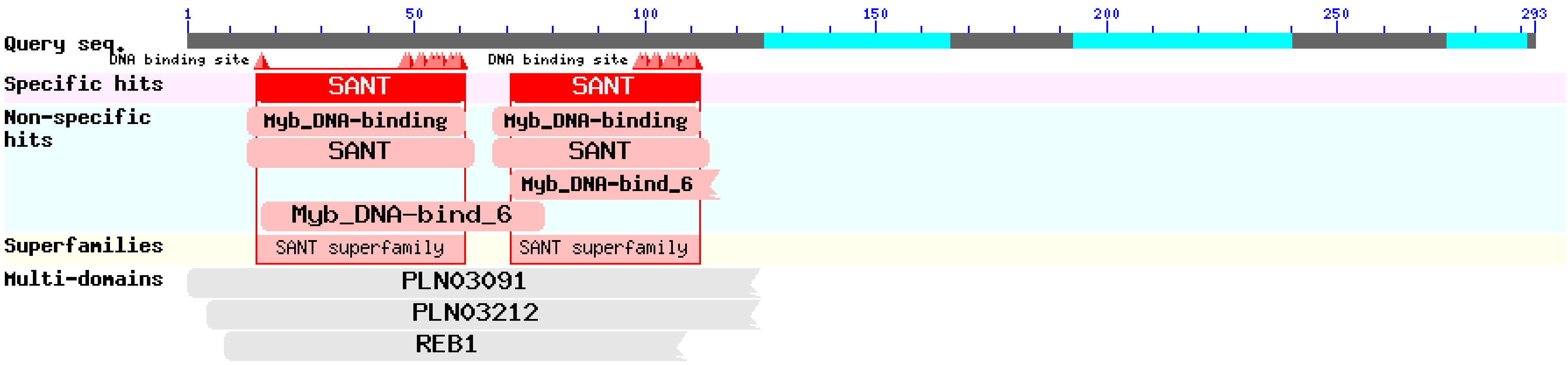

SiMYB087

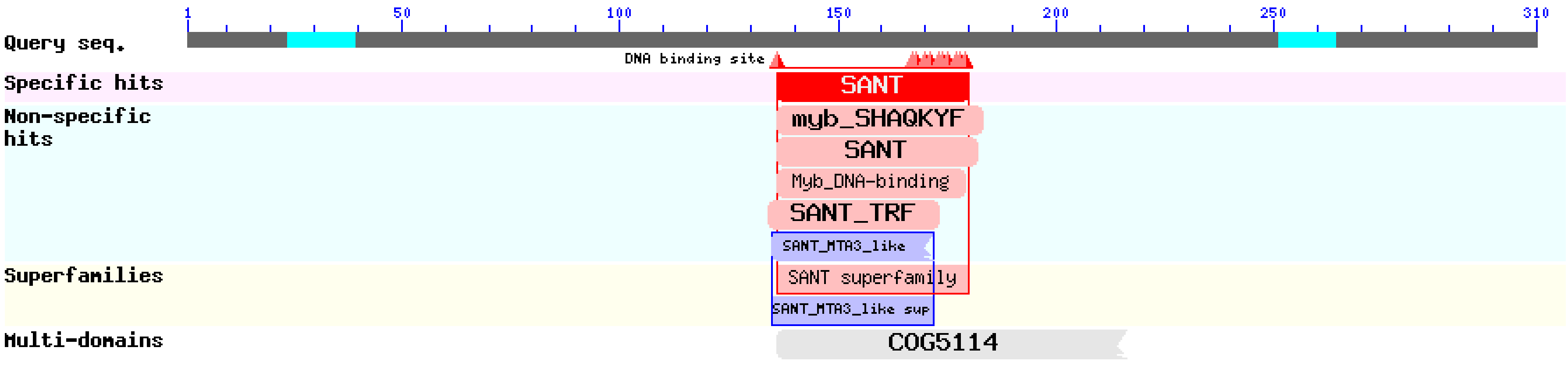

SiMYB088

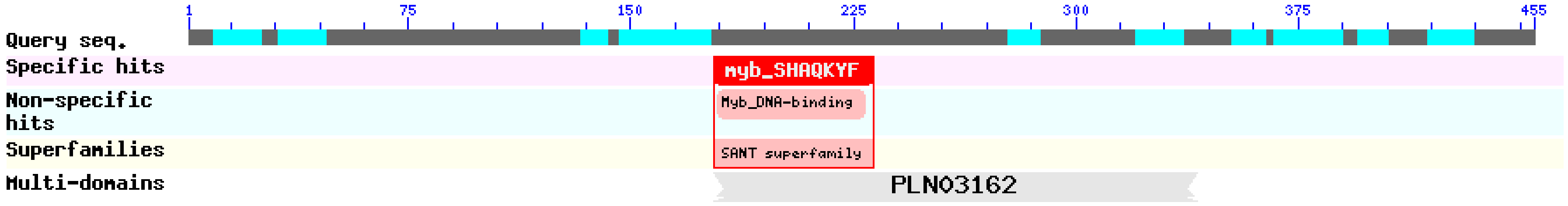

SiMYB089

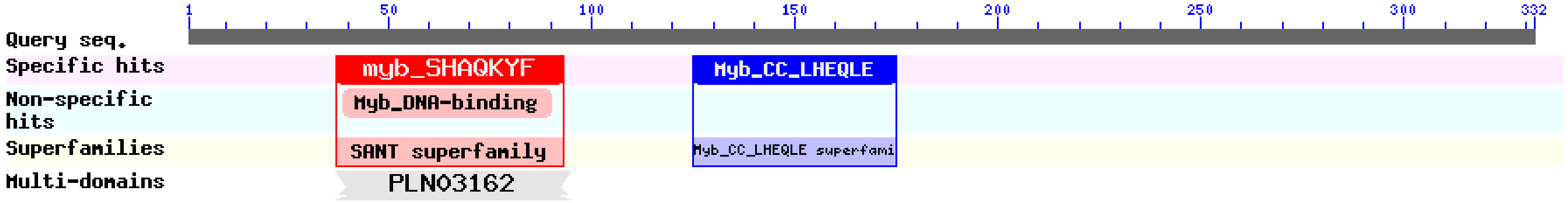

SiMYB090

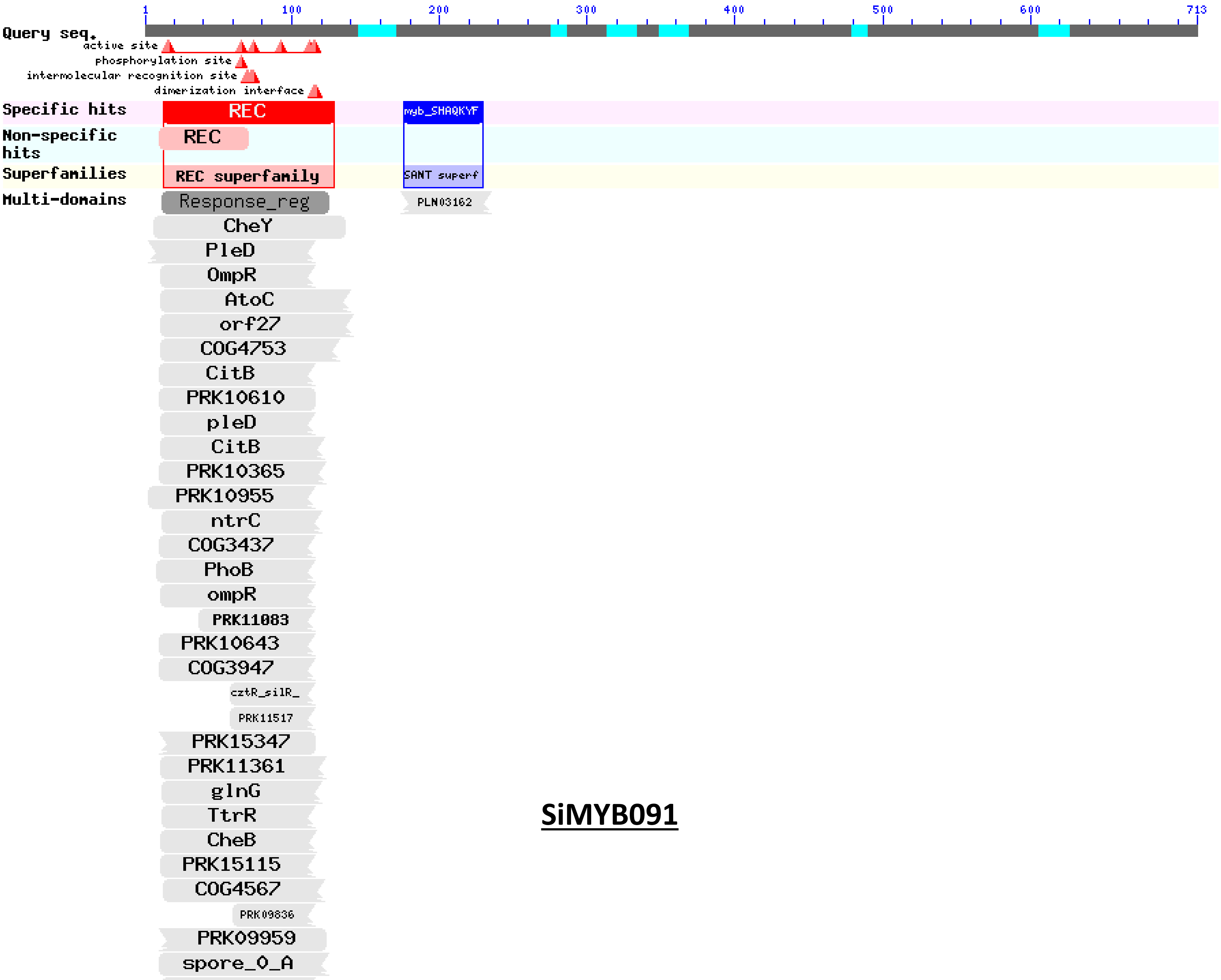
1
100
200
300
400
500
600
713
Query seq.
active site
phosphorylation site
intermolecular recognition site
dimerization interface
Specific hits
REC
myb_SHAQKYF
Non-specific hits
REC
Superfamilies
REC superfamily
SANT superf
Multi-domains
Response_reg
PLN03162
CheY
PleD
OmpR
AtoC
orf27
COG4753
CitB
PRK10610
pleD
CitB
PRK10365
PRK10955
ntrC
COG3437
PhoB
ompR
PRK11083
PRK10643
COG3947
cztR_silR_
PRK11517
PRK15347
PRK11361
glnG
TtrR
CheB
PRK15115
COG4567
PRK09836
PRK09959
spore_0_A

SiMYB091

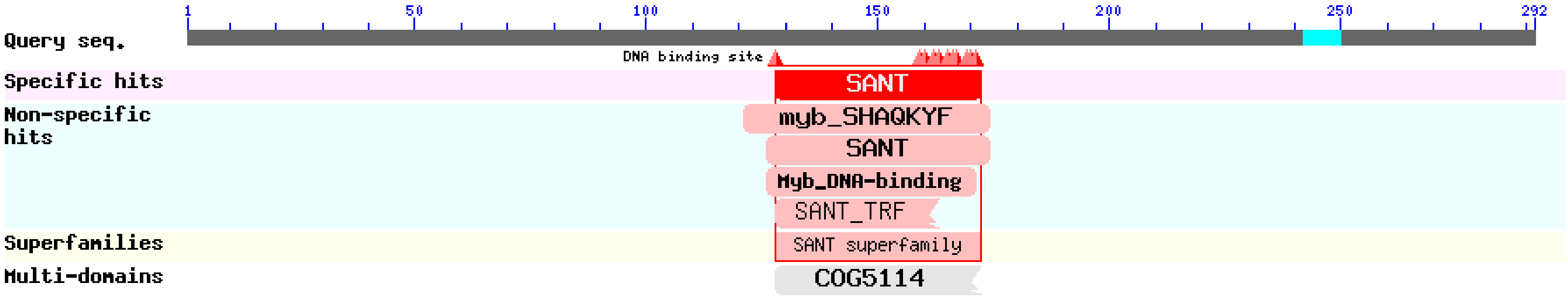

SiMYB092

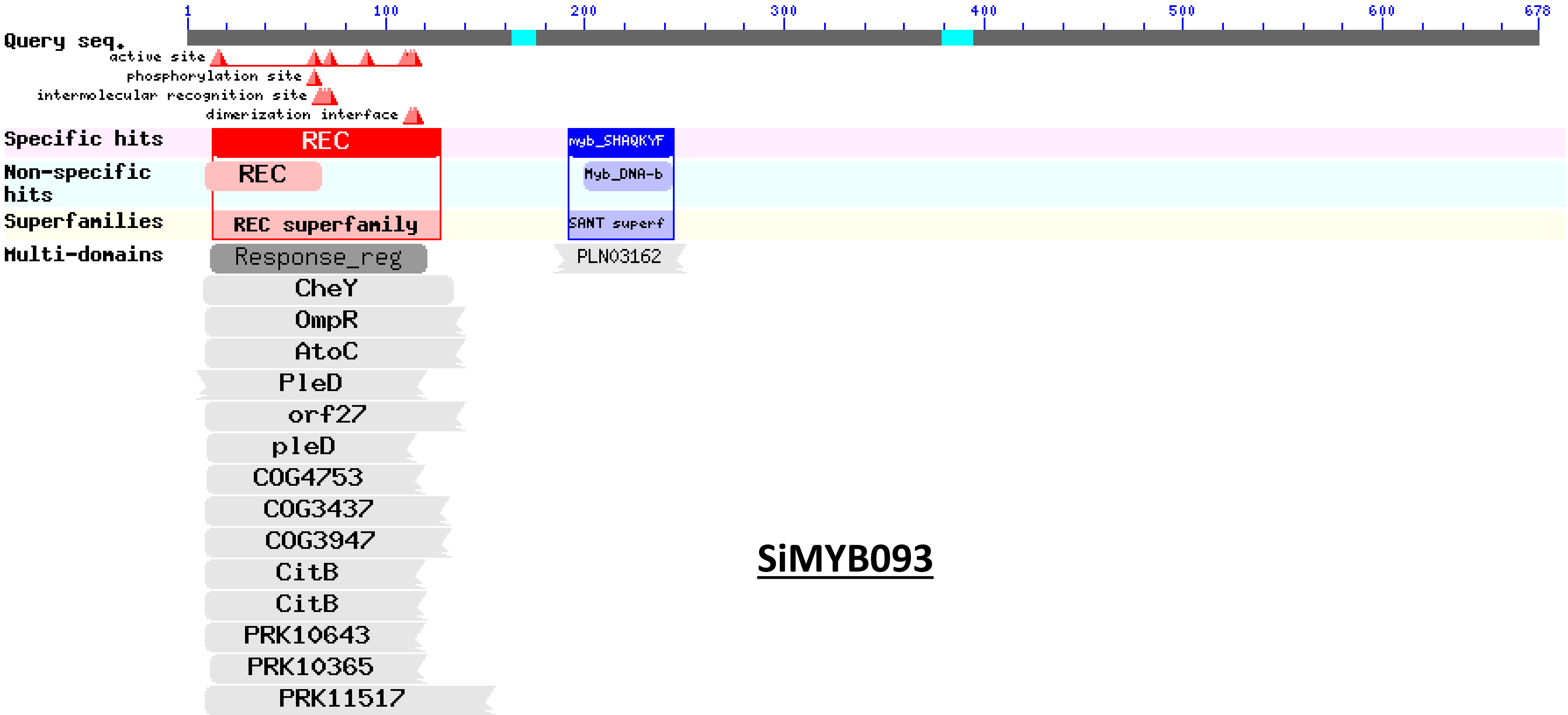

SiMYB093

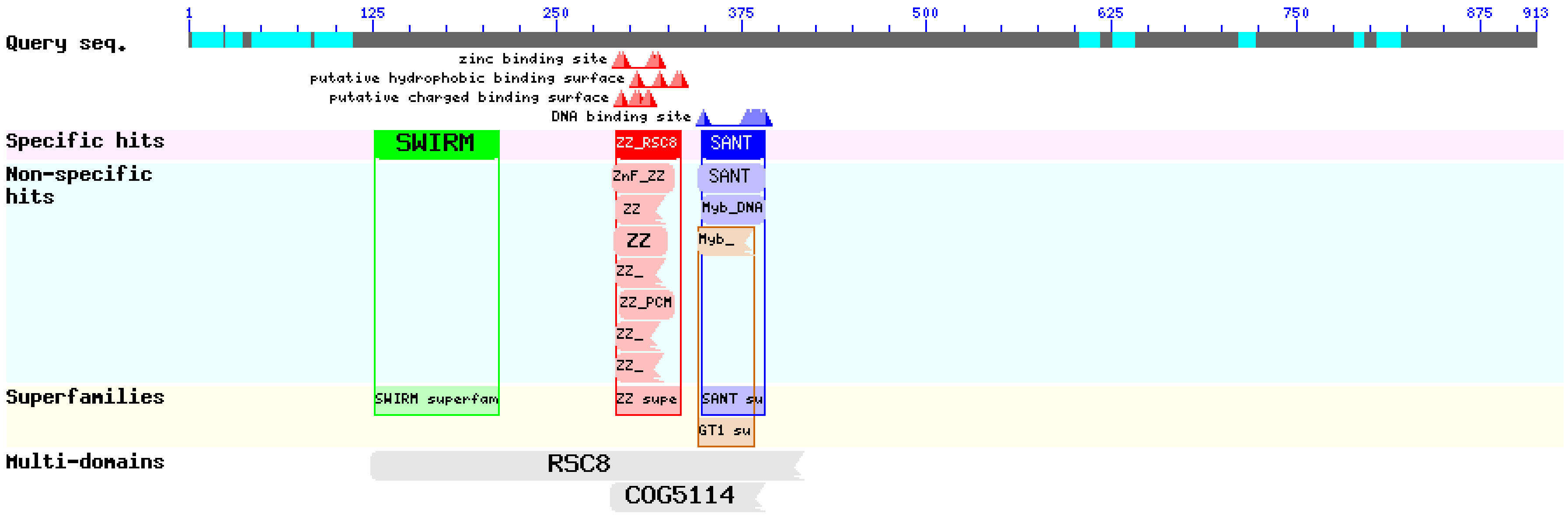

SiMYB094

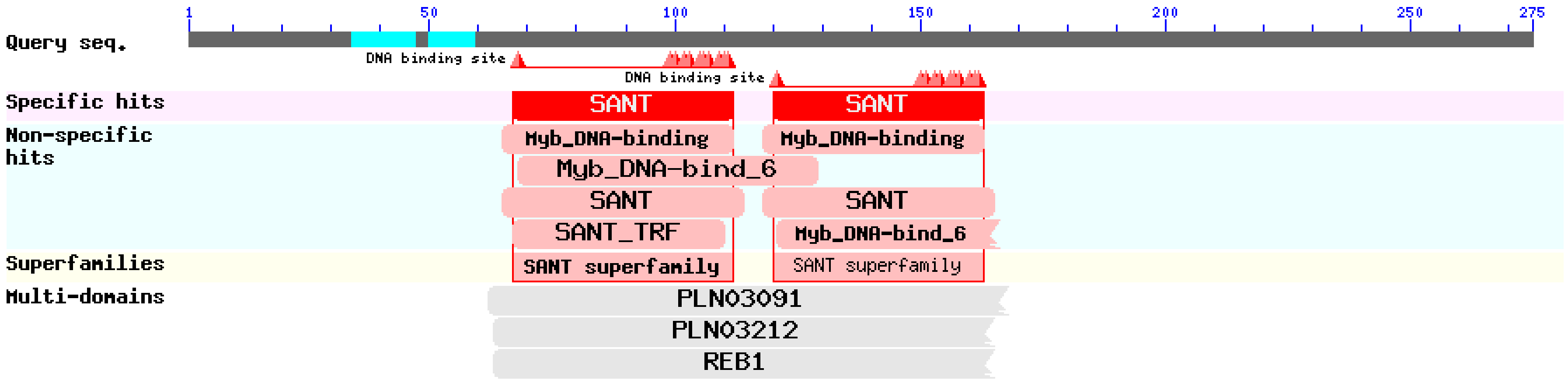

SiMYB095

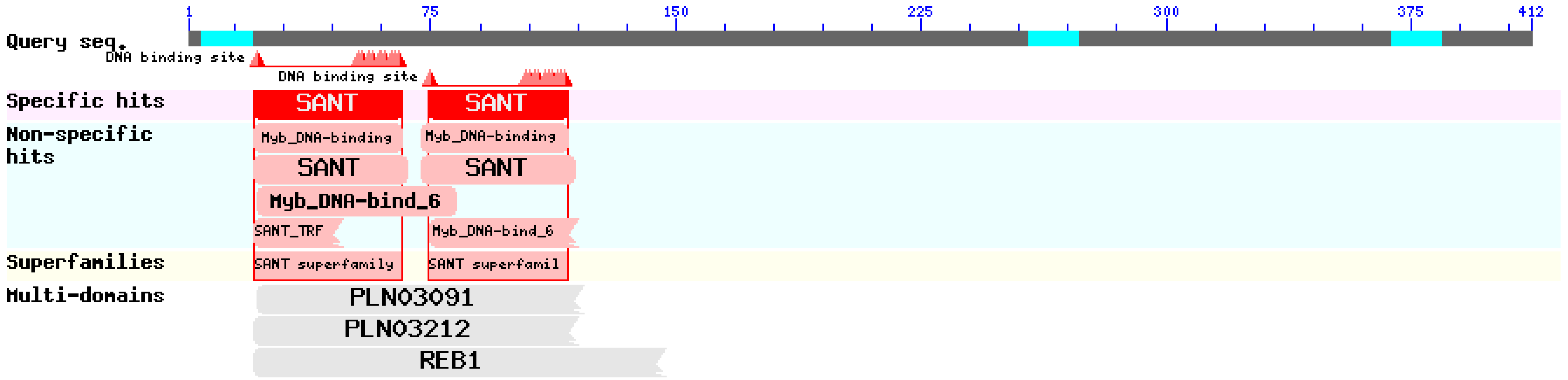

SiMYB096

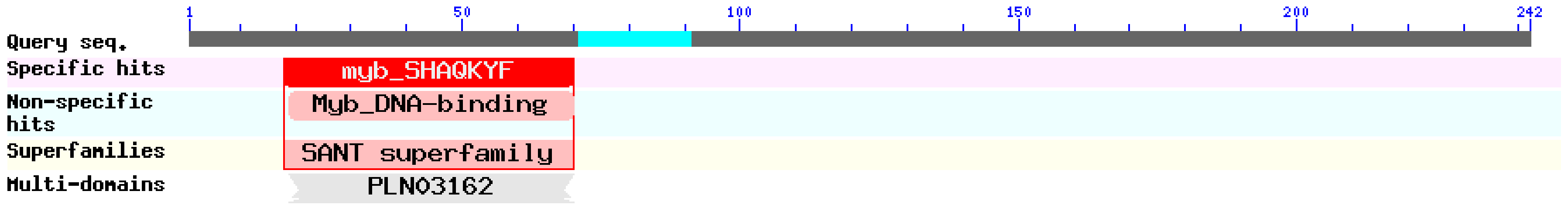

SiMYB097

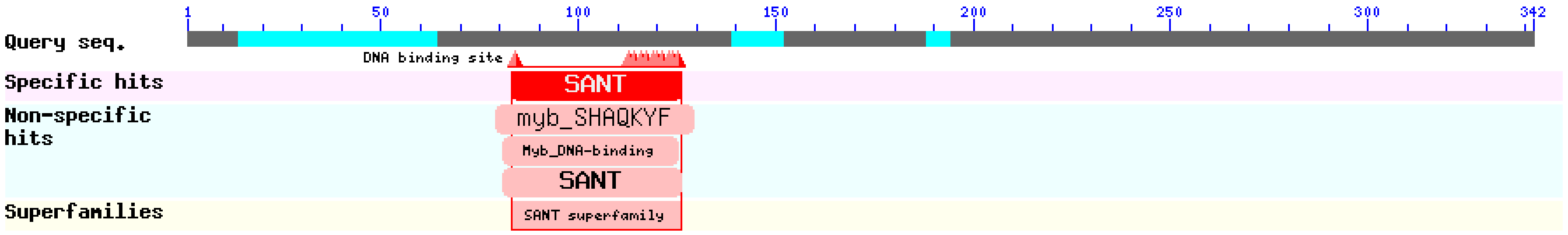

SiMYB098

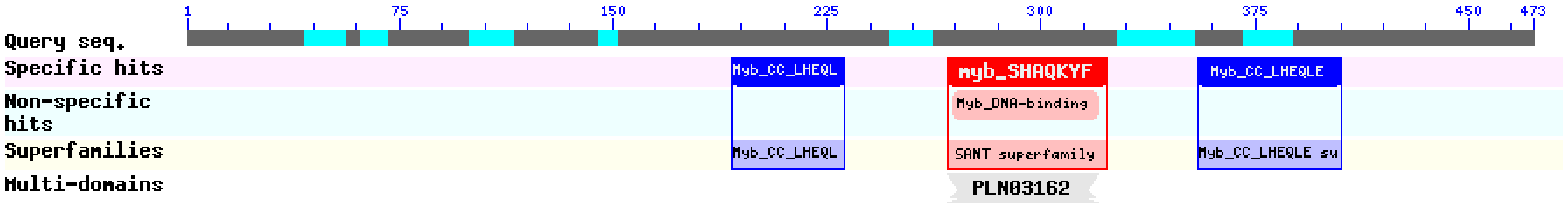

SiMYB099

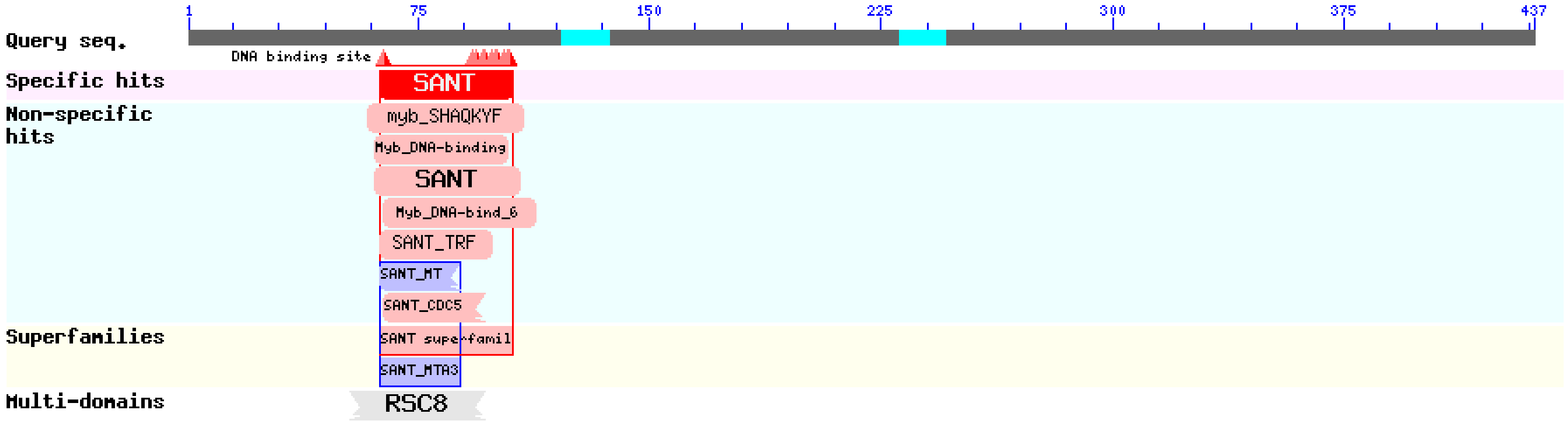

SiMYB100

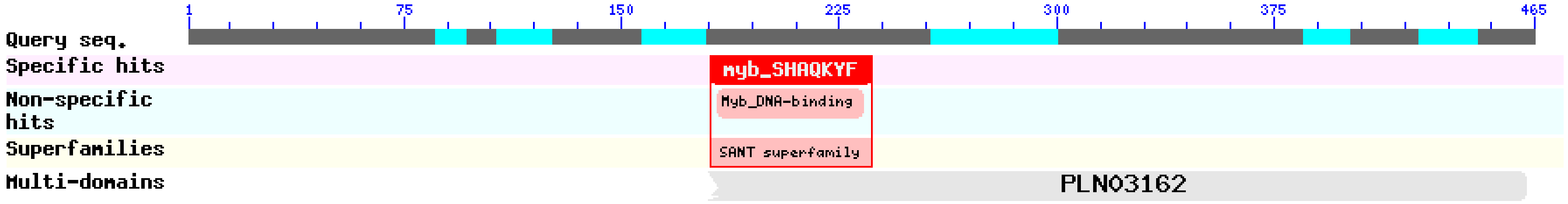

SiMYB101

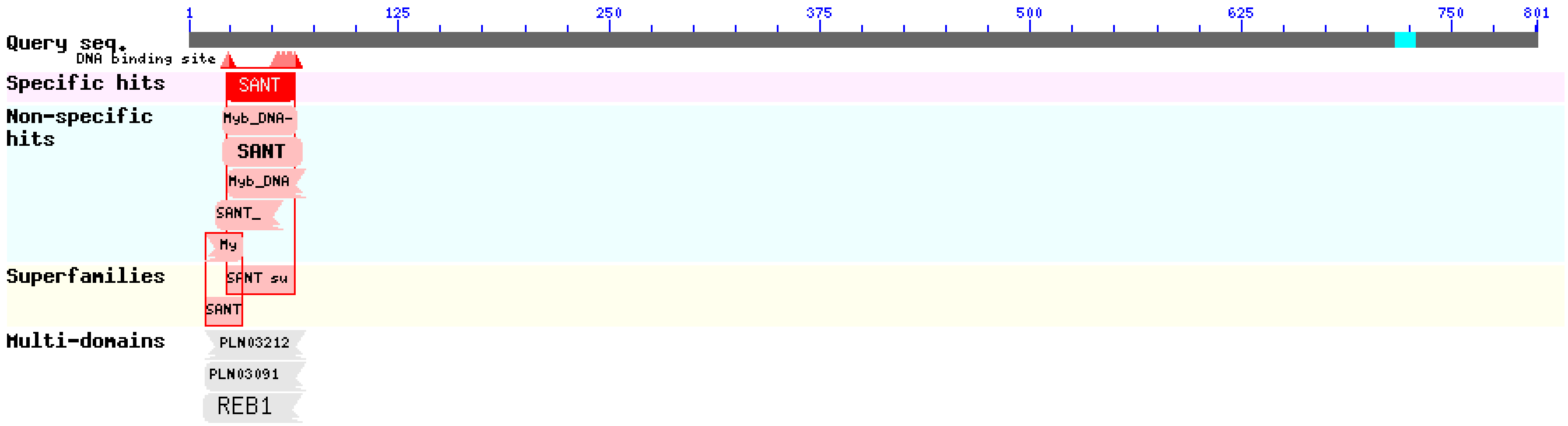

SiMYB102

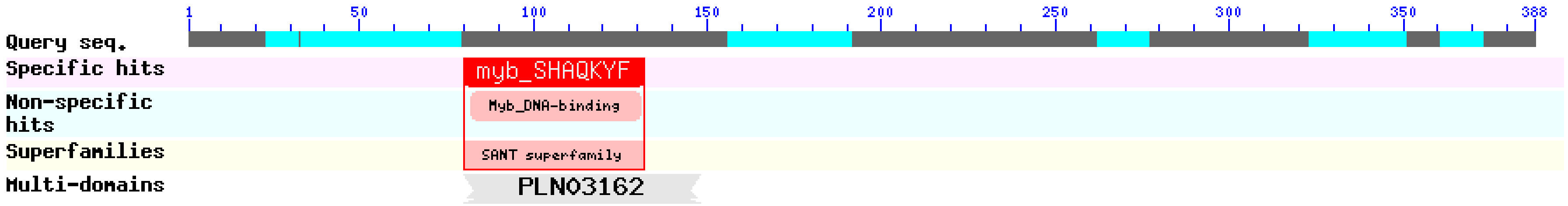

SiMYB103

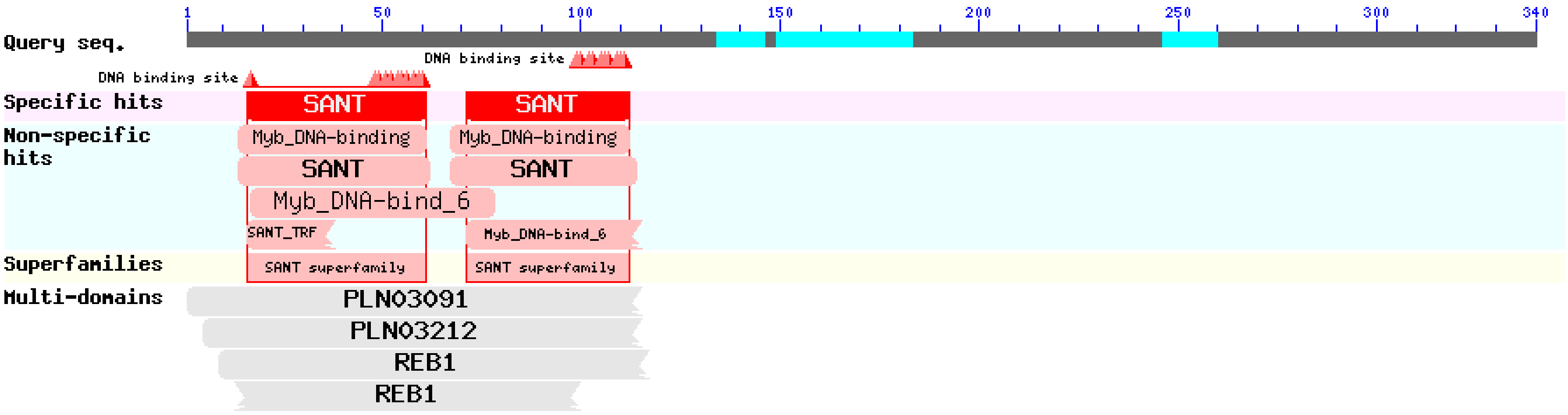

SiMYB104

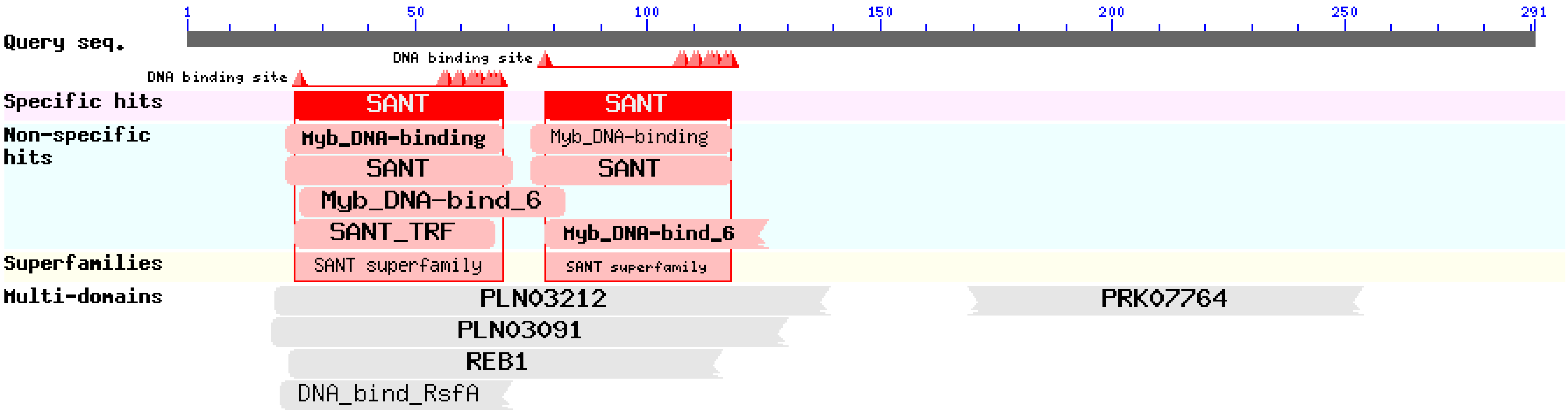

SiMYB105

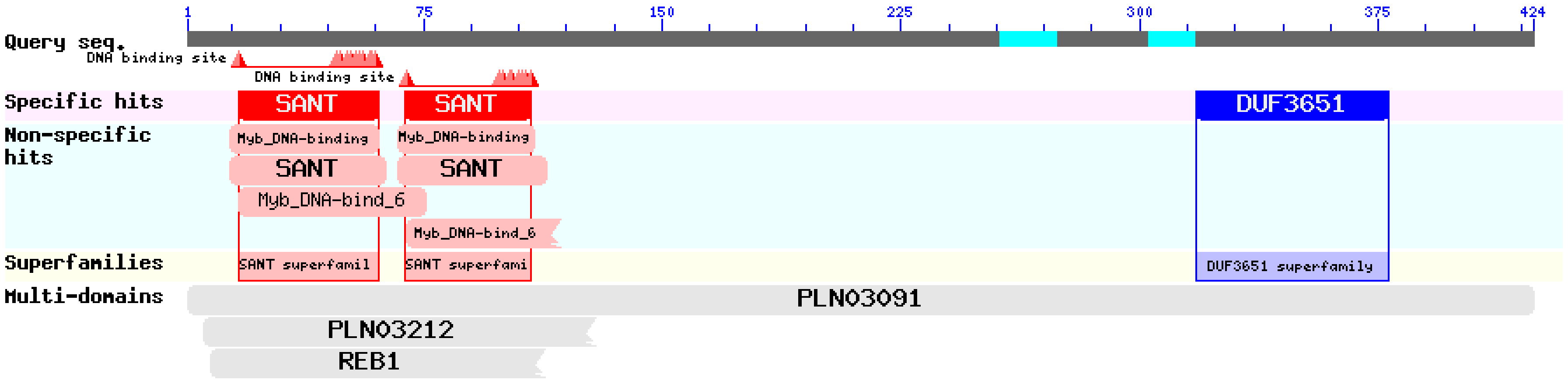

SiMYB106

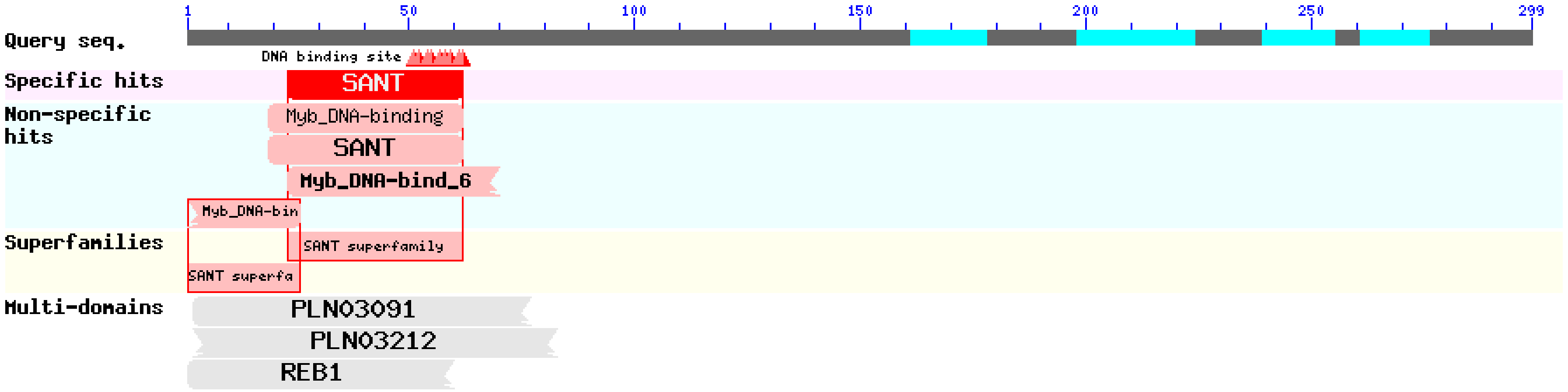

SiMYB107

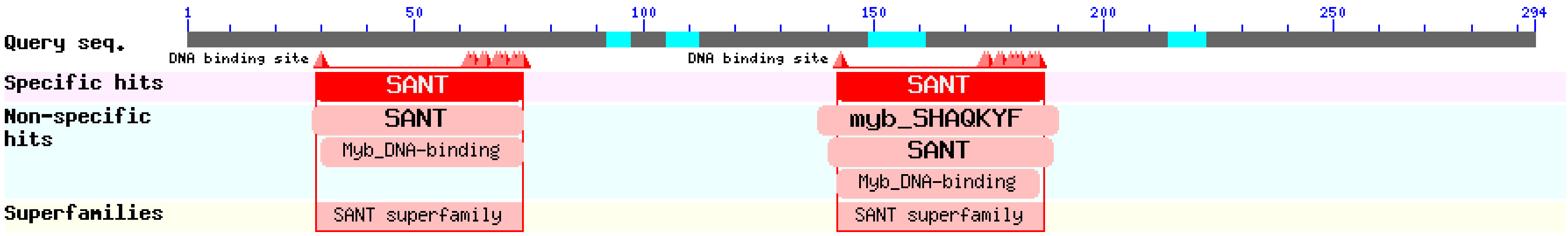

SiMYB108

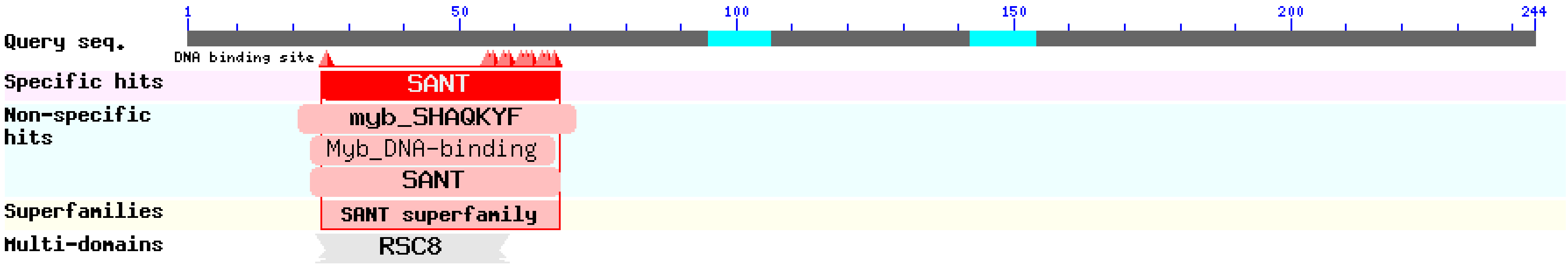

SiMYB109

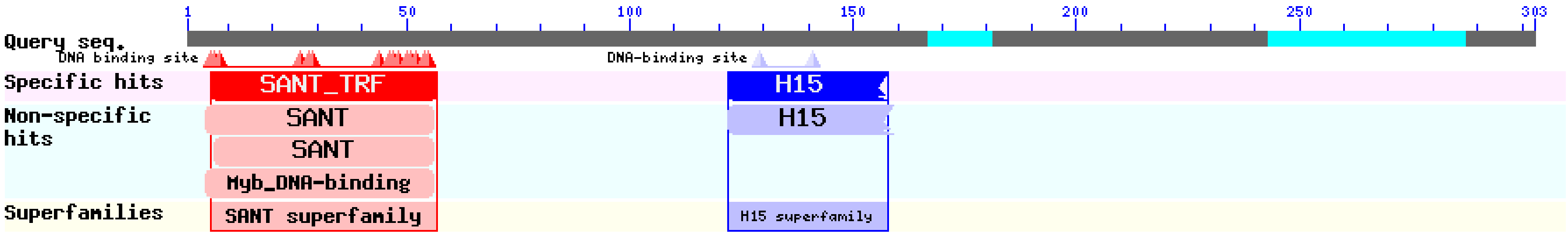

SiMYB110

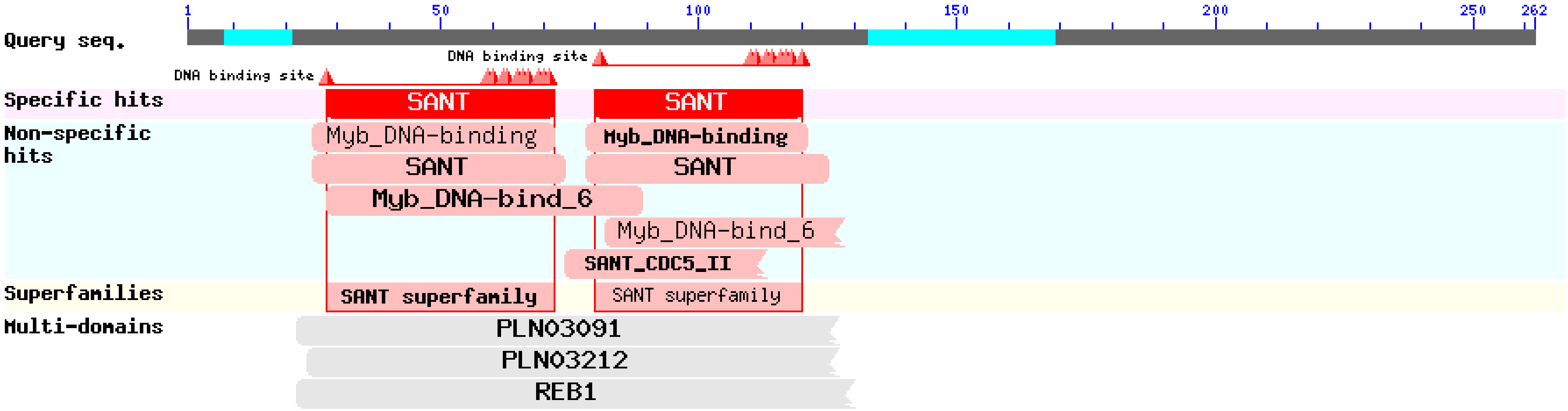

SiMYB111

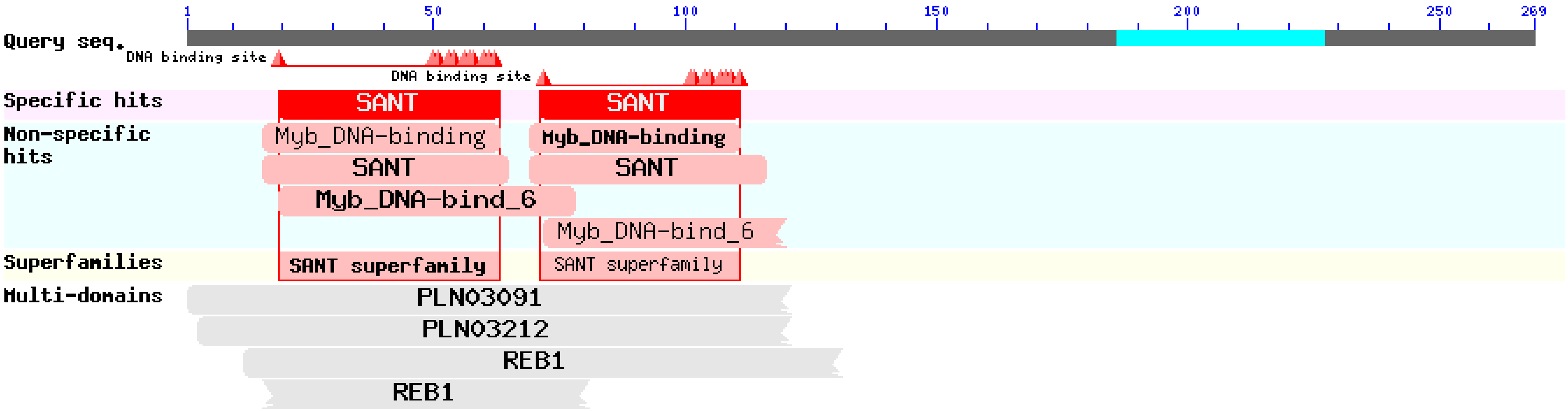

SiMYB112

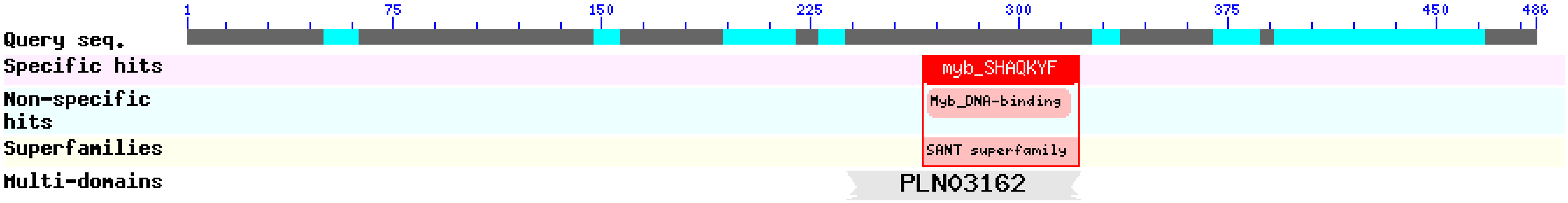

SiMYB113

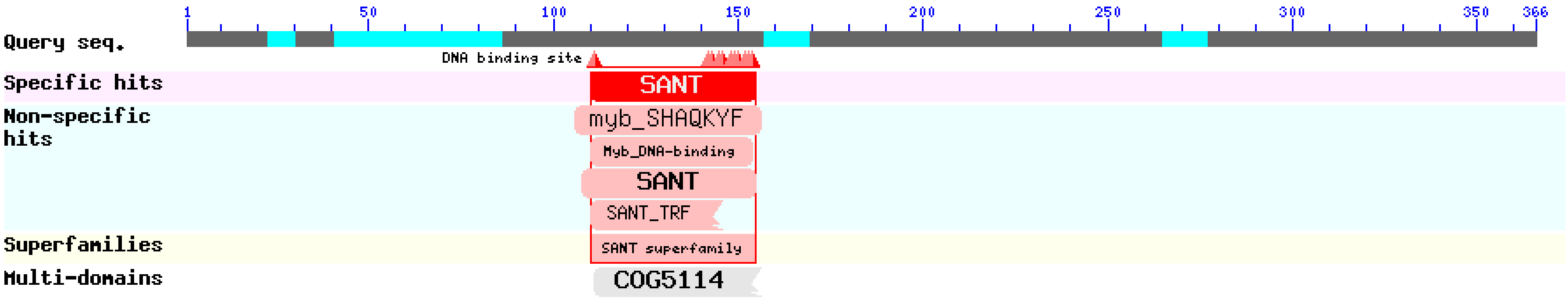

SiMYB114

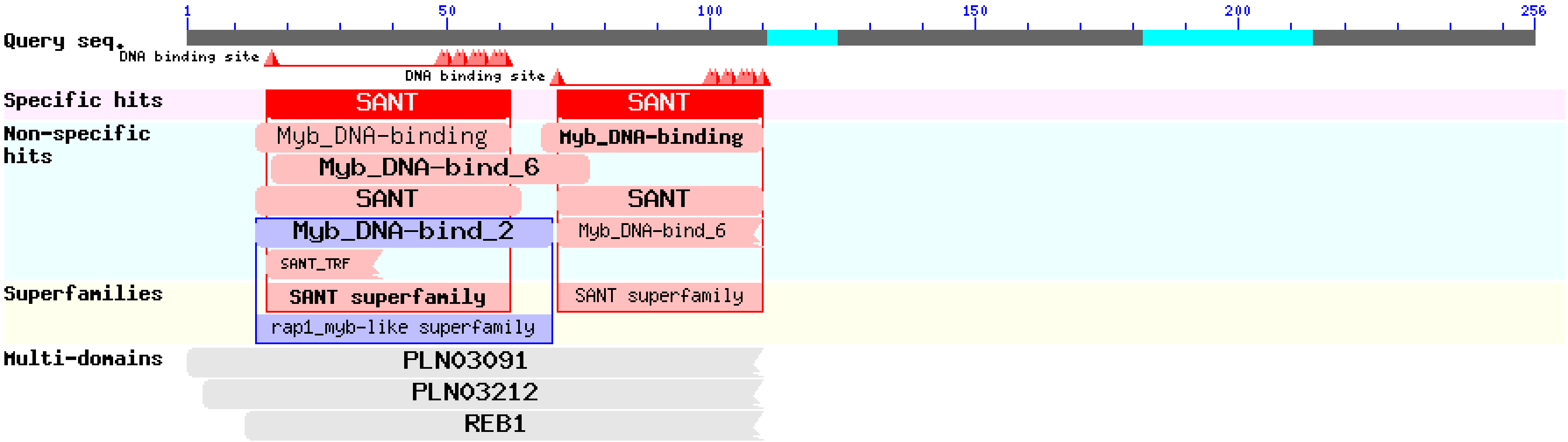

SiMYB115

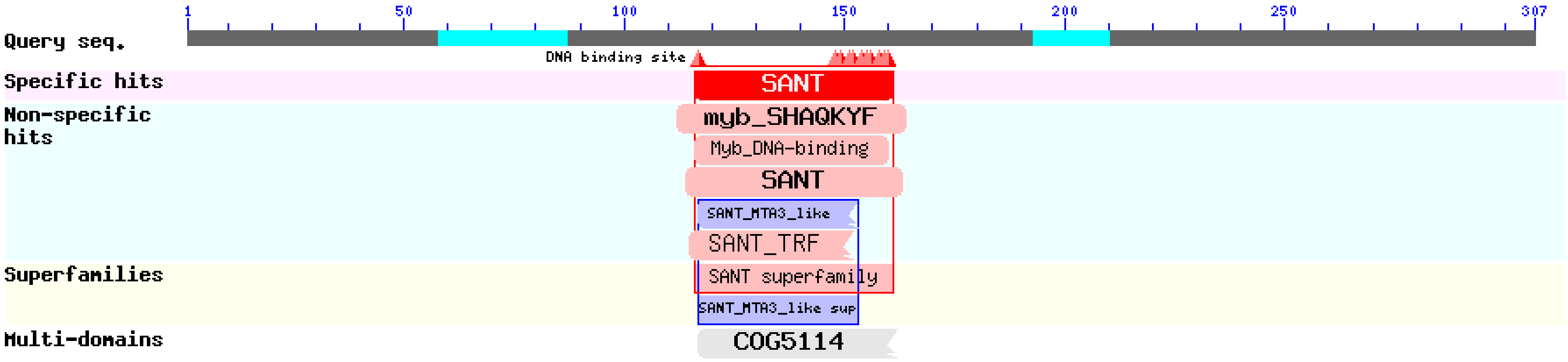

SiMYB116

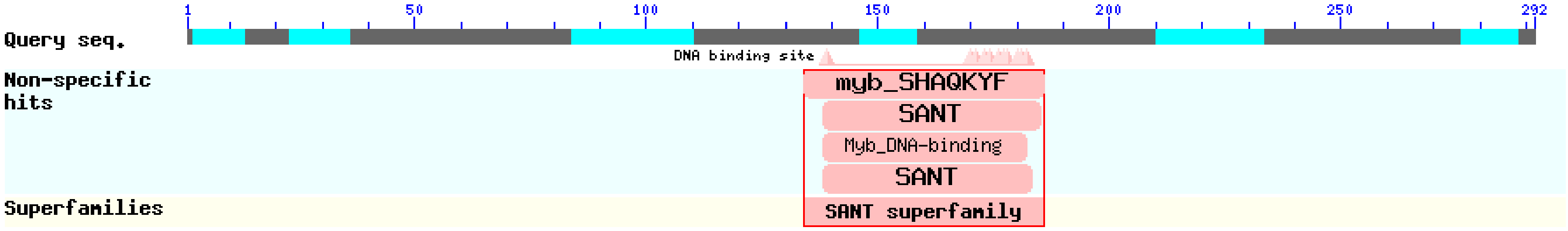

SiMYB117

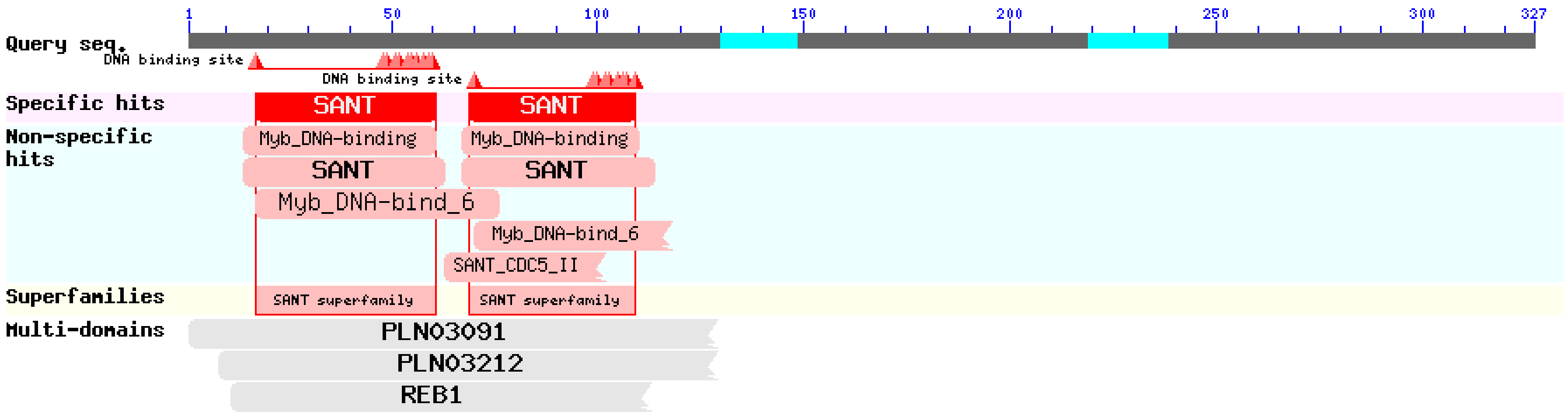

SiMYB118

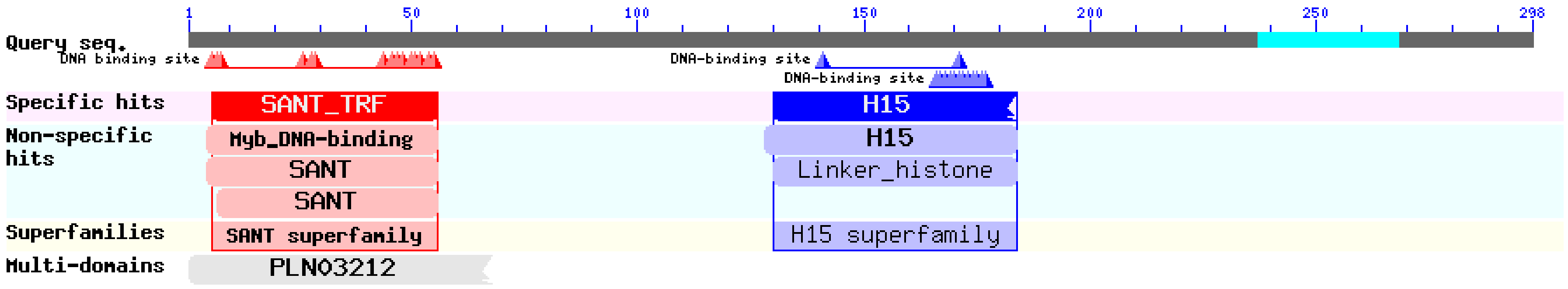

SiMYB119

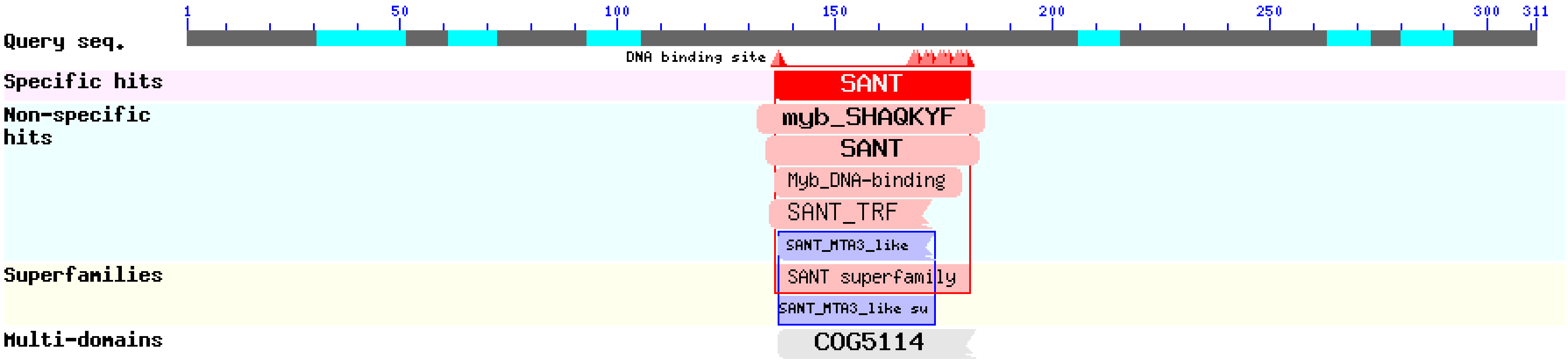

SiMYB120

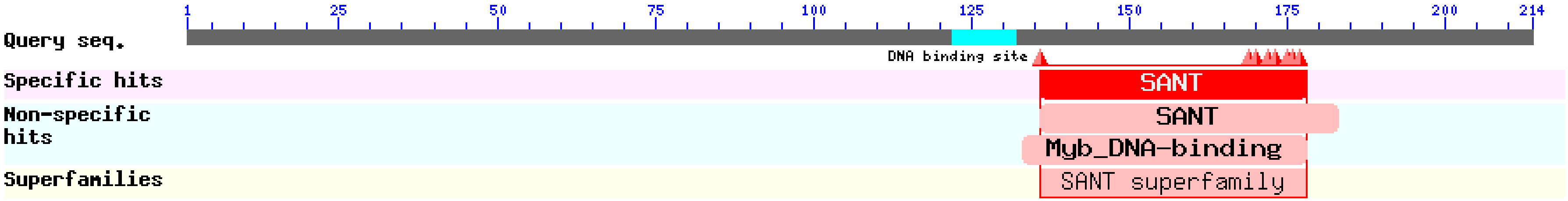

SiMYB121

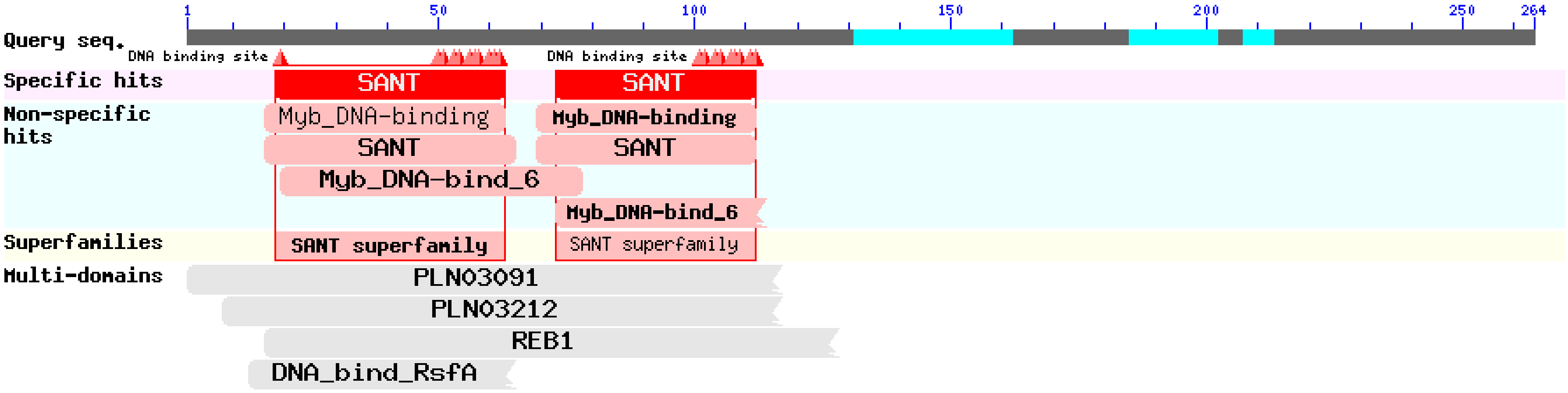

SiMYB122

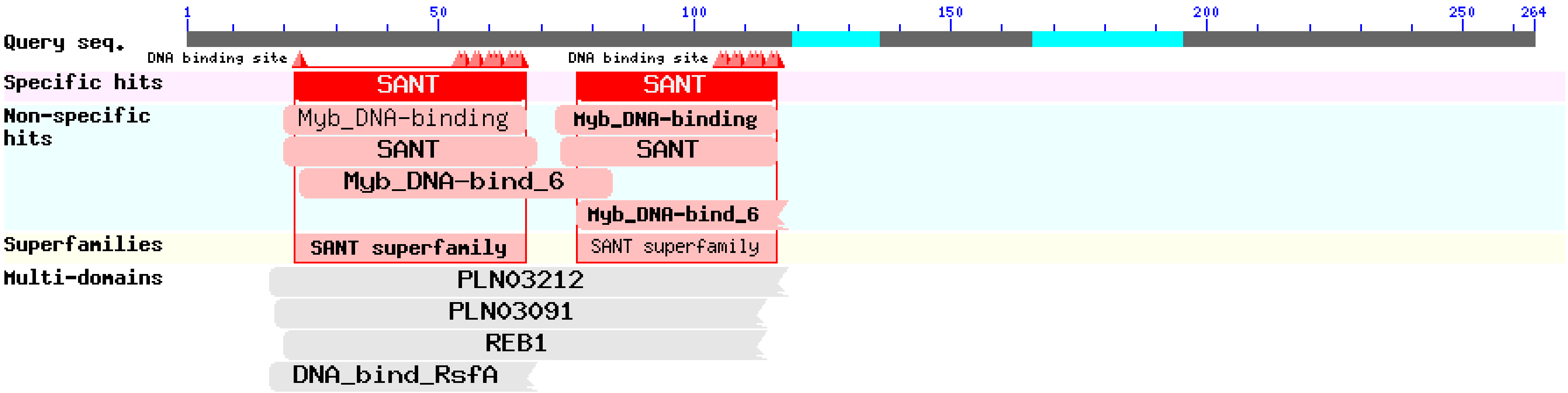

SiMYB123

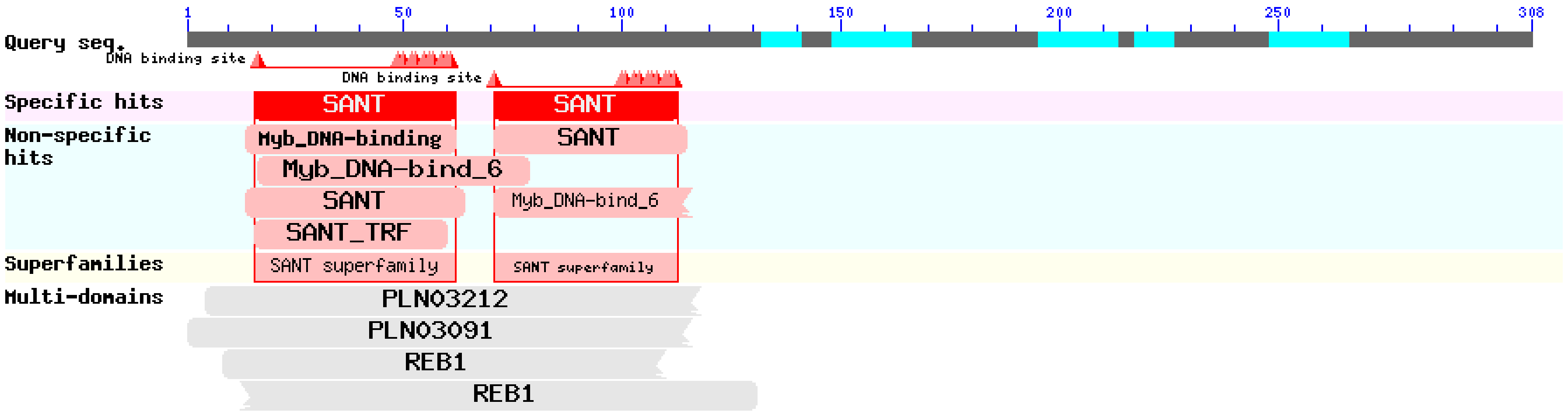

SiMYB124

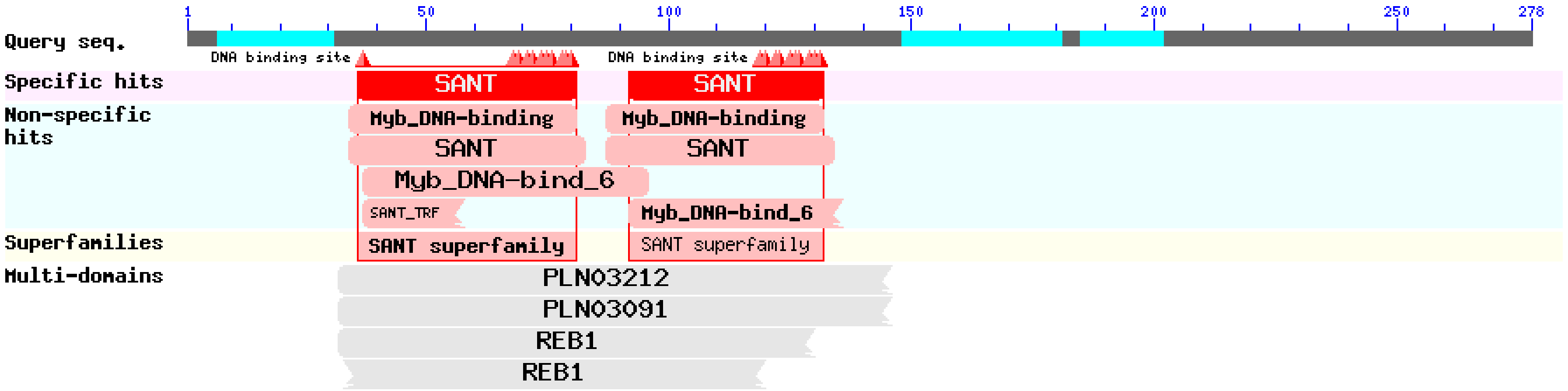

SiMYB125

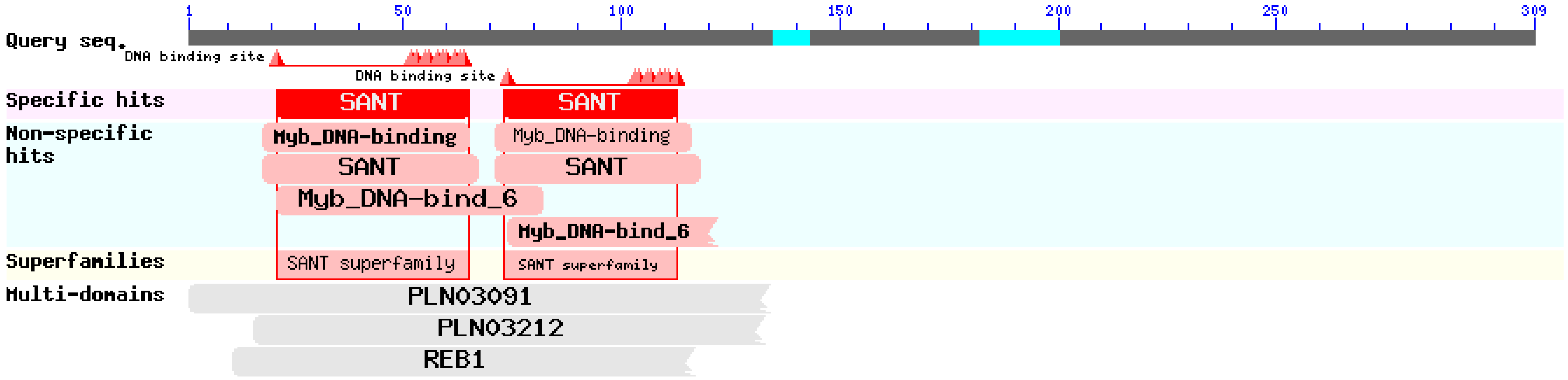

SiMYB126

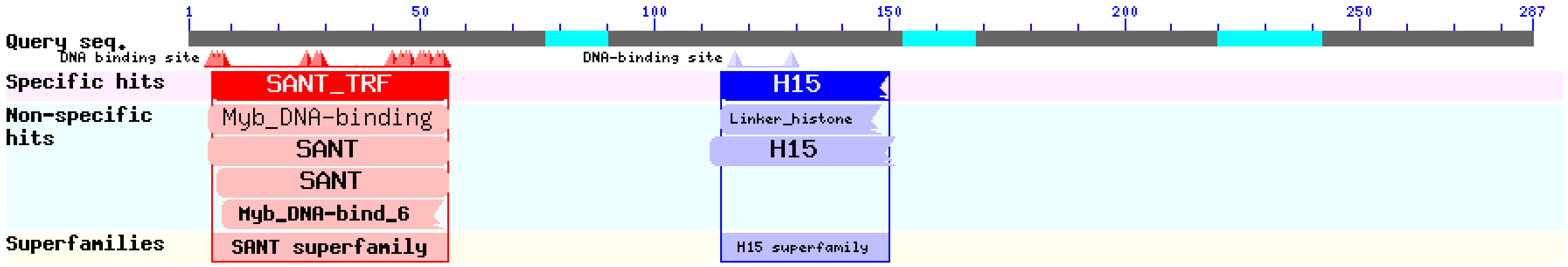

SiMYB127

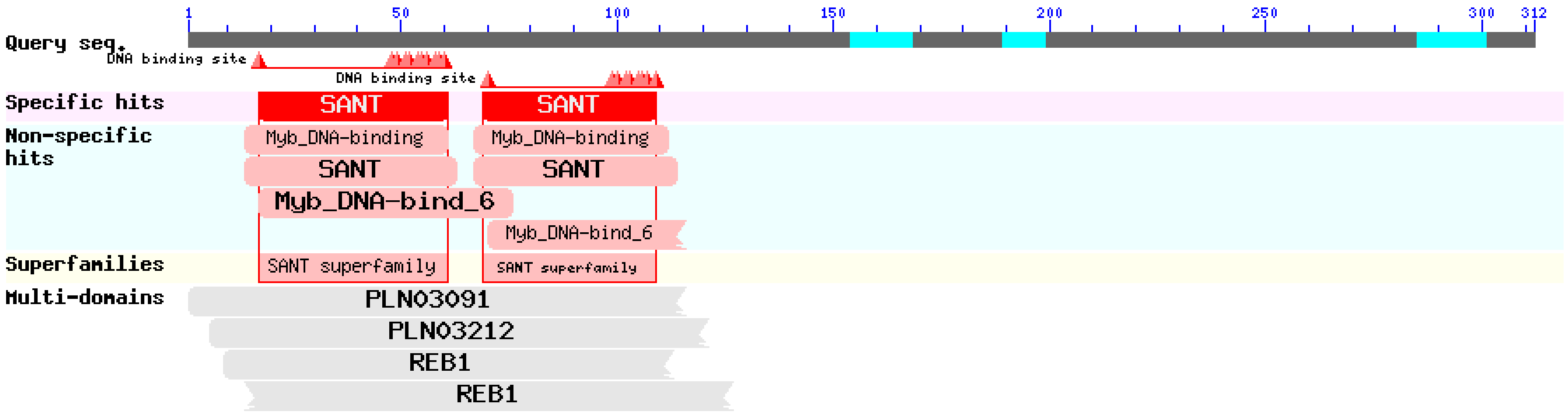

SiMYB128

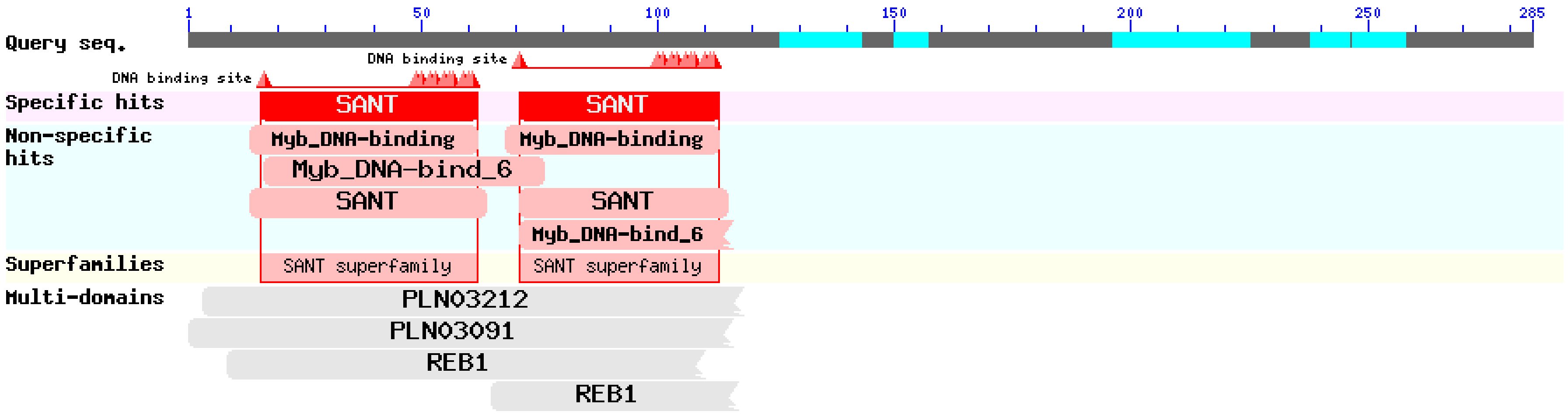

SiMYB129

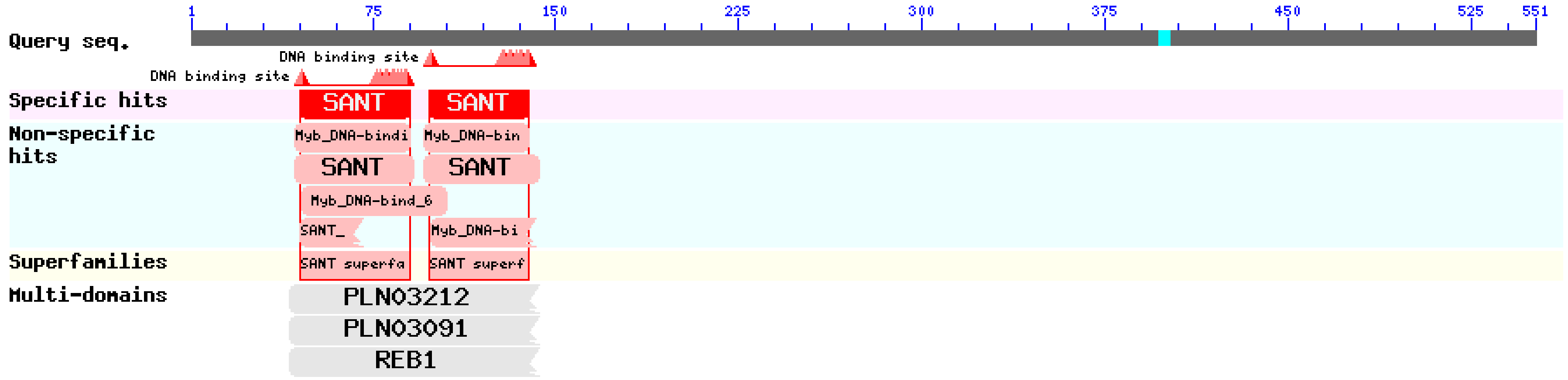

SiMYB130

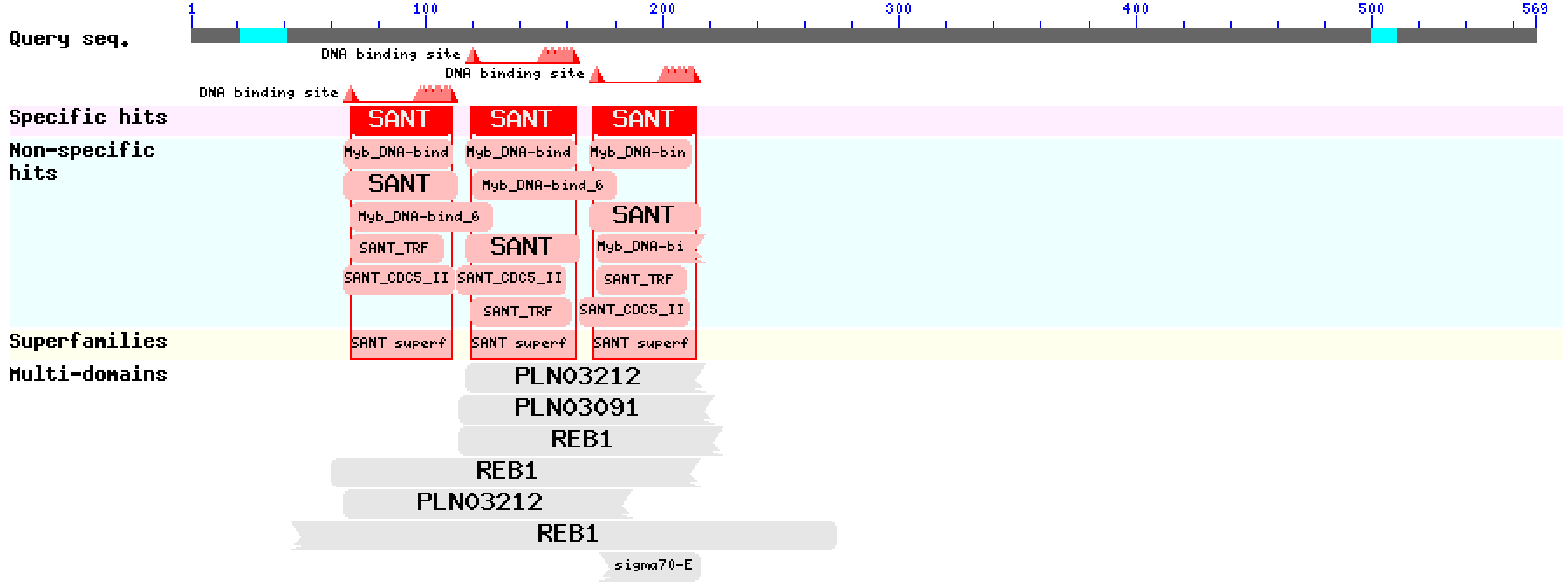

SiMYB131

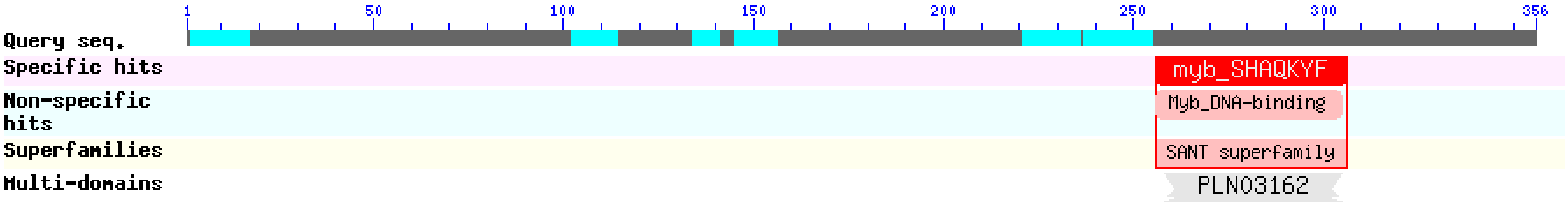

SiMYB132

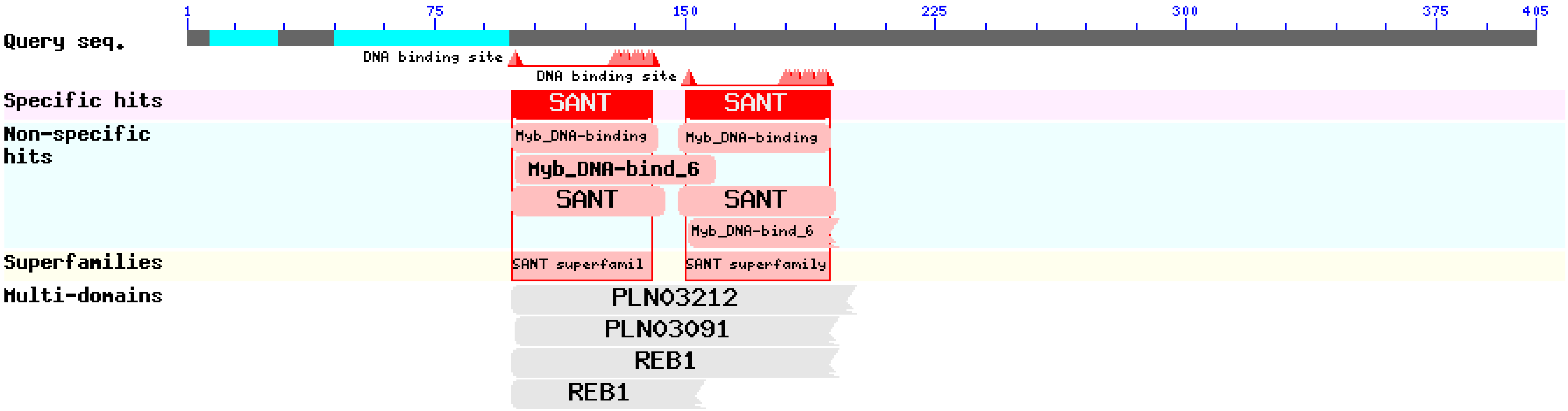

SiMYB133

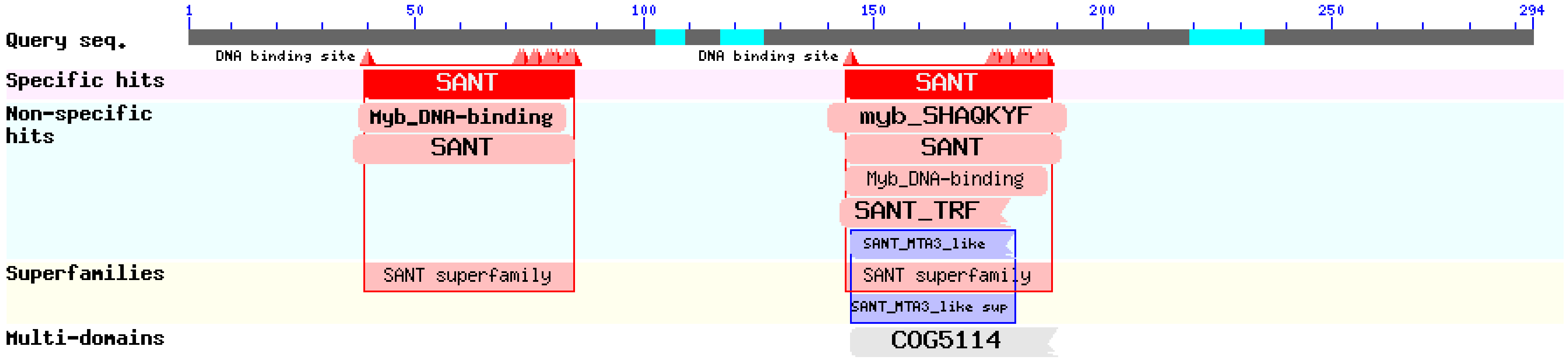

SiMYB134

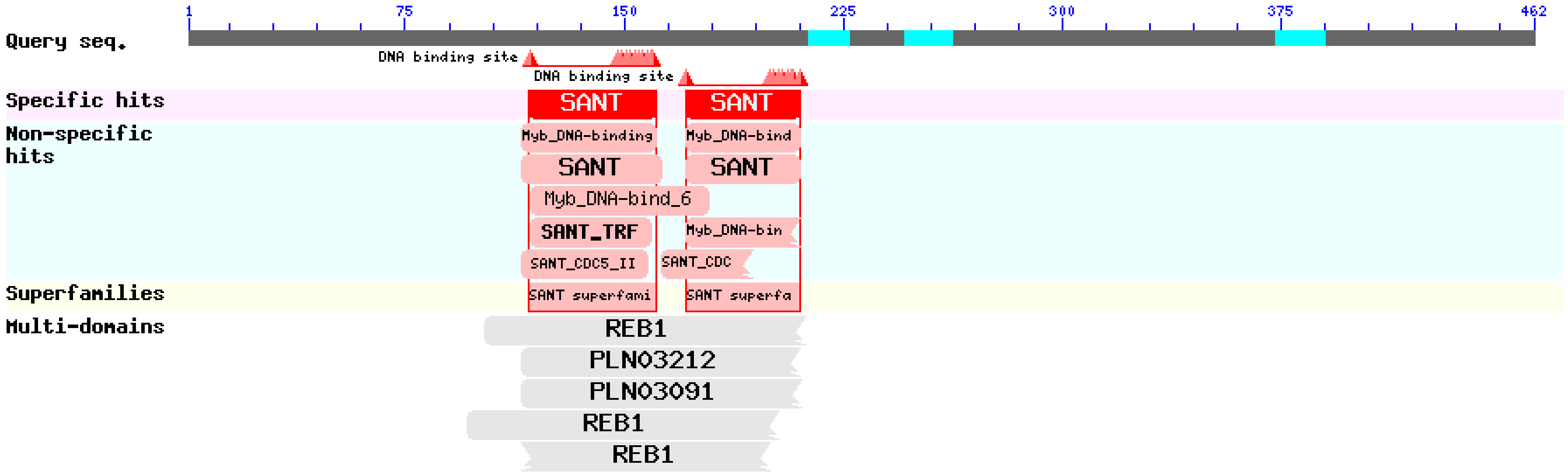

SiMYB135

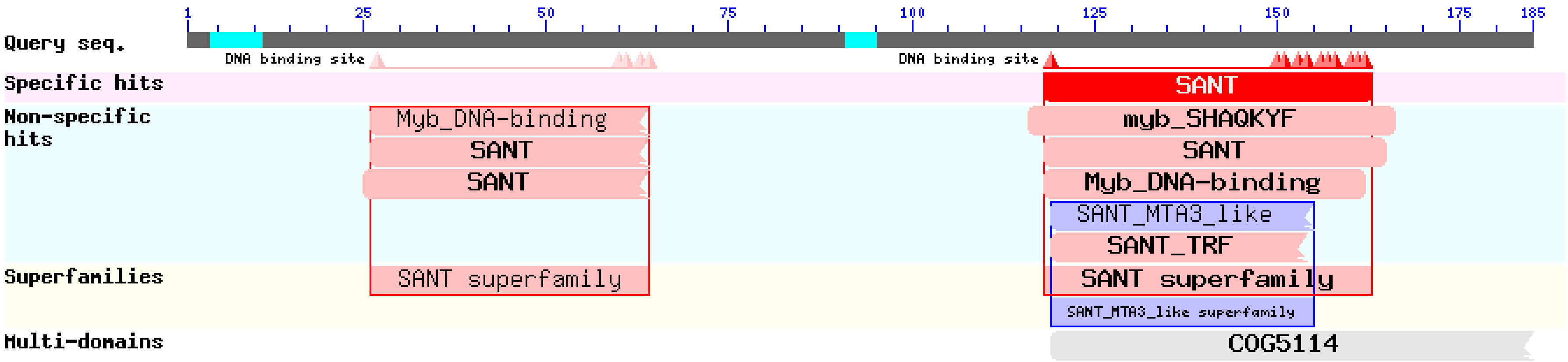

SiMYB136

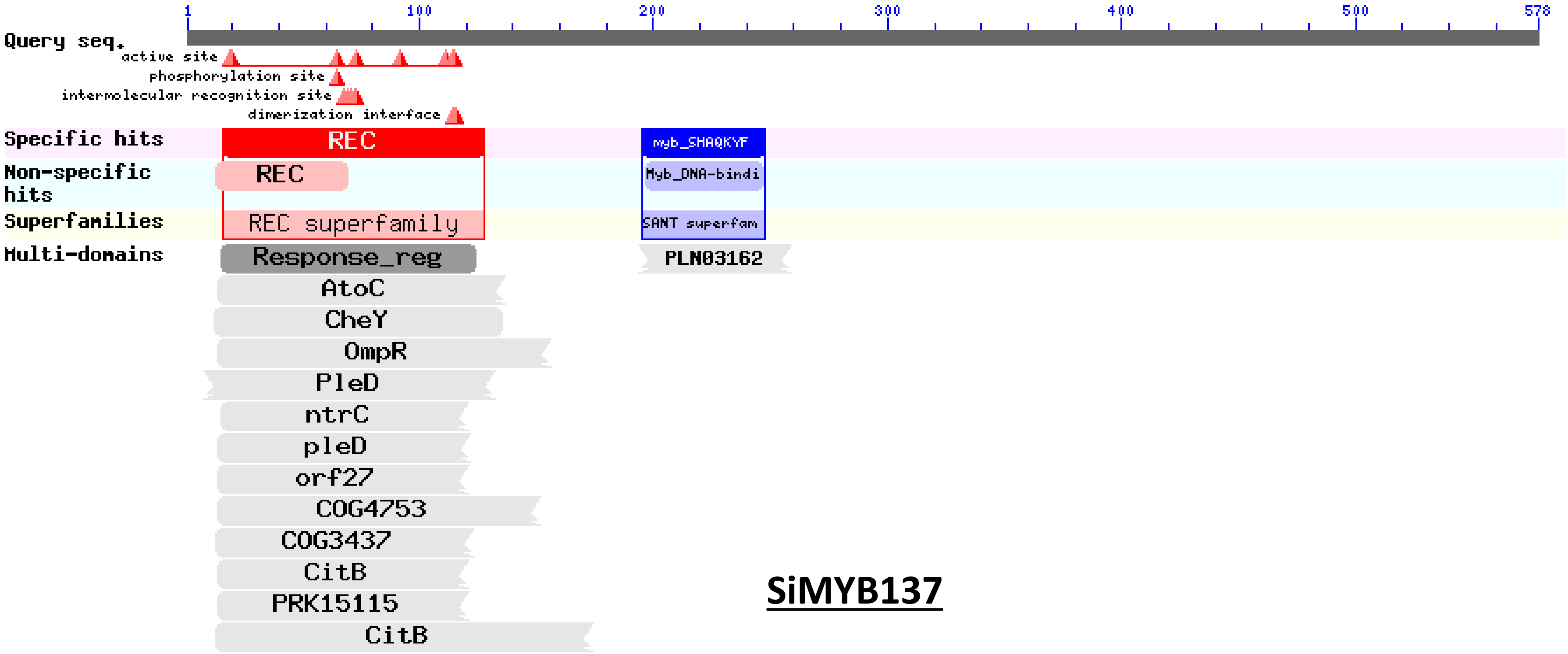

SiMYB137

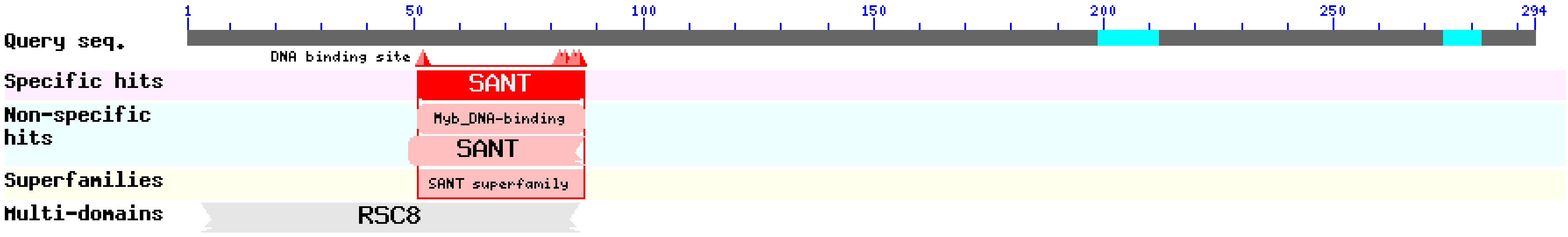

SiMYB138

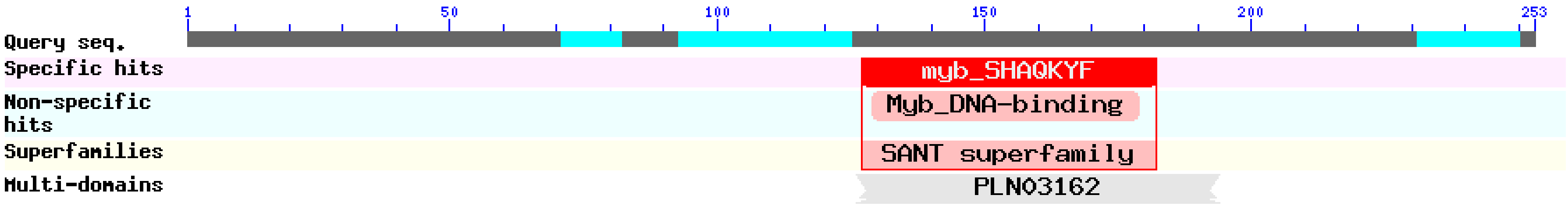

SiMYB139

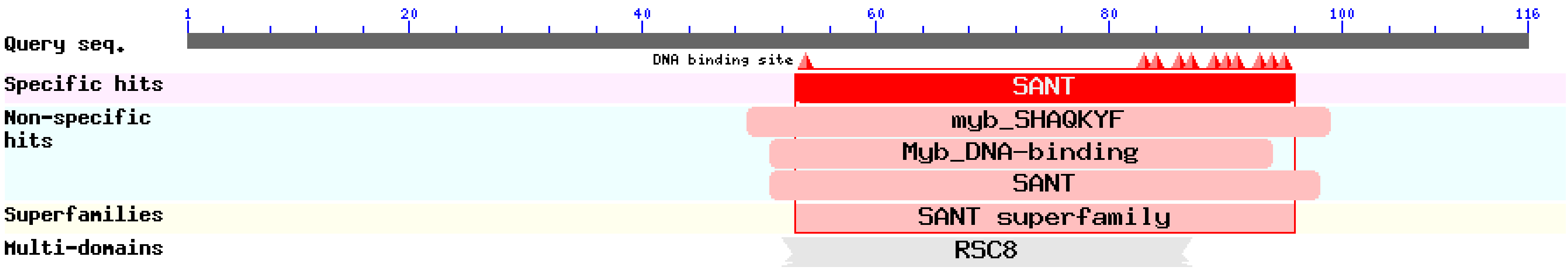

SiMYB140

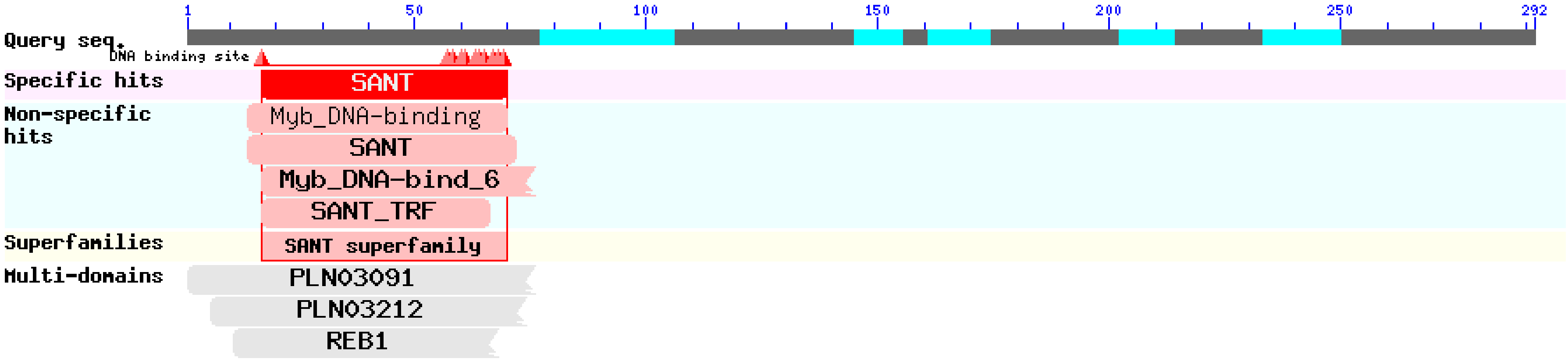

SiMYB141

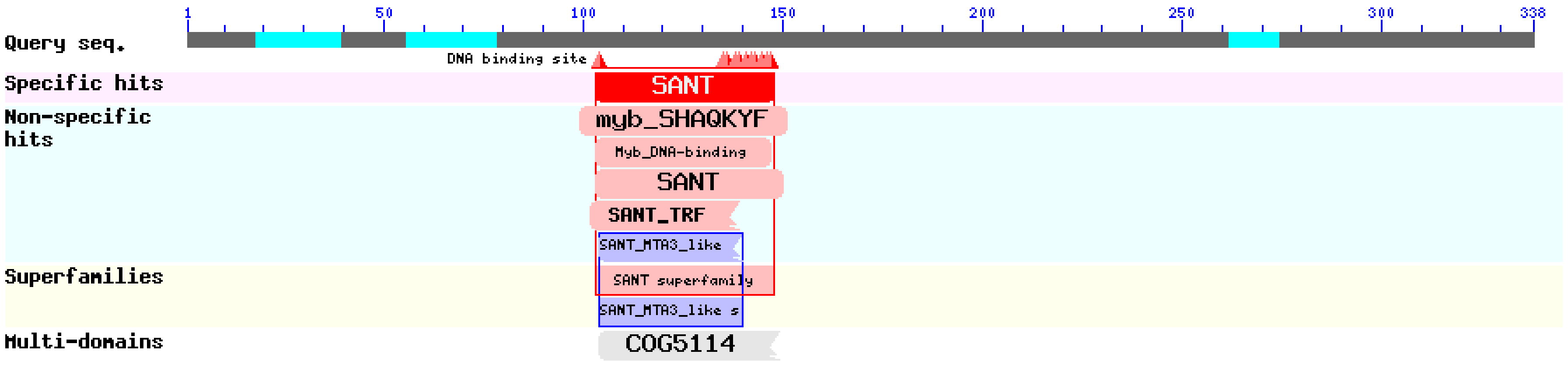

SiMYB142

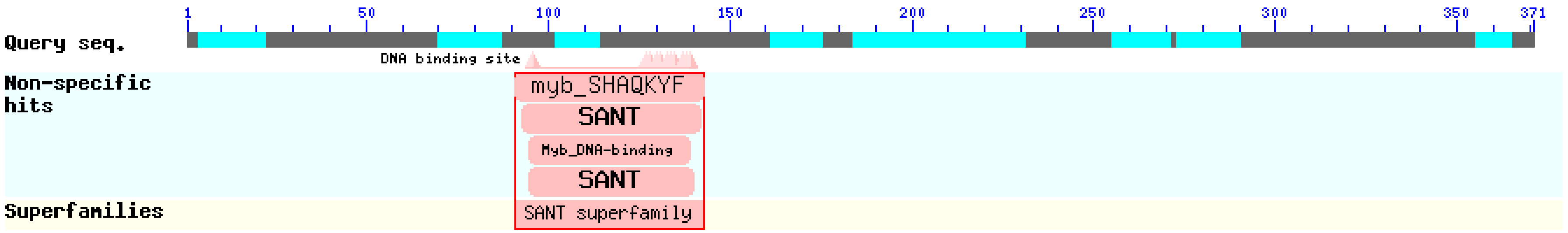

SiMYB143

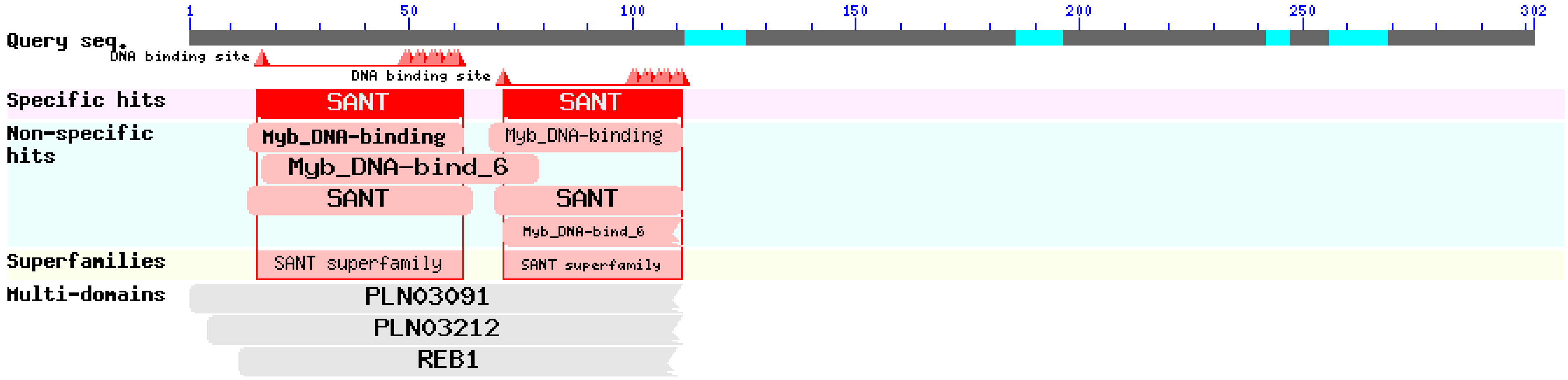

SiMYB144

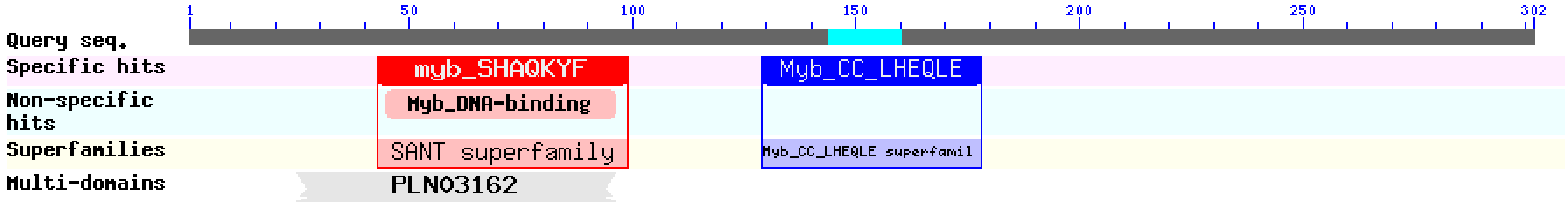

SiMYB145

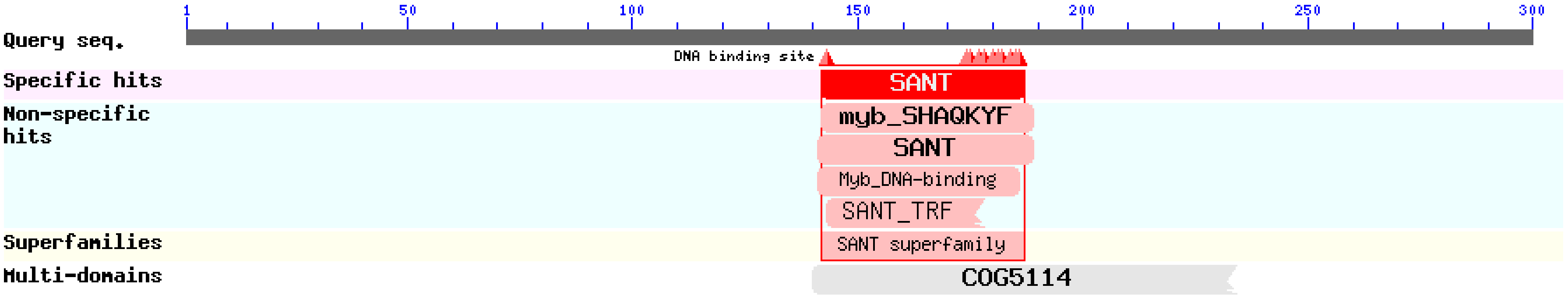

SiMYB146

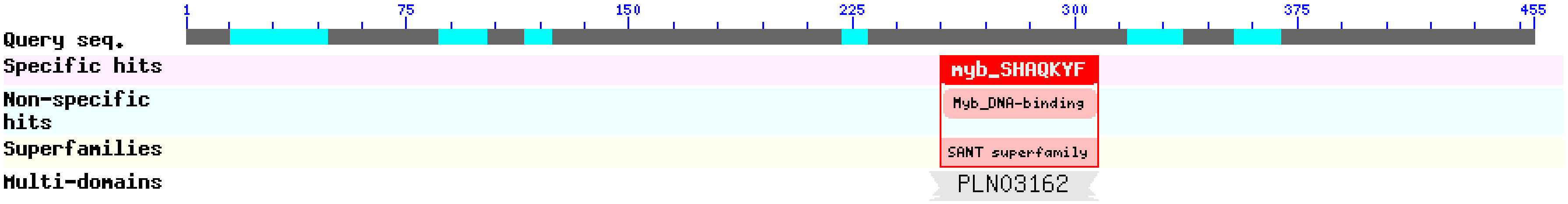

SiMYB147

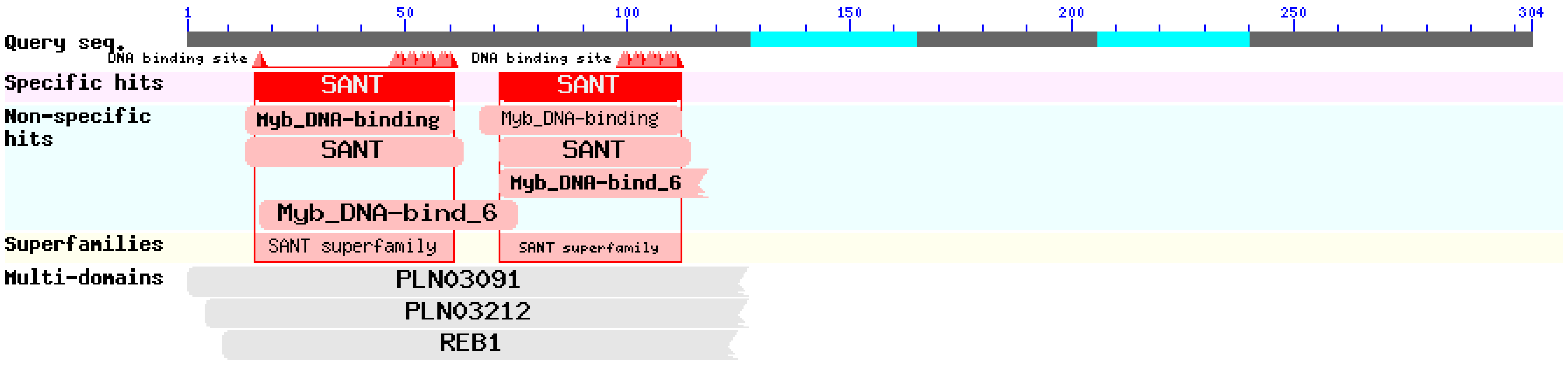

SiMYB148

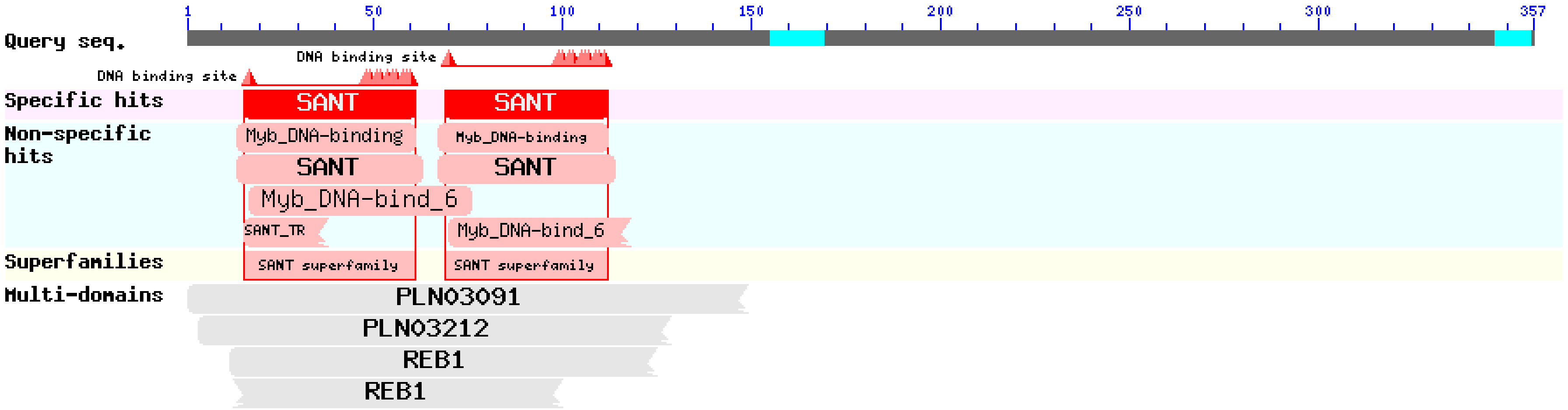

SiMYB149

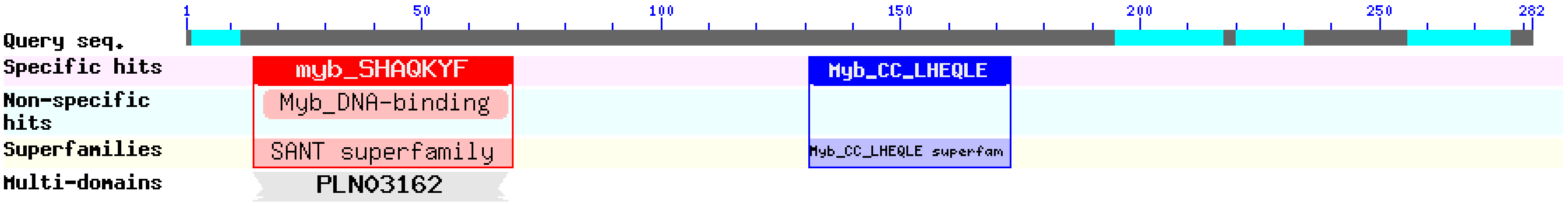

SiMYB150

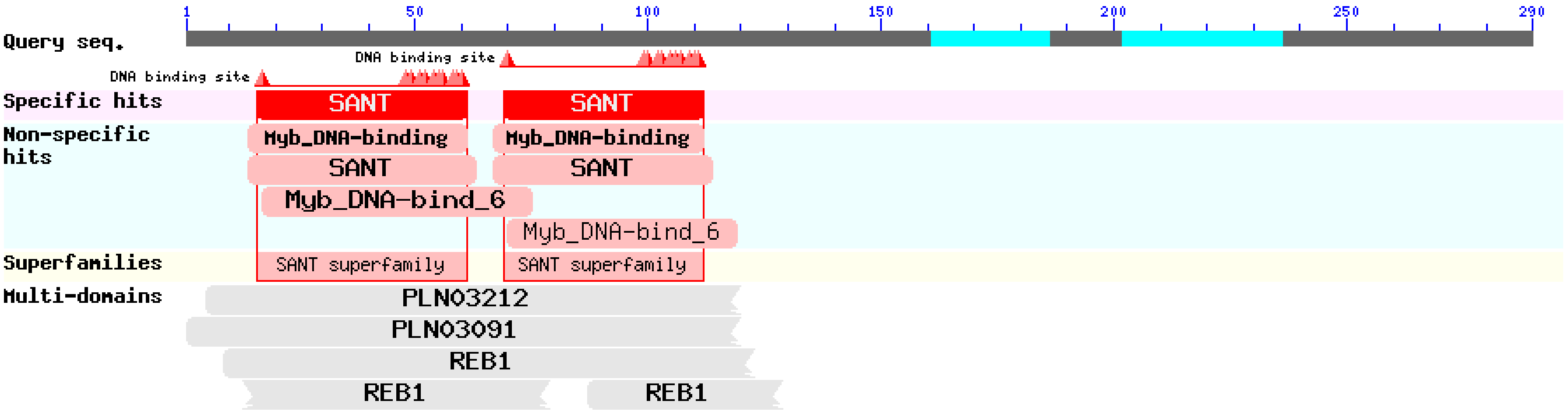

SiMYB151

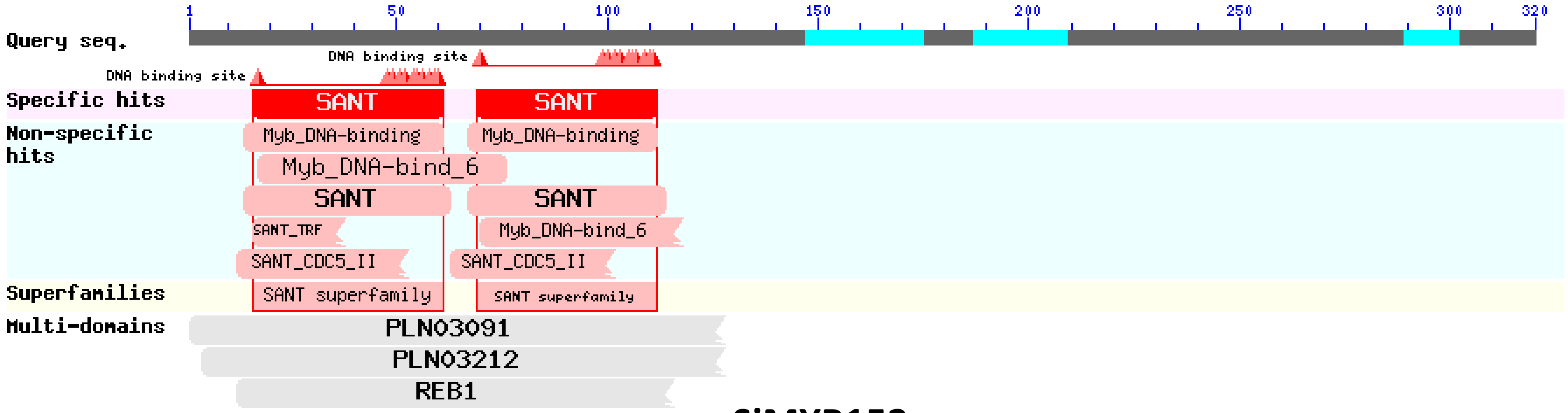

SiMYB152

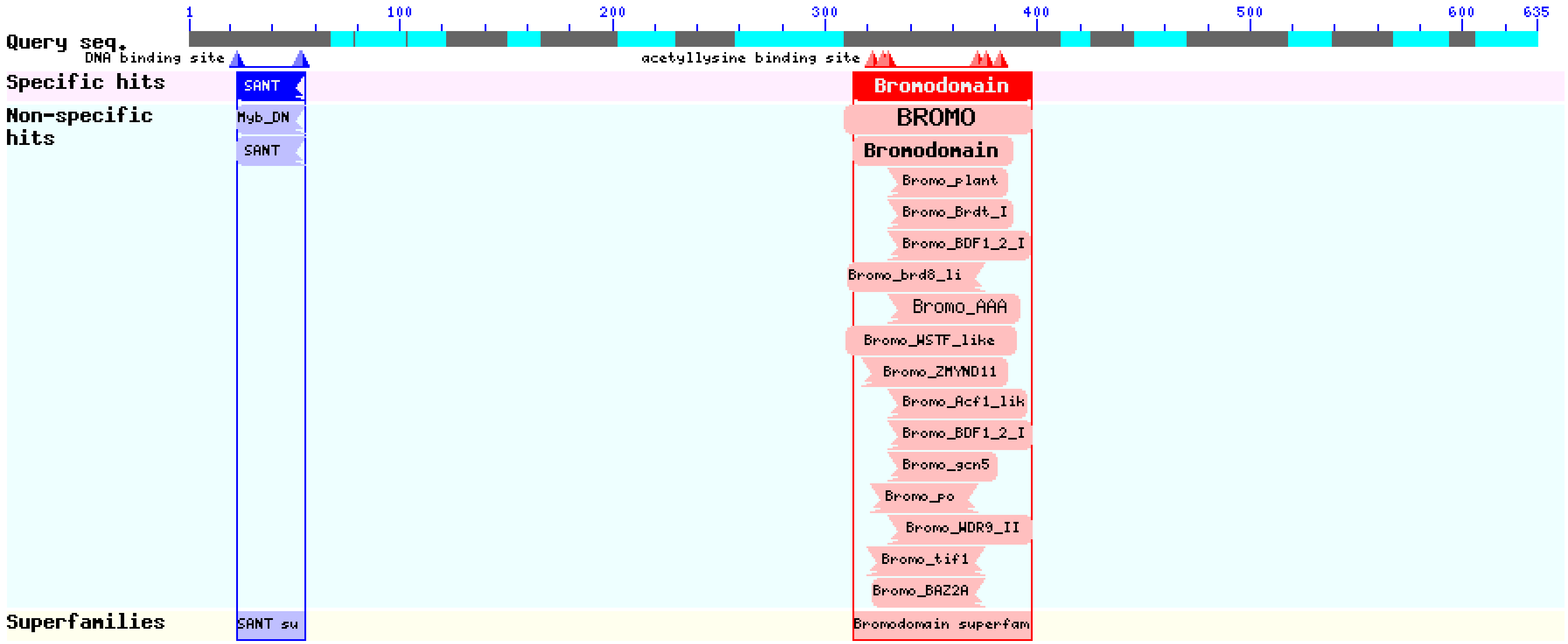

SiMYB153

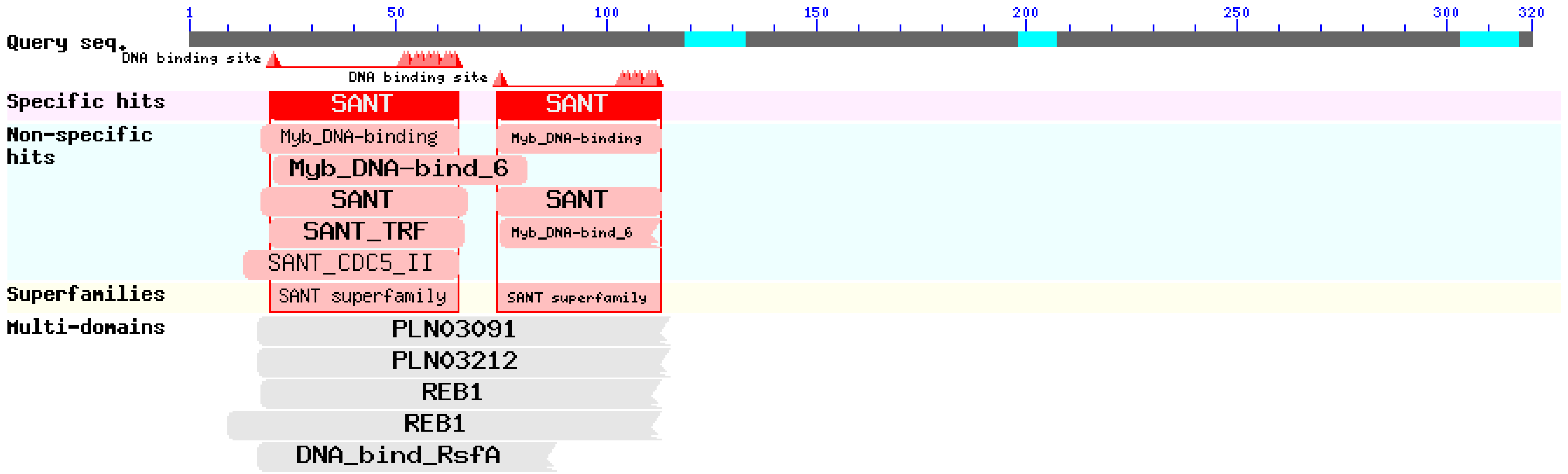

SiMYB154

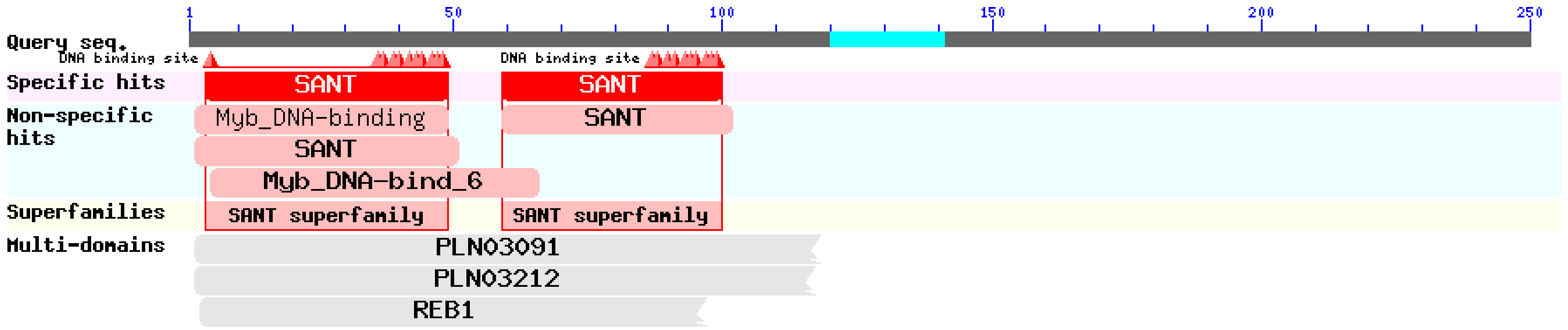

SiMYB155

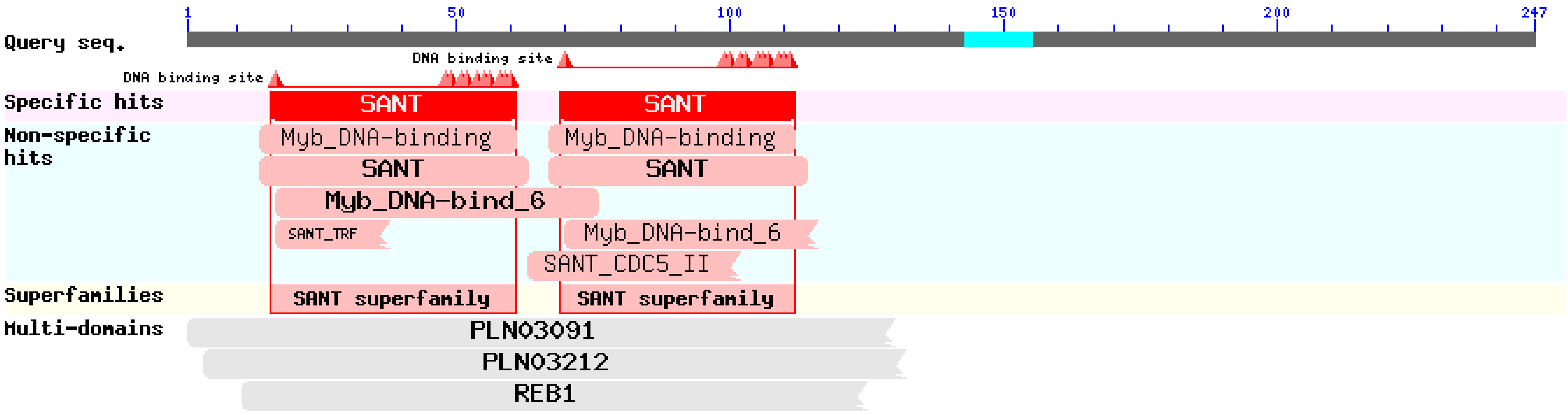

SiMYB156

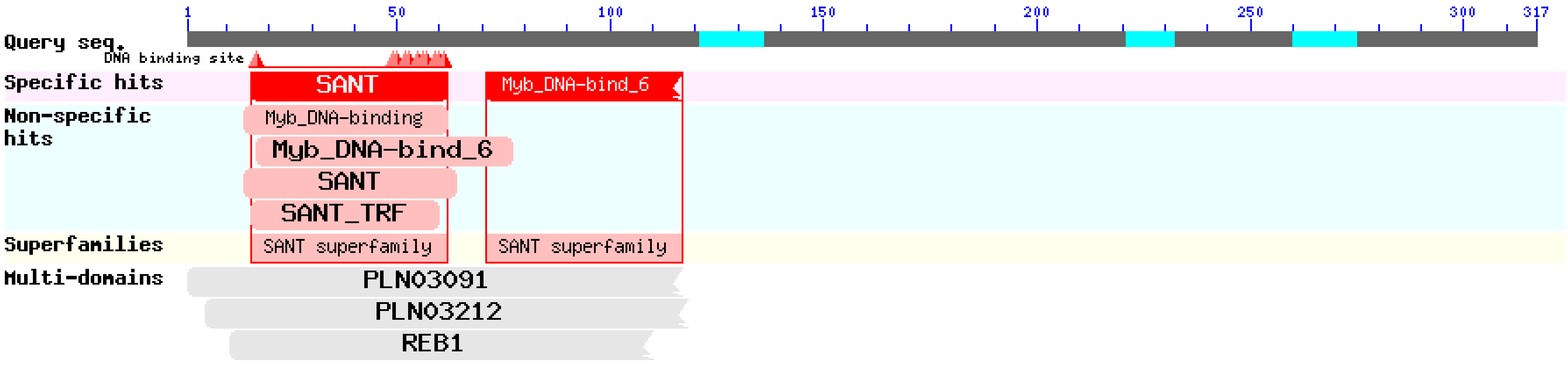

SiMYB157

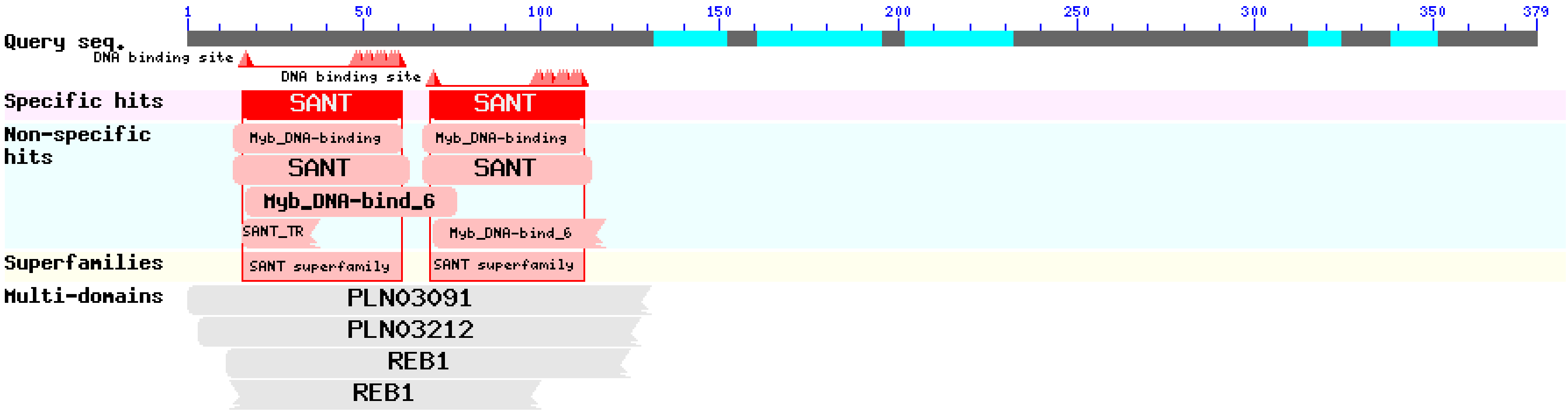

SiMYB158

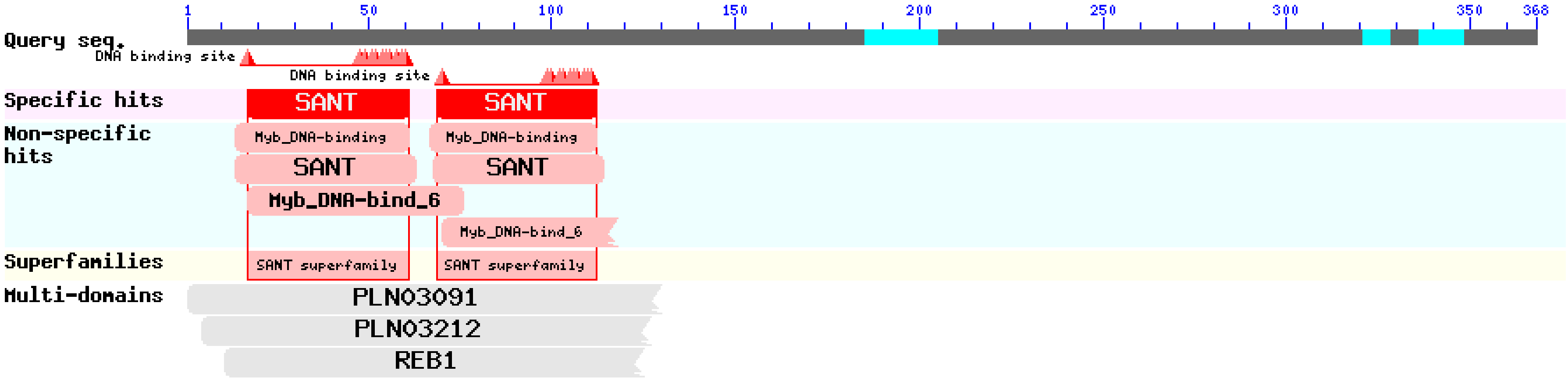

SiMYB159

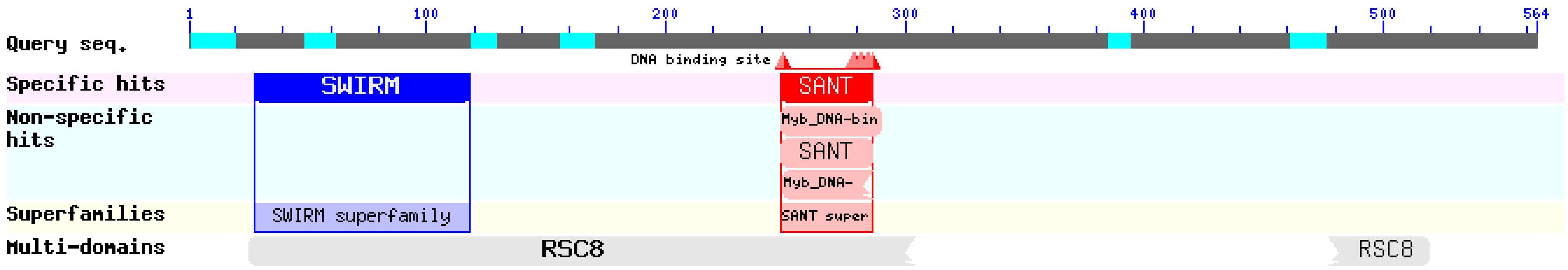

SiMYB160

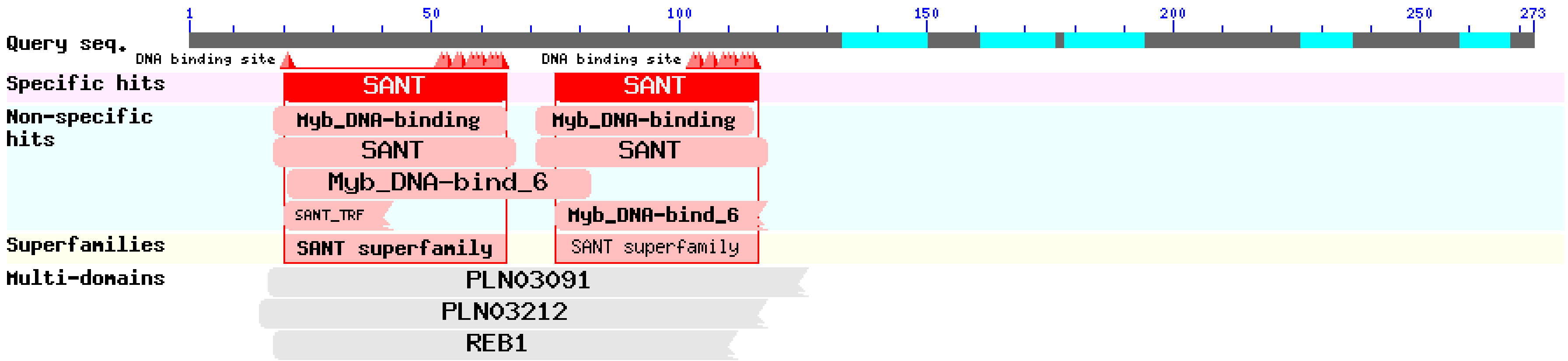

SiMYB161

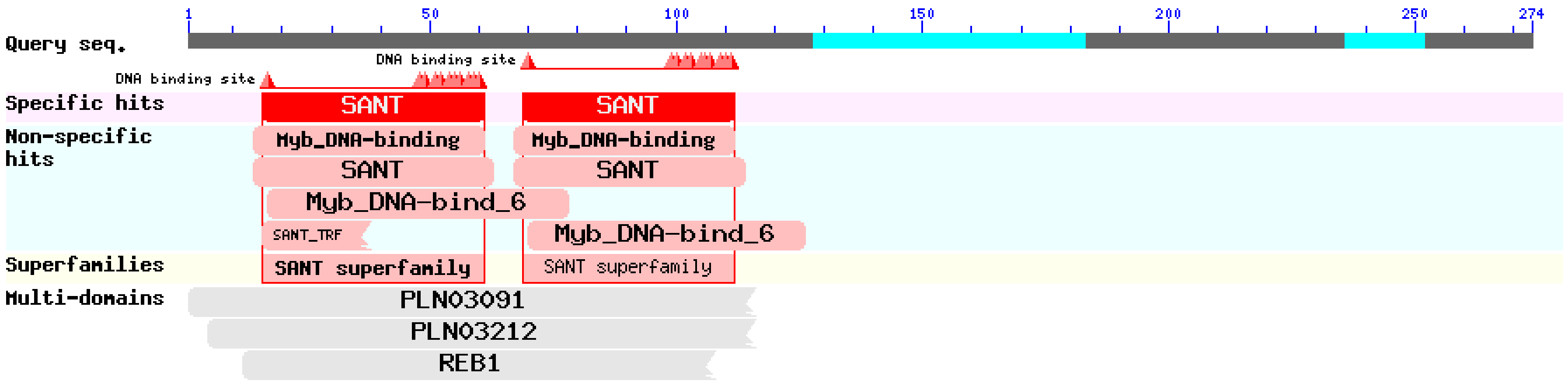

SiMYB162

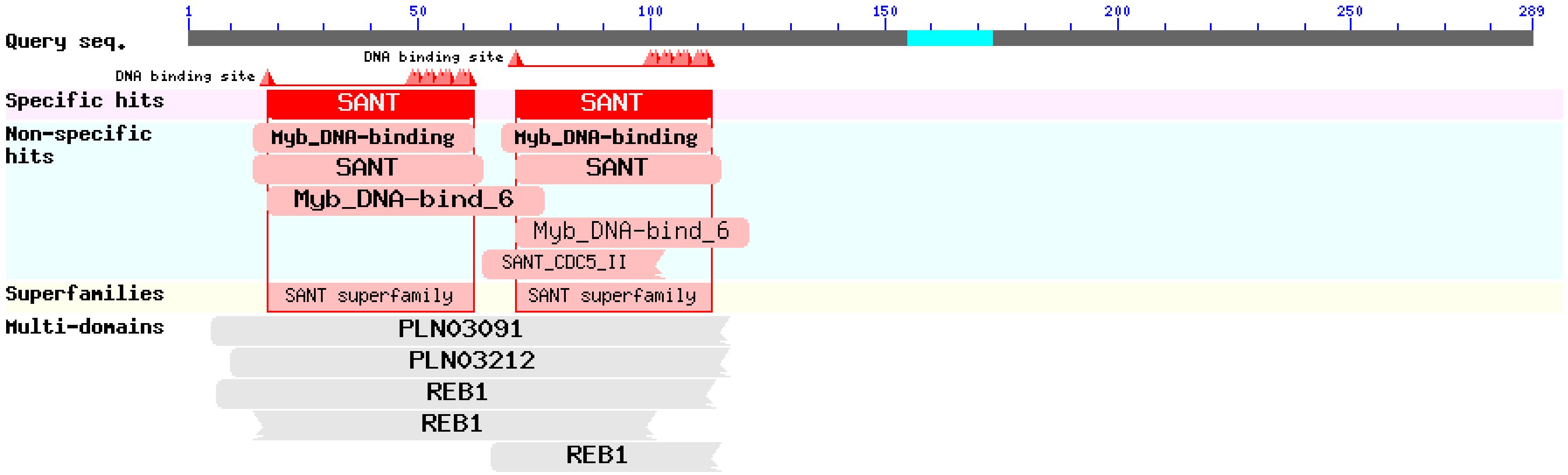

SiMYB163

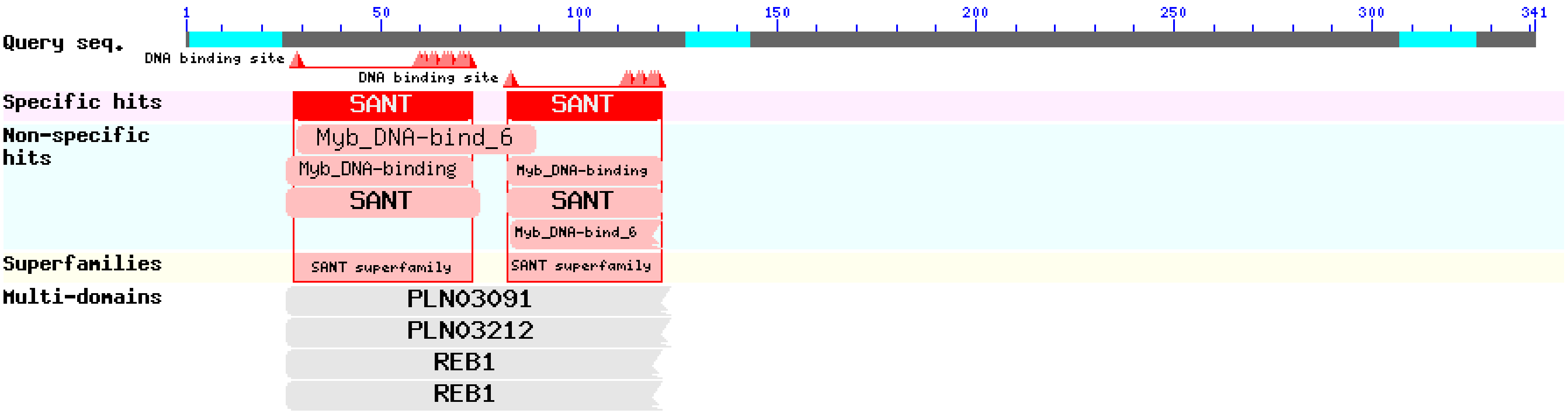

SiMYB164

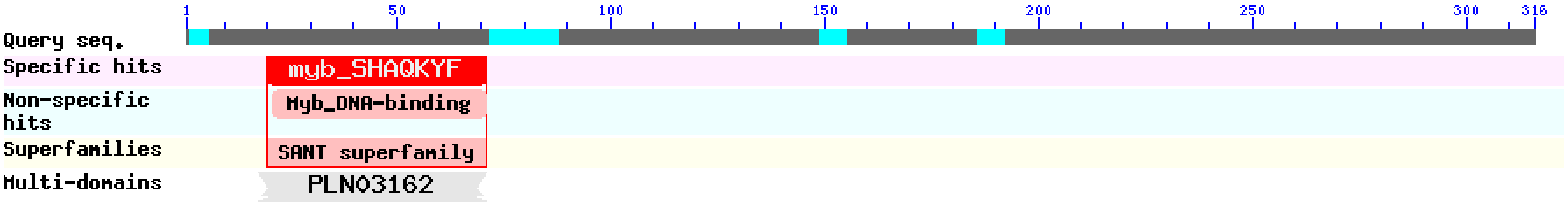

SiMYB165

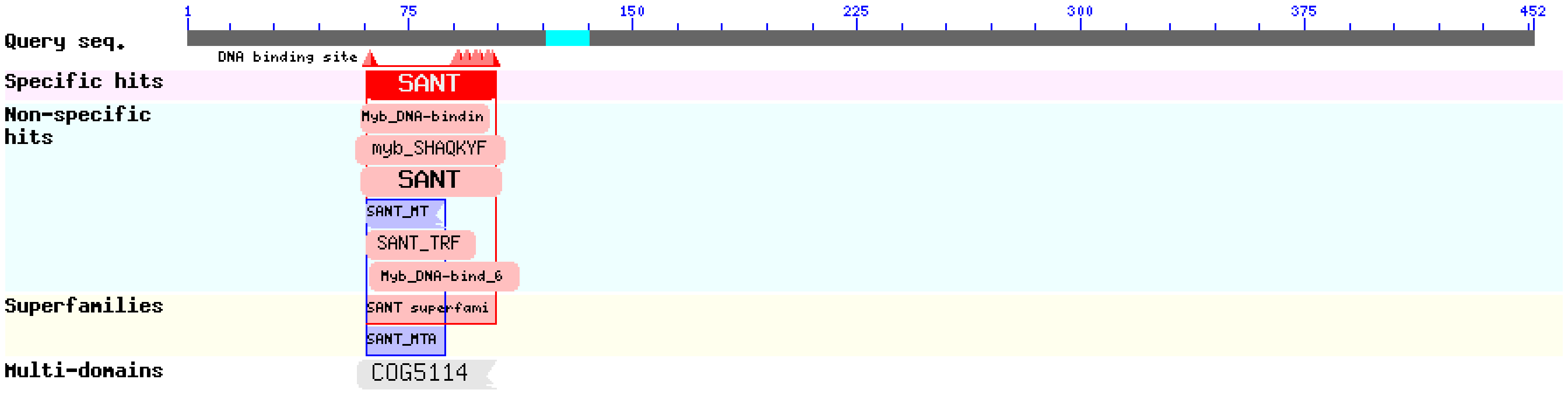

SiMYB166

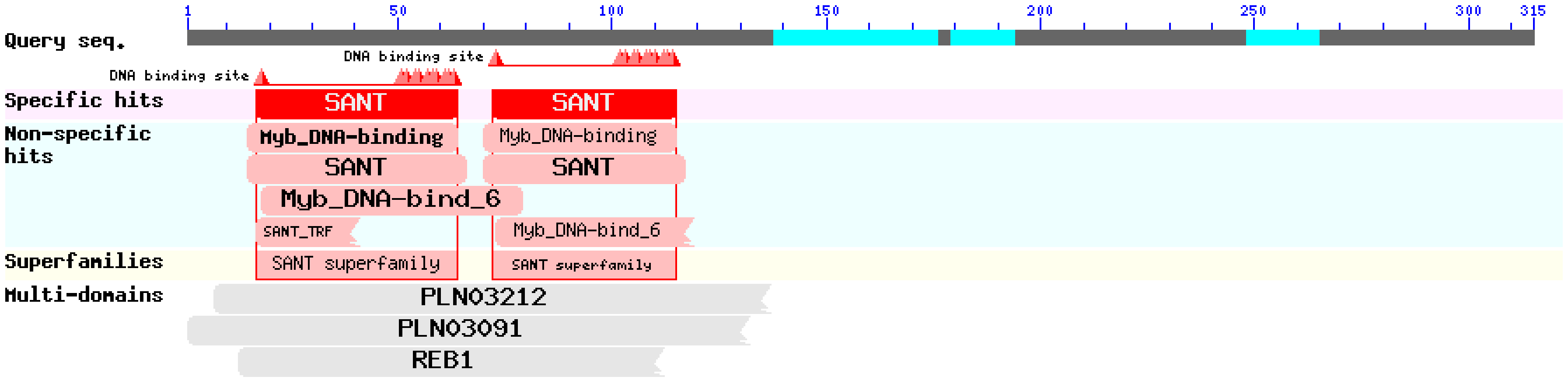

SiMYB167

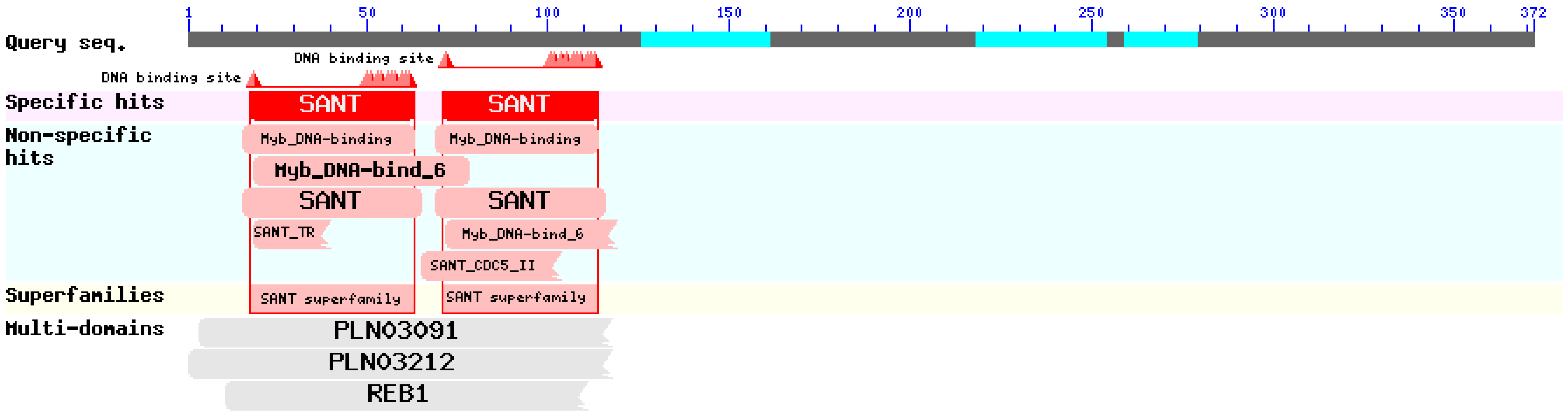

SiMYB168

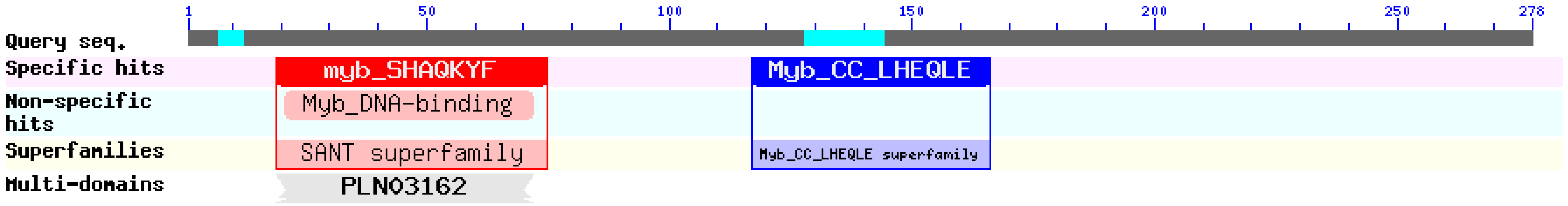

SiMYB169

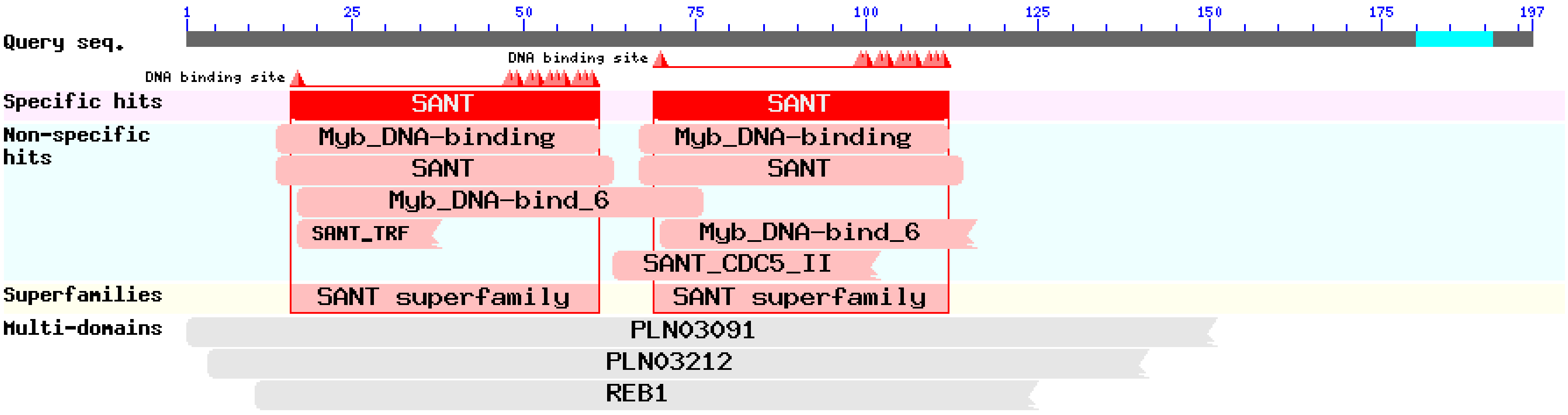

SiMYB170

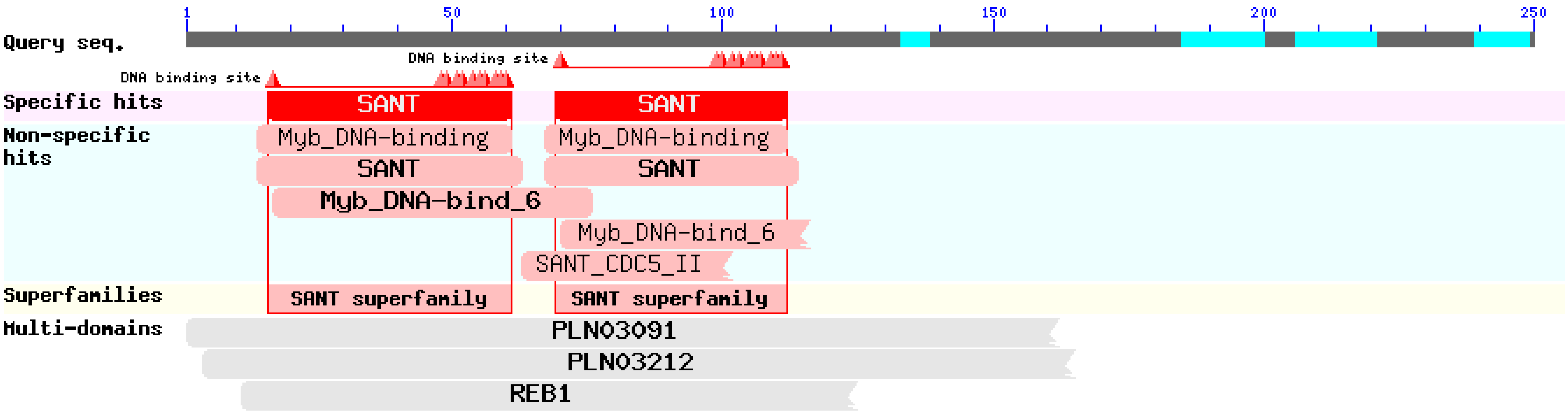

SiMYB171

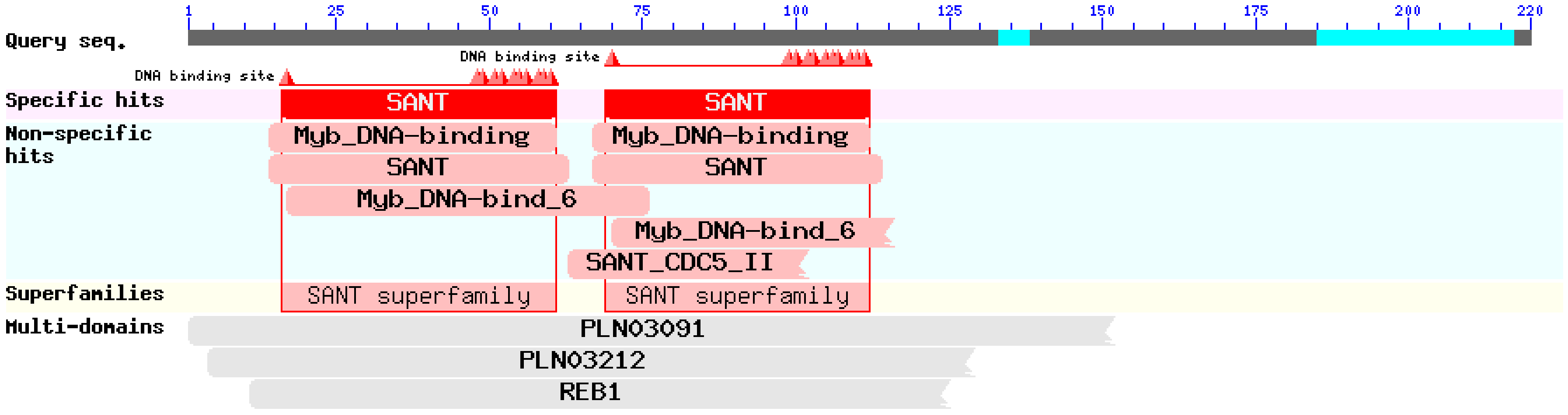

SiMYB172

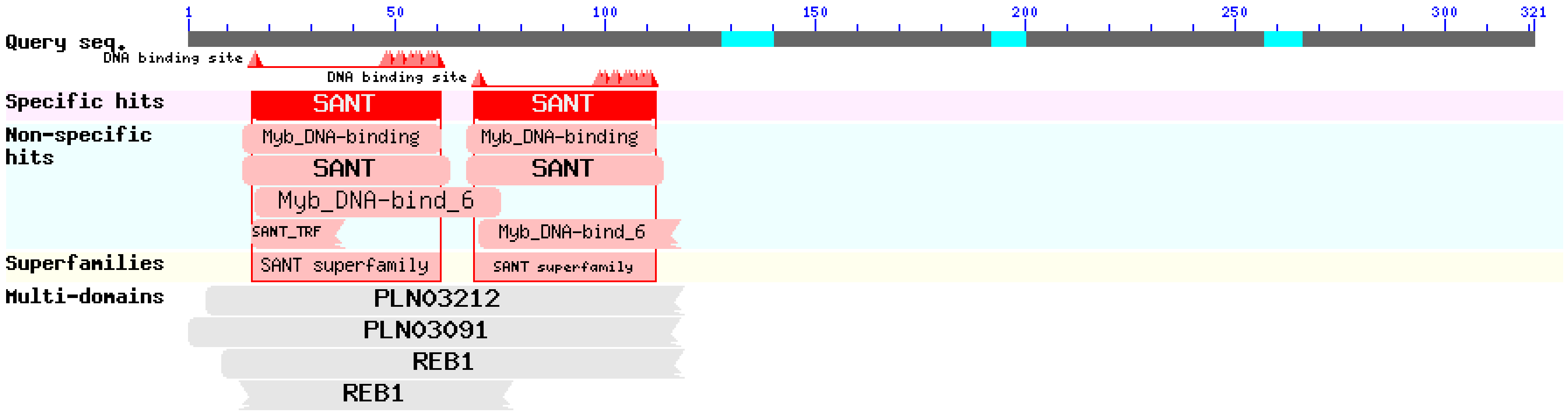

SiMYB173

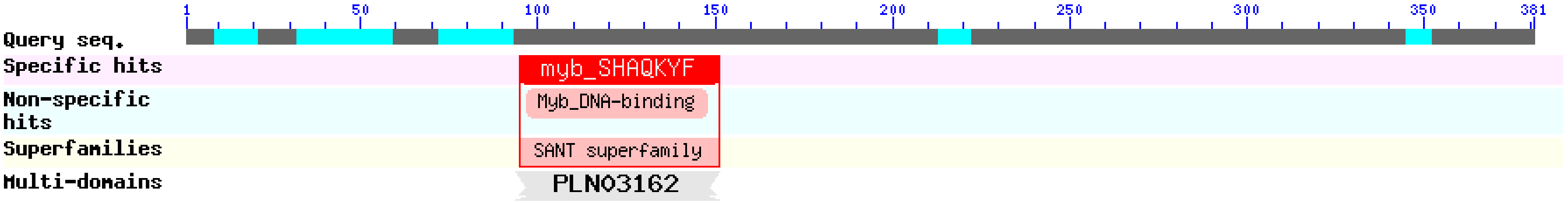

SiMYB174

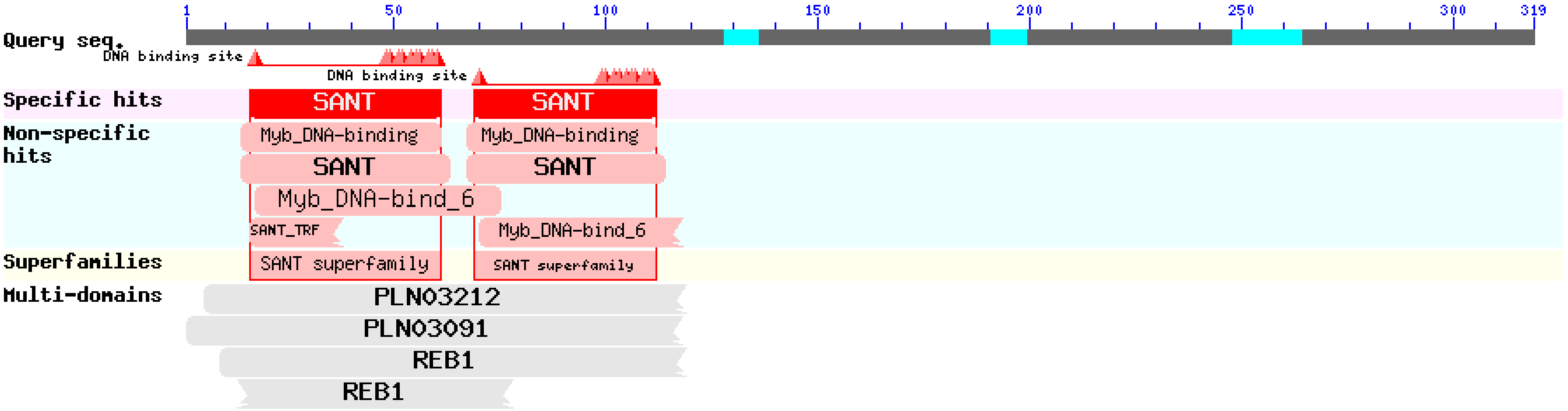

SiMYB175

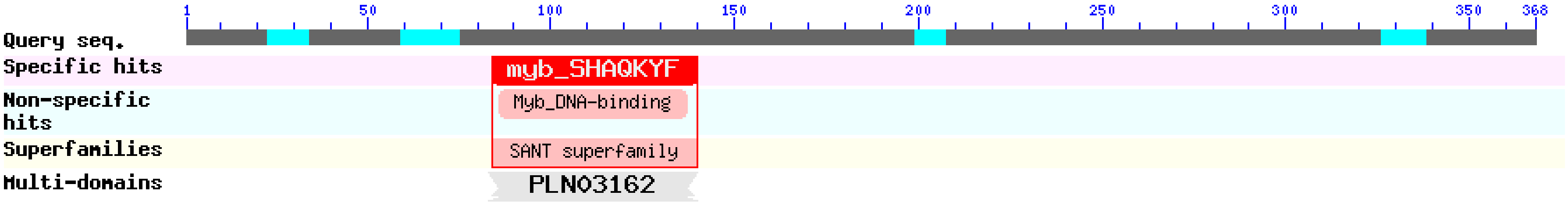

SiMYB176

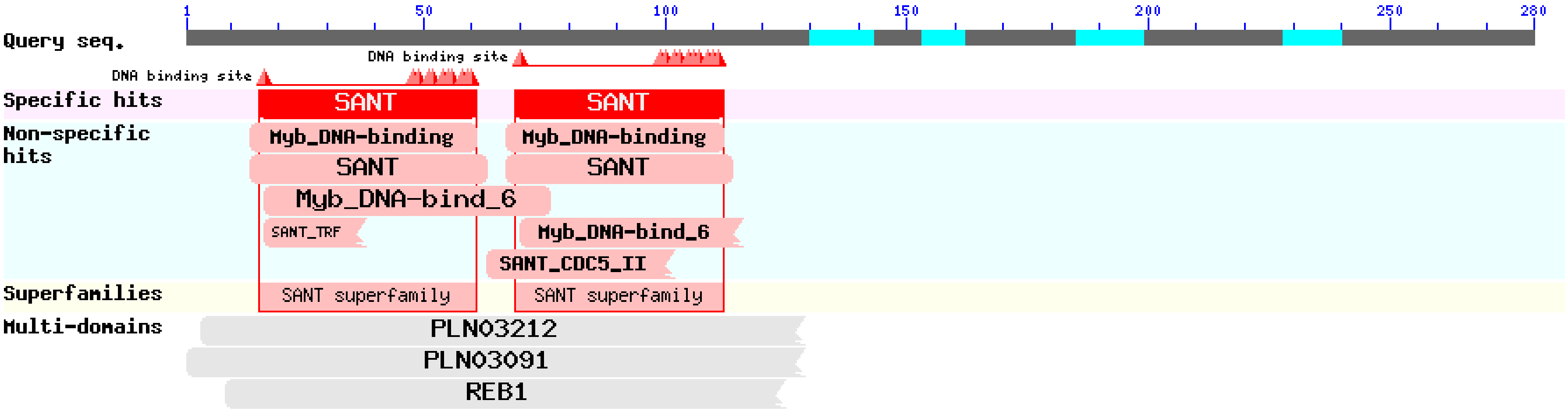

SiMYB177

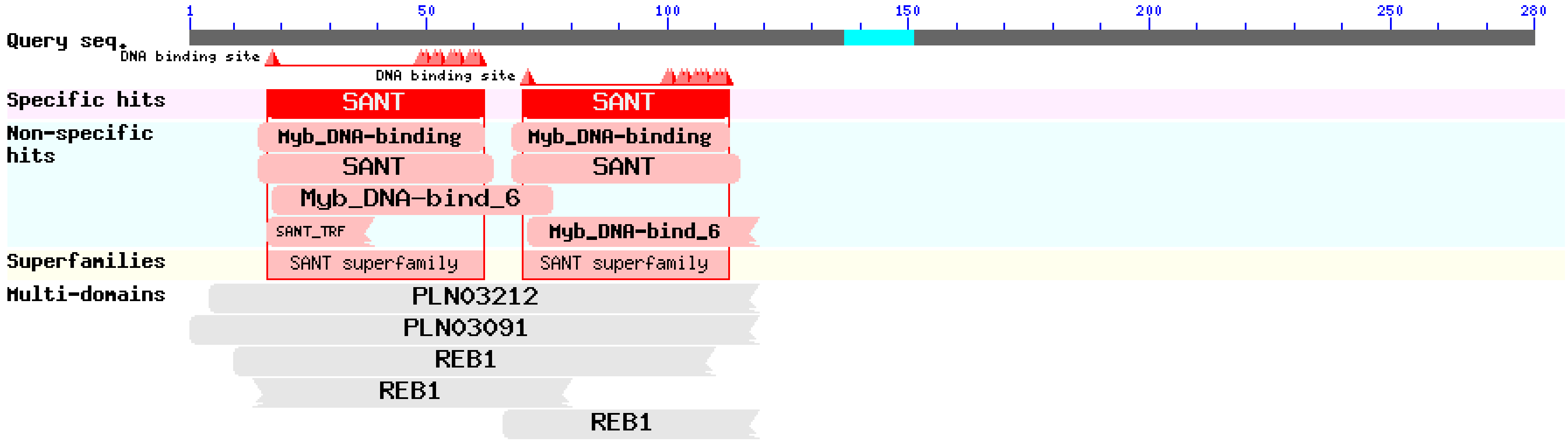

SiMYB178

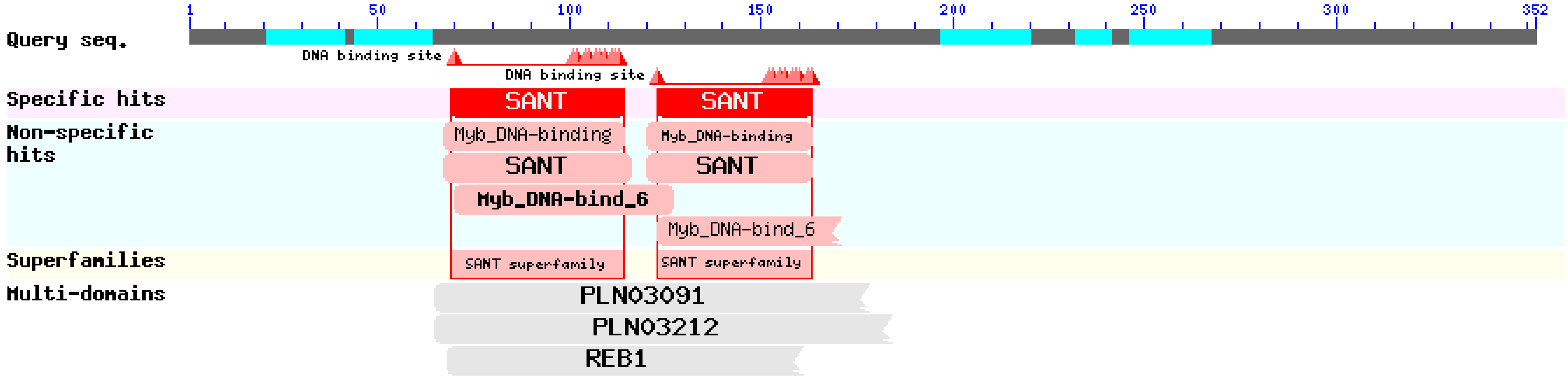

SiMYB179

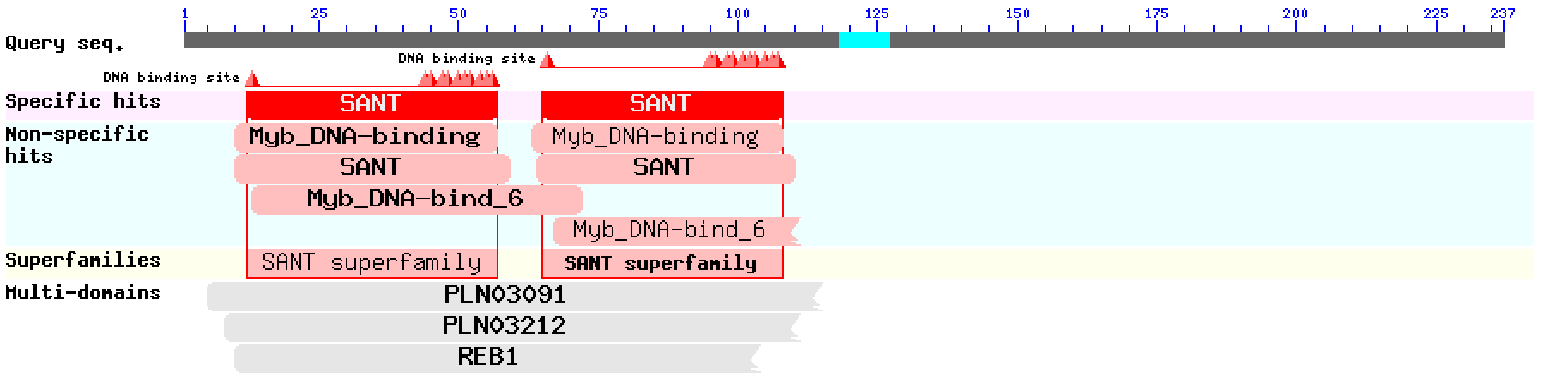

SiMYB180

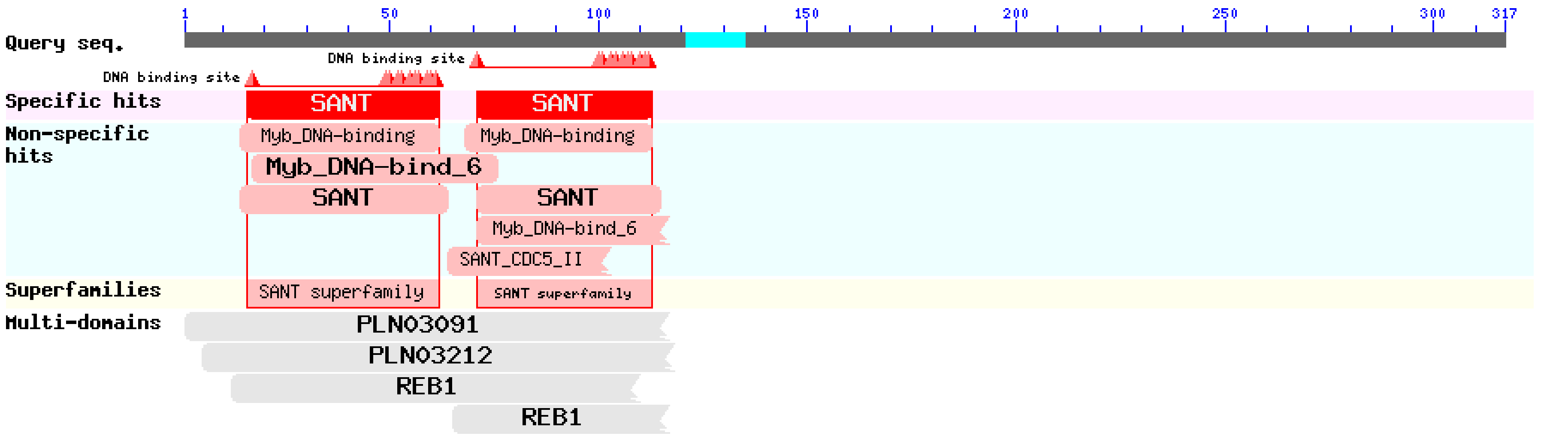

SiMYB181

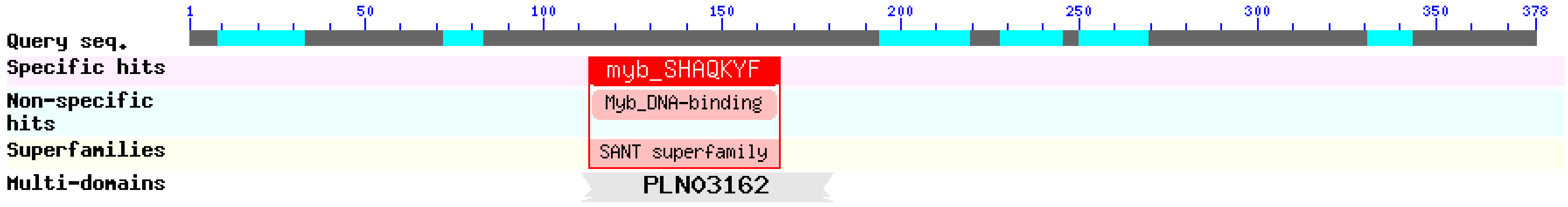

SiMYB182

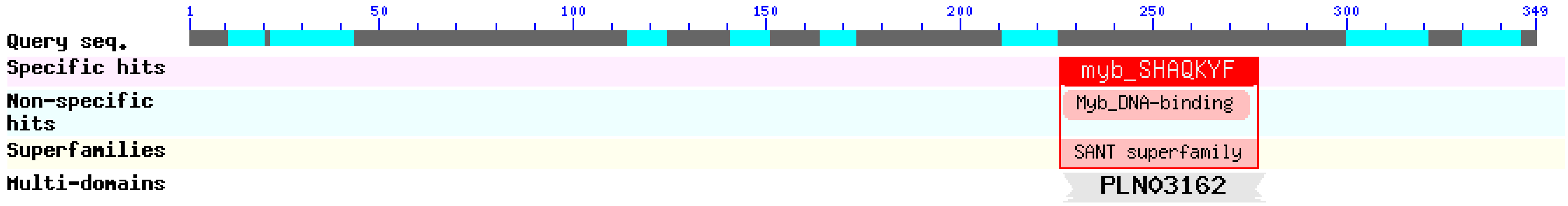

SiMYB183

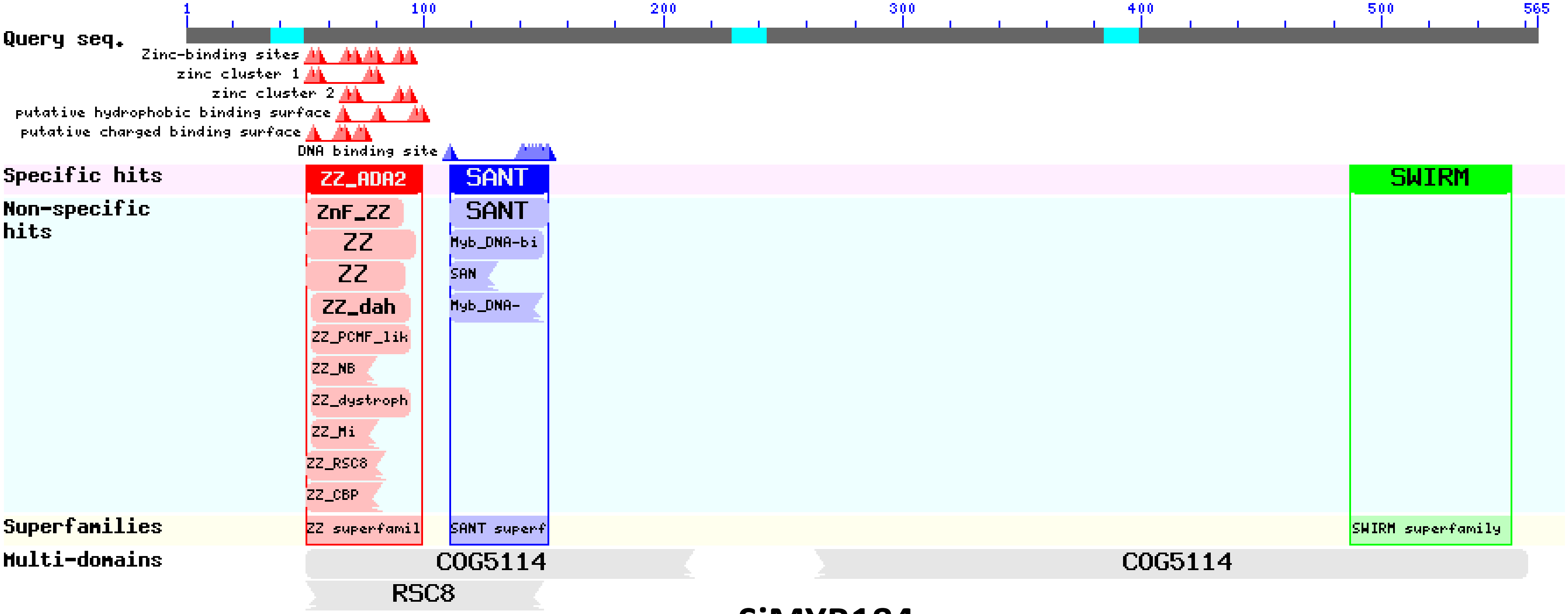

SiMYB184

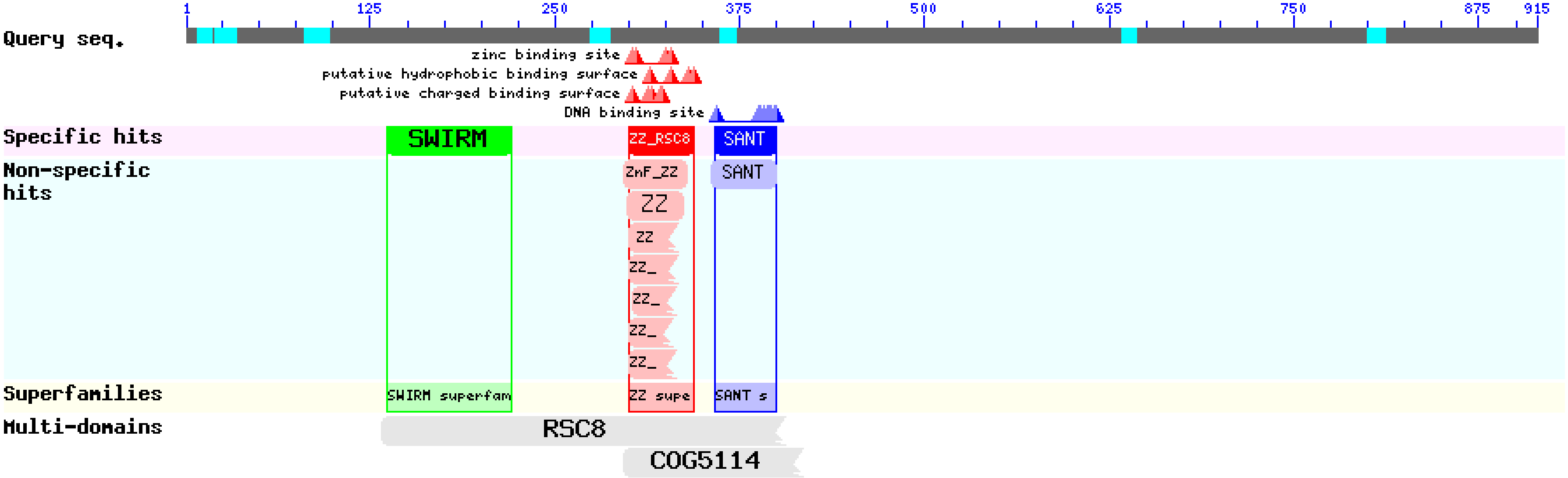

SiMYB185

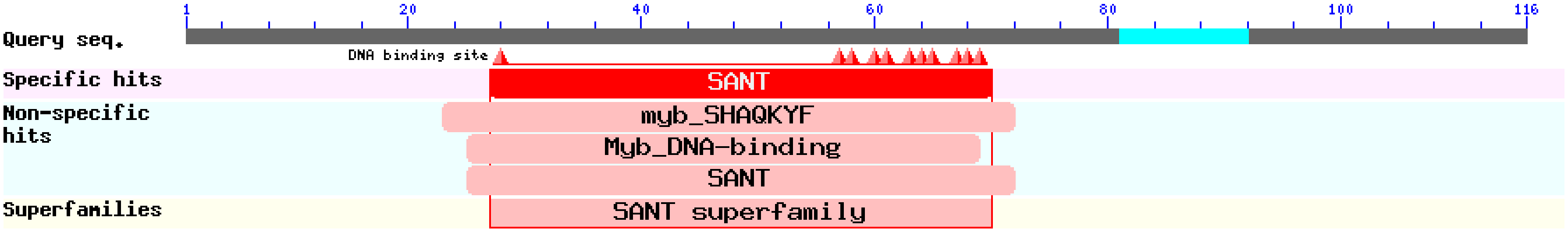

SiMYB186

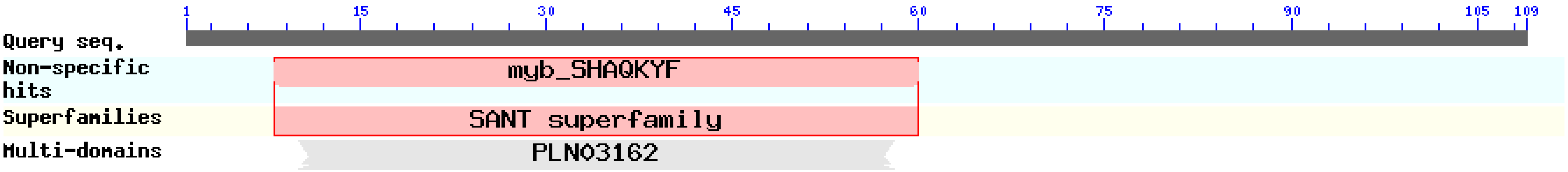

SiMYB187

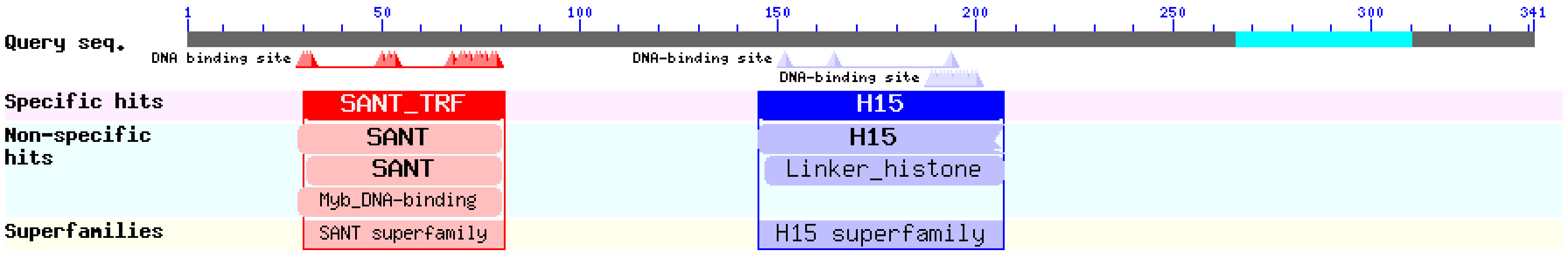

SiMYB188

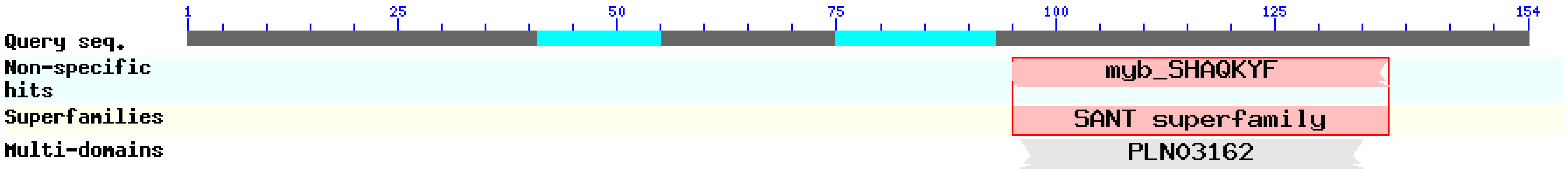

SiMYB189

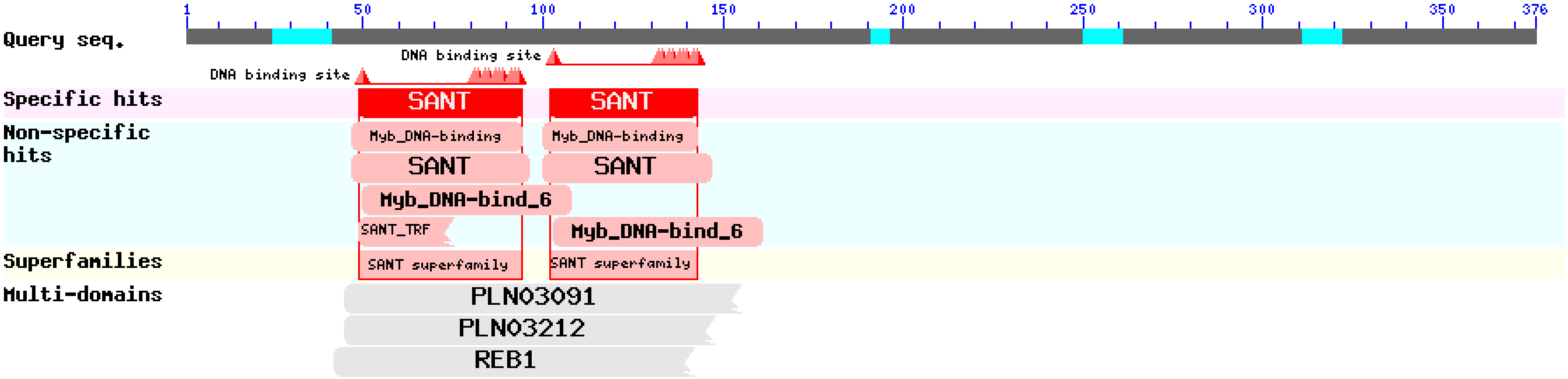

SiMYB190

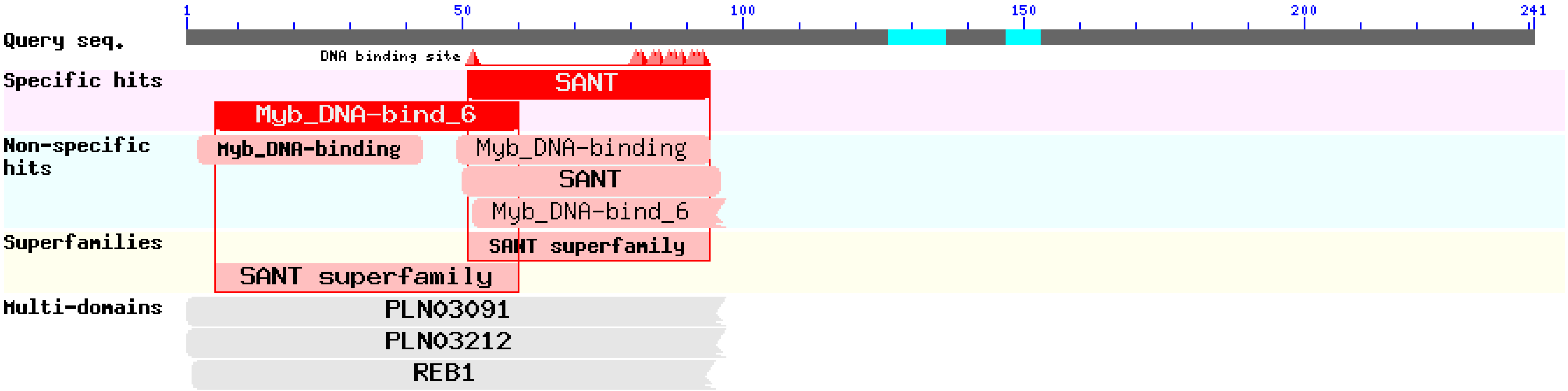

SiMYB191

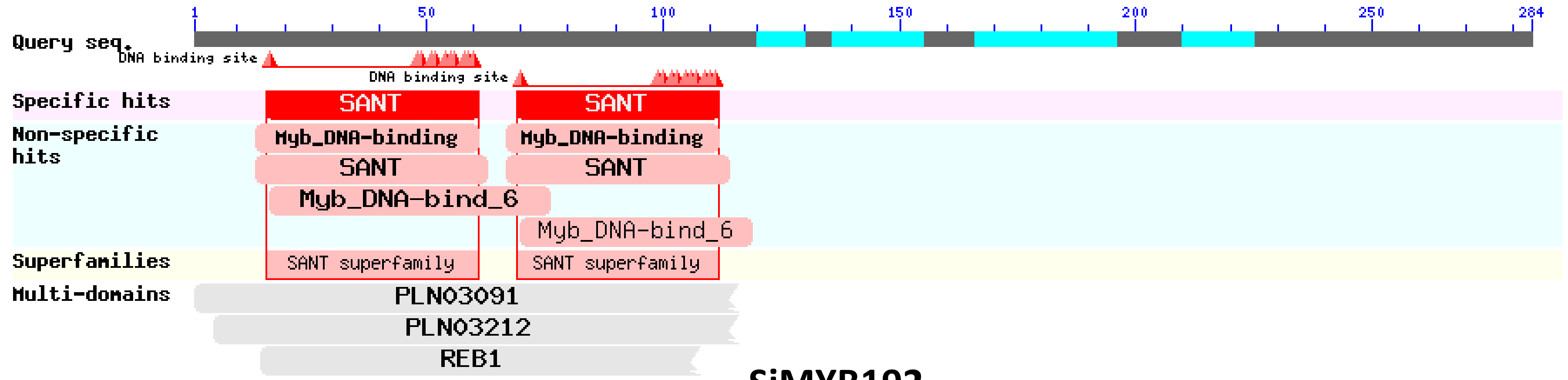

SiMYB192

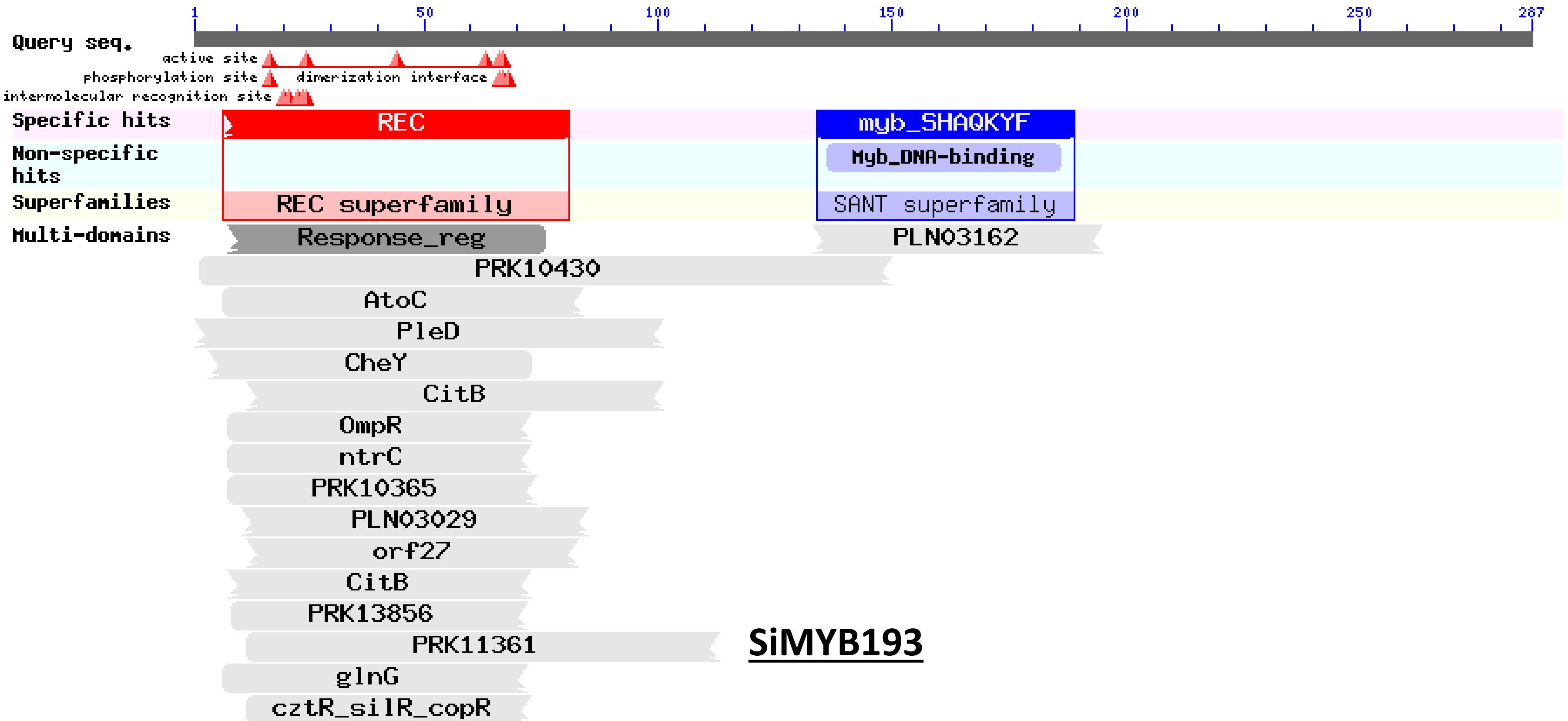

SiMYB193

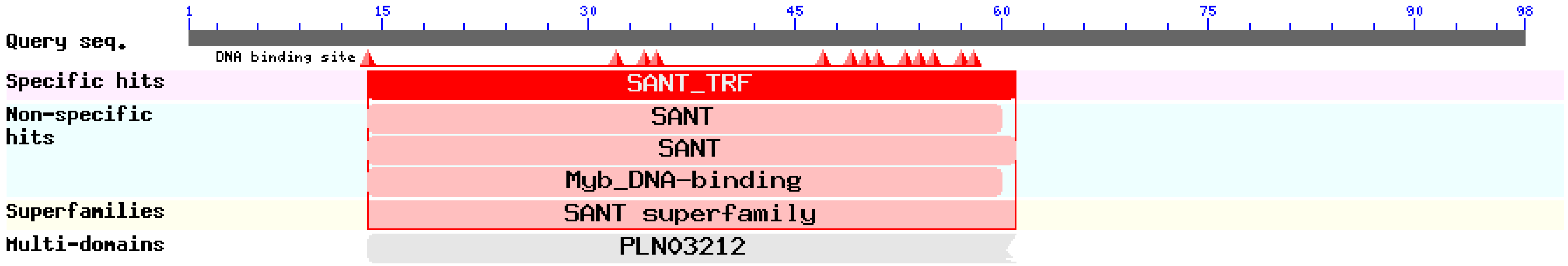

SiMYB194

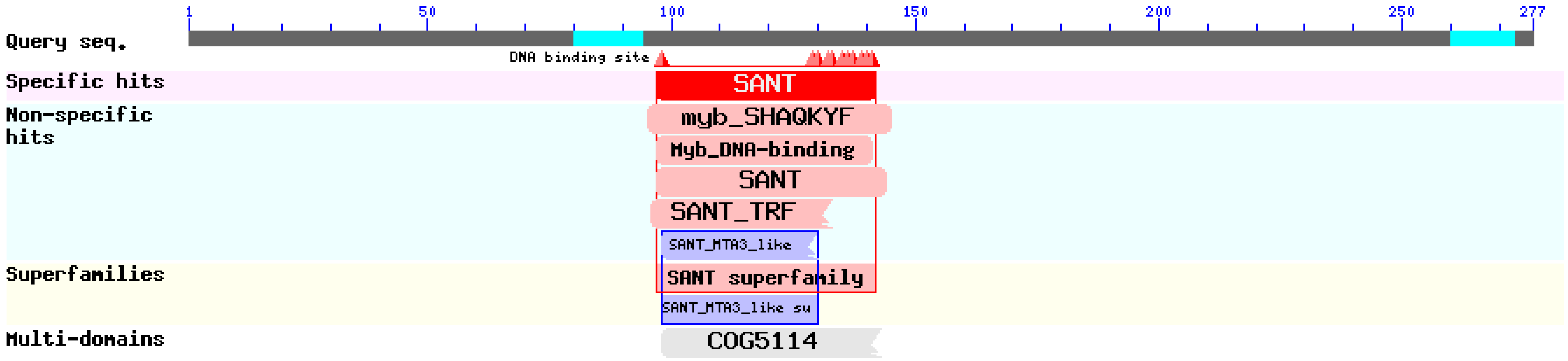

SiMYB195

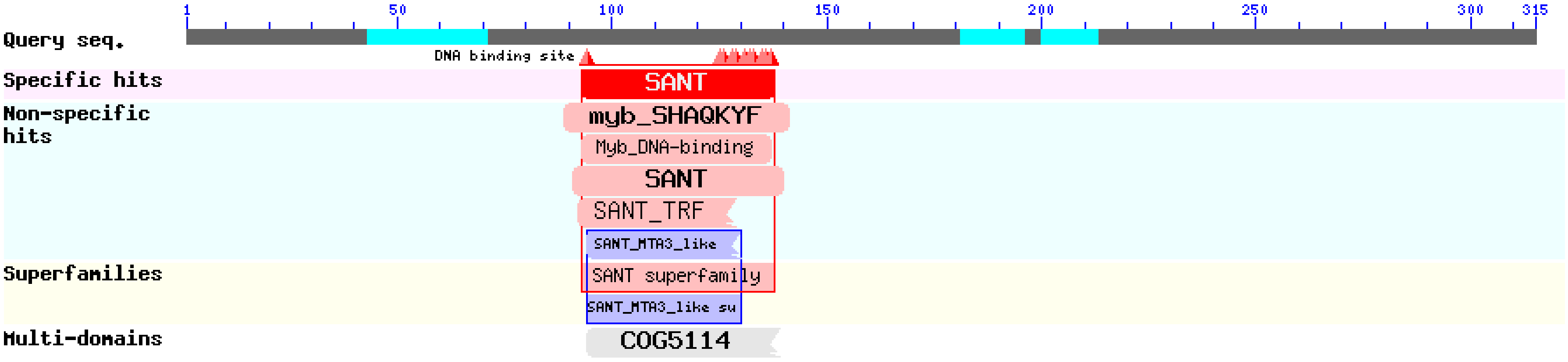

SiMYB196

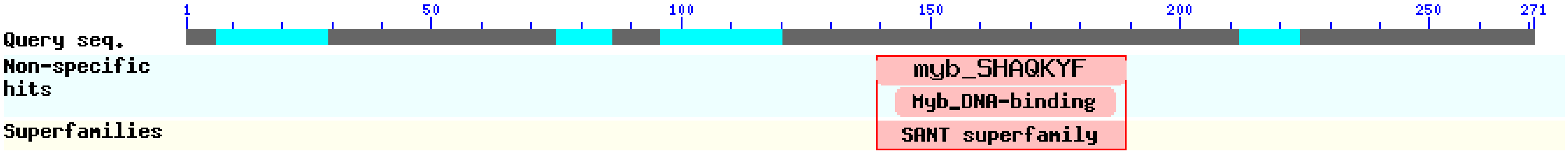

SiMYB197

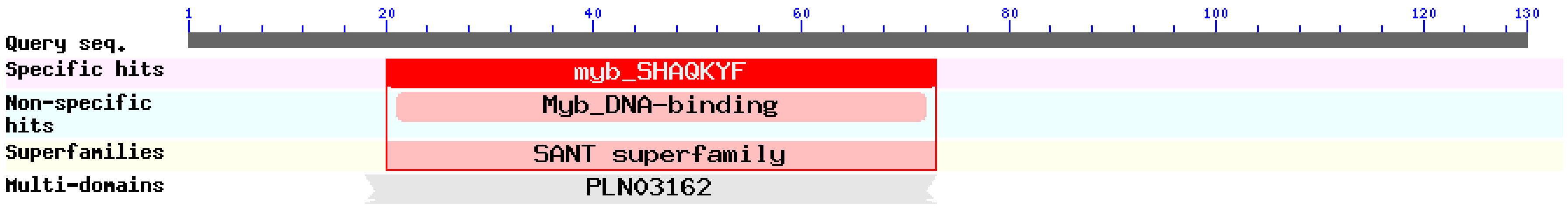

SiMYB198

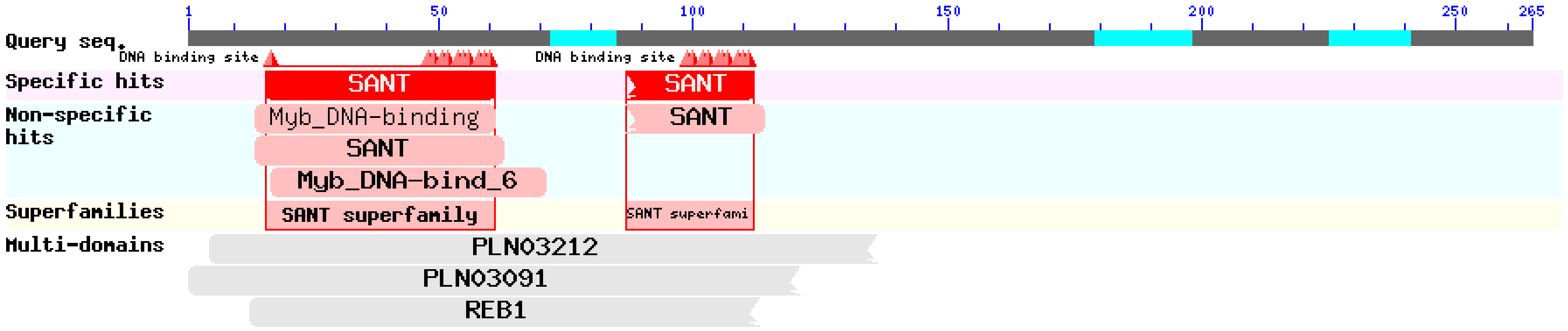

SiMYB199

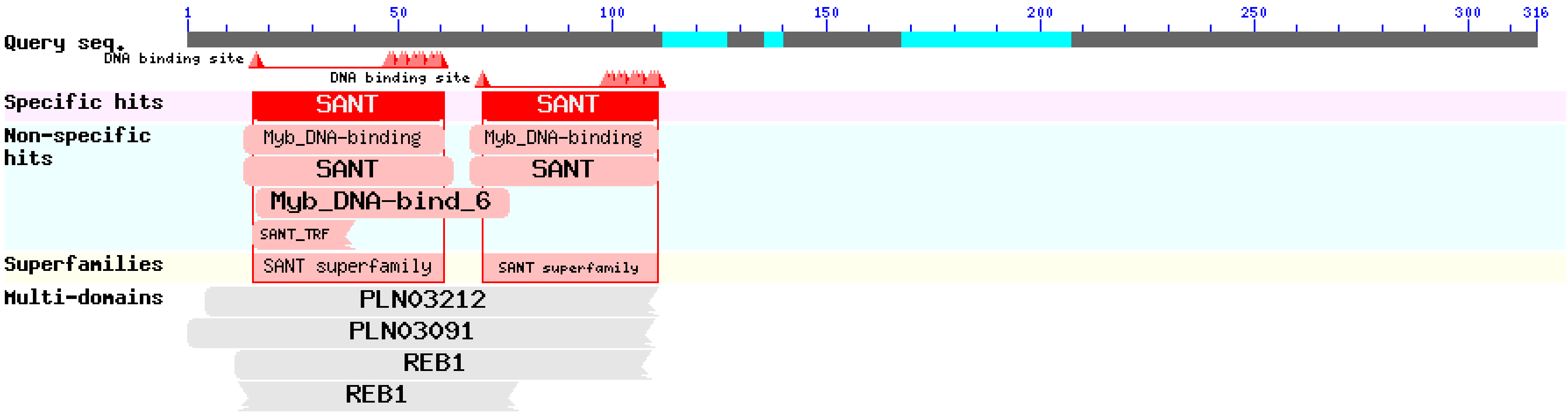

SiMYB200

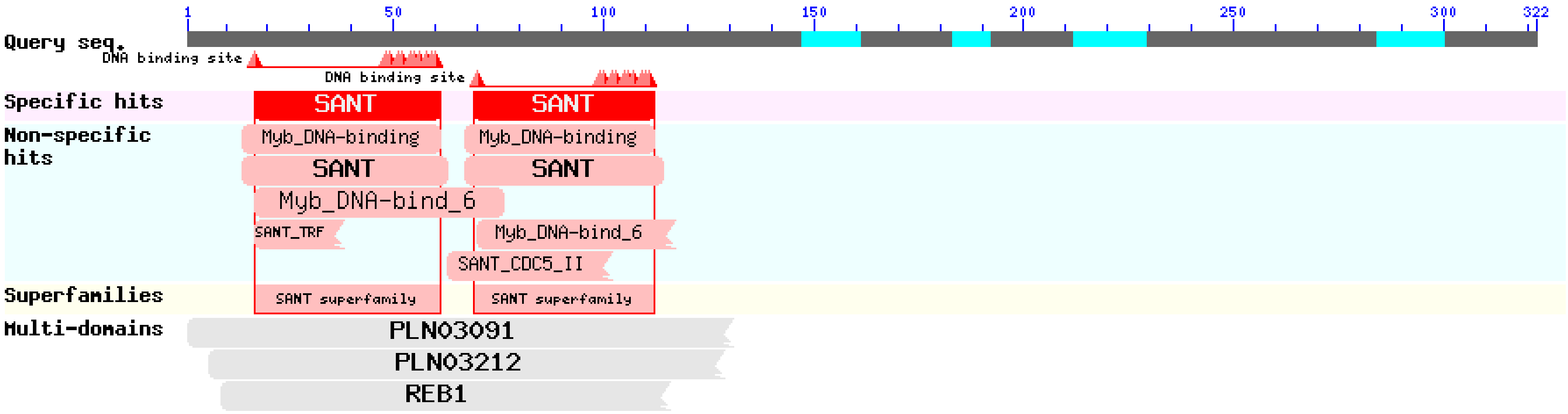

SiMYB201

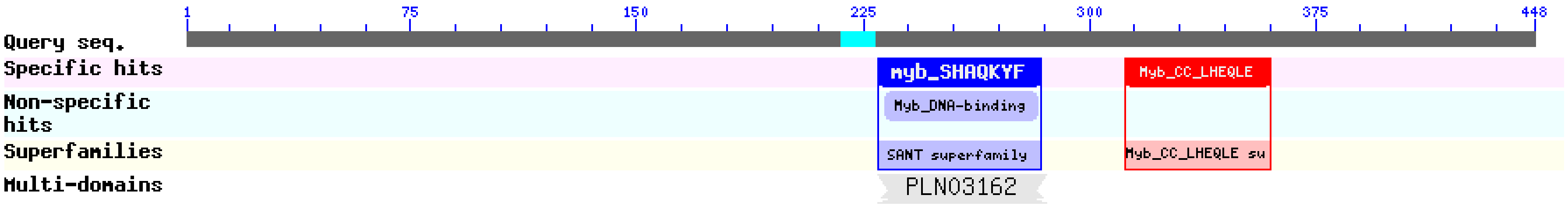

SiMYB202

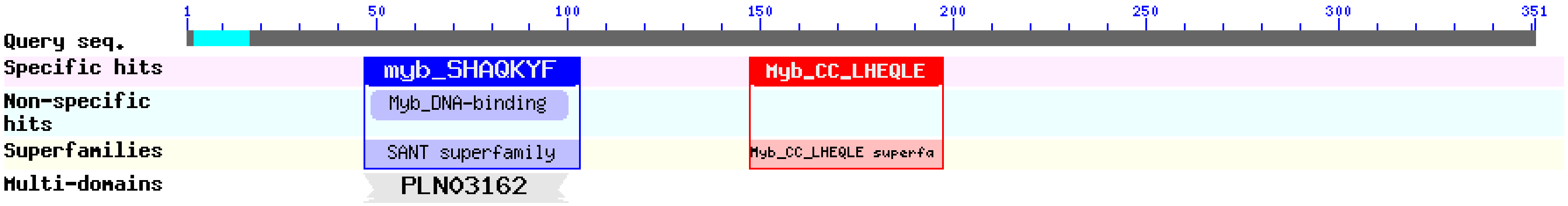

SiMYB203

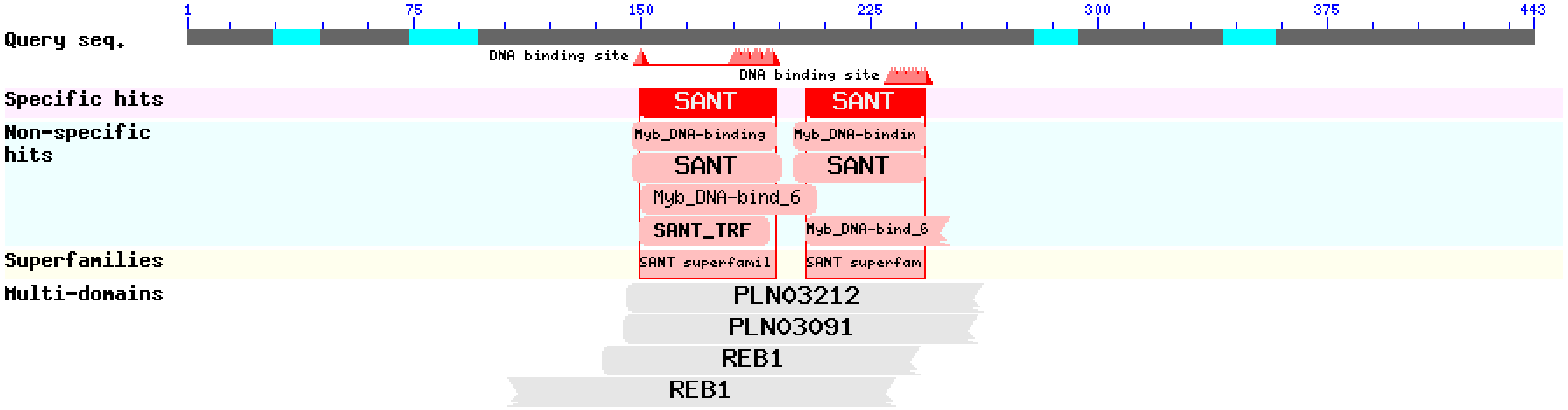

SiMYB204

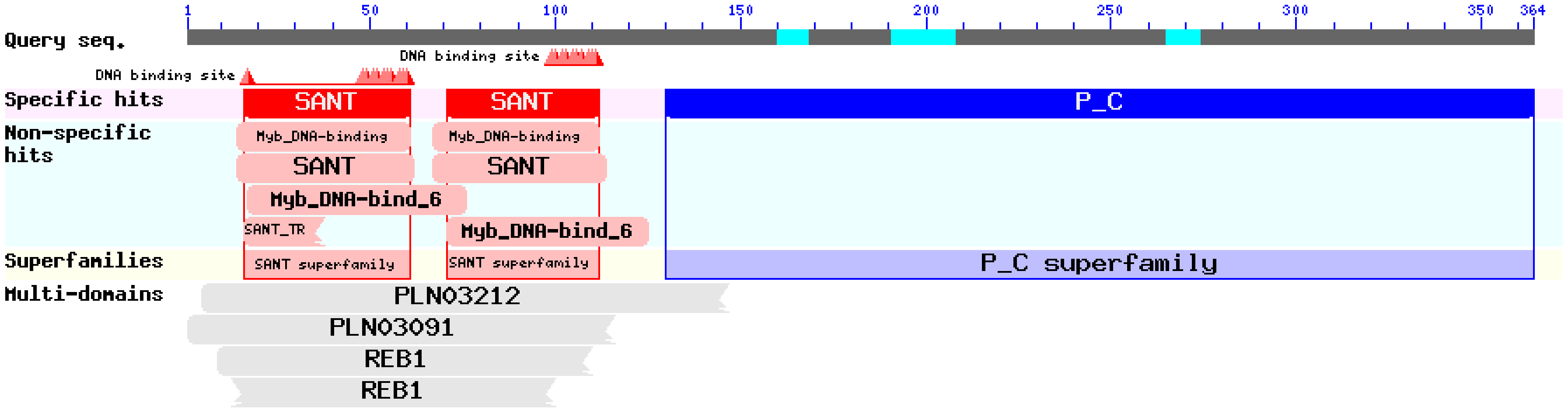

SiMYB205

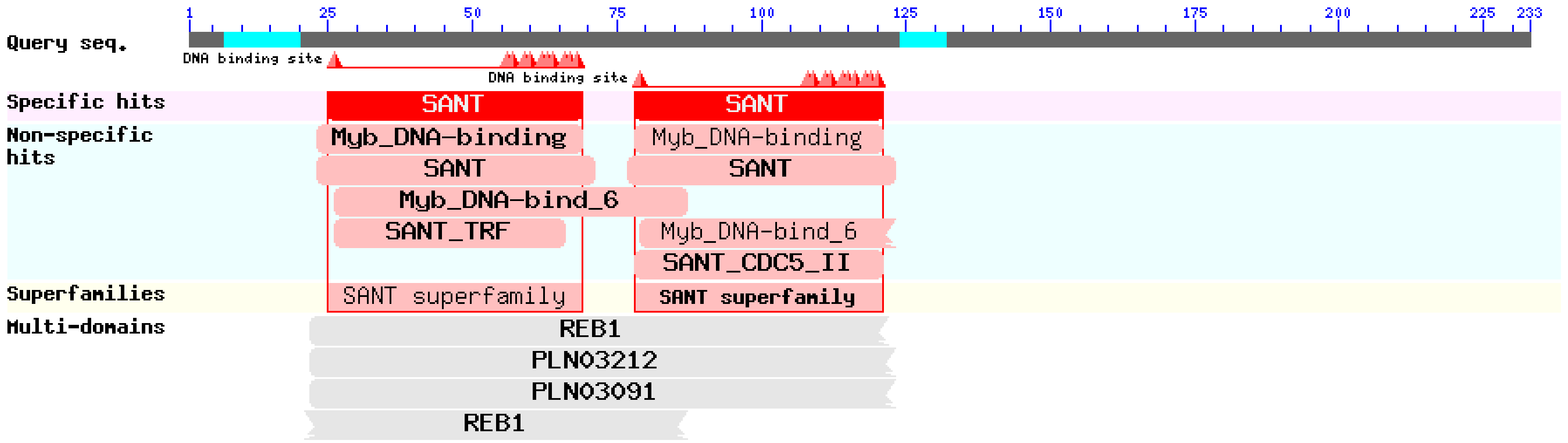

SiMYB206

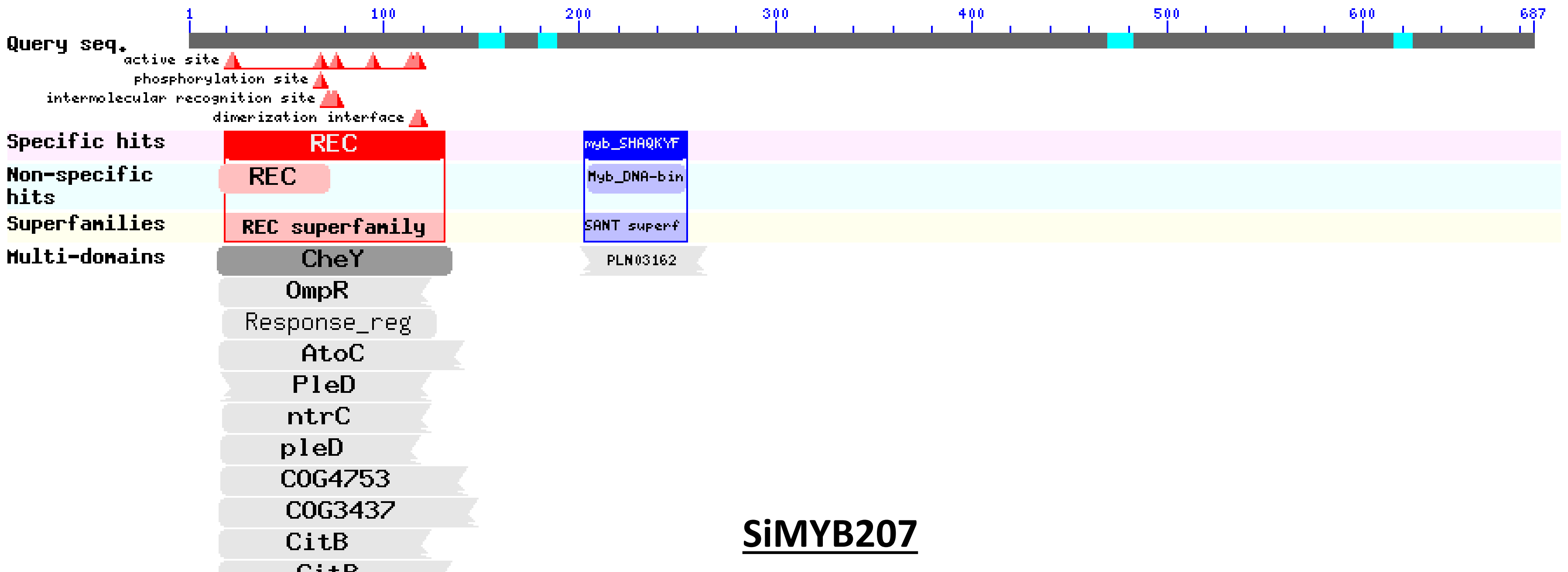

SiMYB207

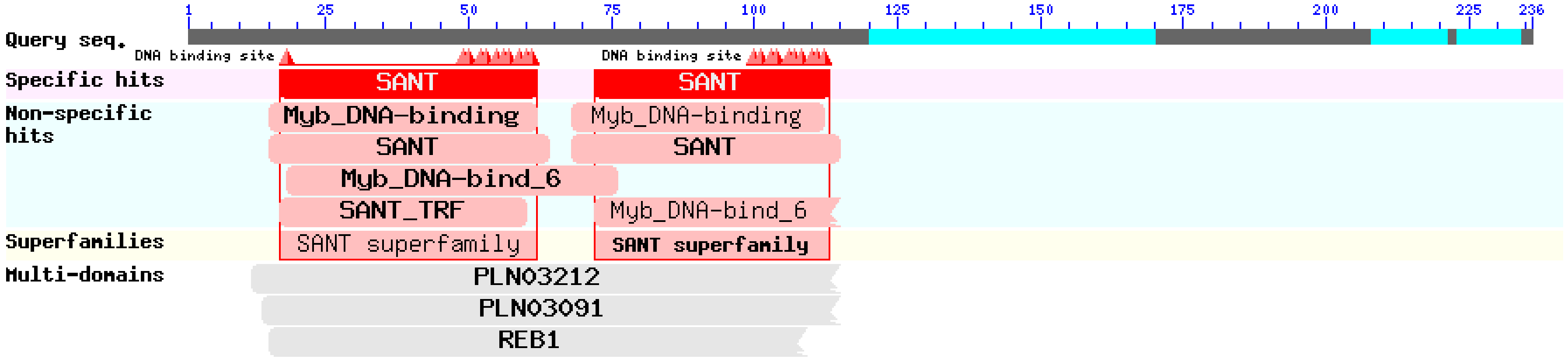

SiMYB208

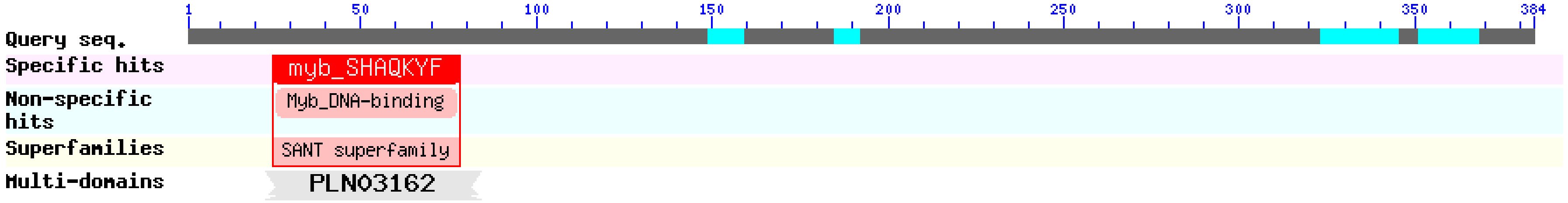

SiMYB209
