# Supplementary material for: Identification and Molecular Characterization of MYB Transcription Factor Superfamily in C4 Model Plant Foxtail Millet (Setaria italica L.)
Source: PLoS One. 2014 Oct 3;9(10):e109920. doi: 10.1371/journal.pone.0109920 (PMC4184890; doi:10.1371/journal.pone.0109920)
Supplement: Figure S3 — The multiple sequence alignment of ‘MYB-related’ proteins. (PDF) [file pone.0109920.s003.pdf]

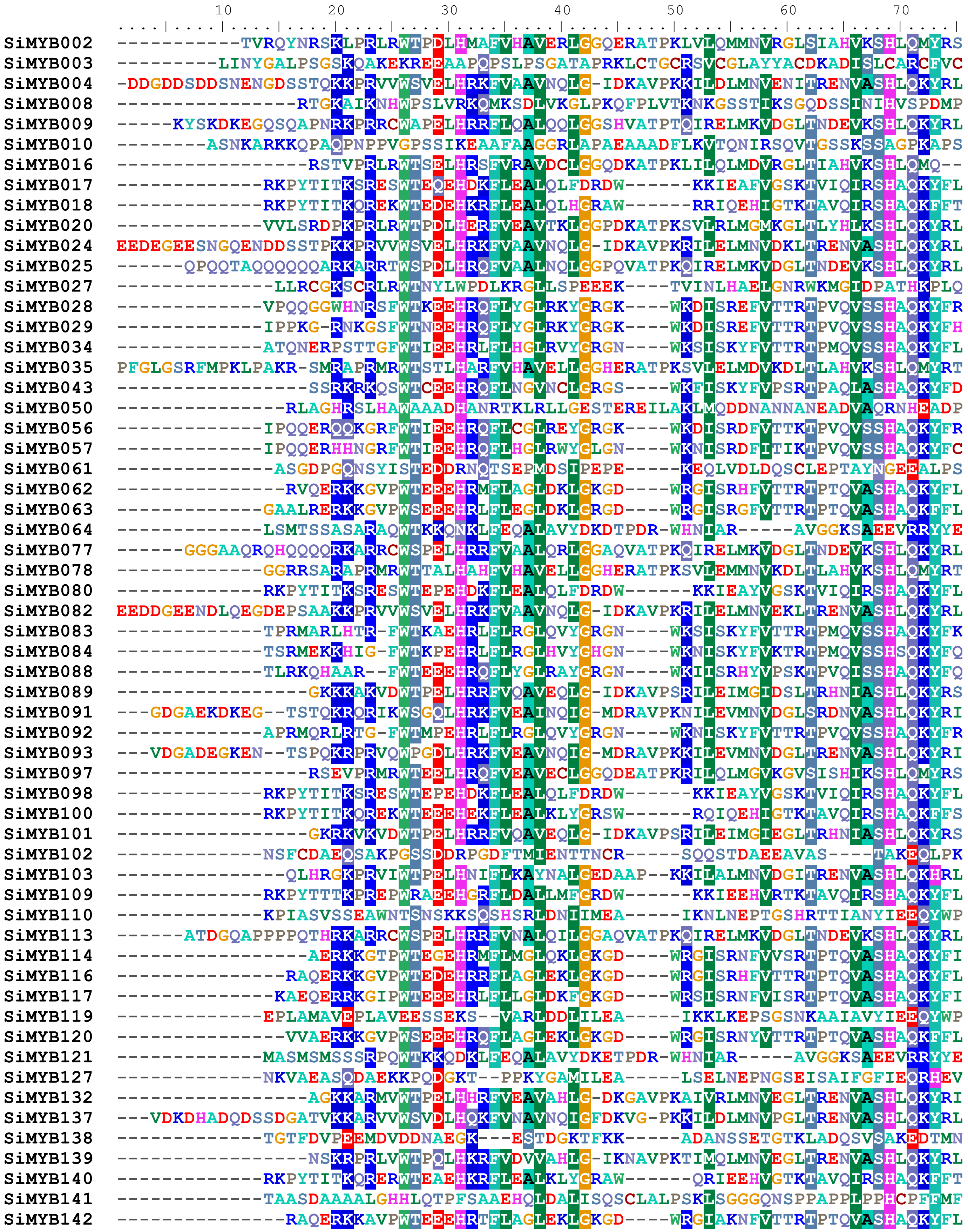

10 20 30 40 50 60 70
SiMYB002 -----------TVRQYNRSKLPRLRWTPDLHMAFVHAVERLGGQERATPKLVLQMMNVRGLSIAHVKSHLQMYRS
SiMYB003 ---------LINYGALPSGSKQAKEKREEAAPQPSLPSGATAPRKLCTGCRSVCGLAYYACDKADISLCARCFVC
SiMYB004 -DDGDDSDDSNENGDSSTQKKPRVVWSVELHRKFVAAVNQLG-IDKAVPKKILDLMNVENITRENVASHLQKYRL
SiMYB008 ----------------RTGKAIKNHWPSLVRKQMKSDLVKGLPKQFPLVTKNKGSSTIKSGQDSSINIHVSPDMP
SiMYB009 -----KYSKDKEGQSQAPNRKPRRCWAPELHRRFLQALQQLGGSHVATPTQIRELMKVDGLTNDEVKSHLQKYRL
SiMYB010 --------ASNKARKKQPAQPNPPVGPSSIKEAAFAAGGRLAPAEAAADFLKVTQNIRSQVTGSSKSSAGPKAPS
SiMYB016 -----------------RSTVPRLRWTSELHRSFVRAVDCLGGQDKATPKLILQLMDVRGLTIAHVKSHLQMQ--
SiMYB017 -------------RKPYTITKSRESWTEQEHDKFLEALQLFDRDW------KKIEAFVGSKTVIQIRSHAQKYFL
SiMYB018 -------------RKPYTITKQREKWTEDEHKRFLEALQLHGRAW------RRIQEHIGTKTAVQIRSHAQKFFT
SiMYB020 -------------VVLSRDPKPRLRWTPDLHERFVEAVTKLGGPDKATPKSVLRLMGMKGLTLYHLKSHLQKYRL
SiMYB024 EEDEGEESNGQENDDSSTPKKPRVVWSVELHRKFVAAVNQLG-IDKAVPKRILELMNVDKLTRENVASHLQKYRL
SiMYB025 ------QPQQTAQQQQQQARKARRTWSPDLHRQFVAALNQLGGPQVATPKQIRELMKVDGLTNDEVKSHLQKYRL
SiMYB027 --------------LLRCGKSCRLRWTNYLWPDLKRGLLSPEEEK----TVINLHAELGNRWKMGIDPATHKPLQ
SiMYB028 -------------VPQQGGWHNRSFWTKEEHRQFLYGLRKYGRGK----WKDISREFVTTRTPVQVSSHAQKYFR
SiMYB029 -------------IPPKG-RNKGSFWTNEEHRQFLYGLRKYGRGK----WKDISREFVTTRTPVQVSSHAQKYFH
SiMYB034 -------------ATQNERPSTTGFWTIEEHRLFLHGLRVYGRGN----WKSISKYFVTTRTPMQVSSHAQKYFL
SiMYB035 PFGLGSRFMPKLPAKR-SMRAPRMRWTSTLHARFVHAVELLGGHERATPKSVLELMDVKDLTLAHVKSHLQMYRT
SiMYB043 -----------------SSRKRKQSWTCEEHRQFLNGVNCLGRGS----WKFISKYFVPSRTPAQLASHAQKYFD
SiMYB050 ---------------RLAGHRSLHAWAAADHANRTKLRLLGESTEREILAKLMQDDNANNANEADVAQRNHEADP
SiMYB056 -------------IPQQERQQKGRFWTIEEHRQFLCGLREYGRGK----WKDISRDFVTTKTPVQVSSHAQKYFR
SiMYB057 -------------IPQQERHHNGRFWTIEEHRQFLHGLRWYGLGN----WKNISRDFITIKTPVQVSSHAQKYFC
SiMYB061 --------------ASGDPGQNSYISTEDDRNQTSEPMDSIPEPE-----KEQLVDLDQSCLEPTAYNGEEALPS
SiMYB062 ---------------RVQERKKGVPWTEEEHRMFLAGLDKLGKGD----WRGISRHFVTTRTPTQVASHAQKYFL
SiMYB063 -------------GAALRERKKGVPWSEEEHRLFLEGLDKLGRGD----WRGISRGFVTTRTPTQVASHAQKFFL
SiMYB064 -------------LSMTSSASARAQWTKKQNKLFEQALAVYDKDTPDR-WHNIAR-----AVGGKSAEEVRRYYE
SiMYB077 ------GGGAAQRQHQQQQRKARRCWSPELHRRFVAALQRLGGAQVATPKQIRELMKVDGLTNDEVKSHLQKYRL
SiMYB078 -------------GGRRSARAPRMRWTTALHAHFVHAVELLGGHERATPKSVLEMMNVKDLTLAHVKSHLQMYRT
SiMYB080 -------------RKPYTITKSRESWTEPEHDKFLEALQLFDRDW------KKIEAYVGSKTVIQIRSHAQKYFL
SiMYB082 EEDDGEENDLQEGDEPSAAKKPRVVWSVELHRKFVAAVNQLG-IDKAVPKRILELMNVEKLTRENVASHLQKYRL
SiMYB083 -------------TPRMARLHTR-FWTKAEHRLFLRGLQVYGRGN----WKSISKYFVTTRTPMQVSSHAQKYFK
SiMYB084 -------------TSRMEKKHIG-FWTKPEHRLFLRGLHVYGHGN----WKNISKYFVKTRTPMQVSSHSQKYFQ
SiMYB088 -------------TLRKQHAAR--FWTEEEHRQFLYGLRAYGRGN----WKIISRHYVPSKTPVQIISHAQKYFQ
SiMYB089 -----------------GKKKAKVDWTPELHRRFVQAVEQLG-IDKAVPSRILEIMGIDSLTRHNIASHLQKYRS
SiMYB091 ---GDGAEKDKEG--TSTQKRQRIKWSGQLHRKFVEAINQIG-MDRAVPKNILEVMNVDGLSRDNVASHLQKYRI
SiMYB092 -------------APRMQRLRTG-FWTMPEHRLFLRGLQVYGRGN----WKNISKYFVTTRTPVQVSSHAQKYFR
SiMYB093 ---VDGADEGKEN--TSPQKRPRVQWPGDLHRKFVEAVNQIG-MDRAVPKKILEVMNVDGLTRENVASHLQKYRI
SiMYB097 -----------------RSEVPRMRWTEELHRQFVEAVECLGGQDEATPKRILQLMGVKGVSISHIKSHLQMYRS
SiMYB098 -------------RKPYTITKSRESWTEPEHDKFLEALQLFDRDW------KKIEAYVGSKTVIQIRSHAQKYFL
SiMYB100 -------------RKPYTITKQREKWTEEEHEKFLEALKLYGRSW------RQIQEHIGTKTAVQIRSHAQKFFS
SiMYB101 -----------------GKRKVKVDWTPELHRRFVQAVEQLG-IDKAVPSRILEIMGIEGLTRHNIASHLQKYRS
SiMYB102 -------------NSFCDAEQSAKPGSSDDRPGDFTMIENTTNCR-----SQQSTDAEEAVAS----TAKEQLPK
SiMYB103 ---------------QLHRGKPRVIWTPELHNIFLKAYNALGEDAAP--KKILALMNVDGITRENVASHLQKHRL
SiMYB109 -------------RKPYTTTKPREPWRAEEHGRFLDALLMFGRDW------KKIEEHVRTKTAVQIRSHAQKYFL
SiMYB110 -------------KPIASVSSEAWNTSNSKKSQSHSRLDNIIMEA-----IKNLNEPTGSHRTTIANYIEEQYWP
SiMYB113 ------ATDGQAPPPPQTHRKARRCWSPELHRRFVNALQILGGAQVATPKQIRELMKVDGLTNDEVKSHLQKYRL
SiMYB114 -----------------AERKKGTPWTEGEHRMFLMGLQKLGKGD----WRGISRNFVVSRTPTQVASHAQKYFI
SiMYB116 ---------------RAQERKKGVPWTEDEHRRFLAGLEKLGKGD----WRGISRHFVTTRTPTQVASHAQKYFL
SiMYB117 --------------KAEQERRKGIPWTEEEHRLFLLGLDKFGKGD----WRSISRNFVISRTPTQVASHAQKYFI
SiMYB119 -------------EPLAMAVEPLAVEESSEKS--VARLDDLILEA-----IKKLKEPSGSNKAAIAVYIEEQYWP
SiMYB120 ---------------VVAERKKGVPWSEEEHRQFLAGLEKLGKGD----WRGISRNYVTTRTPTQVASHAQKFFL
SiMYB121 -------------MASMSMSSSRPQWTKKQDKLFEQALAVYDKETPDR-WHNIAR-----AVGGKSAEEVRRYYE
SiMYB127 -------------NKVAEASQDAEKKPQDGKT--PPKYGAMILEA-----LSELNEPNGSEISAIFGFIEQRHEV
SiMYB132 -----------------AGKKARMVWTPELHHRFVEAVAHLG-DKGAVPKAIVRLMNVEGLTRENVASHLQKYRI
SiMYB137 ---VDKDHADQDSSDGATVKKARVVWSVDLHQKFVNAVNQIGFDKVG-PKKILDLMNVPGLTRENVASHLQKYRL
SiMYB138 -------------TGTFDVPEEMDVDDNAEGK---ESTDGKTFKK-----ADANSSETGTKLADQSVSAKEDTMN
SiMYB139 -----------------NSKRPRLVWTPQLHKRFVDVVAHLG-IKNAVPKTIMQLMNVEGLTRENVASHLQKYRL
SiMYB140 -------------RKPYTITKQRERWTEAEHKRFLEALKLYGRAW------QRIEEHVGTKTAVQIRSHAQKFFT
SiMYB141 -------------TAASDAAAALGHHLQTPFSAAEHQLDALISQSCLALPSKLSGGGQNSPPAPPLPPHCPFEMF
SiMYB142 ---------------RAQERKKAVPWTEEEHRTFLAGLEKLGKGD----WRGIAKNFVTTRTPTQVASHAQKYFL
