# Supplementary material for: Identification and Molecular Characterization of MYB Transcription Factor Superfamily in C4 Model Plant Foxtail Millet (Setaria italica L.)
Source: PLoS One. 2014 Oct 3;9(10):e109920. doi: 10.1371/journal.pone.0109920 (PMC4184890; doi:10.1371/journal.pone.0109920)
Supplement: Figure S4 — The multiple sequence alignment of ‘MYB-R2R3’ proteins. (PDF) [file pone.0109920.s004.pdf]

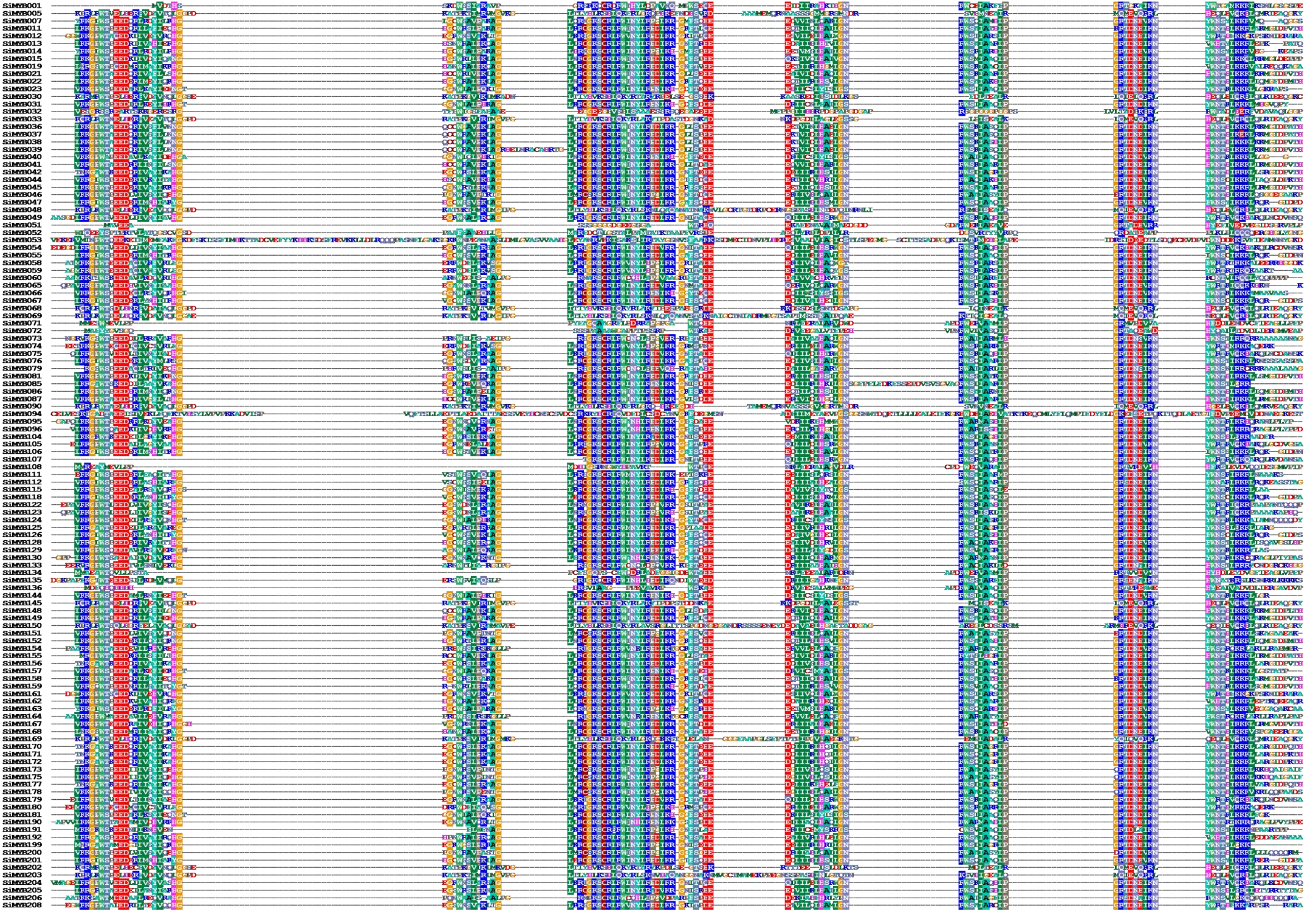
SiMYB001
SiMYB005
SiMYB007
SiMYB011
SiMYB012
SiMYB013
SiMYB014
SiMYB015
SiMYB019
SiMYB021
SiMYB022
SiMYB023
SiMYB030
SiMYB031
SiMYB032
SiMYB033
SiMYB036
SiMYB037
SiMYB038
SiMYB039
SiMYB040
SiMYB041
SiMYB042
SiMYB044
SiMYB045
SiMYB046
SiMYB047
SiMYB048
SiMYB049
SiMYB051
SiMYB052
SiMYB053
SiMYB054
SiMYB055
SiMYB058
SiMYB059
SiMYB060
SiMYB065
SiMYB066
SiMYB067
SiMYB068
SiMYB069
SiMYB071
SiMYB072
SiMYB073
SiMYB074
SiMYB075
SiMYB076
SiMYB079
SiMYB081
SiMYB085
SiMYB086
SiMYB087
SiMYB090
SiMYB094
SiMYB095
SiMYB096
SiMYB104
SiMYB105
SiMYB106
SiMYB107
SiMYB108
SiMYB111
SiMYB112
SiMYB115
SiMYB118
SiMYB122
SiMYB123
SiMYB124
SiMYB125
SiMYB126
SiMYB128
SiMYB129
SiMYB130
SiMYB133
SiMYB134
SiMYB135
SiMYB136
SiMYB144
SiMYB145
SiMYB148
SiMYB149
SiMYB150
SiMYB151
SiMYB152
SiMYB154
SiMYB155
SiMYB156
SiMYB157
SiMYB158
SiMYB159
SiMYB161
SiMYB162
SiMYB163
SiMYB164
SiMYB167
SiMYB168
SiMYB169
SiMYB170
SiMYB171
SiMYB172
SiMYB173
SiMYB175
SiMYB177
SiMYB178
SiMYB179
SiMYB180
SiMYB181
SiMYB190
SiMYB191
SiMYB192
SiMYB199
SiMYB200
SiMYB201
SiMYB202
SiMYB203
SiMYB204
SiMYB205
SiMYB206
SiMYB208
