# Supplementary material for: Identification and Molecular Characterization of MYB Transcription Factor Superfamily in C4 Model Plant Foxtail Millet (Setaria italica L.)
Source: PLoS One. 2014 Oct 3;9(10):e109920. doi: 10.1371/journal.pone.0109920 (PMC4184890; doi:10.1371/journal.pone.0109920)
Supplement: Figure S5 — The multiple sequence alignment of ‘MYB-R1R2R3’ proteins. (PDF) [file pone.0109920.s005.pdf]

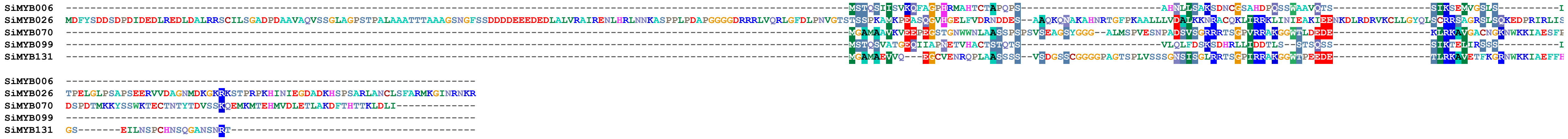
SiMYB006
SiMYB026
SiMYB070
SiMYB099
SiMYB131
