# Supplementary material for: Identification and Molecular Characterization of MYB Transcription Factor Superfamily in C4 Model Plant Foxtail Millet (Setaria italica L.)
Source: PLoS One. 2014 Oct 3;9(10):e109920. doi: 10.1371/journal.pone.0109920 (PMC4184890; doi:10.1371/journal.pone.0109920)
Supplement: Table S1 — Details of primers used for quantitative real-time PCR. (DOC) [file pone.0109920.s008.doc]

**Table S1.** List of primers used in quantitative real time-PCR expression analysis of *SiMYB* genes.

| **NAME** | **FORWARD PRIMERS (5′- 3′)** | **REVERSE PRIMERS (5′- 3′)** |
| --- | --- | --- |
| *SiMYB003* | TGGCTTCCTTCCCTCTAATC | ATTCCAAACCATTACCGCTT |
| *SiMYB041* | ATGTTGCTTTTTATCCCTTT | ACACCACCTAATCACCCACC |
| *SiMYB074* | GCTGGCTGGAAGATGGAT | GCCAACTCCTTCACCGTAAC |
| *SiMYB100* | GCCCCTTGTTCCAGCGTG | ACCTTTCTTCAGTCTACATTTGC |
| *SiMYB124* | ACCAACCAGTCAGTCAGTCA | GGGAAGGAAGAGCGAGCC |
| *SiMYB126* | CGAGTTCTTCTGAGGTTTTC | TAAGGAGGAGTGAGGACGA |
| *SiMYB150* | GTTCTTGTGTTTCATCTACC | GCTACGACAGTTCACAGTTCT |
| *SiMYB174* | TGCTGGGTTATCTCTTTCTTTG | CTTCTTCCCCTTTATCCAT |
| *SiMYB177* | GTGTGCCTGTGCCTGAAC | ATCTGCCCCCTTCCCTCC |
| *SiMYB184* | TGCCAAAAAAACCAAATA | GCTACACATTCTCGTTGG |
| *SiMYB202* | ATGCGTAAGTCTATTGAGT | TACACACGGCTACCAGAAGG |
| *Actin2* | CGCATATGTGGCTCTTGACT | GGGCACCTAAATCTCTCTGC |
