# Supplementary material for: Identification and Molecular Characterization of MYB Transcription Factor Superfamily in C4 Model Plant Foxtail Millet (Setaria italica L.)
Source: PLoS One. 2014 Oct 3;9(10):e109920. doi: 10.1371/journal.pone.0109920 (PMC4184890; doi:10.1371/journal.pone.0109920)
Supplement: Table S11 — The Ka/Ks ratios and estimated divergence time for segmentally duplicated SiMYB genes. (DOC) [file pone.0109920.s018.doc]

| **Table S11.** The Ka/Ks ratios and estimated divergence time for segmentally duplicated *SiMYB* genes. | | | | | | | | | | |  |  |  |
| --- | --- | --- | --- | --- | --- | --- | --- | --- | --- | --- | --- | --- | --- |
| **Gene 1** | **Chromosome** | **Start** | **End** | **Gene 2** | **Chromosome** | **Start** | **End** | **Ks** | **Ka** | **Ka/Ks** | | | **Mya** |
|
| SiMYB001 | 1 | 520710 | 522991 | SiMYB181 | 9 | 4382284 | 4383587 | 0.44 | 0.03 | 0.07 | | | 33.8 |
| SiMYB002 | 1 | 1864051 | 1866143 | SiMYB182 | 9 | 4604006 | 4608506 | 0.43 | 0.02 | 0.05 | | | 33.1 |
| SiMYB003 | 1 | 5001092 | 5004976 | SiMYB183 | 9 | 4727729 | 4729778 | 0.4 | 0.03 | 0.08 | | | 30.8 |
| SiMYB007 | 1 | 10216744 | 10218257 | SiMYB087 | 4 | 10929498 | 10930771 | 0.44 | 0.02 | 0.05 | | | 33.8 |
| SiMYB008 | 1 | 10409810 | 10411875 | SiMYB088 | 4 | 11918439 | 11919643 | 0.41 | 0.04 | 0.10 | | | 31.5 |
| SiMYB167 | 7 | 28720650 | 28721770 | SiMYB205 | 9 | 48621411 | 48625382 | 0.47 | 0.04 | 0.09 | | | 36.2 |
| SiMYB168 | 7 | 28765322 | 28766641 | SiMYB204 | 9 | 48079232 | 48081675 | 0.4 | 0.01 | 0.03 | | | 30.8 |
| SiMYB169 | 7 | 28947148 | 28949550 | SiMYB203 | 9 | 47609623 | 47612326 | 0.47 | 0.04 | 0.09 | | | 36.2 |
| SiMYB170 | 7 | 33127802 | 33128675 | SiMYB202 | 9 | 47319386 | 47325300 | 0.45 | 0.01 | 0.02 | | | 34.6 |
| SiMYB171 | 7 | 33135455 | 33136305 | SiMYB201 | 9 | 45414214 | 45416012 | 0.39 | 0.05 | 0.13 | | | 30.0 |
| SiMYB172 | 7 | 33146761 | 33147523 | SiMYB200 | 9 | 44272175 | 44273137 | 0.43 | 0.02 | 0.05 | | | 33.1 |
| SiMYB173 | 7 | 34503168 | 34504218 | SiMYB199 | 9 | 42813187 | 42815090 | 0.42 | 0.02 | 0.05 | | | 32.3 |
|  |  |  |  |  |  |  | **Mean** | 0.43 | 0.03 | 0.06 | | | 33.01 |
