# Supplementary material for: Identification and Molecular Characterization of MYB Transcription Factor Superfamily in C4 Model Plant Foxtail Millet (Setaria italica L.)
Source: PLoS One. 2014 Oct 3;9(10):e109920. doi: 10.1371/journal.pone.0109920 (PMC4184890; doi:10.1371/journal.pone.0109920)
Supplement: Table S12 — The Ka/Ks ratios and estimated divergence time for tandemly duplicated SiMYB genes. (DOC) [file pone.0109920.s019.doc]

| **Table S12.** The Ka/Ks ratios and estimated divergence time for tandemly duplicated *SiMYB* genes. | | | | | | | | | | |  |  | |  |  |
| --- | --- | --- | --- | --- | --- | --- | --- | --- | --- | --- | --- | --- | --- | --- | --- |
| **Gene 1** | **Chromosome** | **Start** | **End** | **Gene 2** | **Chromosome** | **Start** | **End** | **Distance (bp)** | **Ks** | **Ka** | | | **Ka/Ks** | | **Mya** |
|
| SiMYB036 | 2 | 29895281 | 29896084 | SiMYB038 | 2 | 29913361 | 29914295 | 17277 | 0.48 | 0.05 | | | 0.10 | | 36.92 |
| SiMYB037 | 2 | 29903880 | 29904653 | SiMYB039 | 2 | 29921746 | 29922742 | 17093 | 0.45 | 0.06 | | | 0.13 | | 34.62 |
| SiMYB085 | 4 | 7031008 | 7031928 | SiMYB086 | 4 | 7611005 | 7612122 | 579077 | 0.42 | 0.03 | | | 0.07 | | 32.31 |
| SiMYB140 | 6 | 4438639 | 4440257 | SiMYB142 | 6 | 5231810 | 5236257 | 791553 | 0.41 | 0.04 | | | 0.10 | | 31.54 |
| SiMYB141 | 6 | 5225925 | 5227824 | SiMYB143 | 6 | 5596458 | 5599488 | 368634 | 0.43 | 0.05 | | | 0.12 | | 33.08 |
| SiMYB158 | 7 | 21987239 | 21989689 | SiMYB159 | 7 | 22506782 | 22507888 | 517093 | 0.42 | 0.04 | | | 0.10 | | 32.31 |
|  |  |  |  |  |  |  |  | **Mean** | 0.44 | 0.05 | | | 0.10 | | 33.46 |
